# Development of a screening tool to predict the risk of chronic pain and disability following musculoskeletal trauma: protocol for a prospective observational study in the United Kingdom

**DOI:** 10.1136/bmjopen-2017-017876

**Published:** 2018-04-28

**Authors:** Alison B Rushton, David W Evans, Nicola Middlebrook, Nicola R Heneghan, Charlotte Small, Janet Lord, Jaimin M Patel, Deborah Falla

**Affiliations:** 1 Centre of Precision Rehabilitation for SpinalPain (CPR Spine), School of Sport, Exercise and Rehabilitation Sciences, College of Life and Environmental Sciences, University of Birmingham, Birmingham, UK; 2 NIHR Surgical Reconstruction and Microbiology Research Centre, University of Birmingham, Birmingham, UK

**Keywords:** musculoskeletal trauma, precision rehabilitation, pain mechanisms

## Abstract

**Introduction:**

Pain is an expected and appropriate experience following traumatic musculoskeletal injury. By contrast, chronic pain and disability are unhelpful yet common sequelae of trauma-related injuries. Presently, the mechanisms that underlie the transition from acute to chronic disabling post-traumatic pain are not fully understood. Such knowledge would facilitate the development and implementation of precision rehabilitation approaches that match interventions to projected risk of recovery, with the aim of preventing poor long-term outcomes. The aim of this study is to identify a set of predictive factors to identify patients at risk of developing ongoing post-traumatic pain and disability following acute musculoskeletal trauma. To achieve this, we will use a unique and comprehensive combination of patient-reported outcome measures, psychophysical testing and biomarkers.

**Methods and analysis:**

A prospective observational study will recruit two temporally staggered cohorts (n=250 each cohort; at least 10 cases per candidate predictor) of consecutive patients with acute musculoskeletal trauma aged ≥16 years, who are emergency admissions into a Major Trauma Centre in the United Kingdom, with an episode inception defined as the traumatic event. The first cohort will identify candidate predictors to develop a screening tool to predict development of chronic and disabling pain, and the second will allow evaluation of the predictive performance of the tool (validation). The outcome being predicted is an individual’s absolute risk of poor outcome measured at a 6-month follow-up using the Chronic Pain Grade Scale (poor outcome ≥grade II). Candidate predictors encompass the four primary mechanisms of pain: *nociceptive* (eg, injury location), *neuropathic* (eg, painDETECT), *inflammatory* (biomarkers) and *nociplastic* (eg, quantitative sensory testing). Concurrently, patient-reported outcome measures will assess general health and psychosocial factors (eg, pain self-efficacy). Risk of poor outcome will be calculated using multiple variable regression analysis.

**Ethics and dissemination:**

Approved by the NHS Research Ethics Committee (17/WA/0421).

**Strengths and limitations of this study**

- A comprehensive array of candidate predictive factors will allow for the prediction of chronic and disabling pain following trauma.
- These predictive factors will enable the development and validation of a predictive tool to predict good and poor outcome at 6 months postinjury.
- The prospective design of the study enables control of unwarranted influences and enables a stronger case for inferring causal relationships.
- Identifying predictive factors related to poor outcome of pain and disability outcome will facilitate targeting of effective interventions.
- Other candidate predictors may have been useful to include (eg, vibration thresholds), but consideration of burden to participants of testing and sample size considerations necessitated prioritisation of candidate predictive factors.

## Introduction

Pain is an expected and appropriate experience that usually follows traumatic injury.^1^ By contrast, chronic pain and disability are unhelpful but common sequelae of trauma-related injuries.^2^ Gaining an understanding of why some people develop chronic and disabling post-traumatic pain is therefore a priority for individual patients, the military and society at large. Notwithstanding, the mechanisms that underlie the transition from acute to chronic disabling post-traumatic pain are not fully understood. Such knowledge would facilitate the development and implementation of a clinical pathway of care that matches interventions to projected risk of poor recovery, with the aim of preventing poor long-term outcomes. This project stems from advances in knowledge relating to the assessment and management of pain^3^ and the quantification of potential predictive factors to inform personalised rehabilitation; identifying which patients to target with rehabilitation and when and how to target them.

Few studies have specifically explored predictive factors for recovery, whether poor or good, following physical trauma. Of those that have psychosocial variables, such as anxiety, depression and post-traumatic stress, have so far been identified as the strongest predictors of outcome.^4–7^ However, only a limited number of variables have hitherto been evaluated as potential predictive factors. Indeed, current opinion regarding pain mechanisms^8^ suggests that the development of chronic pain and disability cannot be entirely attributable to psychosocial factors. This is consistent with research in primary care that has identified predictive factors for poor outcome across a range of musculoskeletal pain conditions,^9^ which include: widespread pain, high functional disability, high pain intensity, long pain duration, high depression/anxiety, presence of previous pain episodes, movement restriction and poor coping strategies. Moreover, developments in the mechanistic understanding of pain^10–12^ suggest that other measures (eg, indicators of central sensitisation and inflammatory activity) may have potential predictive utility, especially in an acute trauma population.

### Aims of study

1. Using a unique combination of (1) general patient characteristics including premorbid neuropsychological status, (2) quality of life and physical functioning, (3) psychosocial features, (4) injury characteristics, (5) pain characteristics, (6) quantitative sensory testing and (7) biomarkers, we aim to find a set of predictive factors to identify patients at risk of developing ongoing post-traumatic pain and disability following acute musculoskeletal trauma. This will subsequently inform the feasibility of developing and evaluating a new clinical care pathway of precision rehabilitation that matches interventions to the predicted risk of poor recovery.

### Objectives

(1) Identify predictive factors for poor outcome (chronic pain and disability at 6 months postinjury) following acute musculoskeletal trauma. (2) Develop a predictive model to inform a screening tool to identify the predicted risk of poor recovery (transition from acute post-traumatic pain to chronic pain and disability). (3) Estimate the predictive performance of the screening tool through validation of the model in a separate dataset. (4) Document the clinical course of symptoms at 3 and 12 months following acute musculoskeletal trauma.

## Methods and analysis

### Source of data

**Figure 1:**
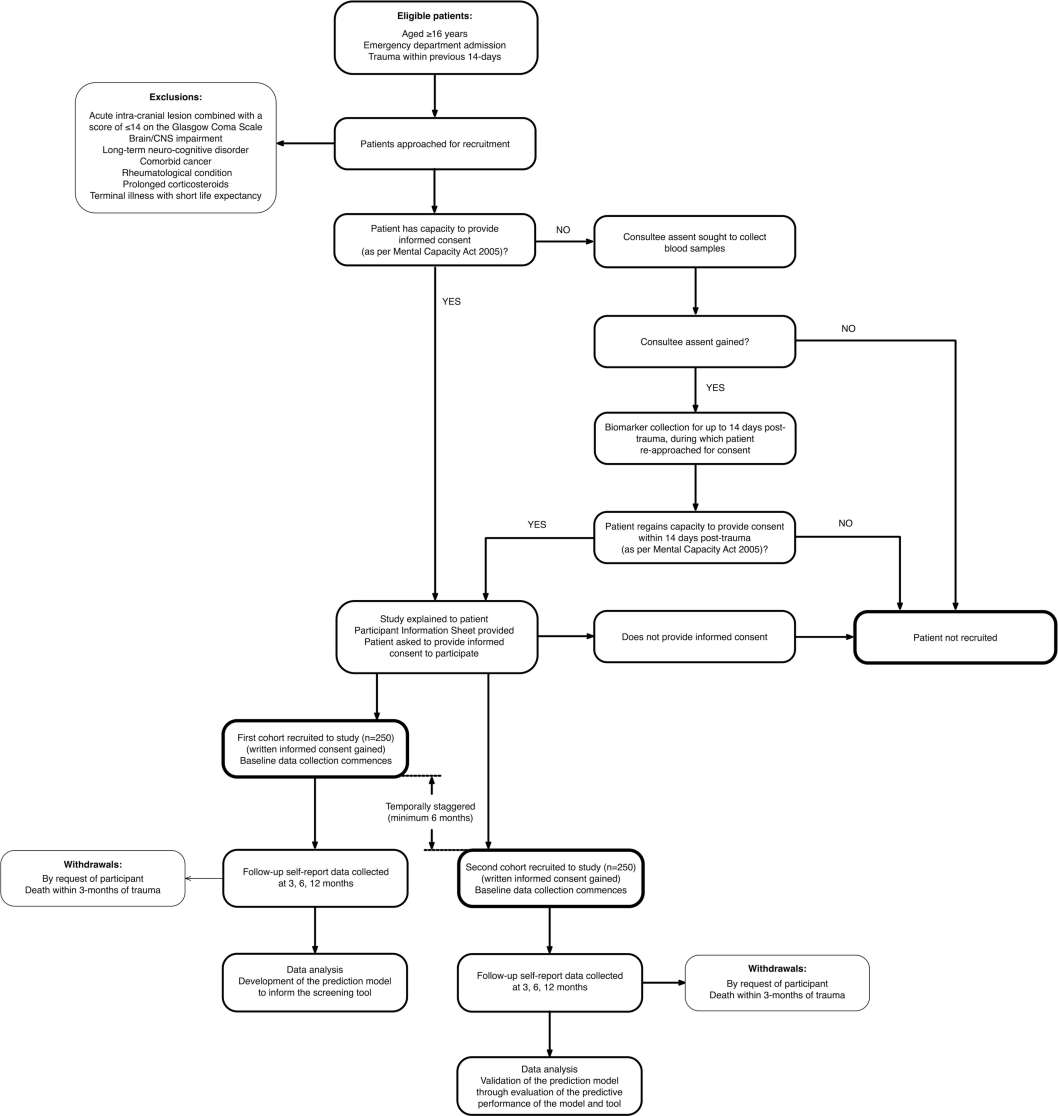
Study design. CNS, central nervous system.

The study will be a prospective, observational study of two temporally staggered cohorts of patients with trauma, who are emergency department admissions into a Major Trauma Centre in the United Kingdom, with an episode inception defined as the traumatic event (figure 1). The first cohort will facilitate development of the prediction model to inform the screening tool, and the second will enable validation of the prediction model through evaluation of the predictive performance of the model and tool.^13 14^ There will be an interval of at least 6 months between recruitment into the two respective cohorts. The prospective design enables control of unwarranted influences and enables a stronger case for inferring causal relationships. The nature of the study necessitates predictive statistical modelling.^15^ This protocol is written in line with the TRIPOD (transparent reporting of a multivariable prediction model for individual prognosis or diagnosis) statement,^16^ in which recommendations are given for the reporting of prediction model development and validation.

Self-reported and physical assessment predictive data will be collected at baseline over a period of up to 14 days (or duration of inpatient stay if shorter), which will commence immediately following recruitment. Biomarker data collection will occur throughout the same baseline period, but can commence prior to recruitment providing assent is gained from a legal consultee. The outcome data will be collected at 6 months postinjury (working definition of chronic pain)^17^, the point of evaluation of an individual’s absolute risk of poor outcome (objectives 1, 2 and 3). In addition, selected data will be measured at 3 and 12 months postinjury to explore the clinical course of recovery following injury in the shorter and longer terms (objective 4).

### Participants

Participants will be recruited from the register of a Major Trauma Centre in the United Kingdom for a period of up to 24 months (planned start date January 2018). All consecutive eligible patients will be approached for recruitment until the sample size is achieved.

### Eligibility criteria

Inclusion criteria: adult patients aged ≥16 years who are admitted to emergency department of the Major Trauma Centre, with their main criteria for admission being acute musculoskeletal trauma within the preceding 14 days, and a capacity to use and understand written and verbal English language and a mental capacity to provide informed consent (eg, no confusion, delirium, severe cognitive impairment or severe mental illness, defined by a score of ≤6 on the Abbreviated Mental Test).^18^ The primary reason for including patients with trauma occurring up to 14 days, is to be inclusive of patients who are critically ill and/or with diminished mental capacity initially following their trauma, patients requiring surgery as a result of the trauma and representative of the broad trauma population.

Exclusion criteria: exclusions will be made where the patient has an acute intracranial lesion (eg, bleed) combined with a score of ≤14 on the Glasgow Coma Scale^19^ (a 15-item measure of consciousness impairment with adequate reliability^20^ that is routinely taken in patients with trauma at hospital admission), where there is evident brain or central nervous system injury or impairment, long-term neurocognitive disorders (such as brain tumour, multiple sclerosis, Alzheimer’s and Parkinson’s diseases and so on), comorbid cancer, the presence of an ongoing rheumatological condition, prolonged use of corticosteroids or terminal illness with short life expectancy.

Withdrawals: participants will be informed that they are free to withdraw from the study at any time, without needing to provide reason. In the event of death within 3 months of being recruited, participants will be automatically withdrawn from the study and the primary predictive analysis. Baseline data of all withdrawn participants will be kept and compared with those of retained participants to assess for any differences.

### Recruitment

Based on feasibility data (site data from the Trauma Audit and Research Network), it is estimated that at least 1000 eligible patients with trauma will be approachable for recruitment over a 24-month period, and that 50% would be expected to consent to participation. A dedicated team of research nurses will be available to recruit patients 7 days per week (from 0700 to 1930).

Because of impairments resulting from their injuries, some otherwise eligible patients will lack the mental capacity to provide informed consent when first approached to enrol in the study. Recruitment into the study will therefore be undertaken under the guidance and provision of the (UK) Mental Capacity Act 2005 for research in emergency situations. If the patient lacks sufficient capacity to consent, written assent for study participation will be sought from a legal consultee to begin biomarker data collection (blood samples), with informed consent for full recruitment (and subsequent data collection) being sought from the patient only if, and when, they regain sufficient capacity to provide this. If the patient does not regain capacity to provide consent within 14 days of their trauma, they will not be recruited into the study, biomarker data collection will cease and any blood samples already collected will be destroyed before analysis.

Once informed consent is gained and the participant recruited, following a minimum 1 hour lead time after the informed consent process (to reduce patient burden), members of the research team will visit the patient at their bedside to collect baseline self-reported data via questionnaires (table 1). On the next available working day following completion of the questionnaires (again, to reduce patient burden), members of the study team will return to the patient to conduct the first physical (quantitative sensory testing) assessment. At each visit, if deemed necessary, the capacity of the participant will be checked using an Abbreviated Mental Test^18^ (a score of ≤6 is indicative of reduced capacity), and asked if they are happy to proceed with data collection.

**Table 1:** Summary of data collection at different assessment points

Domain/Candidate predictor	Measure/data item	Baseline *commencing ≤14 days post-trauma*	3-month *clinical course*	6-month *outcome assessment point/clinical course*	12-month *clinical course*
General patient characteristics including premorbid neuropsychological status					
Age	In years	√			
Gender	Female/male/other	√			
BMI	Calculated from height and weight measurements	√			
Education	Highest educational level attained	√			
Employment status	Full time/part time/not working/retired/student Employed/self-employed	√	√	√	√
Circumstance of trauma	Military/civilian	√			
Previous history of musculoskeletal pain and injury	Patient history data from patient recollection and medical records	√			
Comorbidity of other health problems	Patient history data from patient recollection and medical records	√			
Premorbid physical health	Patient history data from patient recollection and medical records	√			
Premorbid psychological health	Patient history data from medical records and patient recollection (including non-somatic items from the Subjective Health Complaints Inventory)^44^	√			
Number of days in hospital	Intensive care/ward/total	√			
Quality of life and physical functioning					
General health	SF-36v2^45^	√	√	√	√
Health-related quality of life	EuroQol EQ-5D-5L^46^	√	√	√	√
Self-care and mobility during activities of daily living	Barthel Index of Activities of Daily Living,^47^ collected from hospital data	√			
Sleep quality	11-point (0–10) Numerical Rating Scale, relating to current pain, from ‘best possible sleep’ to ‘worst possible sleep’^48^	√	√	√	√
Brain/CNS impairment	Glasgow Coma Scale^19^	√			
Psychosocial features					
Anxiety and depression	HADS^49^	√	√	√	√
Coping strategies applied during a painful experience	CSQ-24^50^	√	√	√	√
Fear of movement or fear of injury or re-injury during movement	TSK-11^51^	√	√	√	√
Confidence in ability to perform activities despite pain	Pain Self-Efficacy Questionnaire^52^	√	√	√	√
Subjective post-traumatic distress	IES-R^53^	√	√	√	√
Injury characteristics					
Time of injury/incident	Hospital record data	√			
Injury location	Adapted pain drawings, based on hospital record data	√			
Tissues damaged	Based on imaging data and hospital records Fractures Penetrating/non-penetrating injury/both	√			
Surgery required	Location and postinjury timing of surgery, based on hospital record data	√			
Assisted mechanical ventilation required	Yes/no/duration	√			
Severity of injury	Injury Severity Scale^54^	√			
Pain characteristics					
Chronic pain severity	Chronic Pain Grade Scale^21^			√	√
Pain intensity	11-point (0–10) Numerical Rating Scale, relating to current pain, from ‘no pain’ to ‘pain as bad as could be’ (collected as part of the Chronic Pain Grade Scale)	√*	√	√	√
Pain medication intake (type, dosage and timing)	Medication Quantification Scale,^55–57^ based on hospital record data	√*			
Pain location	Pain drawing	√*	√	√	√
Pain extent	Electronic pain drawing^58^	√*			
Self-reported features of neuropathic pain	painDETECT questionnaire^59^	√*	√	√	√
Quantitative sensory testing					
Heat pain threshold	Evaluation of pain threshold using a heat stimulus	√*			
Cold pain threshold	Evaluation of pain threshold using a cold stimulus	√*			
Pressure pain threshold	Evaluation of pain threshold using a pressure stimulus	√*			
Temporal summation	Evaluation of pain intensity in response to repetitive pressure stimuli	√*			
Biomarkers					
CRP	Serum levels of CRP, a broad indicator of inflammation (via blood analysis)	√†			
cfDNA	Plasma levels of cfDNA (nuclear and mitochondrial), indicators of tissue damage (via blood analysis)	√†			

*Measurements to be taken repeatedly throughout the baseline period, which will commence immediately following recruitment via informed consent (up to 14 days post-trauma) for a period of up to 14 days (i.e. until a maximum of 28 days post-trauma), while the participant is in hospital.

†Measurements to be taken repeatedly throughout the baseline period, but may be commenced prior to this, subject to assent from a legal consultee.

BMI, body mass index; cfDNA, cell-free DNA; CRP, C reactive protein; CSQ-24, Coping Strategies Questionnaire-2; HADS, Hospital Anxiety and Depression Scale; IES-R, Impact of Event Scale revise; SF-36v2, 36-item Short Form Health Survey, version 2; TSK-11, Tampa Scale of Kinesiophobia, 11-item.

### Outcome

The outcome for the prediction model is an individual’s absolute risk of poor outcome (chronic pain and disability) at 6 months postinjury. Outcome will be measured using the Chronic Pain Grade Scale (CPGS),^21^ which combines pain intensity and pain-related disability over the preceding 6 months into a single measure of pain *severity*. The CPGS has previously been used to assess the severity of body-wide chronic pain in general population,^22^ primary care^23^ and post-trauma^24^ populations. Each item of the CPGS relates to at least one of the three categories of the International Classification of Functioning, Disability and Health (ICF)^25^: impairment, activity limitations and restricted participation. Furthermore, all ICF categories are encompassed by the CPGS.^26^ The CPGS is a unidimensional scale, with good internal consistency across different pain populations; Cronbach’s alpha of 0.84 to 0.91 in back pain, 0.79 for headache and 0.84 for temporomandibular pain.^21 27^ With regards to construct validity, cross-sectional and longitudinal studies of general practice patients have shown high scores on the CPGS, indicating greater chronic pain, to be associated with higher rates of unemployment, greater pain impact scale scores, greater use of opioid analgesics and physician visits, depressed mood and lower self-rated health status.^21 27 28^ For convergent validity, the CPGS has been found to have good correlation with equivalent dimensions of the SF-36.^27 28^ In terms of responsiveness, changes in score over time in patients with chronic musculoskeletal pain correlated significantly with changes in SF-36 scores.^29^ The CPGS has also been shown to have good test–retest reliability in primary care patients with back pain (weighted kappa 0.81, 95% CI 0.65 to 0.98).^27^

Although pain persistence is not used in assigning pain grade, a measure of pain days in the prior 6 months is included in the CPGS.^30^ The responses on the remaining seven items are used for computing scores for the three subscales of the CPGS^21^: characteristic pain intensity, disability score and disability points. The characteristic pain intensity score (range: 0–100) is obtained by calculating the mean of three pain intensity measurements: ‘at the present time’, the ‘worst pain’ in the preceding 6 months and the ‘average’ pain over the preceding 6 months. The disability score (range: 0–100) is obtained through the mean ratings of how much the pain has interfered in performing activities of daily living, recreational, social and family activities, and work (including housework) activities in the last 6 months. The disability points are scored 0–3 and are derived from a combination of ranked categories of the number of disability days (the number of days that the respondent was away from usual activities in the preceding 6 months due to pain) and disability score. Based on these scores, the participant’s chronic pain and disability status can then be classified into one of the five ordinal categories of chronic pain severity^21^: no pain (Grade 0), low disability and low intensity pain (Grade I), low disability and high intensity pain (Grade II), high disability and moderately limiting intensity pain (Grade III), and high disability and severely limiting intensity pain (Grade IV). As in previous studies, poor outcome will be defined as grade ≥II.^23 31–34^

### Candidate predictors

Candidate predictors have been selected that are: (1) reliable and valid measures of their domain, and (2) have a theoretical association with the development of chronic pain. Both modifiable and non-modifiable candidate predictors are included. Candidate predictors are summarised in table 1, with further detail in the online supplementary file S1. Table 1 details important data that will be measured at 3, 6 and 12 months postinjury to explore the clinical course of recovery following injury in the shorter and longer terms. All data collection will be standardised through protocols and clinical report forms.

10.1136/bmjopen-2017-017876.supp1 Supplementary data

### Data handling

Blood samples will be collected through the clinical and research nurse teams, while the participant is in the hospital, and either analysed immediately (C-reactive protein) or securely stored for subsequent analysis (cell-free DNA). Baseline self-reported questionnaires, pain and injury drawings, and physical assessments will be collected by one of three trained assessors from the study team. Inter-rater reliability studies (across two assessors) will first be conducted in both healthy and trauma populations to inform definitive testing protocols. The order of physical assessment data collection will be randomly assigned (using computerised randomisation software) according to the modality of testing and by site, to prevent order effects. Follow-up self-reported questionnaires will be posted to participants at their home addresses; with up to two postal reminders and a telephone call for non-response. All questionnaires will be formatted so that data can be scanned or entered directly into an electronic database. Following data entry, data will be checked by a second researcher for completeness and accuracy. In addition, regular audits of data collection and storage will be performed by an independent study management committee. Participant identifiable information will be securely stored within the hospital, in line with current United Kingdom data protection legislation, and only accessible by the site Principal Investigator and Research Nurse team who will not be involved in data analysis. All outcome measure data will be securely transferred within an anonymised database file to physically secure servers at the University of Birmingham, and stored for a period of 10 years in line with Research Governance procedures. Participants will receive usual care and interventions received will be recorded for descriptive analysis. Anonymised data will be analysed using IBM SPSS Statistics.

### Sample size

In predictive modelling, a larger sample size enables lower bias and variance, and permits the prospective prediction of new observations.^15^ The number of predictors will be reduced using exploratory factor analysis. This process will ensure that the sample size provides at least 10 cases per candidate predictor, to adequately power the final regression analysis.^35 36^ Data will be collected for an estimated 300 participants per cohort (n=600 total) to allow for withdrawals (primarily expected deaths within the first 3 months) and losses to follow-up, so that final data are available for 250 participants per cohort (n=500 total).

### Statistical analysis methods and management of missing data

For each cohort, potentially eligible patients, numbers examined for eligibility, confirmed eligible, recruited into the study, completing follow-up and analysed will be reported in a flow diagram. Reasons for non-participation, exclusion, drop-outs and withdrawal (eg, death) will be reported at each stage. Participant characteristics will be descriptively presented. For each variable of interest, the number of participants with missing data will be reported.

For the first cohort to develop the predictive model, an initial exploratory data analysis stage will summarise the data.^15^ Correlations between candidate predictive factors will be calculated at baseline. Outcome (CPGS) scores will be dichotomised into good and poor categories as described previously. Data reduction will use exploratory factor analysis to assess factor loading of candidate predictors (summary scores) on poor outcome at 6 months. This will enable the number of candidate predictors entered into the final model to be reduced to 25, which can be supported by the cohort sample of 250. This process reduces the risk of over-fitting the model and the risk of selecting the wrong variables due to correlation between predictor variables (multicollinearity).^37^

Statistical modelling for prediction has been planned *a priori*. To explore the influence of each predictive factor on poor outcome at 6 months, a logistic multivariable regression model will be fitted to the dichotomised outcome scores to calculate low and high risk of poor outcome. Odds ratios for each candidate predictive factor will be reported, adjusted for other factors and account for clustering (eg, level of injury severity). If necessary, multiple imputation^38^ will be used to deal with missing outcome data. The characteristics of those patients with and without 6-month data will also be compared, to inform whether patients with no 6-month data were missing at random. Reduced multivariable analyses will be considered if necessary (eg, removing one of two candidate predictive factors that are highly correlated at baseline), to examine the robustness of the conclusions.

### Risk groups and development of the predictive screening tool

The predictive model will be used to develop a risk stratification tool to inform an individual’s absolute risk of poor outcome. The stratification tool will inform clinical decision-making for precision rehabilitation. Items will be selected for the model if they are statistically significantly (P<0.05) associated with poor outcome in the logistic regression analysis, and those deemed clinically important to retain using expert opinion (regardless of statistical significance, study steering group) to improve face validity for clinicians and avoid overfitting of the model.^37^ The regression model with included predictive factors will be fitted to the data from the first of the two cohorts to obtain a final set of parameter estimates (i.e. alpha and beta terms), which will be used to form the model. An important requirement of the stratification tool is that it should be sufficiently brief to facilitate use in clinical practice. Thus, we will look to simplify the model where possible to facilitate its use, but without important reduction in its predictive ability in terms of calibration and discrimination. For example, if multi-item questionnaire scores are included in the model, then we will evaluate whether just one of the questionnaire items is sufficient. Ideally, this process will result in a full and simplified model.

### Development versus validation

For validation of the model, data from the second of the two cohorts will be compared with that of the first to enable analysis of the distribution of important variables, inclusive of demographic, predictor and outcome variables. The predictive performance of the screening tool (discrimination, calibration and goodness of fit) will be assessed using data from the second cohort. Data in both cohorts will be consistent in terms of setting, eligibility criteria, outcome and predictors.

## Discussion

There is an urgent need for a robust predictive study to predict the transition from acute to chronic pain in a musculoskeletal trauma population. Using such a comprehensive array of outcome measures will allow the development and validation of a predictive tool to predict development of chronic and disabling pain, and begin the process of identifying appropriate and precision interventions.

The candidate predictors used in this study have been chosen to be as comprehensive as possible, based on current knowledge of pain science. Other candidate predictors were considered (eg, microRNA biomarkers), but their mechanistic functions and temporal progression are not yet sufficiently clear to justify the expense of their inclusion. The combination of patient-reported outcome measures, psychophysical testing and biomarkers that are included are designed to act as surrogates for the four primary mechanisms of pain^8 39 40^: *nociceptive* (injury location, severity and characteristics), *neuropathic* (painDETECT tool and pain extent, *inflammatory* (biomarkers) and *nociplastic* (quantitative sensory testing, painDETECT and pain location and extent). In addition, other patient-reported outcome measures (eg, pain intensity, post-traumatic stress, anxiety and depression, coping and pain self-efficacy) are included as the domains that they measure have been shown to influence prognosis for long-term outcomes in populations with pain in a range of locations.^9 23 24^

Rehabilitation is widely regarded as an important component of post-trauma healthcare^41^; however, the current position of equipoise means that precision rehabilitation has not yet been identified. Understanding mechanisms that underlie the transition from acute to chronic pain is essential to moving beyond this position. Identifying predictive factors related to poor outcome of pain and disability outcome will facilitate targeting of effective interventions. This will inform rehabilitation decision-making and facilitate improvements in clinical and cost-effectiveness of rehabilitation interventions.

Limited research has identified criteria for quality in a predictive model, but authors have identified potential quality issues to ensure methodological rigour.^42^ These issues are summarised in table 2 and incorporated into the study design to ensure low risk of bias in development and validation of the predictive model.

**Table 2:** Methodological decisions to improve study quality

Criteria^42^	Methodological decisions to improve quality
Study design	
Inception cohort	Clear description of population Clear description of the participants at baseline
Source population	Clear description of population Clear description of sampling frame and recruitment (method and timing)
Inclusion and exclusion criteria	Clarity of eligibility criteria
Prospective design	Clarity of study design
Study attrition	
Number of drop-outs	Adequate participation rate Clear description of attempts to collect information on participants who dropped out Reporting numbers and reasons for loss to follow-up
Information provided on method of management of missing data	Appropriate methods of imputation of missing data
Predictive factors	
All predictive factors described used to develop the model	Clear definition of predictive factors An adequate proportion of participants has complete data for the predictive factor
Standardised or valid measurements	The measurement of the predictive factor is reliable and valid The measurement of the predictive factor is the same for all participants
Linearity assumption studied	Linearity of data will be reported
No dichotomisation of predictive variables	Continuous variables will be reported where possible
Data presentation all predictive factors	Complete data will be presented
Outcome measures	
Description of outcome measures	The outcome is clearly defined
Standardised or valid measurements	The measurement of the outcome is reliable and valid The measurement of the outcome is the same for all participants
Data presentation of most important outcome measures	Complete data will be presented
Analysis	
Presentation of univariate crude estimates	An appropriate strategy for model building is described An adequate statistical model described
Sufficient numbers of subjects per variable	Adequate data will be presented
Selection method of variables explained	Sufficient data will be presented to enable assessment of the adequacy of the analytic strategy All results will be reported
Presentation of multivariate estimates	An appropriate strategy for model building is described An adequate statistical model described
Clinical performance/validity	
Clinical performance	Clinical performance of the model will be reported
Internal validation	Internal validation will be reported
External validation	Not a focus of this study

### Patient burden and potential distress

The primary ethical concern is to limit distress on participants. As such, to reduce the patient burden when collecting baseline data, the self-reported questionnaires will be administered by members of the study team shortly following obtaining fully informed consent, and physical assessment outcomes will be measured at least 24 hours later. Patients will be asked for consent to not only provide new data for the study, but also for the study team to access information that will have been routinely collected by the hospital staff since the time of admission (eg, nature and circumstances of injury, medical history, medication details and blood test results). This will be fully explained to patients and explicitly detailed in the participant information sheet.

### Mental capacity

Because of the nature of their injuries, the patient’s mental capacity will be assessed on admission into hospital and thereafter by clinical staff and/or research nurses. The mental capacity of eligible patients at the time of being approached for recruitment will therefore fall into one of two groups: either they possess or are lacking mental capacity (in accordance with the Mental Capacity Act 2005) to provide informed consent to voluntarily participate in the study.

For patients possessing mental capacity to provide consent, a research nurse or member of the research team will ask if they are interested in participating in the study. If they are interested, a copy of the participant information sheet will be provided (and if necessary read to them) to give them an outline of the study. Following an opportunity to seek additional information and ask questions, the patient will be asked if they wish to provide their written informed consent to participate in the study, at which point a consent form will need to be signed.

On admission to the hospital, an otherwise eligible patient may lack the mental capacity to decide whether to provide consent to participate in a research study (eg, due to the severity of their injuries, because they are arriving intubated and ventilated, or as a side-effect of medication for their injuries). They may or may not regain this capacity during their stay in the hospital. Due to our wish to begin measuring biomarkers as early as possible following the onset of trauma, for some otherwise eligible patients it would be necessary to take blood samples before the patient has regained the capacity to provide informed consent. Using the convention of previous studies in trauma populations,^43^ recruitment into the study will be undertaken under the provision and guidance of the Mental Capacity Act 2005 for research in emergency situations, and in accordance with the Declaration of Helsinki. As such, if a patient does not possess this capacity when first approached for recruitment, the research team will request a mandate to collect blood samples from a legal consultee. This mandate can continue until the patient gains sufficient capacity to make an informed decision as to whether they wish to provide consent or not. We will use this mandate up to 14 days from the date of the trauma. If the patient does not regain capacity within 14 days following the trauma, or if informed consent is not provided by the patient once capacity to do so is regained, any samples collected will be destroyed before any non-clinical biomarker analysis (i.e. cell-free DNA) is performed. Furthermore, only once informed consent has been gained from the patient would the research team proceed to collect any self-reported questionnaire or physical assessment data. The legal consultee can either be a ‘personal consultee’, for example, family member, or a ‘nominated consultee’, for example, intensive care consultant. Once a consultee (personal or nominated) has been identified, they will be provided with the participant information sheet, to inform them about the study. The consultee will be asked if they feel participating in the study would be something to which the patient would agree or object to. If, in their opinion, the patient would agree to participating in the study, the consultee will be asked to sign a declaration form, and the research team can begin the schedule of blood sample collections. If, at any time prior to the patient regaining capacity, the consultee decides to withdraw assent, then no further samples will be collected until the patient can be approached for formal recruitment (if appropriate).

### Other ethical issues

Participants will be informed that they are free to withdraw from the study at any time, without needing to provide reason. At each data collection visit, the capacity of the participant will be checked (using an Abbreviated Mental Test) and asked if they are happy to proceed with data collection. Any concerns for a participant by the study team will be fed back to clinical staff. All blood samples will be collected by hospital staff and the research nurse team and will be stored, tested and disposed of in accordance with current United Kingdom guidelines and regulations. In the event of death within 3 months of being recruited, participants will be automatically withdrawn from the study and the primary predictive analysis. Baseline characteristics of withdrawn participants will be compared with those of retained participants to assess for any differences.

## Supplementary Material

Reviewer comments

Author's manuscript

## References

[1] Berben SA, Schoonhoven L, Meijs TH, et al Prevalence and relief of pain in trauma patients in emergency medical services. Clin J Pain 2011;27:587–92. 10.1097/AJP.0b013e3182169036 21505324

[2] Williamson OD, Epi GD, Gabbe BJ, et al Predictors of moderate or severe pain 6 months after orthopaedic injury: a prospective cohort study. J Orthop Trauma 2009;23:139–44. 10.1097/BOT.0b013e3181962e29 19169107

[3] Mannion RJ, Woolf CJ Pain mechanisms and management: a central perspective. Clin J Pain 2000;16(3 Suppl):S144–56. 10.1097/00002508-200009001-00006 11014459

[4] Holbrook TL, Anderson JP, Sieber WJ, et al Outcome after major trauma: discharge and 6-month follow-up results from the Trauma Recovery Project. J Trauma 1998;45:315–23. Discussion 23-4. 9715189 10.1097/00005373-199808000-00018

[5] Holbrook TL, Anderson JP, Sieber WJ, et al Outcome after major trauma: 12-month and 18-month follow-up results from the Trauma Recovery Project. J Trauma 1999;46:765–71. Discussion 71-3. 10338392 10.1097/00005373-199905000-00003

[6] Harris IA, Young JM, Rae H, et al Predictors of general health after major trauma. J Trauma 2008;64:969–74. 10.1097/01.ta.0000245972.83948.1a 18404063

[7] Ponsford J, Hill B, Karamitsios M, et al Factors influencing outcome after orthopedic trauma. J Trauma 2008;64:1001–9. 10.1097/TA.0b013e31809fec16 18404068

[8] Vardeh D, Mannion RJ, Woolf CJ Toward a mechanism-based approach to pain diagnosis. J Pain 2016;17(9 Suppl):T50–69. 10.1016/j.jpain.2016.03.001 27586831 PMC5012312

[9] Artus M, Campbell P, Mallen CD, et al Generic prognostic factors for musculoskeletal pain in primary care: a systematic review. BMJ Open 2017;7:e012901 10.1136/bmjopen-2016-012901 PMC5253570 28096253

[10] Latremoliere A, Woolf CJ Central sensitization: a generator of pain hypersensitivity by central neural plasticity. J Pain 2009;10:895–926. 10.1016/j.jpain.2009.06.012 19712899 PMC2750819

[11] Linley JE, Rose K, Ooi L, et al Understanding inflammatory pain: ion channels contributing to acute and chronic nociception. Pflugers Arch 2010;459:657–69. 10.1007/s00424-010-0784-6 20162302

[12] Woolf CJ Central sensitization: implications for the diagnosis and treatment of pain. Pain 2011;152:S2–S15. 10.1016/j.pain.2010.09.030 20961685 PMC3268359

[13] Altman DG, Vergouwe Y, Royston P, et al Prognosis and prognostic research: validating a prognostic model. BMJ 2009;338:b605 10.1136/bmj.b605 19477892

[14] Moons KG, Kengne AP, Grobbee DE, et al Risk prediction models: II. External validation, model updating, and impact assessment. Heart 2012;98:691–8. 10.1136/heartjnl-2011-301247 22397946

[15] Shmueli G To explain or to predict? Statistical Science 2010;25:289–310. 10.1214/10-STS330

[16] Collins GS, Reitsma JB, Altman DG, et al Transparent reporting of a multivariable prediction model for individual prognosis or diagnosis (TRIPOD): the TRIPOD statement. BMC Med 2015;13:1 10.1186/s12916-014-0241-z 25563062 PMC4284921

[17] Merskey H, Bogduk N Classification of chronic pain: descriptions of chronic pain syndromes and definitions of pain terms. USA: IASP Press, 1994.

[18] Hodkinson HM Evaluation of a mental test score for assessment of mental impairment in the elderly. Age Ageing 1972;1:233–8. 10.1093/ageing/1.4.233 4669880

[19] Teasdale G, Jennett B Assessment of coma and impaired consciousness. A practical scale. Lancet 1974;2:81–4. 4136544 10.1016/s0140-6736(74)91639-0

[20] Reith FC, Van den Brande R, Synnot A, et al The reliability of the Glasgow Coma Scale: a systematic review. Intensive Care Med 2016;42:3–15. 10.1007/s00134-015-4124-3 26564211

[21] Von Korff M, Ormel J, Keefe FJ, et al Grading the severity of chronic pain. Pain 1992;50:133–49. 10.1016/0304-3959(92)90154-4 1408309

[22] Parsons S, Breen A, Foster NE, et al Prevalence and comparative troublesomeness by age of musculoskeletal pain in different body locations. Fam Pract 2007;24:308–16. 10.1093/fampra/cmm027 17602173

[23] Muller S, Thomas E, Dunn KM, et al A prognostic approach to defining chronic pain across a range of musculoskeletal pain sites. Clin J Pain 2013;29:411–6. 10.1097/AJP.0b013e318257099e 23549065 PMC3616264

[24] Rivara FP, Mackenzie EJ, Jurkovich GJ, et al Prevalence of pain in patients 1 year after major trauma. Arch Surg 2008;143:282–7. discussion 88. doi 10.1001/archsurg.2007.61 18347276

[25] Organization WH. The International Classification of Functioning, Disability and Health (ICF. Geneva: WHO, 2001.

[26] Dixon D, Pollard B, Johnston M What does the chronic pain grade questionnaire measure? Pain 2007;130:249–53. 10.1016/j.pain.2006.12.004 17257751

[27] Smith BH, Penny KI, Purves AM, et al The Chronic Pain Grade questionnaire: validation and reliability in postal research. Pain 1997;71:141–7. 10.1016/S0304-3959(97)03347-2 9211475

[28] Penny KI, Purves AM, Smith BH, et al Relationship between the chronic pain grade and measures of physical, social and psychological well-being. Pain 1999;79:275–9. 10.1016/S0304-3959(98)00166-3 10068173

[29] Elliott AM, Smith BH, Smith WC, et al Changes in chronic pain severity over time: the Chronic Pain Grade as a valid measure. Pain 2000;88:303–8. 10.1016/S0304-3959(00)00337-7 11068118

[30] Von Korff M, Jensen MP, Karoly P Assessing global pain severity by self-report in clinical and health services research. Spine 2000;25:3140–51. 10.1097/00007632-200012150-00009 11124730

[31] Von Korff M, Miglioretti DL A prognostic approach to defining chronic pain. Pain 2005;117:304–13. 10.1016/j.pain.2005.06.017 16153772

[32] Von Korff M, Dunn KM Chronic pain reconsidered. Pain 2008;138:267–76. 10.1016/j.pain.2007.12.010 18226858 PMC2613775

[33] Dunn KM, Croft PR, Main CJ, et al A prognostic approach to defining chronic pain: replication in a UK primary care low back pain population. Pain 2008;135(1-2):48–54. 10.1016/j.pain.2007.05.001 17570585

[34] Thomas E, Dunn KM, Mallen C, et al A prognostic approach to defining chronic pain: application to knee pain in older adults. Pain 2008;139:389–97. 10.1016/j.pain.2008.05.010 18583051

[35] Peduzzi P, Concato J, Kemper E, et al A simulation study of the number of events per variable in logistic regression analysis. J Clin Epidemiol 1996;49:1373–9. 10.1016/S0895-4356(96)00236-3 8970487

[36] Vittinghoff E, McCulloch CE Relaxing the rule of ten events per variable in logistic and Cox regression. Am J Epidemiol 2007;165:710–8. 10.1093/aje/kwk052 17182981

[37] Sainani KL Multivariate regression: the pitfalls of automated variable selection. Pm R 2013;5:791–4. 10.1016/j.pmrj.2013.07.007 24054854

[38] Sterne JA, White IR, Carlin JB, et al Multiple imputation for missing data in epidemiological and clinical research: potential and pitfalls. BMJ 2009;338:b2393 10.1136/bmj.b2393 19564179 PMC2714692

[39] Woolf CJ, Bennett GJ, Doherty M, et al Towards a mechanism-based classification of pain? Pain 1998;77:227–9. 10.1016/S0304-3959(98)00099-2 9808347

[40] Kosek E, Cohen M, Baron R, et al Do we need a third mechanistic descriptor for chronic pain states? Pain 2016;157:1382–6. 10.1097/j.pain.0000000000000507 26835783

[41] NICE. Rehabilitation after critical illness in adults Clinical guideline [CG83], 2009.

[42] van Oort L, van den Berg T, Koes BW, et al Preliminary state of development of prediction models for primary care physical therapy: a systematic review. J Clin Epidemiol 2012;65:1257–66. 10.1016/j.jclinepi.2012.05.007 22959592

[43] Hazeldine J, Naumann DN, Toman E, et al Prehospital immune responses and development of multiple organ dysfunction syndrome following traumatic injury: A prospective cohort study. PLoS Med 2017;14:e1002338 10.1371/journal.pmed.1002338 28719602 PMC5515405

[44] Eriksen HR, Ihlebaek C, Ursin H A scoring system for subjective health complaints (SHC). Scand J Public Health 1999;27:63–72. 10.1177/14034948990270010401 10847674

[45] Ware JE SF-36 health survey update. Spine 2000;25:3130–9. 10.1097/00007632-200012150-00008 11124729

[46] Brooks R EuroQol: the current state of play. Health Policy 1996;37:53–72. 10.1016/0168-8510(96)00822-6 10158943

[47] Mahoney FI, Barthel DW Functional evaluation: the barthel index. Md State Med J 1965;14:61–5. 14258950

[48] Cappelleri JC, Bushmakin AG, McDermott AM, et al Psychometric properties of a single-item scale to assess sleep quality among individuals with fibromyalgia. Health Qual Life Outcomes 2009;7:54 10.1186/1477-7525-7-54 19534799 PMC2706811

[49] Zigmond AS, Snaith RP The hospital anxiety and depression scale. Acta Psychiatr Scand 1983;67:361–70. 10.1111/j.1600-0447.1983.tb09716.x 6880820

[50] Harland NJ Georgieff K. Development of the coping strategies questionnaire 24, a clinically utilitarian version of the coping strategies questionnaire. Rehabilitation Psychology 2003;48:296–300.

[51] Woby SR, Roach NK, Urmston M, et al Psychometric properties of the TSK-11: a shortened version of the Tampa Scale for Kinesiophobia. Pain 2005;117(1-2):137–44. 10.1016/j.pain.2005.05.029 16055269

[52] Nicholas MK The pain self-efficacy questionnaire: taking pain into account. Eur J Pain 2007;11:153–63. 10.1016/j.ejpain.2005.12.008 16446108

[53] Beck JG, Grant DM, Read JP, et al The impact of event scale-revised: psychometric properties in a sample of motor vehicle accident survivors. J Anxiety Disord 2008;22:187–98. 10.1016/j.janxdis.2007.02.007 17369016 PMC2259224

[54] Baker SP, O’Neill B, Haddon W, et al The injury severity score: a method for describing patients with multiple injuries and evaluating emergency care. J Trauma 1974;14:187–96. 4814394

[55] Masters Steedman S, Middaugh SJ, Kee WG, et al Chronic-pain medications: equivalence levels and method of quantifying usage. Clin J Pain 1992;8:204–14. 1421733

[56] Harden RN, Weinland SR, Remble TA, et al Medication Quantification Scale Version III: update in medication classes and revised detriment weights by survey of American Pain Society Physicians. J Pain 2005;6:364–71. 10.1016/j.jpain.2005.01.350 15943958

[57] Gallizzi M, Gagnon C, Harden RN, et al Medication Quantification Scale Version III: internal validation of detriment weights using a chronic pain population. Pain Pract 2008;8:1–4. 10.1111/j.1533-2500.2007.00163.x 18211588

[58] Barbero M, Moresi F, Leoni D, et al Test-retest reliability of pain extent and pain location using a novel method for pain drawing analysis. Eur J Pain 2015;19:1129–38. 10.1002/ejp.636 25565607

[59] Freynhagen R, Baron R, Gockel U, et al painDETECT: a new screening questionnaire to identify neuropathic components in patients with back pain. Curr Med Res Opin 2006;22:1911–20. 10.1185/030079906X132488 17022849

