# Supplementary material for: Development of a screening tool to predict the risk of chronic pain and disability following musculoskeletal trauma: protocol for a prospective observational study in the United Kingdom
Source: BMJ Open. 2018 Apr 28;8(4):e017876. doi: 10.1136/bmjopen-2017-017876 (PMC5931282; doi:10.1136/bmjopen-2017-017876)
Supplement: Supplementary data [file bmjopen-2017-017876supp001.pdf]

Supplementary file 1. Candidate predictors

General participant characteristics

Several participant demographic features will be recorded at baseline, based on available hospital records and patient self-reported recollection, including smoking status, age, gender, height and weight to calculate body mass index [BMI], education [highest attained educational level], employment status [at the time of trauma], circumstance of trauma [military or civilian], previous history of musculoskeletal pain and injury, comorbidity of other current health problems.

Quality of life and physical functioning

<u>36-Item Short Form Health Survey, Version 2.0 [SF-36v2]</u>

The SF-36v2 is a self-reported measure of health-related quality of life, modified from the original SF-36, which was developed as part of the Medical Outcomes Study.[1] The 36-item questionnaire has subscales that assess physical function, social and psychological wellbeing.[2 3] The scores can be divided into physical and mental component summary scales.[4] The SF-36 has been shown to be valid and has been tested extensively in a trauma population.[5] Ware[6] reports multiple studies showing internal consistency above 0.70, with physical and mental scores exceeding 0.90. Minimal clinically important difference has been reported as 5.5 in a musculoskeletal trauma population.[7] Introduced in 1996, version 2.0 of the SF-36 is comparable to the original, retaining all subscales, with improvements to layout, presentation, response scales, wording and scoring.[6] The 'acute' [1 week recall period] version will be used, since the 4 week recall would not be appropriate for post-injury recall at baseline.

EuroQol Five Dimension Scale, 5-level [EQ-5D-5L]

Health-related quality of life will be quantified using the EQ-5D-5L through which 243 possible health states are converted to a single index value of range 0 to 1 where 1 is perfect health, and a visual analogue scale range 0–100, representing 'worst' to 'best' imaginable health state, respectively.[8] The EQ-5D-5L, with each item having 5 possible responses, has improved inter-observer [ICC 2,1 0.57] and test-retest [ICC 2,1 0.69] reliability compared to the previous EQ-5D-3L.[9] In addition, it has less ceiling effects [20.8% reduction] and adequate convergent validity when compared with the WHO-5 [spearman rank 0.38-0.51].[10]

Barthel Index of Activities of Daily Living

The Barthel Index of Activities of Daily Living is routinely collected by clinical staff at the hospital, and will be used to evaluate self-care and mobility during activities of daily living.[11 12] It is a 10-item ordinal scale encompassing a range of mobility physical activity tasks. Each item is related to a specific task and rated with a given number of points. A score of '0' is given for least independence/function on that item and scores above that [1 or 2] are given for increasing independence/function [range: 0-20]. The amount of time and physical assistance required to perform each task are used in determining the assigned value of each item. A higher score is associated with a greater likelihood of being able to live at home with a degree of independence following discharge from hospital. With most measurement testing performed in the stroke population, the Barthel Index has demonstrated excellent internal consistency [0.89-0.90][13] and is highly responsive in detecting changes[14] with a minimal detectable change of 4.02 and minimally clinically important difference of 1.85.[15] High correlations have been demonstrated with the Functional Independence Measure [FIM], indicating convergent validity of the instrument.[16]

Subjective Health Complaints Inventory

Premorbid subjective health complaints will be assessed for the 6 months preceding the traumatic injury, using the single-item questions for non-somatic domains from the Subjective Health Complaints Inventory.[17] The Subjective Health Complaints Inventory has been shown to be a reliable measure of recording subjective health complaints for a 30 day recall period,[17] although psychometric properties have not been reported for an extended 6 month recall period.

Sleep quality

Current sleep quality [over the previous 24 hour period] will be assessed using an 11-point Numerical Rating Scale [NRS], ranging from 0 ['best possible sleep'] to 10 ['worst possible sleep']. This scale has been shown to possess moderate psychometric properties in fibromyalgia patients using a symptom diary.[18] We will aim to assess current pain intensity at baseline, as frequently as every 48-hours while the patient is in hospital up to a maximum of 14 days following recruitment [depending upon patient accessibility and assessor availability], to gain accurate average and rate-of-change data. In addition, we will use the 0-10 NRS to assess average sleep quality, related to the preceding 6-months at the 6 and 12-month assessment points, although no psychometric properties have previously been reported for this recall period.

Psychosocial features

The predictive strength of psychosocial factors demonstrated in both primary care,[19 20] and post-trauma pain literature[21-24] demonstrates the importance of including these domains as candidate predictors.

The Hospital Anxiety and Depression Scales [HADS]

The HADS will be used to measure depression and anxiety, and their role in the manifestation of somatic symptoms.[25] There are 7 items which produce a cumulative score [range 0–21] for the anxiety [HADS-A] and depression [HADS-D] subscales, with a higher score indicative of greater anxiety and depression.[26] HADS has been tested in multiple populations demonstrating adequate to excellent internal consistency of HADS-A [0.68-0.93] and HADS-D [0.67-0.90].[26] Standard measurement of error in a coronary heart disease population was identified as 1.37 and 1.44 for anxiety and depression scales respectively, and minimal detectable change as 3.80 and 3.99 respectively.[27] The HADS has also demonstrated excellent concurrent validity when compared to various other depression/anxiety scales.[26]

Coping Strategies Questionnaire 24 [CSQ-24]

The CSQ-24 will be used to provide an indication of coping strategies used by participants when they are in pain.[28] Developed from items from the earlier, much larger Coping Strategies Questionnaire,[29] the CSQ-24 is a 23-item scale, composed of 4 subscales: *catastrophizing* [6 items], *diversion* [6 items], *reinterpreting* [6 items], and *cognitive coping* [5 items]. Participants are asked to indicate if they have particular thoughts and feelings when they are experiencing pain. A score on each item is summed to yield an aggregate score for each subscale, with a higher score reflecting greater attribution of that particular coping strategy. The CSQ-24 has demonstrated good internal consistency in populations with low back pain patients [Chronbach's alpha for the 4 factors ranged from 0.75 to 0.85] and work-related pain [0.80 to 0.86].[30] Harland & Georgieff[28] suggested that, since individuals may have a positive score on more than one subscale, the highest scoring subscale should be deemed the dominant coping strategy.[28] However, a recent study in a low back pain cohort,[31]

in which individual items from multiple questionnaires were factorised, suggested that *diversion*, *reinterpreting* and *cognitive coping* clustered together as a single factor, representing coping cognitions. By contrast, *catastrophizing* clustered with pain-related distress items.

Tampa Scale of Kinesiophobia [TSK-11]

The TSK-11 will be used to assess fear of movement or fear of injury or re-injury during movement.[32] It is an 11-item questionnaire, eliminating psychometrically poor items from its original 17-item version,[33] thus creating a shorter questionnaire with comparable internal consistency and a 2-factor structure [activity avoidance and harm]. Each of the 11 items is measured using a 4-point scale using the end points 1 [‘totally agree’] and 4 [‘totally disagree’] [scoring range 11–44]. Higher scores indicate more fear-avoidance behaviour. The TSK-11 has demonstrated acceptable to good internal consistency in acute and chronic musculoskeletal pain populations.[32 34] Test-retest reliability has been reported as excellent with a high standardised response mean; with good construct validity in relation to changes in disability and pain.[32]

Pain Self-Efficacy Questionnaire [PSEQ]

The patient’s confidence in their ability to perform activities despite their pain will be evaluated using the PSEQ. Developed from the Self-Efficacy Scale,[35] the PSEQ consists of 10 physical and psychosocial activity items measuring from 0 [‘not at all confident’] to 6 [‘completely confident’] thus generating a total score from 0-60.[36] The PSEQ has demonstrated excellent internal consistency [0.92], internal reliability [0.93], and test-re-test correlations [r=0.73] and has demonstrated validity when compared to other self-efficacy

measurements.[36] It has been used in several large population studies, for example Campbell et al.[31]

Impact of Event Scale Revised [IES-R]

The IES-R will be used to measure the subjective stress experienced by the participant following their traumatic event. The IES-R is a 22-item tool [range: 0-88] that consists of 8 intrusion and 8 avoidance items that are derived from the original IES,[37] with an additional 7-items assessing hyperarousal.[38] Accordingly, items correspond directly to symptoms of post-traumatic stress disorder.[38] Respondents are asked to identify a specific stressful life event and then indicate how much they were distressed or bothered during the past seven days by each ‘difficulty’ listed. Each item is rated on a 5-point scale ranging from 0 [‘not at all’] to 4 [‘extremely’]. The IES-R yields a total score ranging 0 to 88 and subscale scores can also be calculated for the Intrusion, Avoidance, and Hyperarousal sub-scales. The IES-R has demonstrated good internal consistency for all subscales [intrusion 0.87-0.94, avoidance 0.84-0.97, hyperarousal 0.79-0.91].[39] High correlations have been found between the IES-R and the original scale, supporting the concurrent validity of both measures.[38]

Injury characteristics

Several measures relating to the characteristics of the sustained injury will be taken at baseline, since it is plausible that some of these should possess predictive value.[40] The time of the injury will be gained from hospital records. The location of the injury/injuries will be recorded using an adapted version of previously developed pain drawing software, via a tablet computer.[41] Information relating to the tissues damaged from the injury [e.g. fractures sustained, whether the injury was penetrating, non-penetrating or both, review of available imaging data] will be gathered from hospital records, where possible. Whether the participant

received surgery following their admission [where, for what and when], and whether the participant received assisted mechanical ventilation will also be recorded.

Injury Severity Scale [ISS]

The ISS will be retrospectively calculated for each participant, including those who withdraw. The ISS is a numerical score with a range 0-75, that is used to describe the overall severity of injury, and can be used for both multiple and single injuries. The score is calculated, based on the Abbreviated Injury Scale [AIS] scores.[42 43] Higher ISS scores have been associated with increased rates of mortality[42 44 45] and length/cost of hospital stay.[46] It is the recommended tool for summarising injury severity by the Trauma Audit and Research Network [TARN]. Both TARN and the National Institute for Clinical Excellence[47] recommend any participant with a score of >8 to be referred for rehabilitation.

Pain characteristics

Pain characteristics [e.g. pain intensity, multi-site pain] have long been reported to hold predictive value for long-term pain across a variety of conditions.[19-21 48 49] It is therefore sensible that we include these domains as candidate predictors for post-trauma pain.

Pain intensity

Pain intensity will be measured using an 11-point [0-10] Numerical Rating Scale [NRS], measuring current pain from ‘no pain’ to ‘pain as bad as could be’, from the Chronic Pain Grade Scale.[50] We will aim to assess *current* pain intensity at baseline, as frequently as every 48-hours while the participant is in hospital [depending upon participant accessibility and

assessor availability], to gain accurate mean and rate-of-change data. At the 6 and 12 month assessment points, c*urrent* pain intensity, as well as *average* and *worst* pain intensity related to the preceding 6 months, will be collected as part of the Chronic Pain Grade Scale. NRS scales are sensitive, reliable and valid instruments for pain intensity measurement,[51-54] and have been recommended for use in clinical populations in preference to visual analogue scales or verbal rating scales.[55] A 30% change on a pain NRS score is considered clinically meaningful.[56-60]

Pain medication

The patient's pain medication [type, dosage and time since trauma] intake will be noted and the Medication Quantification Score [MQS], which is a reliable and validated score for quantifying analgesics, will be calculated to obtain a comparable metric for all different analgesics.[61-63] It enables characterisation of analgesics when many different medications are involved and doses are irregular. It will be calculated for each non-opioid and opioid, based on weights assigned by medication class and dosage level [level 1 = sub-therapeutic dosage and/or on demand, level 2 = lower 50% of the therapeutic dose range, level 3 = upper 50% of the therapeutic dose range, level 4 = supra-therapeutic dose] using the 1998 detriment weights.[64] The detriment weights are summed by the dosage level to provide the final score. These scores will be summed to provide a quantitative index for analgesic usage suitable for statistical analysis.

Pain drawing

All participants will be requested to complete a pain drawing, indicating the spatial distribution of their pain, over two body charts; one reporting a frontal view of the body and one a dorsal view. We will also ask patients to mark their single 'most painful' site on one of

these body charts. Pain drawing data will be collected using a custom software application on a tablet computer, and will be analysed with Matlab software, as described previously.[41] The software automatically calculates the number of shaded pixels from the pain drawing, which is defined as *pain extent*. Summaries of, and relationships between, pre-defined painful body regions will also be evaluated. Conventional pain drawing data will also be collected on paper follow-up questionnaires to assess painful body regions.

The painDETECT questionnaire

It is assumed that all post-trauma patients will have significant nociception at baseline, but given the relatively high proportion of neuropathic pain following traumatic injury,[65] the contribution of other pain-related mechanistic pathways should also be assessed. The painDETECT questionnaire[66] will be used to facilitate the identification of neuropathic pain. It consists of 9 items [7 evaluating pain quality, 1 evaluating pain pattern, and 1 evaluating pain radiation], all of which contribute to an aggregate score [range: -1-38]. This aggregate score can be divided into three classifications that represent the likelihood of neuropathic pain: ‘unlikely’ [0-12], ‘ambiguous’ [13–18] and ‘likely’ [19–38].[66] Although developed as a screening questionnaire for neuropathic pain, painDETECT may also function as a measure of characteristics that indicate augmented central pain processing.[66] The painDETECT questionnaire has demonstrated good internal consistency [0.76][67] and excellent test-re-test reliability[68] within 1-hour of consultation [ICC{model not reported} 0.911] and 1-week post consultation [ICC{model not reported} 0.79].[69] Convergent validity has been demonstrated in comparison to pain severity,[67 70] health-related quality of life[71] and similar neuropathic pain screening tools.[66] As such, painDETECT outcomes will be measured at regular intervals while the participant is an inpatient in the hospital [subject to participant accessibility and

assessor availability] to assess for emerging neuropathic pain and sensitization. Measurements will also be taken at all follow-up assessment points.

Quantitative sensory testing [QST]

QST methods will be used to assess pain sensibility, throughout which measurements will be concealed from participants. Owing to the clinical heterogeneity of the post-trauma population, precise standardisation of test sites between participants will not be possible. Instead, we have developed a standardised protocol that will be used to evaluate pain thresholds for multiple stimulus modalities [mechanical pressure, heat and cold] at the same sites in each participant. Each site where multi-modality pain threshold testing is performed will be within the receptive field of the same nerve root using described regions,[72] so that segmental cross-modality excitability may be compared. All pain thresholds will be measured at the same ‘local’ and ‘remote’ sites for each participant. We define local sites as being uninjured but within [or, if not accessible, as close as possible to] the same receptive field as the most painful inured tissue [e.g. skin over gastrocnemius in a participant with an ankle fracture]. By contrast, we define remote sites as a distant, accessible, site from the receptive fields in which tissues are injured [e.g. skin over tibialis anterior in a participant without lower limb injury], and on the contralateral side of the body where injured tissue is unilateral. Where possible, remote sites will be a mirror-image of the local site [to allow for comparison of absolute values], but in a trauma population we are aware that this may not always be possible. For all threshold testing modalities, an ascending method of limits design[73] will be used, whereby stimulus intensity will begin at a low level and gradually increase until the participant first perceives pain. Participants will be instructed to push a button or tell the assessor when the sensation has changed from one of the stimulus alone [e.g. just pressure] to

a sensation of both the stimulus and pain [e.g. pressure and pain]. Following a brief demonstration of equipment to familiarise participants, two consecutive assessments will be performed for each modality at each site, and the means used for further analysis.[74] A minimum of 30-seconds inter-stimulus interval will be given between each threshold measurement within a single session. Measurements will be taken at baseline, while the participant is an inpatient in the hospital; we will aim to collect data as frequently as every 48 hours to gain accurate rate-of-change data, but this will depend upon participant accessibility and assessor availability. To ensure pain thresholds are consistently measured at the same sites every session, sites will be labelled using a sterile, skin marking pen [Schuco Ltd, UK]. Because sites cannot be standardised between participants, the rates-of-change of these values will be used as candidate predictive variables, to allow for comparisons between participants. The order of pain threshold testing will be randomly assigned by modality at each session to avoid order effects.

Thermal [heat and cold] pain thresholds will be measured using skin-contact stimulation, using the same thermode at the same sites, within specified local and remote dermatomes. Thermal pain threshold assessments will be performed by delivering thermal stimuli directly to the skin through a metal 30x30 mm Peltier thermode, using a TSA-II NeuroSensory Analyzer thermal stimulator and accompanying software [Medoc Ltd, Israel]. To evaluate heat pain threshold, temperature will be gradually increased, at a rate of 1°C/s from a ‘neutral’ baseline of 32°C, to a maximum temperature of 50.5°C to avoid thermal injury.[75] During each measurement, participants will be instructed to press a button when the stimulus becomes painful, and this will be documented as the threshold value. Once pain threshold is achieved [and recorded], the temperature will return to the baseline value at the same rate [1°C/s]. For cold pain threshold measurements, the temperature will be gradually reduced, at

a rate of 1°C/s from the baseline of 32°C, to a minimum temperature of 0°C,[75] before also returning to baseline at a rate of 1°C/s.

Pressure pain thresholds will be measured using a digital pressure algometer [Series 7 force gauge, Mark-10 Corporation, USA], providing real-time force measurement and an analogue output that can be linked to a computer. Skin and muscle tissue are simultaneously stimulated during pressure threshold testing; sites will therefore be chosen where a dermatome and myotome are likely to share a common nerve root innervation [e.g. skin over tibialis anterior]. The algometer has a hard rubber circular contact tip of 1.2cm^2 area, with no sharp edges so to avoid an uneven pressure stimulus.[76] In order to preserve hygiene and attend to infection control measures in trauma patients, the contact tip will be covered with a clean, thin disposable covering. The tip will be applied perpendicular to the skin at a constant rate of pressure increase of 50kPa/s [6.0N/s using the 1.2cm^2 tip], until the first onset of pain. For each measurement, pressure will be unloaded immediately once the participant indicates that their pain threshold has been reached.[74]

To measure excitability of nociceptive pathways in response to mechanical stimuli, a series of repetitive, pressure stimulus ‘pulses’ will be applied via the digital algometer, with the aim of provoking temporal summation responses.[77-79] A minimum of 2 minutes after all threshold tests have been completed, a series of 10 consecutive pressure pulses will be applied at the remote and local sites [the order of site being randomly assigned]. The peak pressure reached during each pulse will be the mean pressure pain threshold that was measured for that particular site, as described previously. For each pulse, pressure will be gradually increased to the peak value over a period of 5 seconds, maintained at that peak value for 1 second, and then immediately released. A 5 second inter-stimulus interval will be used between pulses,

during which the tip of the algometer will remain in contact with the skin.[78 79] Pain intensity from the pulses will be rated on a numerical rating scale [0 being 'no pain' to 10 being 'pain as bad as could be']. In the event that participants indicate that pain has become intolerable, the sequence will be stopped immediately, and the NRS score and number of impulses performed at that point will be noted.

Biomarkers

Serum levels of C-reactive protein [CRP] will be used as a biomarker for inflammation; one of the primary mechanistic pathways that can evoke pain.[80] CRP is an acute-phase response protein produced by hepatocytes and is usually found in concentrations of 0.3 to 1.7 mg/l[81]. Increased production is due to cytokine-dependent induction of synthesis and elevated levels may be detected within eight hours of a stimulus and can reach 500 mg/1.28. Besides trauma,[82] elevated levels of CRP may be seen in conditions such as autoimmune disease, infection and malignancy. It has also been associated with acute sciatica.[83] The level of CRP usually peaks within 48 hours of the stimulus. In contrast, when the stimulus for increased production completely ceases, the circulating CRP concentration falls rapidly, at almost the rate of plasma CRP clearance.[84] A fall in serial measurements usually indicates resolution of the underlying process, while persisting elevated levels indicates ongoing inflammation.[85] Where possible, measurements of serum CRP will be repeatedly taken on a 48 hour schedule while the participant is an inpatient; absolute and rates of change of CRP values will be used as candidate predictive factors.[86]

Plasma cell-free DNA [cfDNA] will be used as an indicator of tissue damage. This includes both nuclear DNA [nDNA] and mitochondrial DNA [mtDNA], which circulate after being

released from cells when they are damaged and are thought to be amongst the important initiators of systemic inflammatory responses following tissue injury known as Damage Associated Molecular Patterns [DAMPs]. [87] Clinical outcomes in trauma patients have been related to plasma mtDNA concentration.[88 89] Other work with severe trauma patients has shown that cfDNA values rise to their peak value in the second week post-trauma, and then gradually return to baseline values after approximately 2 months.[90] Where possible, measurements of circulating cfDNA will be repeatedly taken on a 48 hour schedule while the participant is an inpatient; absolute values and rates of change of cfDNA values will be used as candidate predictive factors.

References

1. Stewart AL, Greenfield S, Hays RD, et al. Functional status and well-being of patients with chronic conditions. Results from the Medical Outcomes Study. *JAMA* 1989;262(7):907-13.
2. Ware JE, Jr., Sherbourne CD. The MOS 36-item short-form health survey (SF-36). I. Conceptual framework and item selection. *Med Care* 1992;30(6):473-83.
3. Hays RD, Sherbourne CD, Mazel RM. The RAND 36-Item Health Survey 1.0. *Health Econ* 1993;2(3):217-27.
4. McHorney CA, Ware JE, Jr., Raczek AE. The MOS 36-Item Short-Form Health Survey (SF-36): II. Psychometric and clinical tests of validity in measuring physical and mental health constructs. *Med Care* 1993;31(3):247-63.
5. Revell MP, Pynsent, P.B., Abudu, A., Fairbank, J.C.T. . Trauma Scores and Trauma Outcome Measures *Trauma* 2003;5:61-70.
6. Ware J. SF-36 Health Survey Updated. *Spine* 2000;25(24):3130-39.
7. Dattani R, Slobogean, G.P., O'Brien, P.J., Broekhuyse, H.M., Blachut, P.A., Guy, P., Lefaivre, K.A. Psychometric Analysis of Measuring Functional Outcomes in Tibial Plateau Fractures using the Short Form (SF-36), Short Musculoskeletal Function Assessment (SMFA) and the Western Ontario McMaster Osteoarthritis (WOMAC) Questionnaires. *Injury Int J Care Injured* 2013;44:825-29.
8. Brooks R. EuroQol: the current state of play. *Health Policy* 1996;37(1):53-72.
9. Janssen MF, Birnie E, Haagsma JA, et al. Comparing the standard EQ-5D three-level system with a five-level version. *Value Health* 2008;11(2):275-84. doi: 10.1111/j.1524-4733.2007.00230.x
10. Janssen MF, Pickard AS, Golicki D, et al. Measurement properties of the EQ-5D-5L compared to the EQ-5D-3L across eight patient groups: a multi-country study. *Qual Life Res* 2013;22(7):1717-27. doi: 10.1007/s11136-012-0322-4
11. Mahoney FI, Barthel DW. Functional Evaluation: The Barthel Index. *Md State Med J* 1965;14:61-5.
12. Shah S, Vanclay F, Cooper B. Improving the sensitivity of the Barthel Index for stroke rehabilitation. *J Clin Epidemiol* 1989;42(8):703-9.
13. Hseuh IP, Lee, M.M., Hsieh, C.L. . Psychometric Characteristics of the Barthel Activities of Daily Living Index in Stroke Patients *J Formos Med Assoc* 2001;100(526-532)
14. Hseuh IP, Lin, J.H., Jeng, J.S., Hsieh, C.L. Comparison of the Psychometric Characteristics of the Functional Independence Measure, 5 Item Barthel Index and 10 Item Barthel Index in Patients with Stroke. *J Neurol Neurosurg Psychiatry* 2002;73:188-90.
15. Hsieh YW, Wang, C.H., Wu, S.C., Chen, P.C., Sheu, C.F., Hsieh, C.L. . Establishing the Minimally Clinically Important Difference of the Barthel Index in Stroke Patients. *Neurorehabilitation and Neural Repair* 2007;21(3):233-38.
16. Hobart JC, Thompson AJ. The five item Barthel index. *J Neurol Neurosurg Psychiatry* 2001;71(2):225-30.
17. Eriksen HR, Ihlebaek C, Ursin H. A scoring system for subjective health complaints (SHC). *Scand J Public Health* 1999;27(1):63-72.
18. Cappelleri JC BA, McDermott AM, Sadosky AB, Petrie CD, Martin S. Psychometric properties of a single-item scale to assess sleep quality among individuals with fibromyalgia. *Health Qual Life Outcomes* 2009;17(7):54.
19. Mallen CD, Peat G, Thomas E, et al. Prognostic factors for musculoskeletal pain in primary care: a systematic review. *Br J Gen Pract* 2007;57(541):655-61.
20. Artus M, Campbell P, Mallen CD, et al. Generic prognostic factors for musculoskeletal pain in primary care: a systematic review. *BMJ Open* 2017;7(1):e012901. doi: 10.1136/bmjopen-2016-012901

21. Clay FJ, Newstead SV, Watson WL, et al. Bio-psychosocial determinants of persistent pain 6 months after non-life-threatening acute orthopaedic trauma. *J Pain* 2010;11(5):420-30. doi: 10.1016/j.jpain.2009.12.002
22. Archer KR, Castillo RC, Wegener ST, et al. Pain and satisfaction in hospitalized trauma patients: the importance of self-efficacy and psychological distress. *J Trauma Acute Care Surg* 2012;72(4):1068-77. doi: 10.1097/TA.0b013e3182452df5
23. Rosenbloom BN, Khan S, McCartney C, et al. Systematic review of persistent pain and psychological outcomes following traumatic musculoskeletal injury. *J Pain Res* 2013;6:39-51. doi: 10.2147/JPR.S38878
24. Vranceanu AM, Bachoura A, Weening A, et al. Psychological factors predict disability and pain intensity after skeletal trauma. *J Bone Joint Surg Am* 2014;96(3):e20. doi: 10.2106/JBJS.L.00479
25. Zigmond AS, Snaith RP. The hospital anxiety and depression scale. *Acta Psychiatr Scand* 1983;67(6):361-70.
26. Bjelland I, Dahl AA, Haug TT, et al. The validity of the Hospital Anxiety and Depression Scale. An updated literature review. *J Psychosom Res* 2002;52(2):69-77.
27. Wang W, Chair, S.Y., Thompson, D.R., Twinn, S.F. A Psychometric Evaluation of the Chinese Version of the Hospital Anxiety and Depression Scale in Patients with Coronary Heart Disease *Journal of Clinical Nursing* 2009;18:1908-15.
28. Harland NJ Georgieff K. Development of the coping strategies questionnaire 24, a clinically utilitarian version of the coping strategies questionnaire. *Rehabilitation Psychology* 2003;48(4):296–300.
29. Rosenstiel AK KF. The use of coping strategies in chronic low back pain patients: Relationship to patient characteristics and current adjustment. . *Pain* 1983;17: 33–44.
30. Chiu C JJ, Fujikawa M, Strand D, Cheing G, Lee G, Chan F Measurement Structure of the Coping Strategies Questionnaire-24 in a Sample of Individuals with Musculoskeletal Pain: A Confirmatory Factor Analysis. . *Rehabilitation Research, Policy, and Education* 2014;28(2):80-90.
31. Campbell P, Foster NF, Thomas E, Dunn KM. Prognostic Indicators of Low Back Pain in Primary Care: Five-Year Prospective Study. *J Pain* 2013;14(8):873–83.
32. Woby SR, Roach NK, Urmston M, et al. Psychometric properties of the TSK-11: a shortened version of the Tampa Scale for Kinesiophobia. *Pain* 2005;117(1-2):137-44. doi: 10.1016/j.pain.2005.05.029
33. Miller RP KS, Todd D. The Tampa Scale: a measure of kinesiophobia. *Clin J Pain* 1991;7(1):51-52.
34. Swinkels-Meewisse EJCM, Swinkels RAHM, Verbeek ALM, Vlaeyen JWS. Psychometric Propertieis of the Tampa Scale for Kinesiophobia and the Fear Avoidance Beliecs Questionnaire in Acute Low Back Pain *Manual Therapy* 2003;8(1):29-36.
35. Kall LB. Psychological determinants of quality of life in patients with whiplash associated disorders-a prospective study. *Disabil Rehabil* 2009;31(3):227-36. doi: 10.1080/09638280801912030
36. Nicholas MK. The pain self-efficacy questionnaire: Taking pain into account. *Eur J Pain* 2007;11(2):153-63.
37. Horowitz M, Wilner N, Alvarez W. Impact of Event Scale: a measure of subjective stress. *Psychosom Med* 1979;41(3):209-18.
38. Beck JG, Grant DM, Read JP, et al. The impact of event scale-revised: psychometric properties in a sample of motor vehicle accident survivors. *J Anxiety Disord* 2008;22(2):187-98. doi: 10.1016/j.janxdis.2007.02.007
39. Creamer M, Bell R, Failla S. Psychometric properties of the Impact of Event Scale - Revised. *Behav Res Ther* 2003;41(12):1489-96.

40. Walker-Bone K HN, Ntani G, Tinati T, Jones GT, Smith BH, Macfarlane GJ, Cooper C. . Chronic widespread bodily pain is increased among individuals with history of fracture: findings from UK Biobank. . *Arch Osteoporos* 2016;11:1.
41. Barbero M, Mores, Leoni D, Gatti R, Egloff M, Falla D. Test-retest reliability of pain extent and pain location using a novel method for pain drawing analysis. *European Journal of Pain* 2015;19:1129-38.
42. Baker SP, O'Neill B. The injury severity score: an update. *J Trauma* 1976;16(11):882-5.
43. Akmal M, Trivedi R, Sutcliffe J. Functional outcome in trauma patients with spinal injury. *Spine (Phila Pa 1976)* 2003;28(2):180-5. doi: 10.1097/01.BRS.0000041577.12233.96
44. Baker SP, O'Neill B, Haddon W, Jr., et al. The injury severity score: a method for describing patients with multiple injuries and evaluating emergency care. *J Trauma* 1974;14(3):187-96.
45. Linn S. The injury severity score--importance and uses. *Ann Epidemiol* 1995;5(6):440-6.
46. Sears JM, Blanar L, Bowman SM. Predicting work-related disability and medical cost outcomes: a comparison of injury severity scoring methods. *Injury* 2014;45(1):16-22. doi: 10.1016/j.injury.2012.12.024
47. NICE. Low back pain and sciatica in over 16s: assessment and management, NICE guideline [NG59]: National Institute for Health and Care Excellence, 2016.
48. Kamaleri Y, Natvig B, Ihlebaek CM, et al. Change in the number of musculoskeletal pain sites: A 14-year prospective study. *Pain* 2009;141(1-2):25-30. doi: 10.1016/j.pain.2008.09.013
49. Clay FJ, Watson WL, Newstead SV, et al. A systematic review of early prognostic factors for persisting pain following acute orthopedic trauma. *Pain Res Manag* 2012;17(1):35-44.
50. Von Korff M, Ormel J, Keefe FJ, et al. Grading the severity of chronic pain. *Pain* 1992;50(2):133-49.
51. Jensen MP, McFarland CA. Increasing the reliability and validity of pain intensity measurement in chronic pain patients. *Pain* 1993;55(2):195-203.
52. Jensen MP, Turner, J.A. and Romano, J.M. . What is the maximum number of levels needed in pain intensity measurement? . *Pain* 1994;58:387–92.
53. Jensen MP, Turner, L.R., Turner, J.A. and Romano, J.M. The use of multiple-item scales for pain intensity measurement in chronic pain patients. *Pain* 1996;67:35–40.
54. Jensen MP, Turner JA, Romano JM, et al. Comparative reliability and validity of chronic pain intensity measures. *Pain* 1999;83(2):157-62.
55. Von Korff M. Epidemiological and survey methods – assessment of chronic pain. In Turk, D.C. and Melzack, R., editors, Handbook of Pain Assessment. . New York, USA: Guildford Press 2001:603-18.
56. Farrar JT, Young JP, Jr., LaMoreaux L, et al. Clinical importance of changes in chronic pain intensity measured on an 11-point numerical pain rating scale. *Pain* 2001;94(2):149-58.
57. Salaffi F, Stancati A, Silvestri CA, et al. Minimal clinically important changes in chronic musculoskeletal pain intensity measured on a numerical rating scale. *Eur J Pain* 2004;8(4):283-91. doi: 10.1016/j.ejpain.2003.09.004
58. Childs JD, Piva SR, Fritz JM. Responsiveness of the numeric pain rating scale in patients with low back pain. *Spine (Phila Pa 1976)* 2005;30(11):1331-4.
59. Ostelo RW, Deyo RA, Stratford P, Waddell G, Croft P, Von Korff M, Bouter LM, Henrica C. Interpreting change scores for pain and functional status in low back pain: towards international consensus regarding minimal important change. *Spine* 2008;33(1):90-94.
60. Michener LA, Snyder AR, Leggin BG. Responsiveness of the numeric pain rating scale in patients with shoulder pain and the effect of surgical status. *J Sport Rehabil* 2011;20(1):115-28.
61. Masters Steedman S, Middaugh SJ, Kee WG, et al. Chronic-pain medications: equivalence levels and method of quantifying usage. *Clin J Pain* 1992;8(3):204-14.
62. Harden RN, Weinland SR, Remble TA, et al. Medication Quantification Scale Version III: update in medication classes and revised detriment weights by survey of American Pain Society Physicians. *J Pain* 2005;6(6):364-71. doi: 10.1016/j.jpain.2005.01.350

63. Gallizzi M GC, Harden RN, Stanos S, Khan A. Medication Quantification Scale Version III: internal validation of detriment weights using a chronic pain population. . *Pain Pract* 2008;8(1):1-4.
64. Stormo KJ, Kee WG, Steedham S, et al. Medication quantification scores and evaluation of patient pharmacology profiles. *Current Review of Pain* 1998;2(3):171–74.
65. Rosenbloom BN, Katz J, Chin KY, et al. Predicting pain outcomes after traumatic musculoskeletal injury. *Pain* 2016;157(8):1733-43. doi: 10.1097/j.pain.0000000000000580
66. Freynhagen R, Baron R, Gockel U, et al. painDETECT: a new screening questionnaire to identify neuropathic components in patients with back pain. *Curr Med Res Opin* 2006;22(10):1911-20. doi: 10.1185/030079906X132488
67. Cappelleri JC, Koduru V, Bienen EJ, et al. A cross-sectional study examining the psychometric properties of the painDETECT measure in neuropathic pain. *J Pain Res* 2015;8(8):159-67. doi: 10.2147/JPR.S80046
68. Keller T, Freynhagen R, Tolle TR, et al. A retrospective analysis of the long-term test-retest stability of pain descriptors of the painDETECT questionnaire. *Curr Med Res Opin* 2016;32(2):343-9. doi: 10.1185/03007995.2015.1125869
69. Tampin B, Bohne T, Callan M, et al. Reliability of the English version of the painDETECT questionnaire. *Curr Med Res Opin* 2017;33(4):741-48. doi: 10.1080/03007995.2017.1278682
70. Sadosky A, Koduru V, Bienen EJ, et al. Characterizing individual painDETECT symptoms by average pain severity. *Clinicoecon Outcomes Res* 2016;8:361-6. doi: 10.2147/CEOR.S105402
71. Cappelleri JC, Koduru V, Bienen EJ, et al. Mapping painDETECT, a neuropathic pain screening tool, to the EuroQol (EQ-5D-3L). *Qual Life Res* 2017;26(2):467-77. doi: 10.1007/s11136-016-1379-2
72. O'Brien M. Aids to the Examination of the Peripheral Nervous System, 5th Edition: Saunders (Elsevier) 2010.
73. Palmer ST, Martin DJ, Steedman WM, Ravey J, S. C-and Aδ-fibre mediated thermal perception: response to rate of temperature change using method of limits. . *Somatosensory & motor research* 2000;17(4):325-33.
74. Chesterton LS SJ, Wright CC, Foster NE. Interrater reliability of algometry in measuring pressure pain thresholds in healthy humans, using multiple raters. *Clin J Pain* 2007;23(9):760-6.
75. Knutti IA SM, Opsommer E. Test-retest reliability of thermal quantitative sensory testing on two sites within the L5 dermatome of the lumbar spine and lower extremity. *Neurosci Lett* 2014;5(579):157-62.
76. Greenspan JD, McGillis SL. Stimulus features relevant to the perception of sharpness and mechanically evoked cutaneous pain. *Somatosens Mot Res* 1991;8(2):137-47.
77. Sarlani E, Greenspan JD. Gender differences in temporal summation of mechanically evoked pain. *Pain* 2002;97(1-2):163-9.
78. Nie H, Arendt-Nielsen L, Andersen H, et al. Temporal summation of pain evoked by mechanical stimulation in deep and superficial tissue. *J Pain* 2005;6(6):348-55. doi: 10.1016/j.jpain.2005.01.352
79. Nie H, Arendt-Nielsen L, Madeleine P, et al. Enhanced temporal summation of pressure pain in the trapezius muscle after delayed onset muscle soreness. *Exp Brain Res* 2006;170(2):182-90. doi: 10.1007/s00221-005-0196-6
80. Woolf CJ, Bennett GJ, Doherty M, et al. Towards a mechanism-based classification of pain? *Pain* 1998;77(3):227-9.
81. Whicher JT ES. Acute phase proteins. *Hosp Update* 1990:899-905.
82. Gosling P, Dickson GR. Serum c-reactive protein in patients with serious trauma. *Injury* 1992;23(7):483-6.
83. Sturmer T, Raum E, Buchner M, et al. Pain and high sensitivity C reactive protein in patients with chronic low back pain and acute sciatic pain. *Ann Rheum Dis* 2005;64(6):921-5. doi: 10.1136/ard.2004.027045

84. Pepys MB, Hirschfield GM. C-reactive protein: a critical update. *J Clin Invest* 2003;111(12):1805-12. doi: 10.1172/JCI18921
85. Giannoudis PV HF, Pape HC. . Inflammatory serum markers in patients with multiple trauma. Can they predict outcome? *J Bone Joint Surg Br* 2004;86(3):313-23.
86. Barma M, Goodbrand, J.A., Donnan, P.T., McGilchrist, M.M., Frost, H., McMurdo, M.E. and Witham, M.D. Slower Decline in C-Reactive Protein after an Inflammatory Insult Is Associated with Longer Survival in Older Hospitalised Patients. . *PloS one* 2016;11(7):e0159412.
87. Krysko DV, Agostinis P, Krysko O, et al. Emerging role of damage-associated molecular patterns derived from mitochondria in inflammation. *Trends Immunol* 2011;32(4):157-64. doi: 10.1016/j.it.2011.01.005
88. Simmons JD, Lee YL, Mulekar S, et al. Elevated levels of plasma mitochondrial DNA DAMPs are linked to clinical outcome in severely injured human subjects. *Ann Surg* 2013;258(4):591-6; discussion 96-8. doi: 10.1097/SLA.0b013e3182a4ea46
89. Yamanouchi S, Kudo D, Yamada M, et al. Plasma mitochondrial DNA levels in patients with trauma and severe sepsis: time course and the association with clinical status. *J Crit Care* 2013;28(6):1027-31. doi: 10.1016/j.jcrc.2013.05.006
90. Foster MA. Steroids and immunity from injury through to rehabilitation (SIR Study). University of Birmingham, 2013.
