# Supplementary material for: Development of a screening tool to predict the risk of chronic pain and disability following musculoskeletal trauma: protocol for a prospective observational study in the United Kingdom
Source: BMJ Open. 2018 Apr 28;8(4):e017876. doi: 10.1136/bmjopen-2017-017876 (PMC5931282; doi:10.1136/bmjopen-2017-017876)
Supplement: Author's manuscript [file bmjopen-2017-017876.draft_revisions.pdf]

BMJ Open is committed to open peer review. As part of this commitment we make the peer review history of every article we publish publicly available.

When an article is published we post the peer reviewers' comments and the authors' responses online. We also post the versions of the paper that were used during peer review. These are the versions that the peer review comments apply to.

The versions of the paper that follow are the versions that were submitted during the peer review process. They are not the versions of record or the final published versions. They should not be cited or distributed as the published version of this manuscript.

BMJ Open is an open access journal and the full, final, typeset and author-corrected version of record of the manuscript is available on our site with no access controls, subscription charges or pay-per-view fees (http://bmjopen.bmj.com).

If you have any questions on BMJ Open's open peer review process please email info.bmjopen@bmj.com

BMJ Open

DEVELOPMENT OF A PROGNOSTIC SCREENING TOOL TO PREDICT THE RISK OF CHRONIC PAIN AND DISABILITY FOLLOWING MUSCULOSKELETAL TRAUMA: PROTOCOL FOR A PROSPECTIVE OBSERVATIONAL STUDY

Journal:	*BMJ Open*
Manuscript ID	bmjopen-2017-017876
Article Type:	Protocol
Date Submitted by the Author:	25-May-2017
Complete List of Authors:	Rushton, Alison; University of Birmingham, School of Sport, Exercise and Rehabilitation Sciences<br>Evans, David; University of Birmingham, School of Sport, Exercise and Rehabilitation Sciences<br>Middlebrook, Nicola; University of Birmingham, School of Sport, Exercise and Rehabilitation Sciences<br>Heneghan, Nicola; University of Birmingham, School of Sport, Exercise and Rehabilitation Sciences<br>Falla, Deborah; University of Birmingham, School of Sport, Exercise and Rehabilitation Sciences
Keywords:	Musculoskeletal trauma, Precision rehabilitation, Pain mechanisms

For peer review only

SCHOLARONE™
Manuscripts

TITLE PAGE

DEVELOPMENT OF A PROGNOSTIC SCREENING TOOL TO PREDICT THE RISK OF CHRONIC PAIN AND DISABILITY FOLLOWING MUSCULOSKELETAL TRAUMA: PROTOCOL FOR A PROSPECTIVE OBSERVATIONAL STUDY

Rushton A[1,2], Evans DW[1,2], Middlebrook N[1,2], Heneghan N[1], Falla D[1,2].

Affiliations

[1] Centre of Precision Rehabilitation for Spinal Pain
School of Sport, Exercise and Rehabilitation Sciences
College of Life and Environmental Sciences
University of Birmingham
Edgbaston
Birmingham
B15 2TT
United Kingdom

[2] NIHR Surgical Reconstruction & Microbiology Research Centre
University of Birmingham
Edgbaston
Birmingham
B15 2TT
United Kingdom

Correspondence:

Professor Deborah Falla
Centre of Precision Rehabilitation for Spinal Pain
School of Sport, Exercise and Rehabilitation Sciences,
College of Life and Environmental Sciences,
University of Birmingham,
Birmingham, B15 2TT, UK.
Tel: +44 121 415 4220
Email: d.falla@bham.ac.uk

For peer review only

ABSTRACT

Introduction

Pain is an expected and appropriate experience following traumatic musculoskeletal injury. By contrast, chronic pain and disability are unhelpful yet common sequelae of trauma-related injuries. Presently, the mechanisms that underlie the transition from acute to chronic disabling post-traumatic pain are not fully understood. Such knowledge would facilitate the development and implementation of precision rehabilitation approaches that match interventions to projected risk of recovery, with the aim of preventing poor long-term outcomes. The aim of this study is to identify a set of prognostic factors to identify patients at risk of developing ongoing post-traumatic pain and disability following acute musculoskeletal trauma. To achieve this, we will use a unique and comprehensive combination of patient-reported outcome measures, psychophysical testing and biomarkers.

Methods/analysis

A prospective observational study will recruit two temporally staggered cohorts [n=250 each cohort; at least 10 cases per candidate predictor] of consecutive acute musculoskeletal trauma patients aged ≥16 years, who are emergency admissions into a Major Trauma Centre in the United Kingdom, with an episode inception defined as the traumatic event. The first cohort will identify prognostic factors to develop a screening tool to predict development of chronic and disabling pain, and the second will allow evaluation of the predictive performance of the tool [validation]. The outcome being predicted is an individual's absolute risk of poor outcome measured at 6 months follow-up using the Chronic Pain Grade Scale [poor outcome ≥Grade II]. Candidate predictors encompass the four primary mechanisms of pain: *nociceptive* [e.g. injury location], *neuropathic* [e.g. painDETECT], *inflammatory* [biomarkers], and *central hypersensitivity* [e.g. quantitative sensory testing]. Concurrently,

For peer review only - http://bmjopen.bmj.com/site/about/guidelines.xhtml

patient-reported outcome measures will assess general health and psychosocial factors [e.g. Pain Self-Efficacy]. Risk of poor outcome will be calculated using multiple variable regression analysis.

Ethics and dissemination

NHS Research Ethics Committee and institutional R&D approval are in progress.

Keywords

Keywords: Pain mechanisms, Precision rehabilitation, Musculoskeletal trauma

Funding: NIHR SRMRC

Word count

3169 [excluding tables]

For peer review only - http://bmjopen.bmj.com/site/about/guidelines.xhtml

STRENGTHS AND LIMITATIONS OF THIS STUDY

- A comprehensive array of outcome measures will allow the development and validation of a prognostic tool to predict development of chronic and disabling pain following trauma
- Identifying prognostic factors related to poor outcome of pain and disability outcome will facilitate targeting of effective interventions
- The prospective design of the study enables control of unwarranted influences, and enables a stronger case for inferring causal relationships
- Other candidate predictors may have been useful to include [e.g. microRNA biomarkers], but their mechanistic functions and temporal progression are not yet sufficiently clear to justify their use

For peer review only

INTRODUCTION

Pain is an expected and appropriate experience that usually follows traumatic injury.[1] By contrast, chronic pain and disability are unhelpful but common sequelae of trauma-related injuries.[2] Gaining an understanding of why some people develop chronic and disabling post-traumatic pain is therefore a priority for individual patients, the military and society at large. Notwithstanding, the mechanisms that underlie the transition from acute to chronic disabling post-traumatic pain are not fully understood. Such knowledge would facilitate the development and implementation of a clinical pathway of care that matches interventions to projected risk of poor recovery, with the aim of preventing poor long-term outcomes. This project stems from advances in knowledge relating to the assessment and management of pain[3] and the quantification of potential prognostic factors to inform personalised rehabilitation.

Few studies have specifically explored prognostic factors for recovery, whether poor or good, following physical trauma. Of those that have, psychosocial variables, such as anxiety, depression and post-traumatic stress, have so far been identified as the strongest predictors of outcome.[4-7] However, only a limited number of variables have hitherto been evaluated as potential prognostic factors. Indeed, current opinion regarding pain mechanisms[8] suggests that the development of chronic pain and disability cannot be entirely attributable to psychosocial factors. This is consistent with research in primary care that has identified prognostic factors for poor outcome across a range of musculoskeletal pain conditions[9], which include: widespread pain, high functional disability, high pain intensity, long pain duration, high depression/anxiety, presence of previous pain episodes, movement restriction, and poor coping strategies. Moreover, developments in the mechanistic understanding of

For peer review only - http://bmjopen.bmj.com/site/about/guidelines.xhtml

pain[10-12] suggest that other measures [e.g. indicators of central sensitisation, inflammatory activity] may have potential predictive utility, especially in an acute trauma population.

Aims of study

Using a unique combination of: 1) established self-report questionnaires, including measures of pain characteristics, post-traumatic stress, depression and anxiety, catastrophizing, fear of movement, quality of life and self-efficacy; 2) premorbid neuropsychobiological status; together with 3) psychophysical methods to quantify current physical functioning, and indicators of active pain-related mechanisms; we aim to find a set of prognostic factors to identify patients at risk of developing ongoing post-traumatic pain and disability following acute musculoskeletal trauma. This will subsequently inform the feasibility of developing and evaluating a new clinical care pathway of precision rehabilitation [targeted management] that matches interventions to the predicted risk of poor recovery.

Objectives

1) Identify prognostic factors for poor outcome [chronic pain and disability at 6-months post-injury] following acute musculoskeletal trauma.
2) Develop a predictive model to inform a screening tool to identify the predicted risk of poor recovery [transition from acute post-traumatic pain to chronic pain and disability].
3) Estimate the predictive performance of the screening tool through validation of the model in a separate data set.

For peer review only - http://bmjopen.bmj.com/site/about/guidelines.xhtml

METHODS AND ANALYSIS

Source of data

The study will be a prospective, observational study of two temporally staggered cohorts of trauma patients, who are emergency department admissions into a Major Trauma Centre in the United Kingdom, with an episode inception defined as the traumatic event [Figure 1]. The first cohort will facilitate development of the prediction model to inform the screening tool, and the second will enable validation of the prediction model through evaluation of the predictive performance of the model and tool.[13 14] The prospective design enables control of unwarranted influences, and enables a stronger case for inferring causal relationships. The nature of the study necessitates predictive statistical modelling.[15] This protocol is written in line with the TRIPOD statement,[16] in which recommendations are given for the reporting of prediction model development and validation.

Prognostic data will be collected at baseline, defined as commencing within 24 hours of recruitment. The outcome data will be collected at 6-months post-injury [working definition of chronic pain];[17] ; the point of evaluation of an individual's absolute risk of poor outcome. In addition, selected data will be measured at 3 and 12-months post-injury to explore the clinical course of recovery following injury in the shorter and longer term.

For peer review only - http://bmjopen.bmj.com/site/about/guidelines.xhtml

Figure 1. Study design

[Insert Figure 1 here].

Participants

Participants will be recruited from the register of a Major Trauma Centre in the United Kingdom for a period of up to 24 months [planned start date September 2017]. All consecutive eligible patients will be approached for recruitment until the sample size is achieved.

Eligibility criteria

Inclusion criteria: Adult patients aged ≥16 years who are emergency department admissions into the Major Trauma Centre, with their main criteria for admission being acute musculoskeletal trauma in the preceding 14 days, and a capacity to use and understand written and verbal English language. Key reasons for the 14-day criterion are to be inclusive of patients with severe trauma who are likely to be in intensive care for >1 week, patients requiring surgery as a result of the trauma, fractures that are likely to lead to complications [e.g. ribs], multiple limb fractures, and haemorrhage.

Exclusion criteria: Exclusions will be made where the patient's primary injury is not musculoskeletal [e.g. where the primary injury is to the head], a score of ≤13 on the Glasgow Coma Scale [18] [a 15-item measure, routinely taken at admission in trauma patients, with adequate reliability [19]], where there is evident brain or central nervous system injury or

For peer review only - http://bmjopen.bmj.com/site/about/guidelines.xhtml

impairment, long-term neurological conditions [such as brain tumour, multiple sclerosis, Alzheimer's and Parkinson's diseases, etc], the presence of an ongoing rheumatological or inflammatory condition, comorbid cancer or infection, prolonged use of corticosteroids, refusal or lacking capacity to provide written informed consent, or deemed to be vulnerable by the recruitment research nurse [e.g. severe cognitive impairment, terminal illness, or severe mental illness].

Withdrawals: Participants will be informed that they are free to withdraw from the study at any time, without needing to provide reason. In the event of death within 3-months of being recruited, participants will be automatically withdrawn from the study and the primary prognostic analysis. Baseline characteristics of withdrawn participants will be compared to those of retained participants to assess for any differences.

Recruitment

Based on feasibility data, it is estimated that at least 1,000 eligible trauma patients will be approachable for recruitment over a 24-month period, and that 50% would be expected to consent to participation. A dedicated team of research nurses will be available to recruit patients 7-days per week [from 0700 to 1930]. A recruiting research nurse will provide the participant information sheet and, and following an opportunity for patients to seek additional information and ask questions, will ask the patient for their written informed consent. Patients will be informed that they are not only providing consent for future data to be collected, but also for the use of data already routinely collected during their stay in hospital. Once consent is gained and the participant recruited, the research nurse will collect baseline self-reported data via questionnaires [Table 1]. One of the study team will be

For peer review only

notified of the recruitment, and after a minimum 1 hour lead time following the informed consent process [to reduce patient burden] will visit the patient at their bedside to collect the physical baseline data.

Outcome

The outcome for the prediction model is an individual's absolute risk of poor outcome [chronic pain and disability] at 6-months post-injury. Outcome will be measured using the Chronic Pain Grade Scale [CPGS],[20] which combines pain intensity and pain-related disability over the preceding 6-months into a single measure of pain *severity*. The CPGS has previously been used to assess the severity of body-wide chronic pain in general population,[21] primary care[22] and post-trauma[23] populations. Each item of the CPGS relates to at least one of the three categories of the International Classification of Functioning, Disability and Health [ICF] [24]: impairment, activity limitations and restricted participation. Furthermore, all ICF categories are encompassed by the CPGS.[25] The CPGS is a unidimensional scale, with good internal consistency across different pain populations; Cronbach's alpha of 0.84 to 0.91 in back pain, 0.79 for headache, and 0.84 for temporomandibular pain.[20 26] With regards to construct validity, cross-sectional and longitudinal studies of general practice patients have shown high scores on the CPGS, indicating greater chronic pain, to be associated with higher rates of unemployment, greater pain impact scale scores, greater use of opioid analgesics and physician visits, depressed mood, and lower self-rated health status.[20 26 27] For convergent validity, the CPGS has been found to have good correlation with equivalent dimensions of the SF-36.[26 27] In terms of responsiveness, changes in score over time in patients with chronic musculoskeletal pain correlated significantly with changes in SF-36 scores.[28] The CPGS has

For peer review only - http://bmjopen.bmj.com/site/about/guidelines.xhtml

also been shown to have good test-retest reliability in primary care patients with back pain [weighted kappa 0.81, 95% CI 0.65, 0.98].[26]

Although pain persistence is not used in assigning pain grade, a measure of pain days in the prior 6-months is included in the CPGS.[29] The responses on the remaining 7-items are used for computing scores for the 3 subscales of the CPGS:[20] characteristic pain intensity, disability score, and disability points. The characteristic pain intensity score [range: 0-100] is obtained by calculating the mean of 3 pain intensity measurements: 'at the present time', the 'worst pain' in the preceding 6 months, and the 'average' pain over the preceding 6 months. The disability score [range: 0-100] is obtained through the mean ratings of how much the pain has interfered in performing activities of daily living, recreational, social and family activities, and work [including housework] activities in the last 6-months. The disability points are scored 0-3 and are derived from a combination of ranked categories of the number of disability days [the number of days that the respondent was away from usual activities in the preceding 6 months due to pain] and disability score. Based on these scores, the participant's chronic pain and disability status can then be classified into one of the 5 ordinal categories of chronic pain severity:[20] no pain [Grade 0], low disability and low intensity pain [Grade I], low disability and high intensity pain [Grade II], high disability and moderately limiting intensity pain [Grade III], and high disability and severely limiting intensity pain [Grade IV]. As in previous studies, poor outcome will be defined as Grade ≥II.[22 30-33]

Candidate predictors

Candidate predictors have been selected that are: [1] reliable and valid measures of their domain, and [2] have a theoretical association with the development of chronic pain. Both modifiable and non-modifiable candidate predictors are included. Candidate predictors

For peer review only - http://bmjopen.bmj.com/site/about/guidelines.xhtml

are summarised in Table 1, with further detail in Supplementary file S1. Table 1 also details important data that will be measured at 3, 6 and 12-months post-injury to explore the clinical course of recovery following injury in the shorter and longer term.

Table 1: Summary of data collection at different assessment points

Candidate predictor	Measure / data item	Baseline *≤14 days post-trauma*	3 months *Clinical course*	6 months *Outcome assessment point / clinical course*	12 months *Clinical course*
**General patient characteristics including premorbid neuropsychological status**					
Age	In years	√			
Gender	Female / male / other	√			
Body Mass Index [BMI]	Calculated from height and weight measurements	√			
Education	Highest educational level attained	√			
Employment status	Full-time/ part-time / not working / retired / student Employed / self-employed	√	√	√	√
Circumstance of trauma	Military / civilian	√			
Previous history of musculoskeletal pain and injury	Patient history data from patient recollection and medical records	√			
Comorbidity of other health problems	Patient history data from patient recollection and medical records	√			
Premorbid physical health	Patient history data from patient recollection and medical records	√			
Premorbid psychological health	Patient history data from patient recollection and medical records	√			
Number of days in hospital	Intensive care / ward / total	√			
**Quality of life and physical functioning**					
General health	36-item Short Form Health Survey [SF-36][34]	√	√	√	√
Health-related quality of life	EQ-5D-5L[35]	√	√	√	√
Self-care and mobility during activities of daily living	Barthel Index of Activities of Daily Living [36]	√			
Sleep quality	Subjective Health Complaints Inventory	√	√	√	√
Brain/CNS impairment	Glasgow Coma Scale [18]	√			
**Psychosocial features**					
Anxiety and depression	Hospital Anxiety and Depression Scales [HADS][37]	√	√	√	√
Rumination, magnification or helplessness about	Pain Catastrophizing Scale[38]	√	√	√	√

For peer review only - http://bmjopen.bmj.com/site/about/guidelines.xhtml

controlling pain					
Fear of movement or fear of injury or re-injury during movement	Tampa Scale of Kinesiophobia [TSK-11][39]	√	√	√	√
Confidence in ability to perform activities despite pain	Pain Self-Efficacy Questionnaire[40]	√	√	√	√
Subjective post-traumatic distress	Impact of Event Scale revised [IES-R][41]	√	√	√	√
**Injury characteristics**					
Time of injury/incident	Hospital record data	√			
Injury location	Adapted pain drawings, based on hospital record data	√			
Tissues damaged	Based on imaging data and hospital records<br>Fractures<br>Penetrating / non-penetrating injury / both	√			
Surgery required	Location and post-injury timing of surgery	√			
Assisted mechanical ventilation required	Yes / no / duration	√			
Severity of injury	Injury Severity Score[42]	√			
**Pain characteristics**					
Chronic pain severity	Chronic Pain Grade Scale[20]			√	√
Pain intensity	11-point [0-10] Numerical Rating Scale, relating to current pain, from 'no pain' to 'pain as bad as could be' [collected as part of the Chronic Pain Grade Scale]	√[1]	√	√	√
Pain medication intake [type and dosage]	Medication Quantification Score[43-45]	√[1]			
Pain and injury extent	Electronic pain drawing[46]	√[1]			
Self-reported features of neuropathic pain	painDETECT[47]	√[1]	√	√	√
**Quantitative sensory testing**					
Heat pain threshold	Evaluation of pain threshold using a heat stimulus	√[1]			
Cold pain threshold	Evaluation of pain threshold using a cold stimulus	√[1]			
Pressure pain threshold	Evaluation of pain threshold using a pressure stimulus	√[1]			
Temporal summation	Evaluation of pain intensity in response to repetitive pressure stimuli	√[1]			
**Biomarkers**					
C-reactive protein [CRP]	Serum levels of CRP [via blood tests]	√[1]			

[1] Measurements to be taken at baseline [recruitment] and repeatedly whilst in hospital ward

For peer review only - http://bmjopen.bmj.com/site/about/guidelines.xhtml

Data handling

Baseline self-reported questionnaire data will be collected by the recruiting research nurse. Blood test data will be collected and processed through the research nurse team. The remaining baseline data [pain and injury drawings, quantitative sensory testing] will be collected by one of three assessors from the study team, trained to administer these augmented assessments. An inter-rater reliability study [across the 3 assessors] will be initially conducted in a trauma population to inform definitive testing protocols. The order of data collection will be randomised according to the modality of the testing to prevent order effects. Self-reported questionnaires will be formatted using FORMIC document recognition software, so that data can be acquired and scanned directly into an electronic database. Data will be checked for completeness and cross-checked for accuracy. All outcome measure data will be collated into an anonymised database and stored securely for a minimum period of 10-years at the University of Birmingham, in line with Research Governance procedures. Participants will receive usual care, and interventions received will be recorded for descriptive analysis. Data will be analysed using IBM SPSS Statistics. Participant identifiable information will be kept on an encrypted electronic database file, stored in line with current United Kingdom data protection legislation, and only accessible by the Research Nurse Lead who will not be involved in data analysis.

Sample size

In predictive modelling, a larger sample size enables lower bias and variance, and permits the prospective prediction of new observations.[15] The sample size will provide at

For peer review only - http://bmjopen.bmj.com/site/about/guidelines.xhtml

least 10 cases per candidate predictor, to adequately power the final regression analysis.[48 49] Data will be collected for an estimated 250 participants per cohort [n=500 total].

Statistical analysis methods and management of missing data

Potentially eligible patients, numbers examined for eligibility, confirmed eligible, recruited into the study, completing follow-up, and analysed will be reported in a flow diagram. Reasons for non-participation, exclusion, drop-outs or withdrawal [e.g. death] will be reported at each stage. Participant characteristics will be descriptively presented. For each variable of interest, the number of participants with missing data will be reported. The correlation between candidate prognostic factors will be calculated at baseline.

For peer

Statistical modelling for prediction has been planned *a priori*. To explore the influence of each prognostic factor on poor outcome at 6 months, both linear and logistic [to dichotomise the 6-month score into low and high risk] multivariable regression models will be fitted and mean differences or odds ratios for each candidate prognostic factor reported, adjusted for other factors and accounting for clustering [e.g. level of injury severity]. If necessary, multiple imputation[50] will be used to deal with missing outcome data. The characteristics of those patients with and without 6-month data will also be compared, to inform whether patients with no 6-month data were missing at random. Multivariable analysis will initially include all candidate prognostic factors, and full results reported. Reduced multivariable analyses will be considered if necessary [e.g. removing one of two candidate prognostic factors that are highly correlated at baseline], to examine the robustness of the conclusions.

For peer review only - http://bmjopen.bmj.com/site/about/guidelines.xhtml

Risk groups and development of the prognostic screening tool

The prognostic model will be used to develop a risk stratification tool to inform an individual's absolute risk of poor outcome. The stratification tool will inform clinical decision-making for precision rehabilitation. Selection of items for the model will include those factors that are statistically significantly [$p<0.05$] associated with poor outcome according to the full multivariable regression analysis, and those deemed clinically important to retain [regardless of statistical significance] to improve face validity for clinicians. The regression model with included prognostic factors will be fitted to the data from the first of the two cohorts to obtain a final set of parameter estimates [i.e. alpha and beta terms], which will be used to form the model. An important requirement of the stratification tool is that it should be brief to facilitate use in clinical practice. Thus, we will look to simplify the model where possible to facilitate its use, but without important reduction in its prognostic ability in terms of calibration and discrimination. For example, if multi-item questionnaire scores are included in the model, then we will evaluate whether just one of the questionnaire items is sufficient. Ideally, this process will result in a full and simplified model.

Development versus validation

For validation of the model, data from the second of the two cohorts will be compared to that of the first to enable analysis of the distribution of important variables; inclusive of demographic, predictor and outcome variables. An estimate of the predictive performance of the screening tool will be established through validation of the model using data from the second cohort. Data in both cohorts will be consistent in terms of setting, eligibility criteria, outcome, and predictors.

DISCUSSION

There is an urgent need for a robust prognostic study to predict the transition from acute to chronic pain in a musculoskeletal trauma population. Using such a comprehensive array of outcome measures will allow the development and validation of a prognostic tool to predict development of chronic and disabling pain, and begin the process of identifying appropriate and precision interventions.

The candidate predictors used in this study have been chosen to be as comprehensive as possible, based on current knowledge of pain science. Other candidate predictors were considered [e.g. microRNA biomarkers], but their mechanistic functions and temporal progression are not yet sufficiently clear to justify the expense of their inclusion. The combination of patient reported outcome measures, psychophysical testing and biomarkers that are included are designed to act as surrogates for the four primary mechanisms of pain: [51] [8] *nociceptive* [injury location, severity and characteristics], *neuropathic* [painDETECT tool and pain extent, *inflammatory* [biomarkers], and *central hypersensitivity* [quantitative sensory testing, painDETECT and pain extent]. In addition, other patient-reported outcome measures [e.g. pain intensity, post-traumatic stress, anxiety and depression, and self-efficacy] are included as the domains that they measure have been shown to influence prognosis for long-term outcomes in populations with pain in a range of locations.[9 22 23]

Rehabilitation is widely regarded as an important component of post-trauma healthcare;[52] however, the current position of equipoise means that precision rehabilitation has not yet been identified. Understanding mechanisms that underlie the transition from acute to chronic pain is essential to moving beyond this position. Identifying prognostic factors

For peer review only - http://bmjopen.bmj.com/site/about/guidelines.xhtml

related to poor outcome of pain and disability outcome will facilitate targeting of effective interventions. This will inform rehabilitation decision making, and facilitate improvements in clinical and cost effectiveness of rehabilitation interventions.

Limited research has identified criteria for quality in a prognostic model, but authors have identified potential quality issues to ensure methodological rigour.[53] These issues are summarised in Table 2 and incorporated into the study design to ensure low risk of bias in development and validation of the predictive model.

Table 2: Methodological decisions to improve study quality

**Criteria [53]**	**Methodological decisions to improve quality**
**Study design**	
Inception cohort	• Clear description of population<br>• Clear description of the participants at baseline
Source population	• Clear description of population<br>• Clear description of sampling frame and recruitment [method and timing]
Inclusion and exclusion criteria	• Clarity of eligibility criteria
Prospective design	• Clarity of study design
**Study attrition**	
Number of drop-outs	• Adequate participation rate<br>• Clear description of attempts to collect information on participants who dropped out<br>• Reporting numbers and reasons for loss to follow-up
Information provided on method of management of missing data	• Appropriate methods of imputation of missing data
**Prognostic factors**	
All prognostic factors described used to develop the model	• Clear definition of prognostic factors<br>• An adequate proportion of participants has complete data for the prognostic factor
Standardised or valid measurements	• The measurement of the prognostic factor is reliable and valid<br>• The measurement of the prognostic factor is the same for all participants
Linearity assumption studied	• Linearity of data will be reported
No dichotomization of prognostic variables	• Continuous variables will be reported where possible
Data presentation all prognostic factors	• Complete data will be presented

For peer review only - http://bmjopen.bmj.com/site/about/guidelines.xhtml

**Outcome measures**	
Description of outcome measures	• The outcome is clearly defined
Standardised or valid measurements	• The measurement of the outcome is reliable and valid<br>• The measurement of the outcome is the same for all participants
Data presentation of most important outcome measures	• Complete data will be presented
**Analysis**	
Presentation of univariate crude estimates	• An appropriate strategy for model building is described<br>• An adequate statistical model described
Sufficient numbers of subjects per variable	• Adequate data will be presented
Selection method of variables explained	• Sufficient data will be presented to enable assessment of the adequacy of the analytic strategy<br>• All results will be reported
Presentation of multivariate estimates	• An appropriate strategy for model building is described<br>• An adequate statistical model described
**Clinical performance / validity**	
Clinical performance	• Clinical performance of the model will be reported
Internal validation	• Internal validation will be reported
External validation	Not a focus of this study

ETHICS AND DISSEMINATION

Ethical approval will be obtained from the NHS Research Ethics Committee, and institutional R&D approval will also be obtained. The primary ethical concern is to limit distress on participants. As such, to reduce the patient burden when measuring baseline outcomes, the recruiting research nurses will administer self-reported questionnaires immediately following obtaining informed consent, and other outcomes will be measured by members of the study team at least 1 hour later. Patients will be asked to consent to not only providing new data for the study, but also for the study team to access information that had already been routinely collected by the hospital staff since the time of admission [e.g. nature and circumstances of injury, previous medical history, blood test results, pain intensity on

For peer review only - http://bmjopen.bmj.com/site/about/guidelines.xhtml

admission]. This will be fully explained to patients and explicitly detailed in the participant information sheet. All blood and tissue samples will be administered by hospital staff and the research nurse team and will be stored, tested and disposed of in accordance with current United Kingdom guidelines and regulations. Participants will be informed that they are free to withdraw from the study at any time, without needing to provide reason. In the event of death within 3 months of being recruited, participants will be automatically withdrawn from the study and the primary prognostic analysis. Baseline characteristics of withdrawn participants will be compared to those of retained participants to assess for any differences.

For peer review only

For peer review only - http://bmjopen.bmj.com/site/about/guidelines.xhtml

REFERENCES

1. Berben SA, Schoonhoven L, Meijs TH, et al. Prevalence and relief of pain in trauma patients in emergency medical services. *Clin J Pain* 2011;27(7):587-92. doi: 10.1097/AJP.0b013e3182169036
2. Williamson OD, Epi GD, Gabbe BJ, et al. Predictors of moderate or severe pain 6 months after orthopaedic injury: a prospective cohort study. *J Orthop Trauma* 2009;23(2):139-44. doi: 10.1097/BOT.0b013e3181962e29
3. Mannion RJ, Woolf CJ. Pain mechanisms and management: a central perspective. *Clin J Pain* 2000;16(3 Suppl):S144-56.
4. Holbrook TL, Anderson JP, Sieber WJ, et al. Outcome after major trauma: discharge and 6-month follow-up results from the Trauma Recovery Project. *J Trauma* 1998;45(2):315-23; discussion 23-4.
5. Holbrook TL, Anderson JP, Sieber WJ, et al. Outcome after major trauma: 12-month and 18-month follow-up results from the Trauma Recovery Project. *J Trauma* 1999;46(5):765-71; discussion 71-3.
6. Harris IA, Young JM, Rae H, et al. Predictors of general health after major trauma. *J Trauma* 2008;64(4):969-74. doi: 10.1097/01.ta.0000245972.83948.1a
7. Ponsford J, Hill B, Karamitsios M, et al. Factors influencing outcome after orthopedic trauma. *J Trauma* 2008;64(4):1001-9. doi: 10.1097/TA.0b013e31809fec16
8. Vardeh D, Mannion RJ, Woolf CJ. Toward a Mechanism-Based Approach to Pain Diagnosis. *J Pain* 2016;17(9 Suppl):T50-69. doi: 10.1016/j.jpain.2016.03.001
9. Artus M, Campbell P, Mallen CD, et al. Generic prognostic factors for musculoskeletal pain in primary care: a systematic review. *BMJ Open* 2017;7(1):e012901. doi: 10.1136/bmjopen-2016-012901
10. Latremoliere A, Woolf CJ. Central sensitization: a generator of pain hypersensitivity by central neural plasticity. *J Pain* 2009;10(9):895-926. doi: 10.1016/j.jpain.2009.06.012
11. Linley JE, Rose K, Ooi L, et al. Understanding inflammatory pain: ion channels contributing to acute and chronic nociception. *Pflugers Arch* 2010;459(5):657-69. doi: 10.1007/s00424-010-0784-6
12. CJ. W. Central sensitization: implications for the diagnosis and treatment of pain. *Pain* 2011;152(3):S2-15.

For peer review only

For peer review only - http://bmjopen.bmj.com/site/about/guidelines.xhtml

13. Altman DG, Vergouwe Y, Royston P, et al. Prognosis and prognostic research: validating a prognostic model. *BMJ* 2009;338(338):b605. doi: 10.1136/bmj.b605

14. Moons KG, Kengne AP, Grobbee DE, et al. Risk prediction models: II. External validation, model updating, and impact assessment. *Heart* 2012;98(9):691-8. doi: 10.1136/heartjnl-2011-301247

15. Shmueli G. To explain or to predict? *Statistical Science* 2010;25(3):289-310.

16. Collins GS, Reitsma, J.B., Altman, D.G. and Moons, K.G., . Transparent reporting of a multivariable prediction model for individual prognosis or diagnosis (TRIPOD): the TRIPOD statement. *BMC Medicine* 2015;13(1)

17. Merskey H BN. Classification of chronic pain : descriptions of chronic pain syndromes and

definitions of pain terms. USA: IASP Press 1994.

18. Teasdale G, Jennett B. Assessment of coma and impaired consciousness. A practical scale. *Lancet* 1974;2(7872):81-4.

19. Reith FC, Van den Brande R, Synnot A, et al. The reliability of the Glasgow Coma Scale: a systematic review. *Intensive Care Med* 2016;42(1):3-15. doi: 10.1007/s00134-015-4124-3

20. Von Korff M, Ormel J, Keefe FJ, et al. Grading the severity of chronic pain. *Pain* 1992;50(2):133-49.

21. Parsons S, Breen A, Foster NE, et al. Prevalence and comparative troublesomeness by age of musculoskeletal pain in different body locations. *Fam Pract* 2007;24(4):308-16. doi: 10.1093/fampra/cmm027

22. Muller S, Thomas E, Dunn KM, et al. A prognostic approach to defining chronic pain across a range of musculoskeletal pain sites. *Clin J Pain* 2013;29(5):411-6. doi: 10.1097/AJP.0b013e318257099e

23. Rivara FP, Mackenzie EJ, Jurkovich GJ, et al. Prevalence of pain in patients 1 year after major trauma. *Arch Surg* 2008;143(3):282-7; discussion 88. doi: 10.1001/archsurg.2007.61

24. Organization WH. The International Classification of Functioning, Disability and Health (ICF). Geneva: WHO 2001.

25. Dixon D PD, Johnston M. What does the chronic pain grade questionnaire measure? *Pain* 2007;130:249-53.

For peer review only

26. Smith BH, Penny KI, Purves AM, et al. The Chronic Pain Grade questionnaire: validation and reliability in postal research. *Pain* 1997;71(2):141-7.
27. Penny KI PA, Smith BH, Chambers WA, Smith WC. Relationship between the chronic pain grade and measures of physical, social and psychological well-being. *pain* 1999;79:275–9.
28. Elliott AM SB, Smith WC, Chambers WA. Changes in chronic pain severity over time: The Chronic Pain Grade as a valid measure. *Pain* 2000;88:303.
29. Von Korff M, Jensen MP, Karoly P. Assessing global pain severity by self-report in clinical and health services research. *Spine (Phila Pa 1976)* 2000;25(24):3140-51.
30. Von Korff M, Miglioretti DL. A prognostic approach to defining chronic pain. *Pain* 2005;117(3):304-13. doi: 10.1016/j.pain.2005.06.017
31. Von Korff M, Dunn KM. Chronic pain reconsidered. *Pain* 2008;138(2):267-76. doi: 10.1016/j.pain.2007.12.010
32. Dunn KM, Croft PR, Main CJ, et al. A prognostic approach to defining chronic pain: replication in a UK primary care low back pain population. *Pain* 2008;135(1-2):48-54. doi: 10.1016/j.pain.2007.05.001
33. Thomas E, Dunn KM, Mallen C, et al. A prognostic approach to defining chronic pain: application to knee pain in older adults. *Pain* 2008;139(2):389-97. doi: 10.1016/j.pain.2008.05.010
34. Hays RD, Sherbourne CD, Mazel RM. The RAND 36-Item Health Survey 1.0. *Health Econ* 1993;2(3):217-27.
35. Brooks R. EuroQol: the current state of play. *Health Policy* 1996;37(1):53-72.
36. Mahoney FI, Barthel DW. Functional Evaluation: The Barthel Index. *Md State Med J* 1965;14:61-5.
37. Zigmond AS, Snaith RP. The hospital anxiety and depression scale. *Acta Psychiatr Scand* 1983;67(6):361-70.
38. Sullivan JL, Bishop SR, Pivik J. The pain catastrophizing scale: development and validation. *Psychological Assessment* 1995;7:524-32.
39. Woby SR, Roach NK, Urmston M, et al. Psychometric properties of the TSK-11: a shortened version of the Tampa Scale for Kinesiophobia. *Pain* 2005;117(1-2):137-44. doi: 10.1016/j.pain.2005.05.029
40. M.K. N. The pain self-efficacy questionnaire: Taking pain into account. *Eur J Pain* 2007;11(2):153-63.

For peer review only

For peer review only - http://bmjopen.bmj.com/site/about/guidelines.xhtml

41. Beck JG, Grant DM, Read JP, et al. The impact of event scale-revised: psychometric properties in a sample of motor vehicle accident survivors. *J Anxiety Disord* 2008;22(2):187-98. doi: 10.1016/j.janxdis.2007.02.007

42. Baker SP, O'Neill B, Haddon W, Jr., et al. The injury severity score: a method for describing patients with multiple injuries and evaluating emergency care. *J Trauma* 1974;14(3):187-96.

43. Masters Steedman S, Middaugh SJ, Kee WG, et al. Chronic-pain medications: equivalence levels and method of quantifying usage. *Clin J Pain* 1992;8(3):204-14.

44. Harden RN, Weinland SR, Remble TA, et al. Medication Quantification Scale Version III: update in medication classes and revised detriment weights by survey of American Pain Society Physicians. *J Pain* 2005;6(6):364-71. doi: 10.1016/j.jpain.2005.01.350

45. Gallizzi M GC, Harden RN, Stanos S, Khan A. Medication Quantification Scale Version III: internal validation of detriment weights using a chronic pain population. . *Pain Pract* 2008;8(1):1-4.

46. Barbero M, Mores, F., Leoni, D., Gatti, R., Egloff, M., Falla, D. Test-retest reliability of pain extent and pain location using a novel method for pain drawing analysis. *European Journal of Pain* 2015;19:1129-38.

47. Freynhagen R, Baron R, Gockel U, et al. painDETECT: a new screening questionnaire to identify neuropathic components in patients with back pain. *Curr Med Res Opin* 2006;22(10):1911-20. doi: 10.1185/030079906X132488

48. Peduzzi P, Concato J, Kemper E, et al. A simulation study of the number of events per variable in logistic regression analysis. *J Clin Epidemiol* 1996;49(12):1373-9.

49. Vittinghoff E, McCulloch CE. Relaxing the rule of ten events per variable in logistic and Cox regression. *Am J Epidemiol* 2007;165(6):710-8. doi: 10.1093/aje/kwk052

50. Sterne JA, White IR, Carlin JB, et al. Multiple imputation for missing data in epidemiological and clinical research: potential and pitfalls. *BMJ* 2009;338:b2393. doi: 10.1136/bmj.b2393

51. Woolf CJ, Bennett GJ, Doherty M, et al. Towards a mechanism-based classification of pain? *Pain* 1998;77(3):227-9.

52. NICE. Rehabilitation after critical illness in adults Clinical guideline [CG83]. 2009

53. van Oort L, van den Berg T, Koes BW, et al. Preliminary state of development of prediction models for primary care physical therapy: a systematic review. *J Clin Epidemiol* 2012;65(12):1257-66. doi: 10.1016/j.jclinepi.2012.05.007

For peer review only - http://bmjopen.bmj.com/site/about/guidelines.xhtml

Authors' contributions

DF and AR are the Chief Investigators leading protocol development, analyses and dissemination. DE is the Research Fellow with responsibility for study management. NM is a Doctoral Researcher focused to this study. NH is the lead for Patient and Public Involvement. DF and AR are overseeing data analysis. All authors will contribute to data interpretation, conclusions, and dissemination. AR drafted the initial manuscript with DF. Subsequent drafts were developed with DE. All reviewers have read, contributed to, and agreed the final manuscript. DF is the guarantor.

Funding statement

This work was supported by the National Institute for Health Research (NIHR) to DF and AR.

Competing interests

There are no competing interests.

Data sharing statement

No additional data are available.

For peer review only

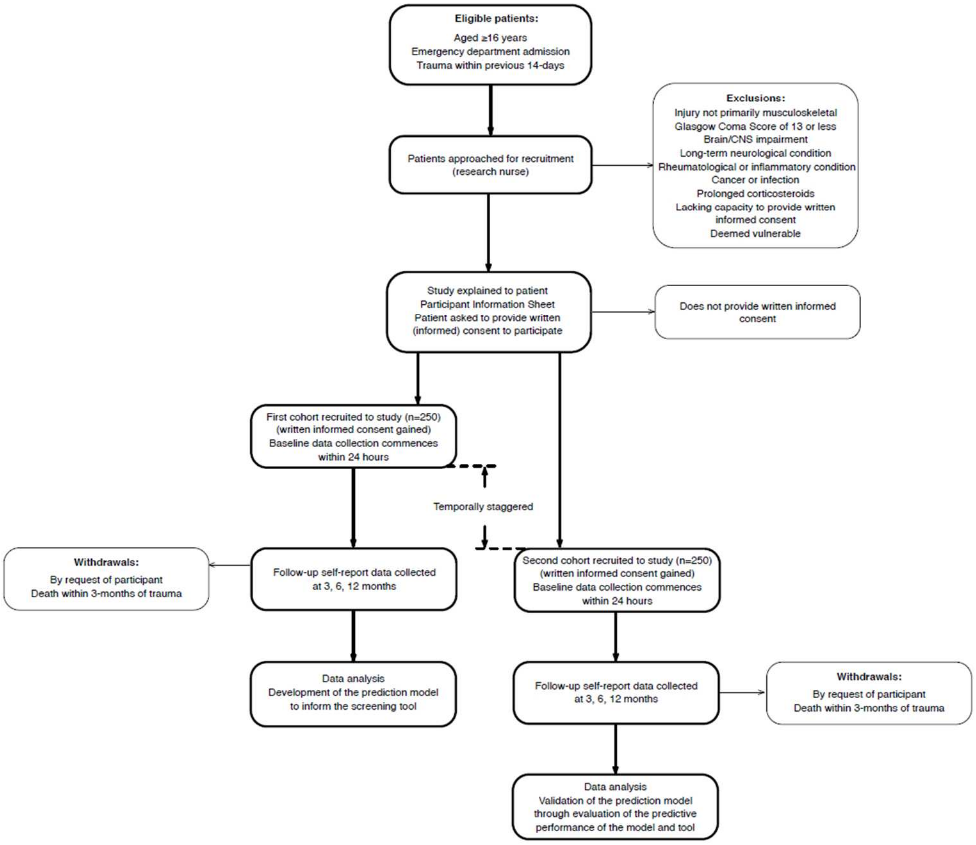

Study design

289x247mm (96 x 96 DPI)

SUPPLEMENTARY FILE 1: CANDIDATE PROGNOSTIC FACTORS

General participant characteristics

Several participant demographic features will be recorded at baseline, based on available hospital records and patient self-reported recollection, including smoking status, age, gender, height and weight to calculate body mass index [BMI], education [highest attained educational level], employment status [at the time of trauma], circumstance of trauma [military or civilian], previous history of musculoskeletal pain and injury, comorbidity of other current health problems.

Quality of life and physical functioning

36-Item Short Form Health Survey [SF-36]

The SF-36 is a self-reported measure of health-related quality of life, developed at RAND as part of the Medical Outcomes Study.[1] The 36-item questionnaire has subscales that assess physical, function, social and psychological wellbeing.[2 3] The scores can be divided into physical and mental component summary scales.[4] The SF-36 has been shown to be valid and has been tested extensively in a trauma population.[5] Ware[6] reports multiple studies showing internal consistency above 0.70, with physical and mental scores exceeding 0.90. Minimal clinically important difference has been reported as 5.5 in a musculoskeletal trauma population.[7]

EuroQol Five Dimension Scale [EQ-5D-5L]

Health-related quality of life will be quantified using the EQ-5D-5L through which 243 possible health states are converted to a single index value of range –0.594 to 1 where 1

For peer review only - http://bmjopen.bmj.com/site/about/guidelines.xhtml

is perfect health, and a visual analogue scale range 0–100, representing 'worst' to 'best' imaginable health state, respectively.[8] The EQ-5D-5L has improved inter-observer [ICC 2,1 0.57] and test-retest [ICC 2,1 0.69] reliability compared to the previous EQ-5D-3L.[9] In addition, it has less ceiling effects [20.8% reduction] and adequate convergent validity when compared with the WHO-5 [spearman rank 0.38-0.51].[10]

Barthel Index of Activities of Daily Living

The Barthel Index of Activities of Daily Living will be used to evaluate self-care and mobility during activities of daily living.[11 12] It is a 10-item ordinal scale encompassing a range of mobility physical activity tasks. Each item is related to a specific task and rated with a given number of points. A score of '0' is given for least independence/function on that item and scores above that [1 or 2] are given for increasing independence/function [range: 0-20]. The amount of time and physical assistance required to perform each task are used in determining the assigned value of each item. A higher score is associated with a greater likelihood of being able to live at home with a degree of independence following discharge from hospital. With most measurement testing performed in the stroke population, the Barthel Index has demonstrated excellent internal consistency [0.89-0.90][13] and is highly responsive in detecting changes[14] with a minimal detectable change of 4.02 and minimally clinically important difference of 1.85.[15] High correlations have been demonstrated to the Functional Independence Measure [FIM], indicating convergent validity of the measure.[16]

Subjective Health Complaints Inventory

Sleep quality will be assessed using the single-item questions from the sleep domain of the Subjective Health Complaints Inventory.[17] The Subjective Health Complaints Inventory has been shown to be a reliable measure of recording subjective health

For peer review only - http://bmjopen.bmj.com/site/about/guidelines.xhtml

complaints;[17] although no study has focused on the reliability of using the single item relating to sleep.

Psychosocial features

The Hospital Anxiety and Depression Scales [HADS]

The HADS will be used to measure depression and anxiety, and their role in the manifestation of somatic symptoms.[18] There are 7 items which produce a cumulative score [range 0–21] for the anxiety [HADS-A] and depression [HADS-D] subscales, with a higher score indicative of greater anxiety and depression.[19] HADS has been tested in multiple populations demonstrating adequate to excellent internal consistency of HADS-A [0.68-0.93] and HADS-D [0.67-0.90].[19] Standard measurement of error in a coronary heart disease population was identified as 1.37 and 1.44 for anxiety and depression scales respectively, and minimal detectable change as 3.80 and 3.99 respectively.[20] The HADS has also demonstrated excellent concurrent validity when compared to various other depression/anxiety scales.[19]

Pain Catastrophizing Scale [PCS]

The PCS will be used to provide an indication of individuals who ruminate, magnify or feel helpless about controlling their pain.[21] It is a 13-item scale, and participants are asked to indicate the degree to which they have particular thoughts and feelings when they are experiencing pain. Items are scored with ‘0’ representing ‘not at all’ and a maximum of ‘4’ represents ‘all the time’. Scores on each item are summed to yield a total score ranging 0-52. A higher score reflects greater negative pain-related thoughts, emotional distress, and pain intensity. The PCS has demonstrated excellent internal consistency in a population of low back pain patients [0.90] with a 4.6% minimal detectable change.[22] Excellent intra

For peer review only - http://bmjopen.bmj.com/site/about/guidelines.xhtml

[ICC{model not reported} 0.88], inter-rater [ICC{model not reported} 0.77] reliability has been demonstrated in a low back pain population.[23]

Tampa Scale of Kinesiophobia [TSK-11]

The TSK-11 will be used to assess fear of movement or fear of injury or re-injury during movement.[24] It is an 11-item questionnaire, eliminating psychometrically poor items from its original 17-item version,[25] thus creating a shorter questionnaire with comparable internal consistency and a 2-factor structure [activity avoidance and harm]. Each of the 11 items is measured using a 4-point scale using the end points 1 [‘totally agree’] and 4 [‘totally disagree’] [scoring range 11–44]. Higher scores indicate more fear-avoidance behaviour. The TSK-11 has demonstrated acceptable to good internal consistency in acute and chronic musculoskeletal pain populations.[24 26] Test-retest reliability has been reported as excellent with a high standardised response mean; with good construct validity in relation to changes in disability and pain.[24]

Pain Self-Efficacy Questionnaire [PSEQ]

The patient’s confidence in their ability to perform activities despite their pain will be evaluated using the PSEQ. Developed from the Self-Efficacy Scale,[27] the PSEQ consists of 10 physical and psychosocial activity items measuring from 0 [‘not at all confident’] to 6 [‘completely confident’] thus generating a total score from 0-60.[28] The PSEQ has demonstrated excellent internal consistency [0.92], internal reliability [0.93], and test-re-test correlations [r=0.73] and has demonstrated validity when compared to other self-efficacy measurements.[28] It has been used in several large population studies, for example Campbell et al.[29]

For peer review only - http://bmjopen.bmj.com/site/about/guidelines.xhtml

Impact of Event Scale Revised [IES-R]

The IES-R will be used to measure the subjective stress experienced by the participant following their traumatic event. The IES-R is a 22-item tool [range: 0-88] that consists of 8 intrusion and 8 avoidance items that are derived from the original IES,[30] with an additional 7-items assessing hyperarousal.[31] Accordingly, items correspond directly to symptoms of post-traumatic stress disorder.[31] Respondents are asked to identify a specific stressful life event and then indicate how much they were distressed or bothered during the past seven days by each 'difficulty' listed. Each item is rated on a 5-point scale ranging from 0 ['not at all'] to 4 ['extremely']. The IES-R yields a total score ranging 0 to 88 and subscale scores can also be calculated for the Intrusion, Avoidance, and Hyperarousal sub-scales. The IES-R has demonstrated good internal consistency for all subscales [intrusion 0.87-0.94, avoidance 0.84-0.97, hyperarousal 0.79-0.91].[32] High correlations have been found between the IES-R and the original scale, supporting the concurrent validity of both measures.[31]

Injury characteristics

Several measures relating to the characteristics of the sustained injury will be taken at baseline. The time of the injury will be gained from hospital records. The location of the injury/injuries will be recorded using an adapted version of previously developed pain drawing software, via a tablet computer.[33] Information relating to the tissues damaged from the injury [e.g. fractures sustained, whether the injury was penetrating, non-penetrating or both, review of available imaging data] will be gathered from hospital records, where possible. Whether the participant received surgery following their admission [where, for what and when], and whether the participant received assisted mechanical ventilation will also be recorded.

For peer review only - http://bmjopen.bmj.com/site/about/guidelines.xhtml

Injury Severity Scale [ISS]

The ISS will be retrospectively calculated for each participant, including those who withdraw. The ISS is a numerical score with a range 0-75, that is used to describe the overall severity of injury, and can be used for both multiple and single injuries. The score is calculated, based on the Abbreviated Injury Scale [AIS] scores.[34 35] Higher ISS scores have been associated with increased rates of mortality[34 36 37] and length/cost of hospital stay.[38] It is the recommended tool for summarising injury severity by the Trauma Audit and Research Network [TARN]. Both TARN and the National Institute for Clinical Excellence[39] recommend any participant with a score of >8 to be referred for rehabilitation.

Pain characteristics

Pain intensity

Pain intensity will be measured using an 11-point [0-10] Numerical Rating Scale [NRS], measuring current pain from 'no pain' to 'pain as bad as could be', from the Chronic Pain Grade Scale.[40] We will aim to assess *current* pain intensity at baseline, as frequently as every 48-hours while the participant is in hospital [depending upon participant accessibility and assessor availability], to gain accurate mean and rate-of-change data. At the 6 and 12 month assessment points, c*urrent* pain intensity, as well as *average* and *worst* pain intensity related to the preceding 6 months, will be collected as part of the Chronic Pain Grade Scale. NRS scales are sensitive, reliable and valid instruments for pain intensity measurement,[41-44] and have been recommended for use in clinical populations in preference to visual analogue scales or verbal rating scales.[45] A 30% change on a pain NRS score is considered clinically meaningful.[46-50]

For peer review only - http://bmjopen.bmj.com/site/about/guidelines.xhtml

Pain medication

The patient's pain medication [type and dosage] intake will be noted and the Medication Quantification Score [MQS], which is a reliable and validated score for quantifying analgesics, will be calculated to obtain a comparable metric for all different analgesics.[51-53] It enables characterisation of analgesics when many analgesics are involved and doses are irregular. It will be calculated for each non-opioid and opioid, based on weights assigned by medication class and dosage level [level 1 = sub-therapeutic dosage and/or on demand, level 2 = lower 50% of the therapeutic dose range, level 3 = upper 50% of the therapeutic dose range, level 4 = supra-therapeutic dose] using the 1998 detriment weights.[54] The detriment weights are summed by the dosage level to provide the final score. To provide a quantitative index for analgesics usage suitable for statistical analysis these scores will be summed.

Pain extent

All participants will be requested to complete a pain drawing indicating their pain on two body charts, one reporting a frontal view of the body and one a dorsal view. We will also ask patients to mark their 'most painful' site. Pain drawing data will be collected using custom software developed with Matlab software, as described previously.[33] The software automatically calculates the number of shaded pixels from the pain drawing, which is defined as *pain extent*. Summaries of, and relationships between, pre-defined painful regions will also be evaluated.

The painDETECT questionnaire

It is assumed that all post-trauma patients will have significant nociception at baseline, but the contribution of other pain-related mechanistic pathways will also be

For peer review only - http://bmjopen.bmj.com/site/about/guidelines.xhtml

assessed. The painDETECT questionnaire[55] will be used to facilitate the identification of neuropathic pain. It consists of 9 items [7 evaluating pain quality, 1 evaluating pain pattern, and 1 evaluating pain radiation], all of which contribute to an aggregate score [range: -1-38]. This aggregate score can be divided into three classifications that represent the likelihood of neuropathic pain: ‘unlikely’ [0-12], ‘ambiguous’ [13–18] and ‘likely’ [19–38].[55] Although developed as a screening questionnaire for neuropathic pain, painDETECT may also function as a measure of characteristics that indicate augmented central pain processing.[55] The painDETECT questionnaire has demonstrated good internal consistency [0.76][56] and excellent test-re-test reliability[57] within 1-hour of consultation [ICC{model not reported} 0.911] and 1-week post consultation [ICC{model not reported} 0.79].[58] Convergent validity has been demonstrated in comparison to pain severity,[56 59] health-related quality of life[60] and similar neuropathic pain screening tools.[55] As such, painDETECT outcomes will be measured at regular intervals while the participant is an inpatient in the hospital [subject to participant accessibility and assessor availability] to assess for emerging neuropathic pain and sensitization. Measurements will also be taken at all follow-up assessment points.

Quantitative sensory testing [QST]

QST methods will be used to assess pain sensibility, throughout which measurements will be concealed from participants. Owing to the clinical heterogeneity of the post-trauma population, precise standardisation of test sites between participants will not be possible. Instead, we have developed a standardised protocol that will be used to evaluate pain thresholds for multiple stimulus modalities [mechanical pressure, heat and cold] at the same sites in each participant. Each site where multi-modality pain threshold testing is performed will be within the receptive field of the same nerve root using described regions,[61] so that segmental cross-modality excitability may be compared. Pain thresholds will be measured at

For peer review only - http://bmjopen.bmj.com/site/about/guidelines.xhtml

a ‘local’ and ‘remote’ site for each participant. We define local sites as being uninjured but within [or, if not accessible, as close as possible to] the same receptive field as the most painful inured tissue [e.g. skin over gastrocnemius in a participant with an ankle fracture]. By contrast, we define remote sites as a distant, accessible, site from the receptive fields in which tissues are injured [e.g. skin over tibialis anterior in a participant without lower limb injury], and on the contralateral side of the body where injured tissue is unilateral. Where possible, remote sites will be a mirror-image of the local site [to allow for comparison of absolute values], but in a trauma population we are aware that this may not always be possible. For all threshold testing modalities, an ascending method of limits design[62] will be used, whereby stimulus intensity will begin at a low level and gradually increase until the participant first perceives pain. Participants will be instructed to push a button or tell the assessor when the sensation has changed from one of the stimulus alone [e.g. just pressure] to a sensation of both the stimulus and pain [e.g. pressure and pain]. Two consecutive assessments will be performed for each modality at each site, and the mean used for further analysis.[63] A minimum of 30-seconds inter-stimulus interval will be given between each threshold measurement within a single session. Measurements will be taken at baseline, while the participant is an inpatient in the hospital; we will aim to collect data as frequently as every 48-hours to gain accurate rate-of-change data, but this will depend upon participant accessibility and assessor availability. To ensure pain thresholds are consistently measured at the same sites every session, sites will be labelled using a sterile, skin marking pen [Schuco Ltd, UK]. Because sites cannot be standardised between participants, the rates-of-change of these values will be used as candidate prognostic variables, to allow for comparisons between participants. The order of testing will be randomised by modality at each session to avoid order effects.

For peer review only - http://bmjopen.bmj.com/site/about/guidelines.xhtml

Thermal [heat and cold] pain thresholds will be measured using skin-contact stimulation, using the same thermode at the same sites, within specified local and remote dermatomes. Thermal pain threshold assessments will be performed by delivering thermal stimuli directly to the skin through a metal 30x30 mm Peltier thermode, using a TSA-II NeuroSensory Analyzer thermal stimulator and accompanying software [Medoc Ltd., Israel]. To evaluate heat pain threshold, temperature will be gradually increased, at a rate of 1°C/s from a 'neutral' baseline of 32°C, to a maximum temperature of 50.5°C to avoid thermal injury.[64] During each measurement, participants will be instructed to press a button when the stimulus becomes painful, and this will be documented as the threshold value. Once pain threshold is achieved [and recorded], the temperature will return to the baseline value at the same rate [1°C/s]. For cold pain threshold measurements, the temperature will be gradually reduced, at a rate of 1°C/s from the baseline of 30°C, to a minimum temperature of 0°C,[64] before also returning to baseline at a rate of 1°C/s.

Pressure pain thresholds will be measured at a local and remote site, using a pressure algometer [Somedic SenseLab AB, Sweden]. Skin and muscle tissue are simultaneously stimulated during pressure threshold testing; sites will therefore be chosen where a dermatome and myotome are likely to share a common nerve root innervation [e.g. skin over tibialis anterior]. The algometer has a circular contact tip of 1cm^2 area.[65] The tip will be applied perpendicular to the skin at a constant rate of pressure increase of 50kPa/s, until the first onset of pain. For each measurement, pressure will be unloaded at the same rate that it was loaded.

To measure excitability of nociceptive pathways in response to mechanical stimuli, a series of repetitive, pressure stimulus 'pulses' will be applied via the algometer, with the aim

For peer review only - http://bmjopen.bmj.com/site/about/guidelines.xhtml

of provoking temporal summation responses.[66-68] The peak pressure reached during each pulse will be the mean pressure pain threshold measured for that particular site, as described previously. A minimum of 2-minutes after all threshold tests have been completed, 10 consecutive pressure pulses will be applied at the remote and local site. For each pulse, pressure will be gradually increased at a rate of $2N/cm^2/s$ to the peak value, and maintained at that value for 1-second, before being released at the same rate. A 1-second inter-stimulus interval will be used between pulses. Pain intensity of the 1st, 5th, and 10th pulses will be rated on a numerical rating scale [0 being 'no pain' to 10 being 'pain as bad as could be']. In the event that participants indicate that pain has become intolerable, the sequence will be stopped immediately, and the NRS score and number of impulses performed at that point will be noted.

Biomarkers

Serum levels of C-reactive protein [CRP] will be used as a biomarker for inflammation. CRP is an acute-phase response protein produced by hepatocytes and is usually found in concentrations of 0.3 to 1.7 mg/l[69]. Increased production is due to cytokine-dependent induction of synthesis and elevated levels may be detected within eight hours of a stimulus and can reach 500 mg/1[70]. Besides trauma,[71] elevated levels of CRP may be seen in conditions such as autoimmune disease, infection and malignancy. It has also been associated with acute sciatica.[72] The level of CRP usually peaks within 48 hours of the stimulus. In contrast, when the stimulus for increased production completely ceases, the circulating CRP concentration falls rapidly, at almost the rate of plasma CRP clearance.[70] A fall in serial measurements usually indicates resolution of the underlying process, while persisting elevated levels indicates ongoing inflammation.[73] Where possible, measurements of serum

For peer review only - http://bmjopen.bmj.com/site/about/guidelines.xhtml

For peer review only

CRP will be repeatedly taken, to include 48-hours following trauma [to obtain the peak value] and every subsequent 48-hours [to measure changes in value], while the participant is an inpatient; absolute and rates of change of CRP values will be used as candidate prognostic factors.[74]

For peer review only

For peer review only - http://bmjopen.bmj.com/site/about/guidelines.xhtml

REFERENCES

1. Stewart AL, Greenfield S, Hays RD, et al. Functional status and well-being of patients with chronic conditions. Results from the Medical Outcomes Study. *JAMA* 1989;262(7):907-13.
2. Ware JE, Jr., Sherbourne CD. The MOS 36-item short-form health survey (SF-36). I. Conceptual framework and item selection. *Med Care* 1992;30(6):473-83.
3. Hays RD, Sherbourne CD, Mazel RM. The RAND 36-Item Health Survey 1.0. *Health Econ* 1993;2(3):217-27.
4. McHorney CA, Ware JE, Jr., Raczek AE. The MOS 36-Item Short-Form Health Survey (SF-36): II. Psychometric and clinical tests of validity in measuring physical and mental health constructs. *Med Care* 1993;31(3):247-63.
5. Revell MP, Pynsent, P.B., Abudu, A., Fairbank, J.C.T. . Trauma Scores and Trauma Outcome Measures *Trauma* 2003;5:61-70.
6. Ware J. SF-36 Health Survey Updated. *Spine* 2000;25(24):3130-39.
7. Dattani R, Slobogean, G.P., O'Brien, P.J., Broekhuyse, H.M., Blachut, P.A., Guy, P., Lefaivre, K.A. Psychometric Analysis of Measuring Functional Outcomes in Tibial Plateau Fractures using the Short Form (SF-36), Short Musculoskeletal Function Assessment (SMFA) and the Western Ontario McMaster Osteoarthritis (WOMAC) Questionnaires. *Injury Int J Care Injured* 2013;44:825-29.
8. Brooks R. EuroQol: the current state of play. *Health Policy* 1996;37(1):53-72.
9. Janssen MF, Birnie E, Haagsma JA, et al. Comparing the standard EQ-5D three-level system with a five-level version. *Value Health* 2008;11(2):275-84. doi: 10.1111/j.1524-4733.2007.00230.x
10. Janssen MF, Pickard AS, Golicki D, et al. Measurement properties of the EQ-5D-5L compared to the EQ-5D-3L across eight patient groups: a multi-country study. *Qual Life Res* 2013;22(7):1717-27. doi: 10.1007/s11136-012-0322-4
11. Mahoney FI, Barthel DW. Functional Evaluation: The Barthel Index. *Md State Med J* 1965;14:61-5.
12. Shah S, Vanclay F, Cooper B. Improving the sensitivity of the Barthel Index for stroke rehabilitation. *J Clin Epidemiol* 1989;42(8):703-9.
13. Hseuh IP, Lee, M.M., Hsieh, C.L. . Psychometric Characteristics of the Barthel Activities of Daily Living Index in Stroke Patients *J Formos Med Assoc* 2001;100(526-532)

For peer review only

14. Hseuh IP, Lin, J.H., Jeng, J.S., Hsieh, C.L. Comparison of the Psychometric Characteristics of the Functional Independence Measure, 5 Item Barthel Index and 10 Item Barthel Index in Patients with Stroke. *J Neurol Neurosurg Psychiatry* 2002;73:188-90.
15. Hsieh YW, Wang, C.H., Wu, S.C., Chen, P.C., Sheu, C.F., Hsieh, C.L. . Establishing the Minimally Clinically Important Difference of the Barthel Index in Stroke Patients. *Neurorehabilitation and Neural Repair* 2007;21(3):233-38.
16. Hobart JC, Thompson AJ. The five item Barthel index. *J Neurol Neurosurg Psychiatry* 2001;71(2):225-30.
17. Eriksen HR, Ihlebaek C, Ursin H. A scoring system for subjective health complaints (SHC). *Scand J Public Health* 1999;27(1):63-72.
18. Zigmond AS, Snaith RP. The hospital anxiety and depression scale. *Acta Psychiatr Scand* 1983;67(6):361-70.
19. Bjelland I, Dahl AA, Haug TT, et al. The validity of the Hospital Anxiety and Depression Scale. An updated literature review. *J Psychosom Res* 2002;52(2):69-77.
20. Wang W, Chair, S.Y., Thompson, D.R., Twinn, S.F. A Psychometric Evaluation of the Chinese Version of the Hospital Anxiety and Depression Scale in Patients with Coronary Heart Disease *Journal of Clinical Nursing* 2009;18:1908-15.
21. Sullivan MJL BS, Pivik J. The Pain Catastrophizing Scale: Development and validation. . *Psychological Assessment* 1995;7(4):524-32.
22. Fernandes L, Storheim, K., Lochting, I., Grotle, M. Cross Cultural Adapation and Validation of the Norweigian Pain Catastrophizing Scale in Patients with Low Back Pain *BMC Musculoskeletal Disorders* 2012;13:111-20.
23. Lopes RA, Dias RC, Queiroz BZ, et al. Psychometric properties of the Brazilian version of the Pain Catastrophizing Scale for acute low back pain. *Arq Neuropsiquiatr* 2015;73(5):436-44. doi: 10.1590/0004-282X20150026
24. Woby SR, Roach NK, Urmston M, et al. Psychometric properties of the TSK-11: a shortened version of the Tampa Scale for Kinesiophobia. *Pain* 2005;117(1-2):137-44. doi: 10.1016/j.pain.2005.05.029
25. Miller RP KS, Todd D. The Tampa Scale: a measure of kinesiophobia. *Clin J Pain* 1991;7(1):51-52.
26. Swinkels-Meewisse EJCM, Swinkels, R.A.H.M., Verbeek, A.L.M., Vlaeyen, J.W.S. . Psychometric Propertieis of the Tampa Scale for Kinesiophobia and the Fear

For peer review only

For peer review only - http://bmjopen.bmj.com/site/about/guidelines.xhtml

Avoidance Beliecs Questionnaire in Acute Low Back Pain *Manual Therapy* 2003;8(1):29-36.

27. Kall LB. Psychological determinants of quality of life in patients with whiplash associated disorders-a prospective study. *Disabil Rehabil* 2009;31(3):227-36. doi: 10.1080/09638280801912030
28. M.K. N. The pain self-efficacy questionnaire: Taking pain into account. *Eur J Pain* 2007;11(2):153-63.
29. Paul Campbell NEF, Elaine Thomas, and Kate M. Dunn. Prognostic Indicators of Low Back Pain in Primary Care: Five-Year Prospective Study. *J Pain* 2013;14(8):873–83.
30. Horowitz M, Wilner N, Alvarez W. Impact of Event Scale: a measure of subjective stress. *Psychosom Med* 1979;41(3):209-18.
31. Beck JG, Grant DM, Read JP, et al. The impact of event scale-revised: psychometric properties in a sample of motor vehicle accident survivors. *J Anxiety Disord* 2008;22(2):187-98. doi: 10.1016/j.janxdis.2007.02.007
32. Creamer M, Bell R, Failla S. Psychometric properties of the Impact of Event Scale - Revised. *Behav Res Ther* 2003;41(12):1489-96.
33. Barbero M, Mores, F., Leoni, D., Gatti, R., Egloff, M., Falla, D. Test-retest reliability of pain extent and pain location using a novel method for pain drawing analysis. *European Journal of Pain* 2015;19:1129-38.
34. Baker SP, O'Neill B. The injury severity score: an update. *J Trauma* 1976;16(11):882-5.
35. Akmal M, Trivedi R, Sutcliffe J. Functional outcome in trauma patients with spinal injury. *Spine (Phila Pa 1976)* 2003;28(2):180-5. doi: 10.1097/01.BRS.0000041577.12233.96
36. Baker SP, O'Neill B, Haddon W, Jr., et al. The injury severity score: a method for describing patients with multiple injuries and evaluating emergency care. *J Trauma* 1974;14(3):187-96.
37. Linn S. The injury severity score--importance and uses. *Ann Epidemiol* 1995;5(6):440-6.
38. Sears JM, Blanar L, Bowman SM. Predicting work-related disability and medical cost outcomes: a comparison of injury severity scoring methods. *Injury* 2014;45(1):16-22. doi: 10.1016/j.injury.2012.12.024
39. NICE. Low back pain and sciatica in over 16s: assessment and management

NICE guideline [NG59]: National Institute for Health and Care Excellence, 2016.

For peer review only - http://bmjopen.bmj.com/site/about/guidelines.xhtml

40. Von Korff M, Ormel J, Keefe FJ, et al. Grading the severity of chronic pain. *Pain* 1992;50(2):133-49.

41. Jensen MP, McFarland CA. Increasing the reliability and validity of pain intensity measurement in chronic pain patients. *Pain* 1993;55(2):195-203.

42. Jensen MP, Turner, J.A. and Romano, J.M. . What is the maximum number of levels needed in pain intensity measurement? . *Pain* 1994;58:387–92.

43. Jensen MP, Turner, L.R., Turner, J.A. and Romano, J.M. The use of multiple-item scales for pain intensity measurement in chronic pain patients. *Pain* 1996;67:35–40.

44. Jensen MP, Turner JA, Romano JM, et al. Comparative reliability and validity of chronic pain intensity measures. *Pain* 1999;83(2):157-62.

45. Von Korff M. Epidemiological and survey methods – assessment of chronic pain. In Turk, D.C. and Melzack, R., editors, Handbook of Pain Assessment. . New York, USA: Guildford Press 2001:603-18.

46. Farrar JT, Young JP, Jr., LaMoreaux L, et al. Clinical importance of changes in chronic pain intensity measured on an 11-point numerical pain rating scale. *Pain* 2001;94(2):149-58.

47. Salaffi F, Stancati A, Silvestri CA, et al. Minimal clinically important changes in chronic musculoskeletal pain intensity measured on a numerical rating scale. *Eur J Pain* 2004;8(4):283-91. doi: 10.1016/j.ejpain.2003.09.004

48. Childs JD, Piva SR, Fritz JM. Responsiveness of the numeric pain rating scale in patients with low back pain. *Spine (Phila Pa 1976)* 2005;30(11):1331-4.

49. Ostelo RW, Deyo, R.A., Stratford, P., Waddell, G., Croft, P., Von Korff, M., Bouter, L.M. and Henrica, C. Interpreting change scores for pain and functional status in low back pain: towards international consensus regarding minimal important change. *Spine* 2008;33(1):90-94.

50. Michener LA, Snyder AR, Leggin BG. Responsiveness of the numeric pain rating scale in patients with shoulder pain and the effect of surgical status. *J Sport Rehabil* 2011;20(1):115-28.

51. Masters Steedman S, Middaugh SJ, Kee WG, et al. Chronic-pain medications: equivalence levels and method of quantifying usage. *Clin J Pain* 1992;8(3):204-14.

52. Harden RN, Weinland SR, Remble TA, et al. Medication Quantification Scale Version III: update in medication classes and revised detriment weights by survey of American Pain Society Physicians. *J Pain* 2005;6(6):364-71. doi: 10.1016/j.jpain.2005.01.350

For peer review only

For peer review only - http://bmjopen.bmj.com/site/about/guidelines.xhtml

53. Gallizzi M GC, Harden RN, Stanos S, Khan A. Medication Quantification Scale Version III: internal validation of detriment weights using a chronic pain population. . *Pain Pract* 2008;8(1):1-4.

54. KJ S, WG K, S S, et al. Medication quantification scores and evaluation of patient pharmacology profiles. *Current Review of Pain* 1998;2(3):171–74.

55. Freynhagen R, Baron R, Gockel U, et al. painDETECT: a new screening questionnaire to identify neuropathic components in patients with back pain. *Curr Med Res Opin* 2006;22(10):1911-20. doi: 10.1185/030079906X132488

56. Cappelleri JC, Koduru V, Bienen EJ, et al. A cross-sectional study examining the psychometric properties of the painDETECT measure in neuropathic pain. *J Pain Res* 2015;8(8):159-67. doi: 10.2147/JPR.S80046

57. Keller T, Freynhagen R, Tolle TR, et al. A retrospective analysis of the long-term test-retest stability of pain descriptors of the painDETECT questionnaire. *Curr Med Res Opin* 2016;32(2):343-9. doi: 10.1185/03007995.2015.1125869

58. Tampin B, Bohne T, Callan M, et al. Reliability of the English version of the painDETECT questionnaire. *Curr Med Res Opin* 2017;33(4):741-48. doi: 10.1080/03007995.2017.1278682

59. Sadosky A, Koduru V, Bienen EJ, et al. Characterizing individual painDETECT symptoms by average pain severity. *Clinicoecon Outcomes Res* 2016;8:361-6. doi: 10.2147/CEOR.S105402

60. Cappelleri JC, Koduru V, Bienen EJ, et al. Mapping painDETECT, a neuropathic pain screening tool, to the EuroQol (EQ-5D-3L). *Qual Life Res* 2017;26(2):467-77. doi: 10.1007/s11136-016-1379-2

61. M OB. Aids to the Examination of the Peripheral Nervous System: Saunders (Elsevier) 2010.

62. T. Palmer DJM, Wilma M. Steedman, John Ravey, S. C-and Aδ-fibre mediated thermal perception: response to rate of temperature change using method of limits. . *Somatosensory & motor research* 2000;17(4):325-33.

63. Chesterton LS SJ, Wright CC, Foster NE. Interrater reliability of algometry in measuring pressure pain thresholds in healthy humans, using multiple raters. *Clin J Pain* 2007;23(9):760-6.

64. Knutti IA SM, Opsommer E. Test-retest reliability of thermal quantitative sensory testing on two sites within the L5 dermatome of the lumbar spine and lower extremity. *Neurosci Lett* 2014;5(579):157-62.

For peer review only - http://bmjopen.bmj.com/site/about/guidelines.xhtml

65. Greenspan JD, McGillis SL. Stimulus features relevant to the perception of sharpness and mechanically evoked cutaneous pain. *Somatosens Mot Res* 1991;8(2):137-47.

66. Sarlani E, Greenspan JD. Gender differences in temporal summation of mechanically evoked pain. *Pain* 2002;97(1-2):163-9.

67. Nie H, Arendt-Nielsen L, Andersen H, et al. Temporal summation of pain evoked by mechanical stimulation in deep and superficial tissue. *J Pain* 2005;6(6):348-55. doi: 10.1016/j.jpain.2005.01.352

68. Nie H, Arendt-Nielsen L, Madeleine P, et al. Enhanced temporal summation of pressure pain in the trapezius muscle after delayed onset muscle soreness. *Exp Brain Res* 2006;170(2):182-90. doi: 10.1007/s00221-005-0196-6

69. Whicher JT ES. Acute phase proteins. *Hosp Update* 1990:899-905.

70. Pepys MB, Hirschfield GM. C-reactive protein: a critical update. *J Clin Invest* 2003;111(12):1805-12. doi: 10.1172/JCI18921

71. Gosling P, Dickson GR. Serum c-reactive protein in patients with serious trauma. *Injury* 1992;23(7):483-6.

72. Sturmer T, Raum E, Buchner M, et al. Pain and high sensitivity C reactive protein in patients with chronic low back pain and acute sciatic pain. *Ann Rheum Dis* 2005;64(6):921-5. doi: 10.1136/ard.2004.027045

73. Giannoudis PV HF, Pape HC. . Inflammatory serum markers in patients with multiple trauma. Can they predict outcome? *J Bone Joint Surg Br* 2004;86(3):313-23.

74. Barma M, Goodbrand, J.A., Donnan, P.T., McGilchrist, M.M., Frost, H., McMurdo, M.E. and Witham, M.D. Slower Decline in C-Reactive Protein after an Inflammatory Insult Is Associated with Longer Survival in Older Hospitalised Patients. . *PloS one* 2016;11(7):e0159412.

For peer review only

For peer review only - http://bmjopen.bmj.com/site/about/guidelines.xhtml

STANDARD PROTOCOL ITEMS: RECOMMENDATIONS FOR INTERVENTIONAL TRIALS

SPIRIT 2013 Checklist: Recommended items to address in a clinical trial protocol and related documents*

Section/item	Item No	Description
**Administrative information**		
Title	1	Descriptive title identifying the study design, population, interventions, and, if applicable, trial acronym – Page 1
Trial registration	2a	Trial identifier and registry name. If not yet registered, name of intended registry – N/A
	2b	All items from the World Health Organization Trial Registration Data Set – N/A
Protocol version	3	Date and version identifier – Page 1
Funding	4	Sources and types of financial, material, and other support – Page 25
Roles and responsibilities	5a	Names, affiliations, and roles of protocol contributors – Pages 1, 25
	5b	Name and contact information for the trial sponsor – Page 25
	5c	Role of study sponsor and funders, if any, in study design; collection, management, analysis, and interpretation of data; writing of the report; and the decision to submit the report for publication, including whether they will have ultimate authority over any of these activities – Page 25
	5d	Composition, roles, and responsibilities of the coordinating centre, steering committee, endpoint adjudication committee, data management team, and other individuals or groups overseeing the trial, if applicable (see Item 21a for data monitoring committee) – Page 25
**Introduction**		
Background and rationale	6a	Description of research question and justification for undertaking the trial, including summary of relevant studies (published and unpublished) examining benefits and harms for each intervention – Page 5 (introduction, page 6 (aims and objectives)
	6b	Explanation for choice of comparators – Supplementary file
Objectives	7	Specific objectives or hypotheses (Page 6)

For peer review only

Trial design	8	Description of trial design including type of trial (eg, parallel group, crossover, factorial, single group), allocation ratio, and framework (eg, superiority, equivalence, noninferiority, exploratory) – Page 7

Methods: Participants, interventions, and outcomes

Study setting	9	Description of study settings (eg, community clinic, academic hospital) and list of countries where data will be collected. Reference to where list of study sites can be obtained – Page 7
Eligibility criteria	10	Inclusion and exclusion criteria for participants. If applicable, eligibility criteria for study centres and individuals who will perform the interventions (eg, surgeons, psychotherapists) – Pages 8-10
Interventions	11a	Interventions for each group with sufficient detail to allow replication, including how and when they will be administered – N/A
	11b	Criteria for discontinuing or modifying allocated interventions for a given trial participant (eg, drug dose change in response to harms, participant request, or improving/worsening disease) – N/A
	11c	Strategies to improve adherence to intervention protocols, and any procedures for monitoring adherence (eg, drug tablet return, laboratory tests) – N/A
	11d	Relevant concomitant care and interventions that are permitted or prohibited during the trial – N/A
Outcomes	12	Primary, secondary, and other outcomes, including the specific measurement variable (eg, systolic blood pressure), analysis metric (eg, change from baseline, final value, time to event), method of aggregation (eg, median, proportion), and time point for each outcome. Explanation of the clinical relevance of chosen efficacy and harm outcomes is strongly recommended – Page 10 (primary outcome), Supplementary file (candidate predictors)
Participant timeline	13	Time schedule of enrolment, interventions (including any run-ins and washouts), assessments, and visits for participants. A schematic diagram is highly recommended (see Figure) – Pages 8-10
Sample size	14	Estimated number of participants needed to achieve study objectives and how it was determined, including clinical and statistical assumptions supporting any sample size calculations – Pages 14-15
Recruitment	15	Strategies for achieving adequate participant enrolment to reach target sample size – Pages 14-15

Methods: Assignment of interventions (for controlled trials)

Allocation:

Sequence generation	16a	Method of generating the allocation sequence (eg, computer-generated random numbers), and list of any factors for stratification. To reduce predictability of a random sequence, details of any planned restriction (eg, blocking) should be provided in a separate document that is unavailable to those who enrol participants or assign interventions – N/A
Allocation concealment mechanism	16b	Mechanism of implementing the allocation sequence (eg, central telephone; sequentially numbered, opaque, sealed envelopes), describing any steps to conceal the sequence until interventions are assigned – N/A
Implementation	16c	Who will generate the allocation sequence, who will enrol participants, and who will assign participants to interventions – N/A
Blinding (masking)	17a	Who will be blinded after assignment to interventions (eg, trial participants, care providers, outcome assessors, data analysts), and how N/A
	17b	If blinded, circumstances under which unblinding is permissible, and procedure for revealing a participant's allocated intervention during the trial – N/A
**Methods: Data collection, management, and analysis**		
Data collection methods	18a	Plans for assessment and collection of outcome, baseline, and other trial data, including any related processes to promote data quality (eg, duplicate measurements, training of assessors) and a description of study instruments (eg, questionnaires, laboratory tests) along with their reliability and validity, if known. Reference to where data collection forms can be found, if not in the protocol – Pages 10-14, Supplementary file
	18b	Plans to promote participant retention and complete follow-up, including list of any outcome data to be collected for participants who discontinue or deviate from intervention protocols – Page 9 (withdrawals)
Data management	19	Plans for data entry, coding, security, and storage, including any related processes to promote data quality (eg, double data entry; range checks for data values). Reference to where details of data management procedures can be found, if not in the protocol – Page 14
Statistical methods	20a	Statistical methods for analysing primary and secondary outcomes. Reference to where other details of the statistical analysis plan can be found, if not in the protocol – Page 15-16
	20b	Methods for any additional analyses (eg, subgroup and adjusted analyses) – Page 16 (screening tool development and validation)

	20c	Definition of analysis population relating to protocol non-adherence (eg, as randomised analysis), and any statistical methods to handle missing data (eg, multiple imputation) – Page 15 (imputation)
**Methods: Monitoring**		
Data monitoring	21a	Composition of data monitoring committee (DMC); summary of its role and reporting structure; statement of whether it is independent from the sponsor and competing interests; and reference to where further details about its charter can be found, if not in the protocol. Alternatively, an explanation of why a DMC is not needed – N/A
	21b	Description of any interim analyses and stopping guidelines, including who will have access to these interim results and make the final decision to terminate the trial – N/A
Harms	22	Plans for collecting, assessing, reporting, and managing solicited and spontaneously reported adverse events and other unintended effects of trial interventions or trial conduct – N/A
Auditing	23	Frequency and procedures for auditing trial conduct, if any, and whether the process will be independent from investigators and the sponsor – N/A
**Ethics and dissemination**		
Research ethics approval	24	Plans for seeking research ethics committee/institutional review board (REC/IRB) approval – Pages 19-20
Protocol amendments	25	Plans for communicating important protocol modifications (eg, changes to eligibility criteria, outcomes, analyses) to relevant parties (eg, investigators, REC/IRBs, trial participants, trial registries, journals, regulators) – N/A
Consent or assent	26a	Who will obtain informed consent or assent from potential trial participants or authorised surrogates, and how (see Item 32) – Pages 9-10
	26b	Additional consent provisions for collection and use of participant data and biological specimens in ancillary studies, if applicable – N/A
Confidentiality	27	How personal information about potential and enrolled participants will be collected, shared, and maintained in order to protect confidentiality before, during, and after the trial – Page 14
Declaration of interests	28	Financial and other competing interests for principal investigators for the overall trial and each study site – Page 25
Access to data	29	Statement of who will have access to the final trial dataset, and disclosure of contractual agreements that limit such access for investigators – Not present

For peer review only

Ancillary and post-trial care	30	Provisions, if any, for ancillary and post-trial care, and for compensation to those who suffer harm from trial participation – N/A
Dissemination policy	31a	Plans for investigators and sponsor to communicate trial results to participants, healthcare professionals, the public, and other relevant groups (eg, via publication, reporting in results databases, or other data sharing arrangements), including any publication restrictions – N/A
	31b	Authorship eligibility guidelines and any intended use of professional writers – N/A
	31c	Plans, if any, for granting public access to the full protocol, participant-level dataset, and statistical code – N/A
**Appendices**		
Informed consent materials	32	Model consent form and other related documentation given to participants and authorised surrogates – N/A
Biological specimens	33	Plans for collection, laboratory evaluation, and storage of biological specimens for genetic or molecular analysis in the current trial and for future use in ancillary studies, if applicable – N/A

*It is strongly recommended that this checklist be read in conjunction with the SPIRIT 2013 Explanation & Elaboration for important clarification on the items. Amendments to the protocol should be tracked and dated. The SPIRIT checklist is copyrighted by the SPIRIT Group under the Creative Commons "Attribution-NonCommercial-NoDerivs 3.0 Unported" license.

For peer review only

BMJ Open

DEVELOPMENT OF A SCREENING TOOL TO PREDICT THE RISK OF CHRONIC PAIN AND DISABILITY FOLLOWING MUSCULOSKELETAL TRAUMA: PROTOCOL FOR A PROSPECTIVE OBSERVATIONAL STUDY

Journal:	*BMJ Open*
Manuscript ID	bmjopen-2017-017876.R1
Article Type:	Protocol
Date Submitted by the Author:	19-Oct-2017
Complete List of Authors:	Rushton, Alison; University of Birmingham, School of Sport, Exercise and Rehabilitation Sciences<br>Evans, David; University of Birmingham, School of Sport, Exercise and Rehabilitation Sciences<br>Middlebrook, Nicola; University of Birmingham, School of Sport, Exercise and Rehabilitation Sciences<br>Heneghan, Nicola; University of Birmingham, School of Sport, Exercise and Rehabilitation Sciences<br>Small, Charlotte; University of Birmingham, NIHR Surgical Reconstruction & Microbiology Research Centre<br>Lord, Janet; University of Birmingham, NIHR Surgical Reconstruction & Microbiology Research Centre<br>Patel, Jaimin; University of Birmingham, NIHR Surgical Reconstruction & Microbiology Research Centre<br>Falla, Deborah; University of Birmingham, School of Sport, Exercise and Rehabilitation Sciences
**Primary Subject Heading**:	Rehabilitation medicine
Secondary Subject Heading:	Medical management
Keywords:	Musculoskeletal trauma, Precision rehabilitation, Pain mechanisms

SCHOLARONE™ Manuscripts

TITLE PAGE

DEVELOPMENT OF A SCREENING TOOL TO PREDICT THE RISK OF CHRONIC PAIN AND DISABILITY FOLLOWING MUSCULOSKELETAL TRAUMA: PROTOCOL FOR A PROSPECTIVE OBSERVATIONAL STUDY

Rushton A[1,2], Evans DW[1,2], Middlebrook N[1,2], Heneghan N[1], Small C[2], Lord J[2], Patel JM[2], Falla D[1,2].

Affiliations

[1] Centre of Precision Rehabilitation for Spinal Pain
School of Sport, Exercise and Rehabilitation Sciences
College of Life and Environmental Sciences
University of Birmingham
Edgbaston
Birmingham
B15 2TT
United Kingdom

[2] NIHR Surgical Reconstruction & Microbiology Research Centre
University of Birmingham
Edgbaston
Birmingham
B15 2TT
United Kingdom

Correspondence:

Professor Deborah Falla
Centre of Precision Rehabilitation for Spinal Pain
School of Sport, Exercise and Rehabilitation Sciences,
College of Life and Environmental Sciences,
University of Birmingham,
Birmingham, B15 2TT, UK.
Tel: +44 121 415 4220
Email: d.falla@bham.ac.uk

For peer review only

For peer review only - http://bmjopen.bmj.com/site/about/guidelines.xhtml

ABSTRACT

Introduction

Pain is an expected and appropriate experience following traumatic musculoskeletal injury. By contrast, chronic pain and disability are unhelpful yet common sequelae of trauma-related injuries. Presently, the mechanisms that underlie the transition from acute to chronic disabling post-traumatic pain are not fully understood. Such knowledge would facilitate the development and implementation of precision rehabilitation approaches that match interventions to projected risk of recovery, with the aim of preventing poor long-term outcomes. The aim of this study is to identify a set of predictive factors to identify patients at risk of developing ongoing post-traumatic pain and disability following acute musculoskeletal trauma. To achieve this, we will use a unique and comprehensive combination of patient-reported outcome measures, psychophysical testing and biomarkers.

Methods/analysis

A prospective observational study will recruit two temporally staggered cohorts [n=250 each cohort; at least 10 cases per candidate predictor] of consecutive acute musculoskeletal trauma patients aged ≥16 years, who are emergency admissions into a Major Trauma Centre in the United Kingdom, with an episode inception defined as the traumatic event. The first cohort will identify candidate predictors to develop a screening tool to predict development of chronic and disabling pain, and the second will allow evaluation of the predictive performance of the tool [validation]. The outcome being predicted is an individual's absolute risk of poor outcome measured at 6 months follow-up using the Chronic Pain Grade Scale [poor outcome ≥Grade II]. Candidate predictors encompass the four primary mechanisms of pain: *nociceptive* [e.g. injury location], *neuropathic* [e.g. painDETECT], *inflammatory* [biomarkers], and *central hypersensitivity* [e.g. quantitative sensory testing]. Concurrently,

For peer review only - http://bmjopen.bmj.com/site/about/guidelines.xhtml

patient-reported outcome measures will assess general health and psychosocial factors [e.g. pain self-efficacy]. Risk of poor outcome will be calculated using multiple variable regression analysis.

Ethics and dissemination

NHS Research Ethics Committee and institutional R&D approval are in progress.

Keywords

Keywords: Pain mechanisms, Prediction, Precision rehabilitation, Musculoskeletal trauma

Funding: NIHR SRMRC

Word count

2412 [excluding tables and references]

For peer review only - http://bmjopen.bmj.com/site/about/guidelines.xhtml

STRENGTHS AND LIMITATIONS OF THIS STUDY

- A comprehensive array of candidate predictive factors will allow for the prediction of chronic and disabling pain following trauma
- These predictive factors will enable the development and validation of a predictive tool to predict good and poor outcome at 6 months post-injury
- The prospective design of the study enables control of unwarranted influences, and enables a stronger case for inferring causal relationships
- Identifying predictive factors related to poor outcome of pain and disability outcome will facilitate targeting of effective interventions
- Other candidate predictors may have been useful to include [e.g. vibration thresholds], but consideration of burden to participants of testing and sample size considerations necessitated prioritisation of candidate predictive factors.

For peer review only

For peer review only - http://bmjopen.bmj.com/site/about/guidelines.xhtml

INTRODUCTION

Pain is an expected and appropriate experience that usually follows traumatic injury.[1] By contrast, chronic pain and disability are unhelpful but common sequelae of trauma-related injuries.[2] Gaining an understanding of why some people develop chronic and disabling post-traumatic pain is therefore a priority for individual patients, the military and society at large. Notwithstanding, the mechanisms that underlie the transition from acute to chronic disabling post-traumatic pain are not fully understood. Such knowledge would facilitate the development and implementation of a clinical pathway of care that matches interventions to projected risk of poor recovery, with the aim of preventing poor long-term outcomes. This project stems from advances in knowledge relating to the assessment and management of pain[3] and the quantification of potential predictive factors to inform personalised rehabilitation; identifying which patients to target with rehabilitation and when and how to target them.

Few studies have specifically explored predictive factors for recovery, whether poor or good, following physical trauma. Of those that have, psychosocial variables such as anxiety, depression and post-traumatic stress, have so far been identified as the strongest predictors of outcome.[4-7] However, only a limited number of variables have hitherto been evaluated as potential predictive factors. Indeed, current opinion regarding pain mechanisms[8] suggests that the development of chronic pain and disability cannot be entirely attributable to psychosocial factors. This is consistent with research in primary care that has identified predictive factors for poor outcome across a range of musculoskeletal pain conditions[9], which include: widespread pain, high functional disability, high pain intensity, long pain duration,

For peer review only - http://bmjopen.bmj.com/site/about/guidelines.xhtml

high depression/anxiety, presence of previous pain episodes, movement restriction, and poor coping strategies. Moreover, developments in the mechanistic understanding of pain[10-12] suggest that other measures [e.g. indicators of central sensitisation, inflammatory activity] may have potential predictive utility, especially in an acute trauma population.

Aims of study

Using a unique combination of: 1) general patient characteristics including premorbid neuropsychological status, 2) quality of life and physical functioning, 3) psychosocial features, 4) injury characteristics, 5) pain characteristics, 6) quantitative sensory testing, and 7) biomarkers; we aim to find a set of predictive factors to identify patients at risk of developing ongoing post-traumatic pain and disability following acute musculoskeletal trauma. This will subsequently inform the feasibility of developing and evaluating a new clinical care pathway of precision rehabilitation that matches interventions to the predicted risk of poor recovery.

Objectives

1) Identify predictive factors for poor outcome [chronic pain and disability at 6-months post-injury] following acute musculoskeletal trauma.
2) Develop a predictive model to inform a screening tool to identify the predicted risk of poor recovery [transition from acute post-traumatic pain to chronic pain and disability].
3) Estimate the predictive performance of the screening tool through validation of the model in a separate data set.

For peer review only - http://bmjopen.bmj.com/site/about/guidelines.xhtml

4) Document the clinical course of symptoms at 3 and 12 months following acute musculoskeletal trauma.

METHODS AND ANALYSIS

Source of data

The study will be a prospective, observational study of two temporally staggered cohorts of trauma patients, who are emergency department admissions into a Major Trauma Centre in the United Kingdom, with an episode inception defined as the traumatic event [Figure 1]. The first cohort will facilitate development of the prediction model to inform the screening tool, and the second will enable validation of the prediction model through evaluation of the predictive performance of the model and tool.[13 14] There will be an interval of at least 6 months between recruitment into the two respective cohorts. The prospective design enables control of unwarranted influences, and enables a stronger case for inferring causal relationships. The nature of the study necessitates predictive statistical modelling.[15] This protocol is written in line with the TRIPOD statement,[16] in which recommendations are given for the reporting of prediction model development and validation.

Self-reported and physical assessment predictive data will be collected at baseline over a period of up to 14 days [or duration of inpatient stay if shorter], which will commence immediately following recruitment. Biomarker data collection will occur throughout the same baseline period, but can commence prior to recruitment providing assent is gained from a legal consultee. The outcome data will be collected at 6-months post-injury [working definition of chronic pain];[17] ; the point of evaluation of an individual's absolute risk of poor

For peer review only - http://bmjopen.bmj.com/site/about/guidelines.xhtml

outcome [objectives 1, 2 and 3]. In addition, selected data will be measured at 3 and 12-months post-injury to explore the clinical course of recovery following injury in the shorter and longer term [objective 4].

For peer review only

For peer review only - http://bmjopen.bmj.com/site/about/guidelines.xhtml

Figure 1. Study design

[Insert Figure 1 here].

Participants

Participants will be recruited from the register of a Major Trauma Centre in the United Kingdom for a period of up to 24 months [planned start date January 2018]. All consecutive eligible patients will be approached for recruitment until the sample size is achieved.

Eligibility criteria

Inclusion criteria: Adult patients aged ≥16 years who are emergency department admissions into the Major Trauma Centre, with their main criteria for admission being acute musculoskeletal trauma within the preceding 14 days, and a capacity to use and understand written and verbal English language and a mental capacity to provide informed consent [e.g. no confusion, delirium, severe cognitive impairment, or severe mental illness, defined by a score of 6 or less on the Abbreviated Mental Test][20]. The primary reason for including patients with trauma occurring up to 14 days previously is to be inclusive of patients who are critically ill and/or with diminished mental capacity initially following their trauma, and patients requiring surgery as a result of the trauma; representative of the broad trauma population.

For peer review only - http://bmjopen.bmj.com/site/about/guidelines.xhtml

Exclusion criteria: Exclusions will be made where the patient has an acute intra-cranial lesion [e.g. bleed] combined with a score of ≤14 on the Glasgow Coma Scale [18] [a 15-item measure of consciousness impairment with adequate reliability[19] that is routinely taken in trauma patients at hospital admission], where there is evident brain or central nervous system injury or impairment, long-term neuro-cognitive disorders [such as brain tumour, multiple sclerosis, Alzheimer's and Parkinson's diseases, etc.], comorbid cancer, the presence of an ongoing rheumatological condition, prolonged use of corticosteroids, or terminal illness with short life expectancy.

Withdrawals: Participants will be informed that they are free to withdraw from the study at any time, without needing to provide reason. In the event of death within 3 months of being recruited, participants will be automatically withdrawn from the study and the primary prognostic analysis. Baseline data of all withdrawn participants will be kept and compared to those of retained participants to assess for any differences.

Recruitment

Based on feasibility data [site data from the Trauma Audit and Research Network], it is estimated that at least 1,000 eligible trauma patients will be approachable for recruitment over a 24-month period, and that 50% would be expected to consent to participation. A dedicated team of research nurses will be available to recruit patients 7 days per week [from 0700 to 1930].

Because of impairments resulting from their injuries, some otherwise eligible patients will lack the mental capacity to provide informed consent when first approached to enrol in

For peer review only - http://bmjopen.bmj.com/site/about/guidelines.xhtml

the study. Recruitment into the study will therefore be undertaken under the guidance and provision of the [UK] Mental Capacity Act 2005 for research in emergency situations. If the patient lacks sufficient capacity to consent, written assent for study participation will be sought from a legal consultee to begin biomarker data collection [blood samples], with informed consent for full recruitment [and subsequent data collection] being sought from the patient only if, and when, they regain sufficient capacity to provide this. If the patient does not regain capacity to provide consent within 14 days of their trauma, they will not be recruited into the study, biomarker data collection will cease, and any blood samples already collected will be destroyed before analysis.

Once informed consent is gained and the participant recruited, following a minimum 1 hour lead time after the informed consent process [to reduce patient burden], members of the research team will visit the patient at their bedside to collect baseline self-reported data via questionnaires [Table 1]. On the next available working day following completion of the questionnaires [again, to reduce patient burden], members of the study team will return to the patient to conduct the first physical [quantitative sensory testing] assessment. At each visit, if deemed necessary the capacity of the participant will be checked using an Abbreviated Mental Test[20] [a score of 6 or lower is indicative of reduced capacity], and asked if they are happy to proceed with data collection.

Outcome

The outcome for the prediction model is an individual's absolute risk of poor outcome [chronic pain and disability] at 6 months post-injury. Outcome will be measured using the Chronic Pain Grade Scale [CPGS],[21] which combines pain intensity and pain-related

For peer review only - http://bmjopen.bmj.com/site/about/guidelines.xhtml

disability over the preceding 6-months into a single measure of pain *severity*. The CPGS has previously been used to assess the severity of body-wide chronic pain in general population,[22] primary care[23] and post-trauma[24] populations. Each item of the CPGS relates to at least one of the three categories of the International Classification of Functioning, Disability and Health [ICF] [25]: impairment, activity limitations and restricted participation. Furthermore, all ICF categories are encompassed by the CPGS.[26] The CPGS is a unidimensional scale, with good internal consistency across different pain populations; Cronbach's alpha of 0.84 to 0.91 in back pain, 0.79 for headache, and 0.84 for temporomandibular pain.[21 27] With regards to construct validity, cross-sectional and longitudinal studies of general practice patients have shown high scores on the CPGS, indicating greater chronic pain, to be associated with higher rates of unemployment, greater pain impact scale scores, greater use of opioid analgesics and physician visits, depressed mood, and lower self-rated health status.[21 27 28] For convergent validity, the CPGS has been found to have good correlation with equivalent dimensions of the SF-36.[27 28] In terms of responsiveness, changes in score over time in patients with chronic musculoskeletal pain correlated significantly with changes in SF-36 scores.[29] The CPGS has also been shown to have good test-retest reliability in primary care patients with back pain [weighted kappa 0.81, 95% CI 0.65, 0.98].[27]

Although pain persistence is not used in assigning pain grade, a measure of pain days in the prior 6-months is included in the CPGS.[30] The responses on the remaining 7-items are used for computing scores for the 3 subscales of the CPGS:[21] characteristic pain intensity, disability score, and disability points. The characteristic pain intensity score [range: 0-100] is obtained by calculating the mean of 3 pain intensity measurements: 'at the present time', the 'worst pain' in the preceding 6 months, and the 'average' pain over the preceding 6 months. The disability score [range: 0-100] is obtained through the mean ratings of how much the pain has interfered in performing activities of daily living, recreational, social and family

For peer review only - http://bmjopen.bmj.com/site/about/guidelines.xhtml

activities, and work [including housework] activities in the last 6-months. The disability points are scored 0-3 and are derived from a combination of ranked categories of the number of disability days [the number of days that the respondent was away from usual activities in the preceding 6 months due to pain] and disability score. Based on these scores, the participant's chronic pain and disability status can then be classified into one of the 5 ordinal categories of chronic pain severity:[21] no pain [Grade 0], low disability and low intensity pain [Grade I], low disability and high intensity pain [Grade II], high disability and moderately limiting intensity pain [Grade III], and high disability and severely limiting intensity pain [Grade IV]. As in previous studies, poor outcome will be defined as Grade ≥II.[23 31-34]

Candidate predictors

Candidate predictors have been selected that are: [1] reliable and valid measures of their domain, and [2] have a theoretical association with the development of chronic pain. Both modifiable and non-modifiable candidate predictors are included. Candidate predictors are summarised in Table 1, with further detail in Supplementary file S1. Table 1 also details important data that will be measured at 3, 6 and 12-months post-injury to explore the clinical course of recovery following injury in the shorter and longer term. All data collection will be standardised through protocols and clinical report forms.

Table 1: Summary of data collection at different assessment points

**Domain / Candidate predictor**	**Measure / data item**	**Baseline** *Commencing ≤14 days post-trauma*	**3 months** *Clinical course*	**6 months** *Outcome assessment point / clinical course*	**12 months** *Clinical course*
**General patient characteristics including premorbid neuropsychological status**					
Age	In years	√			
Gender	Female / male / other	√			
Body Mass Index	Calculated from height and	√			

For peer review only - http://bmjopen.bmj.com/site/about/guidelines.xhtml

[BMI]	weight measurements				
Education	Highest educational level attained	√			
Employment status	Full-time/ part-time / not working / retired / student Employed / self-employed	√	√	√	√
Circumstance of trauma	Military / civilian	√			
Previous history of musculoskeletal pain and injury	Patient history data from patient recollection and medical records	√			
Comorbidity of other health problems	Patient history data from patient recollection and medical records	√			
Premorbid physical health	Patient history data from patient recollection and medical records	√			
Premorbid psychological health	Patient history data from patient recollection and medical records	√			
Number of days in hospital	Intensive care / ward / total	√			
**Quality of life and physical functioning**					
General health	36-item Short Form Health Survey, version 2 [SF-36v2][35]	√	√	√	√
Health-related quality of life	EuroQol EQ-5D-5L[36]	√	√	√	√
Self-care and mobility during activities of daily living	Barthel Index of Activities of Daily Living,[37] collected from hospital data	√			
Sleep quality	Subjective Health Complaints Inventory	√	√	√	√
Brain/CNS impairment	Glasgow Coma Scale [18]	√			
**Psychosocial features**					
Anxiety and depression	Hospital Anxiety and Depression Scales [HADS][38]	√	√	√	√
Coping strategies applied during a painful experience	Coping Strategies Questionnaire-24 [CSQ-24][39]	√	√	√	√
Fear of movement or fear of injury or re-injury during movement	Tampa Scale of Kinesiophobia, 11-item [TSK-11][40]	√	√	√	√
Confidence in ability to perform activities despite pain	Pain Self-Efficacy Questionnaire[41]	√	√	√	√
Subjective post-traumatic distress	Impact of Event Scale revised [IES-R][42]	√	√	√	√
**Injury characteristics**					
Time of injury/incident	Hospital record data	√			
Injury location	Adapted pain drawings, based on hospital record data	√			
Tissues damaged	Based on imaging data and hospital records Fractures Penetrating / non-penetrating injury / both	√			
Surgery required	Location and post-injury timing of surgery, based on hospital	√			

For peer review only

For peer review only - http://bmjopen.bmj.com/site/about/guidelines.xhtml

	record data				
Assisted mechanical ventilation required	Yes / no / duration	√			
Severity of injury	Injury Severity Scale[43]	√			
**Pain characteristics**					
Chronic pain severity	Chronic Pain Grade Scale[21]			√	√
Pain intensity	11-point [0-10] Numerical Rating Scale, relating to current pain, from 'no pain' to 'pain as bad as could be' [collected as part of the Chronic Pain Grade Scale]	√[1]	√	√	√
Pain medication intake [type, dosage and timing]	Medication Quantification Scale,[44-46] based on hospital record data	√[1]			
Pain location	Pain drawing	√[1]	√	√	√
Pain extent	Electronic pain drawing[47]	√[1]			
Self-reported features of neuropathic pain	painDETECT questionnaire[48]	√[1]	√	√	√
**Quantitative sensory testing**					
Heat pain threshold	Evaluation of pain threshold using a heat stimulus	√[1]			
Cold pain threshold	Evaluation of pain threshold using a cold stimulus	√[1]			
Pressure pain threshold	Evaluation of pain threshold using a pressure stimulus	√[1]			
Temporal summation	Evaluation of pain intensity in response to repetitive pressure stimuli	√[1]			
**Biomarkers**					
C-reactive protein [CRP]	Serum levels of CRP, a broad indicator of inflammation [via blood analysis]	√[2]			
Mitochondrial DNA [mtDNA]	Serum levels of mitochondrial DNA, an indicator of tissue damage [via blood analysis]	√[2]			

[1] Measurements to be taken repeatedly throughout the baseline period, which will commence immediately following recruitment via informed consent [up to 14 days post-trauma] for a period of up to 14 days [i.e. until a maximum of 28 days post-trauma], whilst the participant is in hospital
[2] Measurements to be taken repeatedly throughout the baseline period, but may be commenced prior to this, subject to assent from a legal consultee

Data handling

Blood samples will be collected through the clinical and research nurse teams, whilst the participant is in the hospital, and either analysed immediately [C-reactive protein] or securely stored for subsequent analysis [mitochondrial DNA]. Baseline self-reported questionnaires, pain and injury drawings, and physical assessments will be collected by one

For peer review only - http://bmjopen.bmj.com/site/about/guidelines.xhtml

of three trained assessors from the study team. Inter-rater reliability studies [across 2 assessors] will first be conducted in both healthy and trauma populations to inform definitive testing protocols. The order of physical assessment data collection will be randomly assigned [using computerised randomisation software] according to the modality of testing and by site, to prevent order effects. Follow-up self-reported questionnaires will be posted to participants at their home addresses; with up to two reminders sent for non-response. All questionnaires will be formatted so that data can be scanned or entered directly into an electronic database. Following data entry, data will be checked by a second researcher for completeness and accuracy. In addition, regular audits of data collection and storage will be performed by an independent study steering committee. All outcome measure data will be collated into an anonymised database and stored securely for a period of 10-years at the University of Birmingham, in line with Research Governance procedures. Participants will receive usual care, and interventions received will be recorded for descriptive analysis. Data will be analysed using IBM SPSS Statistics. Participant identifiable information will be stored on an encrypted electronic database file on physically and electronically secure servers within the hospital, in line with current United Kingdom data protection legislation, and only accessible by the Trust Principal Investigator who will not be involved in data analysis.

Sample size

In predictive modelling, a larger sample size enables lower bias and variance, and permits the prospective prediction of new observations.[15] The number of predictors will be reduced using exploratory factor analysis. This process will ensure that the sample size provides at least 10 cases per candidate predictor, to adequately power the final regression analysis.[49 50] Data will be collected for an estimated 300 participants per cohort [n=600 total]

For peer review only - http://bmjopen.bmj.com/site/about/guidelines.xhtml

to allow for withdrawals [primarily expected deaths within the first 3 months] and losses to follow-up, so that final data are available for 250 participants per cohort [n=500 total].

Statistical analysis methods and management of missing data

For each cohort, potentially eligible patients, numbers examined for eligibility, confirmed eligible, recruited into the study, completing follow-up, and analysed will be reported in a flow diagram. Reasons for non-participation, exclusion, drop-outs and withdrawal [e.g. death] will be reported at each stage. Participant characteristics will be descriptively presented. For each variable of interest, the number of participants with missing data will be reported.

For the first cohort to develop the predictive model, an initial exploratory data analysis stage will summarise the data.[15] Correlations between candidate prognostic factors will be calculated at baseline. Outcome [CPGS] scores will be dichotomised into good and poor categories as described previously. Data reduction will use exploratory factor analysis to assess factor loading of candidate predictors [summary scores] on poor outcome at 6 months. This will enable the number of candidate predictors entered into the final model to be reduced to 25, which can be supported by the cohort sample of 250. This process reduces the risk of over-fitting the model and the risk of selecting the wrong variables due to correlation between predictor variables [multicollinearity].[51]

Statistical modelling for prediction has been planned *a priori*. To explore the influence of each prognostic factor on poor outcome at 6 months, a logistic multivariable regression model will be fitted to the dichotomised outcome scores to calculate low and high

For peer review only - http://bmjopen.bmj.com/site/about/guidelines.xhtml

risk of poor outcome. Odds ratios for each candidate prognostic factor will be reported, adjusted for other factors and account for clustering [e.g. level of injury severity]. If necessary, multiple imputation[52] will be used to deal with missing outcome data. The characteristics of those patients with and without 6-month data will also be compared, to inform whether patients with no 6-month data were missing at random. Reduced multivariable analyses will be considered if necessary [e.g. removing one of two candidate prognostic factors that are highly correlated at baseline], to examine the robustness of the conclusions.

Risk groups and development of the prognostic screening tool

The prognostic model will be used to develop a risk stratification tool to inform an individual's absolute risk of poor outcome. The stratification tool will inform clinical decision-making for precision rehabilitation. Items will be selected for the model if they are statistically significantly [$p<0.05$] associated with poor outcome in the logistic regression analysis, and those deemed clinically important to retain using expert opinion [regardless of statistical significance, study steering group] to improve face validity for clinicians and avoid over-fitting of the model.[51] The regression model with included prognostic factors will be fitted to the data from the first of the two cohorts to obtain a final set of parameter estimates [i.e. alpha and beta terms], which will be used to form the model. An important requirement of the stratification tool is that it should be brief to facilitate use in clinical practice. Thus, we will look to simplify the model where possible to facilitate its use, but without important reduction in its prognostic ability in terms of calibration and discrimination. For example, if multi-item questionnaire scores are included in the model, then we will evaluate whether just

one of the questionnaire items is sufficient. Ideally, this process will result in a full and simplified model.

Development versus validation

For validation of the model, data from the second of the two cohorts will be compared to that of the first to enable analysis of the distribution of important variables; inclusive of demographic, predictor and outcome variables. The predictive performance of the screening tool [discrimination, calibration, and goodness of fit] will be assessed using data from the second cohort. Data in both cohorts will be consistent in terms of setting, eligibility criteria, outcome, and predictors.

DISCUSSION

There is an urgent need for a robust prognostic study to predict the transition from acute to chronic pain in a musculoskeletal trauma population. Using such a comprehensive array of outcome measures will allow the development and validation of a prognostic tool to predict development of chronic and disabling pain, and begin the process of identifying appropriate and precision interventions.

The candidate predictors used in this study have been chosen to be as comprehensive as possible, based on current knowledge of pain science. Other candidate predictors were considered [e.g. microRNA biomarkers], but their mechanistic functions and temporal progression are not yet sufficiently clear to justify the expense of their inclusion. The combination of patient reported outcome measures, psychophysical testing and biomarkers

For peer review only - http://bmjopen.bmj.com/site/about/guidelines.xhtml

that are included are designed to act as surrogates for the four primary mechanisms of pain: [53] [8] *nociceptive* [injury location, severity and characteristics], *neuropathic* [painDETECT tool and pain extent, *inflammatory* [biomarkers], and *central hypersensitivity* [quantitative sensory testing, painDETECT and pain location and extent]. In addition, other patient-reported outcome measures [e.g. pain intensity, post-traumatic stress, anxiety and depression, coping, and pain self-efficacy] are included as the domains that they measure have been shown to influence prognosis for long-term outcomes in populations with pain in a range of locations.[9 23 24]

Rehabilitation is widely regarded as an important component of post-trauma healthcare;[54] however, the current position of equipoise means that precision rehabilitation has not yet been identified. Understanding mechanisms that underlie the transition from acute to chronic pain is essential to moving beyond this position. Identifying prognostic factors related to poor outcome of pain and disability outcome will facilitate targeting of effective interventions. This will inform rehabilitation decision making, and facilitate improvements in clinical and cost effectiveness of rehabilitation interventions.

Limited research has identified criteria for quality in a prognostic model, but authors have identified potential quality issues to ensure methodological rigour.[55] These issues are summarised in Table 2 and incorporated into the study design to ensure low risk of bias in development and validation of the predictive model.

Table 2: Methodological decisions to improve study quality

Criteria [55]	Methodological decisions to improve quality
**Study design**	
Inception cohort	• Clear description of population<br>• Clear description of the participants at

For peer review only - http://bmjopen.bmj.com/site/about/guidelines.xhtml

	baseline
Source population	• Clear description of population<br>• Clear description of sampling frame and recruitment [method and timing]
Inclusion and exclusion criteria	• Clarity of eligibility criteria
Prospective design	• Clarity of study design
**Study attrition**	
Number of drop-outs	• Adequate participation rate<br>• Clear description of attempts to collect information on participants who dropped out<br>• Reporting numbers and reasons for loss to follow-up
Information provided on method of management of missing data	• Appropriate methods of imputation of missing data
**Predictive factors**	
All predictive factors described used to develop the model	• Clear definition of predictive factors<br>• An adequate proportion of participants has complete data for the predictive factor
Standardised or valid measurements	• The measurement of the predictive factor is reliable and valid<br>• The measurement of the predictive factor is the same for all participants
Linearity assumption studied	• Linearity of data will be reported
No dichotomization of prognostic variables	• Continuous variables will be reported where possible
Data presentation all predictive factors	• Complete data will be presented
**Outcome measures**	
Description of outcome measures	• The outcome is clearly defined
Standardised or valid measurements	• The measurement of the outcome is reliable and valid<br>• The measurement of the outcome is the same for all participants
Data presentation of most important outcome measures	• Complete data will be presented
**Analysis**	
Presentation of univariate crude estimates	• An appropriate strategy for model building is described<br>• An adequate statistical model described
Sufficient numbers of subjects per variable	• Adequate data will be presented
Selection method of variables explained	• Sufficient data will be presented to enable assessment of the adequacy of the analytic strategy<br>• All results will be reported
Presentation of multivariate estimates	• An appropriate strategy for model building is described<br>• An adequate statistical model described
**Clinical performance / validity**	
Clinical performance	• Clinical performance of the model will be reported

For peer review only - http://bmjopen.bmj.com/site/about/guidelines.xhtml

Internal validation	• Internal validation will be reported
External validation	Not a focus of this study

ETHICS AND DISSEMINATION

Ethical approval will be obtained from the NHS Research Ethics Committee, and institutional R&D approval will also be obtained.

Patient burden and potential distress

The primary ethical concern is to limit distress on participants. As such, to reduce the patient burden when collecting baseline data, the self-reported questionnaires will be administered by members of the study team shortly following obtaining fully informed consent, and physical assessment outcomes will be measured at least 24 hours later. Patients will be asked to consent to not only providing new data for the study, but also for the study team to access information that will have been routinely collected by the hospital staff since the time of admission [e.g. nature and circumstances of injury, previous medical history, medication details, blood test results]. This will be fully explained to patients and explicitly detailed in the participant information sheet.

Mental capacity

Because of the nature of their injuries, the patient's mental capacity will be assessed on admission into hospital and thereafter by clinical staff and/or research nurses. The mental capacity of eligible patients at the time of being approached for recruitment will therefore fall

For peer review only - http://bmjopen.bmj.com/site/about/guidelines.xhtml

into one of two groups: either they possess or are lacking mental capacity [in accordance with the Mental Capacity Act 2005] to provide informed consent to voluntarily participate in the study.

For patients possessing mental capacity to provide consent, a research nurse or member of the research team will ask if they are interested in participating in the study. If they are interested, a copy of the participant information sheet will be provided [and if necessary read to them] to give them an outline of the study. Following an opportunity to seek additional information and ask questions, the patient will be asked if they wish to provide their written informed consent to participate in the study, at which point a consent form will need to be signed.

On admission to the hospital, an otherwise eligible patient may lack the mental capacity to decide whether to provide consent to participate in a research study [e.g. due to the severity of their injuries, because they are arriving intubated and ventilated, or as a side-effect of medication for their injuries]. They may or may not regain this capacity during their stay in the hospital. Due to our wish to begin measuring biomarkers as early as possible following the onset of trauma, for some otherwise eligible patients it would be necessary to take blood samples before the patient has regained the capacity to provide informed consent. Using the convention of previous studies in trauma populations,[56] recruitment into the study will be undertaken under the provision and guidance of the Mental Capacity Act 2005 for research in emergency situations, and in accordance with the Declaration of Helsinki. As such, if a patient does not possess this capacity when first approached for recruitment, the research team will request a mandate to collect blood samples from a legal consultee. This mandate can continue until the patient gains sufficient capacity to make an informed decision

For peer review only - http://bmjopen.bmj.com/site/about/guidelines.xhtml

as to whether they wish to provide consent or not. We will use this mandate up to 14 days from the date of the trauma. If the patient does not regain capacity within 14 days following the trauma, or if informed consent is not provided by the patient once capacity to do so is regained, any samples collected will be destroyed before any non-clinical biomarker analysis [i.e. mitochondrial DNA] is performed. Furthermore, only once informed consent has been gained from the patient would the research team proceed to collect any self-reported questionnaire or physical assessment data. The legal consultee can either be a ‘personal consultee’ e.g. family member, or a ‘nominated consultee’ e.g. intensive care consultant. Once a consultee [personal or nominated] has been identified, they will be provided with the participant information sheet, to inform them about the study. The consultee will be asked if they feel participating in the study would be something to which the patient would agree or object to. If, in their opinion, the patient would agree to participating in the study, the consultee will be asked to sign a declaration form, and the research team can begin the schedule of blood sample collections. If, at any time prior to the patient regaining capacity, the consultee decides to withdraw assent, then no further samples will be collected until the patient can be approached for formal recruitment [if appropriate].

Other ethical issues

Participants will be informed that they are free to withdraw from the study at any time, without needing to provide reason. At each data collection visit, the capacity of the participant will be checked [using an Abbreviated Mental Test, if deemed necessary] and asked if they are happy to proceed with data collection. Any concerns for a participant by the study team will be fed back to clinical staff. All blood samples will be collected by hospital staff and the research nurse team and will be stored, tested and disposed of in accordance

For peer review only - http://bmjopen.bmj.com/site/about/guidelines.xhtml

with current United Kingdom guidelines and regulations. In the event of death within 3 months of being recruited, participants will be automatically withdrawn from the study and the primary prognostic analysis. Baseline characteristics of withdrawn participants will be compared to those of retained participants to assess for any differences.

For peer review only

REFERENCES

1. Berben SA, Schoonhoven L, Meijs TH, et al. Prevalence and relief of pain in trauma patients in emergency medical services. *Clin J Pain* 2011;27(7):587-92. doi: 10.1097/AJP.0b013e3182169036
2. Williamson OD, Epi GD, Gabbe BJ, et al. Predictors of moderate or severe pain 6 months after orthopaedic injury: a prospective cohort study. *J Orthop Trauma* 2009;23(2):139-44. doi: 10.1097/BOT.0b013e3181962e29
3. Mannion RJ, Woolf CJ. Pain mechanisms and management: a central perspective. *Clin J Pain* 2000;16(3 Suppl):S144-56.
4. Holbrook TL, Anderson JP, Sieber WJ, et al. Outcome after major trauma: discharge and 6-month follow-up results from the Trauma Recovery Project. *J Trauma* 1998;45(2):315-23; discussion 23-4.
5. Holbrook TL, Anderson JP, Sieber WJ, et al. Outcome after major trauma: 12-month and 18-month follow-up results from the Trauma Recovery Project. *J Trauma* 1999;46(5):765-71; discussion 71-3.
6. Harris IA, Young JM, Rae H, et al. Predictors of general health after major trauma. *J Trauma* 2008;64(4):969-74. doi: 10.1097/01.ta.0000245972.83948.1a
7. Ponsford J, Hill B, Karamitsios M, et al. Factors influencing outcome after orthopedic trauma. *J Trauma* 2008;64(4):1001-9. doi: 10.1097/TA.0b013e31809fec16
8. Vardeh D, Mannion RJ, Woolf CJ. Toward a Mechanism-Based Approach to Pain Diagnosis. *J Pain* 2016;17(9 Suppl):T50-69. doi: 10.1016/j.jpain.2016.03.001
9. Artus M, Campbell P, Mallen CD, et al. Generic prognostic factors for musculoskeletal pain in primary care: a systematic review. *BMJ Open* 2017;7(1):e012901. doi: 10.1136/bmjopen-2016-012901
10. Latremoliere A, Woolf CJ. Central sensitization: a generator of pain hypersensitivity by central neural plasticity. *J Pain* 2009;10(9):895-926. doi: 10.1016/j.jpain.2009.06.012
11. Linley JE, Rose K, Ooi L, et al. Understanding inflammatory pain: ion channels contributing to acute and chronic nociception. *Pflugers Arch* 2010;459(5):657-69. doi: 10.1007/s00424-010-0784-6
12. CJ. W. Central sensitization: implications for the diagnosis and treatment of pain. *Pain* 2011;152(3):S2-15.
13. Altman DG, Vergouwe Y, Royston P, et al. Prognosis and prognostic research: validating a prognostic model. *BMJ* 2009;338(338):b605. doi: 10.1136/bmj.b605
14. Moons KG, Kengne AP, Grobbee DE, et al. Risk prediction models: II. External validation, model updating, and impact assessment. *Heart* 2012;98(9):691-8. doi: 10.1136/heartjnl-2011-301247
15. Shmueli G. To explain or to predict? *Statistical Science* 2010;25(3):289-310.
16. Collins GS, Reitsma, J.B., Altman, D.G. and Moons, K.G., . Transparent reporting of a multivariable prediction model for individual prognosis or diagnosis (TRIPOD): the TRIPOD statement. *BMC Medicine* 2015;13(1)
17. Merskey H BN. Classification of chronic pain : descriptions of chronic pain syndromes and

definitions of pain terms. USA: IASP Press 1994.

18. Teasdale G, Jennett B. Assessment of coma and impaired consciousness. A practical scale. *Lancet* 1974;2(7872):81-4.
19. Reith FC, Van den Brande R, Synnot A, et al. The reliability of the Glasgow Coma Scale: a systematic review. *Intensive Care Med* 2016;42(1):3-15. doi: 10.1007/s00134-015-4124-3
20. HM H. Evaluation of a mental test score for assessment of mental impairment in the elderly. *Age and Ageing* 1972;1(4):233-8.

For peer review only - http://bmjopen.bmj.com/site/about/guidelines.xhtml

21. Von Korff M, Ormel J, Keefe FJ, et al. Grading the severity of chronic pain. *Pain* 1992;50(2):133-49.
22. Parsons S, Breen A, Foster NE, et al. Prevalence and comparative troublesomeness by age of musculoskeletal pain in different body locations. *Fam Pract* 2007;24(4):308-16. doi: 10.1093/fampra/cmm027
23. Muller S, Thomas E, Dunn KM, et al. A prognostic approach to defining chronic pain across a range of musculoskeletal pain sites. *Clin J Pain* 2013;29(5):411-6. doi: 10.1097/AJP.0b013e318257099e
24. Rivara FP, Mackenzie EJ, Jurkovich GJ, et al. Prevalence of pain in patients 1 year after major trauma. *Arch Surg* 2008;143(3):282-7; discussion 88. doi: 10.1001/archsurg.2007.61
25. Organization WH. The International Classification of Functioning, Disability and Health (ICF). Geneva: WHO 2001.
26. Dixon D PD, Johnston M. What does the chronic pain grade questionnaire measure? *Pain* 2007;130:249-53.
27. Smith BH, Penny KI, Purves AM, et al. The Chronic Pain Grade questionnaire: validation and reliability in postal research. *Pain* 1997;71(2):141-7.
28. Penny KI PA, Smith BH, Chambers WA, Smith WC. Relationship between the chronic pain grade and measures of physical, social and psychological well-being. *pain* 1999;79:275–9.
29. Elliott AM SB, Smith WC, Chambers WA. Changes in chronic pain severity over time: The Chronic Pain Grade as a valid measure. *Pain* 2000;88:303.
30. Von Korff M, Jensen MP, Karoly P. Assessing global pain severity by self-report in clinical and health services research. *Spine (Phila Pa 1976)* 2000;25(24):3140-51.
31. Von Korff M, Miglioretti DL. A prognostic approach to defining chronic pain. *Pain* 2005;117(3):304-13. doi: 10.1016/j.pain.2005.06.017
32. Von Korff M, Dunn KM. Chronic pain reconsidered. *Pain* 2008;138(2):267-76. doi: 10.1016/j.pain.2007.12.010
33. Dunn KM, Croft PR, Main CJ, et al. A prognostic approach to defining chronic pain: replication in a UK primary care low back pain population. *Pain* 2008;135(1-2):48-54. doi: 10.1016/j.pain.2007.05.001
34. Thomas E, Dunn KM, Mallen C, et al. A prognostic approach to defining chronic pain: application to knee pain in older adults. *Pain* 2008;139(2):389-97. doi: 10.1016/j.pain.2008.05.010
35. Ware J. SF-36 Health Survey Updated. *Spine* 2000;25(24):3130-39.
36. Brooks R. EuroQol: the current state of play. *Health Policy* 1996;37(1):53-72.
37. Mahoney FI, Barthel DW. Functional Evaluation: The Barthel Index. *Md State Med J* 1965;14:61-5.
38. Zigmond AS, Snaith RP. The hospital anxiety and depression scale. *Acta Psychiatr Scand* 1983;67(6):361-70.
39. Harland NJ GK. Development of the coping strategies questionnaire 24, a clinically utilitarian version of the coping strategies questionnaire. *Rehabilitation Psychology* 2003;48(4):296–300.
40. Woby SR, Roach NK, Urmston M, et al. Psychometric properties of the TSK-11: a shortened version of the Tampa Scale for Kinesiophobia. *Pain* 2005;117(1-2):137-44. doi: 10.1016/j.pain.2005.05.029
41. M.K. N. The pain self-efficacy questionnaire: Taking pain into account. *Eur J Pain* 2007;11(2):153-63.
42. Beck JG, Grant DM, Read JP, et al. The impact of event scale-revised: psychometric properties in a sample of motor vehicle accident survivors. *J Anxiety Disord* 2008;22(2):187-98. doi: 10.1016/j.janxdis.2007.02.007
43. Baker SP, O'Neill B, Haddon W, Jr., et al. The injury severity score: a method for describing patients with multiple injuries and evaluating emergency care. *J Trauma* 1974;14(3):187-96.
44. Masters Steedman S, Middaugh SJ, Kee WG, et al. Chronic-pain medications: equivalence levels and method of quantifying usage. *Clin J Pain* 1992;8(3):204-14.

For peer review only - http://bmjopen.bmj.com/site/about/guidelines.xhtml

45. Harden RN, Weinland SR, Remble TA, et al. Medication Quantification Scale Version III: update in medication classes and revised detriment weights by survey of American Pain Society Physicians. *J Pain* 2005;6(6):364-71. doi: 10.1016/j.jpain.2005.01.350
46. Gallizzi M GC, Harden RN, Stanos S, Khan A. Medication Quantification Scale Version III: internal validation of detriment weights using a chronic pain population. . *Pain Pract* 2008;8(1):1-4.
47. Barbero M, Mores, F., Leoni, D., Gatti, R., Egloff, M., Falla, D. Test-retest reliability of pain extent and pain location using a novel method for pain drawing analysis. *European Journal of Pain* 2015;19:1129-38.
48. Freynhagen R, Baron R, Gockel U, et al. painDETECT: a new screening questionnaire to identify neuropathic components in patients with back pain. *Curr Med Res Opin* 2006;22(10):1911-20. doi: 10.1185/030079906X132488
49. Peduzzi P, Concato J, Kemper E, et al. A simulation study of the number of events per variable in logistic regression analysis. *J Clin Epidemiol* 1996;49(12):1373-9.
50. Vittinghoff E, McCulloch CE. Relaxing the rule of ten events per variable in logistic and Cox regression. *Am J Epidemiol* 2007;165(6):710-8. doi: 10.1093/aje/kwk052
51. KL S. Multivariate Regression: The Pitfalls of Automated Variable Selection. *Physical Medicine and Rehabilitation* 2013;5:791-94,.
52. Sterne JA, White IR, Carlin JB, et al. Multiple imputation for missing data in epidemiological and clinical research: potential and pitfalls. *BMJ* 2009;338:b2393. doi: 10.1136/bmj.b2393
53. Woolf CJ, Bennett GJ, Doherty M, et al. Towards a mechanism-based classification of pain? *Pain* 1998;77(3):227-9.
54. NICE. Rehabilitation after critical illness in adults Clinical guideline [CG83]. 2009
55. van Oort L, van den Berg T, Koes BW, et al. Preliminary state of development of prediction models for primary care physical therapy: a systematic review. *J Clin Epidemiol* 2012;65(12):1257-66. doi: 10.1016/j.jclinepi.2012.05.007
56. Hazeldine J, Naumann DN, Toman E, et al. Prehospital immune responses and development of multiple organ dysfunction syndrome following traumatic injury: A prospective cohort study. *PLoS Med* 2017;14(7):e1002338. doi: 10.1371/journal.pmed.1002338

Authors' contributions

DF and AR are the Chief Investigators leading protocol development, analyses and dissemination. DE is the Research Fellow with responsibility for study management. NM is a Doctoral Researcher focused to this study. NH is the lead for Patient and Public Involvement. DF and AR are overseeing data analysis. JP is the Trust Principal Investigator. JP and CS are clinical leads at the NHS Trust. JL is the lead for biomarker evaluation. All authors will contribute to data interpretation, conclusions, and dissemination. AR drafted the initial manuscript with DF. Subsequent drafts were developed with DE. All reviewers have read, contributed to, and agreed the final manuscript. DF is the guarantor.

For peer review only

For peer review only - http://bmjopen.bmj.com/site/about/guidelines.xhtml

Funding statement

This work was supported by the National Institute for Health Research [NIHR] to DF and AR.

Competing interests

There are no competing interests.

Data sharing statement

No additional data are available.

For peer review only

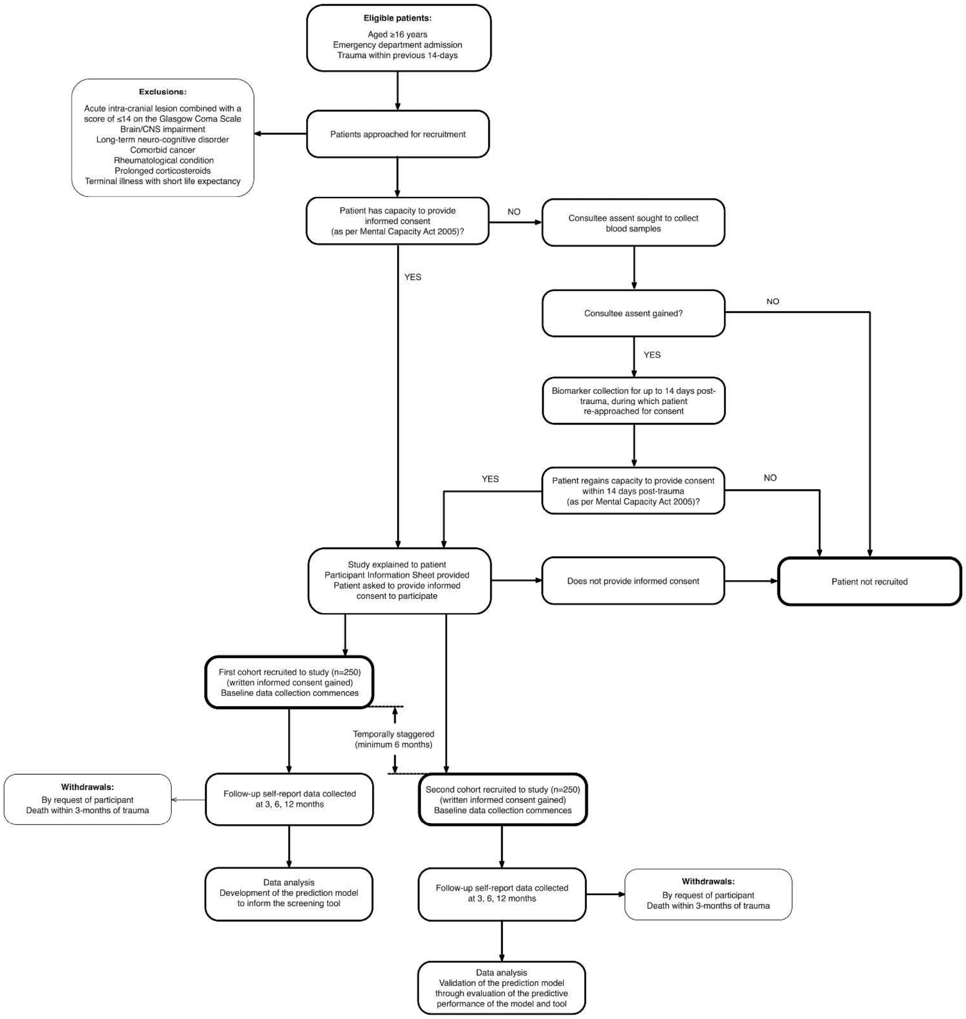

Study design

500x521mm (300 x 300 DPI)

Supplementary file 1. Candidate predictive factors

General participant characteristics

Several participant demographic features will be recorded at baseline, based on available hospital records and patient self-reported recollection, including smoking status, age, gender, height and weight to calculate body mass index [BMI], education [highest attained educational level], employment status [at the time of trauma], circumstance of trauma [military or civilian], previous history of musculoskeletal pain and injury, comorbidity of other current health problems.

Quality of life and physical functioning

<u>36-Item Short Form Health Survey, Version 2.0 [SF-36v2]</u>

The SF-36v2 is a self-reported measure of health-related quality of life, modified from the original SF-36, which was developed as part of the Medical Outcomes Study.[1] The 36-item questionnaire has subscales that assess physical, function, social and psychological wellbeing.[2 3] The scores can be divided into physical and mental component summary scales.[4] The SF-36 has been shown to be valid and has been tested extensively in a trauma population.[5] Ware[6] reports multiple studies showing internal consistency above 0.70, with physical and mental scores exceeding 0.90. Minimal clinically important difference has been reported as 5.5 in a musculoskeletal trauma population.[7] Introduced in 1996, version 2.0 of the SF-36 is comparable to the original, retaining all subscales, with improvements to layout, presentation, response scales, wording and scoring.[6] The 'acute' [1 week recall period] version will be used, since the 4 week recall would not be appropriate for post-injury recall at baseline.

For peer review only - http://bmjopen.bmj.com/site/about/guidelines.xhtml

EuroQol Five Dimension Scale, 5-level [EQ-5D-5L]

Health-related quality of life will be quantified using the EQ-5D-5L through which 243 possible health states are converted to a single index value of range 0 to 1 where 1 is perfect health, and a visual analogue scale range 0–100, representing 'worst' to 'best' imaginable health state, respectively.[8] The EQ-5D-5L, with each item having 5 possible responses, has improved inter-observer [ICC 2,1 0.57] and test-retest [ICC 2,1 0.69] reliability compared to the previous EQ-5D-3L.[9] In addition, it has less ceiling effects [20.8% reduction] and adequate convergent validity when compared with the WHO-5 [spearman rank 0.38-0.51].[10]

Barthel Index of Activities of Daily Living

The Barthel Index of Activities of Daily Living is routinely collected by clinical staff at the hospital, and will be used to evaluate self-care and mobility during activities of daily living.[11] [12] It is a 10-item ordinal scale encompassing a range of mobility physical activity tasks. Each item is related to a specific task and rated with a given number of points. A score of '0' is given for least independence/function on that item and scores above that [1 or 2] are given for increasing independence/function [range: 0-20]. The amount of time and physical assistance required to perform each task are used in determining the assigned value of each item. A higher score is associated with a greater likelihood of being able to live at home with a degree of independence following discharge from hospital. With most measurement testing performed in the stroke population, the Barthel Index has demonstrated excellent internal consistency [0.89-0.90][13] and is highly responsive in detecting changes[14] with a minimal detectable change of 4.02 and minimally clinically important difference of 1.85.[15] High correlations have been demonstrated with the Functional Independence Measure [FIM], indicating convergent validity of the instrument.[16]

Subjective Health Complaints Inventory

For peer review only - http://bmjopen.bmj.com/site/about/guidelines.xhtml

Sleep quality will be assessed using the single-item questions from the sleep domain of the Subjective Health Complaints Inventory.[17] The Subjective Health Complaints Inventory has been shown to be a reliable measure of recording subjective health complaints;[17] although no study has focused on the reliability of using the single item relating to sleep.

Psychosocial features

The Hospital Anxiety and Depression Scales [HADS]

The HADS will be used to measure depression and anxiety, and their role in the manifestation of somatic symptoms.[18] There are 7 items which produce a cumulative score [range 0–21] for the anxiety [HADS-A] and depression [HADS-D] subscales, with a higher score indicative of greater anxiety and depression.[19] HADS has been tested in multiple populations demonstrating adequate to excellent internal consistency of HADS-A [0.68-0.93] and HADS-D [0.67-0.90].[19] Standard measurement of error in a coronary heart disease population was identified as 1.37 and 1.44 for anxiety and depression scales respectively, and minimal detectable change as 3.80 and 3.99 respectively.[20] The HADS has also demonstrated excellent concurrent validity when compared to various other depression/anxiety scales.[19]

Coping Strategies Questionnaire 24 [CSQ-24]

The CSQ-24 will be used to provide an indication of coping strategies used by participants when they are in pain.[21] Developed from items from the earlier, much larger Coping Strategies Questionnaire,[22] the CSQ-24 is a 23-item scale, composed of 4 subscales: *catastrophizing* [6 items], *diversion* [6 items], *reinterpreting* [6 items], and *cognitive coping* [5 items]. Participants are asked to indicate if they have particular thoughts and feelings when they are experiencing pain. A score on each item is summed to yield an aggregate score for

For peer review only - http://bmjopen.bmj.com/site/about/guidelines.xhtml

each subscale, with a higher score reflecting greater attribution of that particular coping strategy. The CSQ-24 has demonstrated good internal consistency in populations with low back pain patients [Chronbach's alpha for the 4 factors ranged from 0.75 to 0.85] and work-related pain [0.80 to 0.86][23] Harland & Georgieff suggested that, since individuals may have a positive score on more than one subscale, the highest scoring subscale should be deemed the dominant coping strategy.[21] However, a recent study in a low back pain cohort,[24] in which individual items from multiple questionnaires were factorised, suggested that *diversion*, *reinterpreting* and *cognitive coping* clustered together as a single factor, representing coping cognitions. By contrast, *catastrophizing* clustered with pain-related distress items.

<u>Tampa Scale of Kinesiophobia [TSK-11]</u>

The TSK-11 will be used to assess fear of movement or fear of injury or re-injury during movement.[25] It is an 11-item questionnaire, eliminating psychometrically poor items from its original 17-item version,[26] thus creating a shorter questionnaire with comparable internal consistency and a 2-factor structure [activity avoidance and harm]. Each of the 11 items is measured using a 4-point scale using the end points 1 ['totally agree'] and 4 ['totally disagree'] [scoring range 11–44]. Higher scores indicate more fear-avoidance behaviour. The TSK-11 has demonstrated acceptable to good internal consistency in acute and chronic musculoskeletal pain populations.[25 27] Test-retest reliability has been reported as excellent with a high standardised response mean; with good construct validity in relation to changes in disability and pain.[25]

<u>Pain Self-Efficacy Questionnaire [PSEQ]</u>

The patient's confidence in their ability to perform activities despite their pain will be evaluated using the PSEQ. Developed from the Self-Efficacy Scale,[28] the PSEQ consists of

For peer review only - http://bmjopen.bmj.com/site/about/guidelines.xhtml

10 physical and psychosocial activity items measuring from 0 [‘not at all confident’] to 6 [‘completely confident’] thus generating a total score from 0-60.[29] The PSEQ has demonstrated excellent internal consistency [0.92], internal reliability [0.93], and test-re-test correlations [r=0.73] and has demonstrated validity when compared to other self-efficacy measurements.[29] It has been used in several large population studies, for example Campbell et al.[24]

For peer review only

Impact of Event Scale Revised [IES-R]

The IES-R will be used to measure the subjective stress experienced by the participant following their traumatic event. The IES-R is a 22-item tool [range: 0-88] that consists of 8 intrusion and 8 avoidance items that are derived from the original IES,[30] with an additional 7-items assessing hyperarousal.[31] Accordingly, items correspond directly to symptoms of post-traumatic stress disorder.[31] Respondents are asked to identify a specific stressful life event and then indicate how much they were distressed or bothered during the past seven days by each ‘difficulty’ listed. Each item is rated on a 5-point scale ranging from 0 [‘not at all’] to 4 [‘extremely’]. The IES-R yields a total score ranging 0 to 88 and subscale scores can also be calculated for the Intrusion, Avoidance, and Hyperarousal sub-scales. The IES-R has demonstrated good internal consistency for all subscales [intrusion 0.87-0.94, avoidance 0.84-0.97, hyperarousal 0.79-0.91].[32] High correlations have been found between the IES-R and the original scale, supporting the concurrent validity of both measures.[31]

Injury characteristics

Several measures relating to the characteristics of the sustained injury will be taken at baseline. The time of the injury will be gained from hospital records. The location of the injury/injuries will be recorded using an adapted version of previously developed pain

For peer review only - http://bmjopen.bmj.com/site/about/guidelines.xhtml

drawing software, via a tablet computer.[33] Information relating to the tissues damaged from the injury [e.g. fractures sustained, whether the injury was penetrating, non-penetrating or both, review of available imaging data] will be gathered from hospital records, where possible. Whether the participant received surgery following their admission [where, for what and when], and whether the participant received assisted mechanical ventilation will also be recorded.

Injury Severity Scale [ISS]

The ISS will be retrospectively calculated for each participant, including those who withdraw. The ISS is a numerical score with a range 0-75, that is used to describe the overall severity of injury, and can be used for both multiple and single injuries. The score is calculated, based on the Abbreviated Injury Scale [AIS] scores.[34 35] Higher ISS scores have been associated with increased rates of mortality[34 36 37] and length/cost of hospital stay.[38] It is the recommended tool for summarising injury severity by the Trauma Audit and Research Network [TARN]. Both TARN and the National Institute for Clinical Excellence[39] recommend any participant with a score of >8 to be referred for rehabilitation.

Pain characteristics

Pain intensity

Pain intensity will be measured using an 11-point [0-10] Numerical Rating Scale [NRS], measuring current pain from 'no pain' to 'pain as bad as could be', from the Chronic Pain Grade Scale.[40] We will aim to assess *current* pain intensity at baseline, as frequently as every 48-hours while the participant is in hospital [depending upon participant accessibility and assessor availability], to gain accurate mean and rate-of-change data. At the 6 and 12 month assessment points, c*urrent* pain intensity, as well as *average* and *worst* pain intensity related

For peer review only - http://bmjopen.bmj.com/site/about/guidelines.xhtml

to the preceding 6 months, will be collected as part of the Chronic Pain Grade Scale. NRS scales are sensitive, reliable and valid instruments for pain intensity measurement,[41-44] and have been recommended for use in clinical populations in preference to visual analogue scales or verbal rating scales.[45] A 30% change on a pain NRS score is considered clinically meaningful.[46-50]

Pain medication

The patient's pain medication [type, dosage and time since trauma] intake will be noted and the Medication Quantification Score [MQS], which is a reliable and validated score for quantifying analgesics, will be calculated to obtain a comparable metric for all different analgesics.[51-53] It enables characterisation of analgesics when many different medications are involved and doses are irregular. It will be calculated for each non-opioid and opioid, based on weights assigned by medication class and dosage level [level 1 = sub-therapeutic dosage and/or on demand, level 2 = lower 50% of the therapeutic dose range, level 3 = upper 50% of the therapeutic dose range, level 4 = supra-therapeutic dose] using the 1998 detriment weights.[54] The detriment weights are summed by the dosage level to provide the final score. These scores will be summed to provide a quantitative index for analgesic usage suitable for statistical analysis.

Pain drawing

All participants will be requested to complete a pain drawing, indicating the spatial distribution of their pain, over two body charts; one reporting a frontal view of the body and one a dorsal view. We will also ask patients to mark their single 'most painful' site on one of these body charts. Pain drawing data will be collected using a custom software application on a tablet computer, and will be analysed with Matlab software, as described previously.[33] The

For peer review only - http://bmjopen.bmj.com/site/about/guidelines.xhtml

For peer review only

software automatically calculates the number of shaded pixels from the pain drawing, which is defined as *pain extent*. Summaries of, and relationships between, pre-defined painful body regions will also be evaluated. Conventional pain drawing data will also be collected on paper follow-up questionnaires to assess painful body regions.

The painDETECT questionnaire

It is assumed that all post-trauma patients will have significant nociception at baseline, but the contribution of other pain-related mechanistic pathways will also be assessed. The painDETECT questionnaire[55] will be used to facilitate the identification of neuropathic pain. It consists of 9 items [7 evaluating pain quality, 1 evaluating pain pattern, and 1 evaluating pain radiation], all of which contribute to an aggregate score [range: -1-38]. This aggregate score can be divided into three classifications that represent the likelihood of neuropathic pain: ‘unlikely’ [0-12], ‘ambiguous’ [13–18] and ‘likely’ [19–38].[55] Although developed as a screening questionnaire for neuropathic pain, painDETECT may also function as a measure of characteristics that indicate augmented central pain processing.[55] The painDETECT questionnaire has demonstrated good internal consistency [0.76][56] and excellent test-re-test reliability[57] within 1-hour of consultation [ICC{model not reported} 0.911] and 1-week post consultation [ICC{model not reported} 0.79].[58] Convergent validity has been demonstrated in comparison to pain severity,[56 59] health-related quality of life[60] and similar neuropathic pain screening tools.[55] As such, painDETECT outcomes will be measured at regular intervals while the participant is an inpatient in the hospital [subject to participant accessibility and assessor availability] to assess for emerging neuropathic pain and sensitization. Measurements will also be taken at all follow-up assessment points.

Quantitative sensory testing [QST]

For peer review only - http://bmjopen.bmj.com/site/about/guidelines.xhtml

QST methods will be used to assess pain sensibility, throughout which measurements will be concealed from participants. Owing to the clinical heterogeneity of the post-trauma population, precise standardisation of test sites between participants will not be possible. Instead, we have developed a standardised protocol that will be used to evaluate pain thresholds for multiple stimulus modalities [mechanical pressure, heat and cold] at the same sites in each participant. Each site where multi-modality pain threshold testing is performed will be within the receptive field of the same nerve root using described regions,[61] so that segmental cross-modality excitability may be compared. All pain thresholds will be measured at the same 'local' and 'remote' sites for each participant. We define local sites as being uninjured but within [or, if not accessible, as close as possible to] the same receptive field as the most painful inured tissue [e.g. skin over gastrocnemius in a participant with an ankle fracture]. By contrast, we define remote sites as a distant, accessible, site from the receptive fields in which tissues are injured [e.g. skin over tibialis anterior in a participant without lower limb injury], and on the contralateral side of the body where injured tissue is unilateral. Where possible, remote sites will be a mirror-image of the local site [to allow for comparison of absolute values], but in a trauma population we are aware that this may not always be possible. For all threshold testing modalities, an ascending method of limits design[62] will be used, whereby stimulus intensity will begin at a low level and gradually increase until the participant first perceives pain. Participants will be instructed to push a button or tell the assessor when the sensation has changed from one of the stimulus alone [e.g. just pressure] to a sensation of both the stimulus and pain [e.g. pressure and pain]. Following a brief demonstration of equipment to familiarise participants, two consecutive assessments will be performed for each modality at each site, and the means used for further analysis.[63] A minimum of 30-seconds inter-stimulus interval will be given between each threshold

For peer review only - http://bmjopen.bmj.com/site/about/guidelines.xhtml

measurement within a single session. Measurements will be taken at baseline, while the participant is an inpatient in the hospital; we will aim to collect data as frequently as every 48 hours to gain accurate rate-of-change data, but this will depend upon participant accessibility and assessor availability. To ensure pain thresholds are consistently measured at the same sites every session, sites will be labelled using a sterile, skin marking pen [Schuco Ltd, UK]. Because sites cannot be standardised between participants, the rates-of-change of these values will be used as candidate predictive variables, to allow for comparisons between participants. The order of pain threshold testing will be randomly assigned by modality at each session to avoid order effects.

Thermal [heat and cold] pain thresholds will be measured using skin-contact stimulation, using the same thermode at the same sites, within specified local and remote dermatomes. Thermal pain threshold assessments will be performed by delivering thermal stimuli directly to the skin through a metal 30x30 mm Peltier thermode, using a TSA-II NeuroSensory Analyzer thermal stimulator and accompanying software [Medoc Ltd, Israel]. To evaluate heat pain threshold, temperature will be gradually increased, at a rate of 1°C/s from a 'neutral' baseline of 32°C, to a maximum temperature of 50.5°C to avoid thermal injury.[64] During each measurement, participants will be instructed to press a button when the stimulus becomes painful, and this will be documented as the threshold value. Once pain threshold is achieved [and recorded], the temperature will return to the baseline value at the same rate [1°C/s]. For cold pain threshold measurements, the temperature will be gradually reduced, at a rate of 1°C/s from the baseline of 32°C, to a minimum temperature of 0°C,[64] before also returning to baseline at a rate of 1°C/s.

For peer review only - http://bmjopen.bmj.com/site/about/guidelines.xhtml

Pressure pain thresholds will be measured using a digital pressure algometer [Series 7 force gauge, Mark-10 Corporation, USA], providing real-time force measurement and an analogue output that can be linked to a computer. Skin and muscle tissue are simultaneously stimulated during pressure threshold testing; sites will therefore be chosen where a dermatome and myotome are likely to share a common nerve root innervation [e.g. skin over tibialis anterior]. The algometer has a hard rubber circular contact tip of 1.2cm^2 area, with no sharp edges so to avoid an uneven pressure stimulus.[65] In order to preserve hygiene and attend to infection control measures in trauma patients, the contact tip will be covered with a clean, thin disposable covering. The tip will be applied perpendicular to the skin at a constant rate of pressure increase of 50kPa/s [6.0N/s using the 1.2cm^2 tip], until the first onset of pain. For each measurement, pressure will be unloaded immediately once the participant indicates that their pain threshold has been reached.

To measure excitability of nociceptive pathways in response to mechanical stimuli, a series of repetitive, pressure stimulus 'pulses' will be applied via the digital algometer, with the aim of provoking temporal summation responses.[66-68] A minimum of 2 minutes after all threshold tests have been completed, a series of 10 consecutive pressure pulses will be applied at the remote and local sites [the order of site being randomly assigned]. The peak pressure reached during each pulse will be the mean pressure pain threshold that was measured for that particular site, as described previously. For each pulse, pressure will be gradually increased to the peak value over a period of 5 seconds, maintained at that peak value for 1 second, and then immediately released. A 5 second inter-stimulus interval will be used between pulses, during which the tip of the algometer will remain in contact with the skin. Pain intensity from the pulses will be rated on a numerical rating scale [0 being 'no pain' to 10 being 'pain as bad as could be']. In the event that participants indicate that pain has become intolerable, the

For peer review only - http://bmjopen.bmj.com/site/about/guidelines.xhtml

sequence will be stopped immediately, and the NRS score and number of impulses performed at that point will be noted.

Biomarkers

Serum levels of C-reactive protein [CRP] will be used as a biomarker for inflammation. CRP is an acute-phase response protein produced by hepatocytes and is usually found in concentrations of 0.3 to 1.7 mg/l[69]. Increased production is due to cytokine-dependent induction of synthesis and elevated levels may be detected within eight hours of a stimulus and can reach 500 mg/1.28. Besides trauma,[70] elevated levels of CRP may be seen in conditions such as autoimmune disease, infection and malignancy. It has also been associated with acute sciatica.[71] The level of CRP usually peaks within 48 hours of the stimulus. In contrast, when the stimulus for increased production completely ceases, the circulating CRP concentration falls rapidly, at almost the rate of plasma CRP clearance.[72] A fall in serial measurements usually indicates resolution of the underlying process, while persisting elevated levels indicates ongoing inflammation.[73] Where possible, measurements of serum CRP will be repeatedly taken on a 48 hour schedule while the participant is an inpatient; absolute and rates of change of CRP values will be used as candidate predictive factors.[74]

Mitochondrial DNA [mtDNA] will be used as an indicator of tissue damage. It is released from cells when they are damaged and is thought to be one of the important initiators of systemic inflammatory responses following tissue injury known as Damage Associated Molecular Patterns [DAMPs]. [75] Clinical outcomes in trauma patients have been related to plasma mtDNA concentration.[76 77] Other work with severe trauma patients has shown that mtDNA values rise to their peak value in the second week post-trauma, and then gradually

For peer review only - http://bmjopen.bmj.com/site/about/guidelines.xhtml

return to baseline values after approximately 2 months.[78] Where possible, measurements of serum mtDNA will be repeatedly taken on a 48 hour schedule while the participant is an inpatient; absolute values and rates of change of mtDNA values will be used as candidate prognostic factors.

For peer review only

For peer review only - http://bmjopen.bmj.com/site/about/guidelines.xhtml

References

1. Stewart AL, Greenfield S, Hays RD, et al. Functional status and well-being of patients with chronic conditions. Results from the Medical Outcomes Study. *JAMA* 1989;262(7):907-13.
2. Ware JE, Jr., Sherbourne CD. The MOS 36-item short-form health survey (SF-36). I. Conceptual framework and item selection. *Med Care* 1992;30(6):473-83.
3. Hays RD, Sherbourne CD, Mazel RM. The RAND 36-Item Health Survey 1.0. *Health Econ* 1993;2(3):217-27.
4. McHorney CA, Ware JE, Jr., Raczek AE. The MOS 36-Item Short-Form Health Survey (SF-36): II. Psychometric and clinical tests of validity in measuring physical and mental health constructs. *Med Care* 1993;31(3):247-63.
5. Revell MP, Pynsent, P.B., Abudu, A., Fairbank, J.C.T. . Trauma Scores and Trauma Outcome Measures *Trauma* 2003;5:61-70.
6. Ware J. SF-36 Health Survey Updated. *Spine* 2000;25(24):3130-39.
7. Dattani R, Slobogean, G.P., O'Brien, P.J., Broekhuyse, H.M., Blachut, P.A., Guy, P., Lefaivre, K.A. Psychometric Analysis of Measuring Functional Outcomes in Tibial Plateau Fractures using the Short Form (SF-36), Short Musculoskeletal Function Assessment (SMFA) and the Western Ontario McMaster Osteoarthritis (WOMAC) Questionnaires. *Injury Int J Care Injured* 2013;44:825-29.
8. Brooks R. EuroQol: the current state of play. *Health Policy* 1996;37(1):53-72.
9. Janssen MF, Birnie E, Haagsma JA, et al. Comparing the standard EQ-5D three-level system with a five-level version. *Value Health* 2008;11(2):275-84. doi: 10.1111/j.1524-4733.2007.00230.x
10. Janssen MF, Pickard AS, Golicki D, et al. Measurement properties of the EQ-5D-5L compared to the EQ-5D-3L across eight patient groups: a multi-country study. *Qual Life Res* 2013;22(7):1717-27. doi: 10.1007/s11136-012-0322-4
11. Mahoney FI, Barthel DW. Functional Evaluation: The Barthel Index. *Md State Med J* 1965;14:61-5.
12. Shah S, Vanclay F, Cooper B. Improving the sensitivity of the Barthel Index for stroke rehabilitation. *J Clin Epidemiol* 1989;42(8):703-9.
13. Hseuh IP, Lee, M.M., Hsieh, C.L. . Psychometric Characteristics of the Barthel Activities of Daily Living Index in Stroke Patients *J Formos Med Assoc* 2001;100(526-532)
14. Hseuh IP, Lin, J.H., Jeng, J.S., Hsieh, C.L. Comparison of the Psychometric Characteristics of the Functional Independence Measure, 5 Item Barthel Index and 10 Item Barthel Index in Patients with Stroke. *J Neurol Neurosurg Psychiatry* 2002;73:188-90.
15. Hsieh YW, Wang, C.H., Wu, S.C., Chen, P.C., Sheu, C.F., Hsieh, C.L. . Establishing the Minimally Clinically Important Difference of the Barthel Index in Stroke Patients. *Neurorehabilitation and Neural Repair* 2007;21(3):233-38.
16. Hobart JC, Thompson AJ. The five item Barthel index. *J Neurol Neurosurg Psychiatry* 2001;71(2):225-30.
17. Eriksen HR, Ihlebaek C, Ursin H. A scoring system for subjective health complaints (SHC). *Scand J Public Health* 1999;27(1):63-72.
18. Zigmond AS, Snaith RP. The hospital anxiety and depression scale. *Acta Psychiatr Scand* 1983;67(6):361-70.
19. Bjelland I, Dahl AA, Haug TT, et al. The validity of the Hospital Anxiety and Depression Scale. An updated literature review. *J Psychosom Res* 2002;52(2):69-77.
20. Wang W, Chair, S.Y., Thompson, D.R., Twinn, S.F. A Psychometric Evaluation of the Chinese Version of the Hospital Anxiety and Depression Scale in Patients with Coronary Heart Disease *Journal of Clinical Nursing* 2009;18:1908-15.
21. Harland NJ GK. Development of the coping strategies questionnaire 24, a clinically utilitarian version of the coping strategies questionnaire. *Rehabilitation Psychology* 2003;48(4):296–300.

For peer review only - http://bmjopen.bmj.com/site/about/guidelines.xhtml

22. Rosenstiel AK KF. The use of coping strategies in chronic low back pain patients: Relationship to patient characteristics and current adjustment. . *Pain* 1983;17: 33–44.
23. Chiu C JJ, Fujikawa M, Strand D, Cheing G, Lee G, Chan F Measurement Structure of the Coping Strategies Questionnaire-24 in a Sample of Individuals with Musculoskeletal Pain: A Confirmatory Factor Analysis. . *Rehabilitation Research, Policy, and Education* 2014;28(2):80-90.
24. Paul Campbell NEF, Elaine Thomas, and Kate M. Dunn. Prognostic Indicators of Low Back Pain in Primary Care: Five-Year Prospective Study. *J Pain* 2013;14(8):873–83.
25. Woby SR, Roach NK, Urmston M, et al. Psychometric properties of the TSK-11: a shortened version of the Tampa Scale for Kinesiophobia. *Pain* 2005;117(1-2):137-44. doi: 10.1016/j.pain.2005.05.029
26. Miller RP KS, Todd D. The Tampa Scale: a measure of kinesiophobia. *Clin J Pain* 1991;7(1):51-52.
27. Swinkels-Meewisse EJCM, Swinkels, R.A.H.M., Verbeek, A.L.M., Vlaeyen, J.W.S. . Psychometric Propertieis of the Tampa Scale for Kinesiophobia and the Fear Avoidance Beliecs Questionnaire in Acute Low Back Pain *Manual Therapy* 2003;8(1):29-36.
28. Kall LB. Psychological determinants of quality of life in patients with whiplash associated disorders-a prospective study. *Disabil Rehabil* 2009;31(3):227-36. doi: 10.1080/09638280801912030
29. M.K. N. The pain self-efficacy questionnaire: Taking pain into account. *Eur J Pain* 2007;11(2):153-63.
30. Horowitz M, Wilner N, Alvarez W. Impact of Event Scale: a measure of subjective stress. *Psychosom Med* 1979;41(3):209-18.
31. Beck JG, Grant DM, Read JP, et al. The impact of event scale-revised: psychometric properties in a sample of motor vehicle accident survivors. *J Anxiety Disord* 2008;22(2):187-98. doi: 10.1016/j.janxdis.2007.02.007
32. Creamer M, Bell R, Failla S. Psychometric properties of the Impact of Event Scale - Revised. *Behav Res Ther* 2003;41(12):1489-96.
33. Barbero M, Mores, F., Leoni, D., Gatti, R., Egloff, M., Falla, D. Test-retest reliability of pain extent and pain location using a novel method for pain drawing analysis. *European Journal of Pain* 2015;19:1129-38.
34. Baker SP, O'Neill B. The injury severity score: an update. *J Trauma* 1976;16(11):882-5.
35. Akmal M, Trivedi R, Sutcliffe J. Functional outcome in trauma patients with spinal injury. *Spine (Phila Pa 1976)* 2003;28(2):180-5. doi: 10.1097/01.BRS.0000041577.12233.96
36. Baker SP, O'Neill B, Haddon W, Jr., et al. The injury severity score: a method for describing patients with multiple injuries and evaluating emergency care. *J Trauma* 1974;14(3):187-96.
37. Linn S. The injury severity score--importance and uses. *Ann Epidemiol* 1995;5(6):440-6.
38. Sears JM, Blanar L, Bowman SM. Predicting work-related disability and medical cost outcomes: a comparison of injury severity scoring methods. *Injury* 2014;45(1):16-22. doi: 10.1016/j.injury.2012.12.024
39. NICE. Low back pain and sciatica in over 16s: assessment and management

NICE guideline [NG59]: National Institute for Health and Care Excellence, 2016.
40. Von Korff M, Ormel J, Keefe FJ, et al. Grading the severity of chronic pain. *Pain* 1992;50(2):133-49.
41. Jensen MP, McFarland CA. Increasing the reliability and validity of pain intensity measurement in chronic pain patients. *Pain* 1993;55(2):195-203.
42. Jensen MP, Turner, J.A. and Romano, J.M. . What is the maximum number of levels needed in pain intensity measurement? . *Pain* 1994;58:387–92.
43. Jensen MP, Turner, L.R., Turner, J.A. and Romano, J.M. The use of multiple-item scales for pain intensity measurement in chronic pain patients. *Pain* 1996;67:35–40.
44. Jensen MP, Turner JA, Romano JM, et al. Comparative reliability and validity of chronic pain intensity measures. *Pain* 1999;83(2):157-62.

For peer review only - http://bmjopen.bmj.com/site/about/guidelines.xhtml

45. Von Korff M. Epidemiological and survey methods – assessment of chronic pain. In Turk, D.C. and Melzack, R., editors, Handbook of Pain Assessment. . New York, USA: Guildford Press 2001:603-18.
46. Farrar JT, Young JP, Jr., LaMoreaux L, et al. Clinical importance of changes in chronic pain intensity measured on an 11-point numerical pain rating scale. *Pain* 2001;94(2):149-58.
47. Salaffi F, Stancati A, Silvestri CA, et al. Minimal clinically important changes in chronic musculoskeletal pain intensity measured on a numerical rating scale. *Eur J Pain* 2004;8(4):283-91. doi: 10.1016/j.ejpain.2003.09.004
48. Childs JD, Piva SR, Fritz JM. Responsiveness of the numeric pain rating scale in patients with low back pain. *Spine (Phila Pa 1976)* 2005;30(11):1331-4.
49. Ostelo RW, Deyo, R.A., Stratford, P., Waddell, G., Croft, P., Von Korff, M., Bouter, L.M. and Henrica, C. Interpreting change scores for pain and functional status in low back pain: towards international consensus regarding minimal important change. *Spine* 2008;33(1):90-94.
50. Michener LA, Snyder AR, Leggin BG. Responsiveness of the numeric pain rating scale in patients with shoulder pain and the effect of surgical status. *J Sport Rehabil* 2011;20(1):115-28.
51. Masters Steedman S, Middaugh SJ, Kee WG, et al. Chronic-pain medications: equivalence levels and method of quantifying usage. *Clin J Pain* 1992;8(3):204-14.
52. Harden RN, Weinland SR, Remble TA, et al. Medication Quantification Scale Version III: update in medication classes and revised detriment weights by survey of American Pain Society Physicians. *J Pain* 2005;6(6):364-71. doi: 10.1016/j.jpain.2005.01.350
53. Gallizzi M GC, Harden RN, Stanos S, Khan A. Medication Quantification Scale Version III: internal validation of detriment weights using a chronic pain population. . *Pain Pract* 2008;8(1):1-4.
54. KJ S, WG K, S S, et al. Medication quantification scores and evaluation of patient pharmacology profiles. *Current Review of Pain* 1998;2(3):171–74.
55. Freynhagen R, Baron R, Gockel U, et al. painDETECT: a new screening questionnaire to identify neuropathic components in patients with back pain. *Curr Med Res Opin* 2006;22(10):1911-20. doi: 10.1185/030079906X132488
56. Cappelleri JC, Koduru V, Bienen EJ, et al. A cross-sectional study examining the psychometric properties of the painDETECT measure in neuropathic pain. *J Pain Res* 2015;8(8):159-67. doi: 10.2147/JPR.S80046
57. Keller T, Freynhagen R, Tolle TR, et al. A retrospective analysis of the long-term test-retest stability of pain descriptors of the painDETECT questionnaire. *Curr Med Res Opin* 2016;32(2):343-9. doi: 10.1185/03007995.2015.1125869
58. Tampin B, Bohne T, Callan M, et al. Reliability of the English version of the painDETECT questionnaire. *Curr Med Res Opin* 2017;33(4):741-48. doi: 10.1080/03007995.2017.1278682
59. Sadosky A, Koduru V, Bienen EJ, et al. Characterizing individual painDETECT symptoms by average pain severity. *Clinicoecon Outcomes Res* 2016;8:361-6. doi: 10.2147/CEOR.S105402
60. Cappelleri JC, Koduru V, Bienen EJ, et al. Mapping painDETECT, a neuropathic pain screening tool, to the EuroQol (EQ-5D-3L). *Qual Life Res* 2017;26(2):467-77. doi: 10.1007/s11136-016-1379-2
61. M OB. Aids to the Examination of the Peripheral Nervous System: Saunders (Elsevier) 2010.
62. T. Palmer DJM, Wilma M. Steedman, John Ravey, S. C-and Aδ-fibre mediated thermal perception: response to rate of temperature change using method of limits. . *Somatosensory & motor research* 2000;17(4):325-33.
63. Chesterton LS SJ, Wright CC, Foster NE. Interrater reliability of algometry in measuring pressure pain thresholds in healthy humans, using multiple raters. *Clin J Pain* 2007;23(9):760-6.
64. Knutti IA SM, Opsommer E. Test-retest reliability of thermal quantitative sensory testing on two sites within the L5 dermatome of the lumbar spine and lower extremity. *Neurosci Lett* 2014;5(579):157-62.

For peer review only - http://bmjopen.bmj.com/site/about/guidelines.xhtml

65. Greenspan JD, McGillis SL. Stimulus features relevant to the perception of sharpness and mechanically evoked cutaneous pain. *Somatosens Mot Res* 1991;8(2):137-47.
66. Sarlani E, Greenspan JD. Gender differences in temporal summation of mechanically evoked pain. *Pain* 2002;97(1-2):163-9.
67. Nie H, Arendt-Nielsen L, Andersen H, et al. Temporal summation of pain evoked by mechanical stimulation in deep and superficial tissue. *J Pain* 2005;6(6):348-55. doi: 10.1016/j.jpain.2005.01.352
68. Nie H, Arendt-Nielsen L, Madeleine P, et al. Enhanced temporal summation of pressure pain in the trapezius muscle after delayed onset muscle soreness. *Exp Brain Res* 2006;170(2):182-90. doi: 10.1007/s00221-005-0196-6
69. Whicher JT ES. Acute phase proteins. *Hosp Update* 1990:899-905.
70. Gosling P, Dickson GR. Serum c-reactive protein in patients with serious trauma. *Injury* 1992;23(7):483-6.
71. Sturmer T, Raum E, Buchner M, et al. Pain and high sensitivity C reactive protein in patients with chronic low back pain and acute sciatic pain. *Ann Rheum Dis* 2005;64(6):921-5. doi: 10.1136/ard.2004.027045
72. Pepys MB, Hirschfield GM. C-reactive protein: a critical update. *J Clin Invest* 2003;111(12):1805-12. doi: 10.1172/JCI18921
73. Giannoudis PV HF, Pape HC. . Inflammatory serum markers in patients with multiple trauma. Can they predict outcome? *J Bone Joint Surg Br* 2004;86(3):313-23.
74. Barma M, Goodbrand, J.A., Donnan, P.T., McGilchrist, M.M., Frost, H., McMurdo, M.E. and Witham, M.D. Slower Decline in C-Reactive Protein after an Inflammatory Insult Is Associated with Longer Survival in Older Hospitalised Patients. . *PloS one* 2016;11(7):e0159412.
75. Krysko DV, Agostinis P, Krysko O, et al. Emerging role of damage-associated molecular patterns derived from mitochondria in inflammation. *Trends Immunol* 2011;32(4):157-64. doi: 10.1016/j.it.2011.01.005
76. Simmons JD, Lee YL, Mulekar S, et al. Elevated levels of plasma mitochondrial DNA DAMPs are linked to clinical outcome in severely injured human subjects. *Ann Surg* 2013;258(4):591-6; discussion 96-8. doi: 10.1097/SLA.0b013e3182a4ea46
77. Yamanouchi S, Kudo D, Yamada M, et al. Plasma mitochondrial DNA levels in patients with trauma and severe sepsis: time course and the association with clinical status. *J Crit Care* 2013;28(6):1027-31. doi: 10.1016/j.jcrc.2013.05.006
78. MA F. Steroids and immunity from injury through to rehabilitation (SIR Study). University of Birmingham, 2013.

For peer review only

For peer review only - http://bmjopen.bmj.com/site/about/guidelines.xhtml

STANDARD PROTOCOL ITEMS: RECOMMENDATIONS FOR INTERVENTIONAL TRIALS

SPIRIT 2013 Checklist: Recommended items to address in a clinical trial protocol and related documents*

Section/item	Item No	Description
**Administrative information**		
Title	1	Descriptive title identifying the study design, population, interventions, and, if applicable, trial acronym – Page 1
Trial registration	2a	Trial identifier and registry name. If not yet registered, name of intended registry – N/A
	2b	All items from the World Health Organization Trial Registration Data Set – N/A
Protocol version	3	Date and version identifier – Page 1
Funding	4	Sources and types of financial, material, and other support – Page 25
Roles and responsibilities	5a	Names, affiliations, and roles of protocol contributors – Pages 1, 25
	5b	Name and contact information for the trial sponsor – Page 25
	5c	Role of study sponsor and funders, if any, in study design; collection, management, analysis, and interpretation of data; writing of the report; and the decision to submit the report for publication, including whether they will have ultimate authority over any of these activities – Page 25
	5d	Composition, roles, and responsibilities of the coordinating centre, steering committee, endpoint adjudication committee, data management team, and other individuals or groups overseeing the trial, if applicable (see Item 21a for data monitoring committee) – Page 25
**Introduction**		
Background and rationale	6a	Description of research question and justification for undertaking the trial, including summary of relevant studies (published and unpublished) examining benefits and harms for each intervention – Page 5 (introduction, page 6 (aims and objectives)
	6b	Explanation for choice of comparators – Supplementary file
Objectives	7	Specific objectives or hypotheses (Page 6)

For peer review only

Trial design	8	Description of trial design including type of trial (eg, parallel group, crossover, factorial, single group), allocation ratio, and framework (eg, superiority, equivalence, noninferiority, exploratory) – Page 7

Methods: Participants, interventions, and outcomes

Study setting	9	Description of study settings (eg, community clinic, academic hospital) and list of countries where data will be collected. Reference to where list of study sites can be obtained – Page 7
Eligibility criteria	10	Inclusion and exclusion criteria for participants. If applicable, eligibility criteria for study centres and individuals who will perform the interventions (eg, surgeons, psychotherapists) – Pages 8-10
Interventions	11a	Interventions for each group with sufficient detail to allow replication, including how and when they will be administered – N/A
	11b	Criteria for discontinuing or modifying allocated interventions for a given trial participant (eg, drug dose change in response to harms, participant request, or improving/worsening disease) – N/A
	11c	Strategies to improve adherence to intervention protocols, and any procedures for monitoring adherence (eg, drug tablet return, laboratory tests) – N/A
	11d	Relevant concomitant care and interventions that are permitted or prohibited during the trial – N/A
Outcomes	12	Primary, secondary, and other outcomes, including the specific measurement variable (eg, systolic blood pressure), analysis metric (eg, change from baseline, final value, time to event), method of aggregation (eg, median, proportion), and time point for each outcome. Explanation of the clinical relevance of chosen efficacy and harm outcomes is strongly recommended – Page 10 (primary outcome), Supplementary file (candidate predictors)
Participant timeline	13	Time schedule of enrolment, interventions (including any run-ins and washouts), assessments, and visits for participants. A schematic diagram is highly recommended (see Figure) – Pages 8-10
Sample size	14	Estimated number of participants needed to achieve study objectives and how it was determined, including clinical and statistical assumptions supporting any sample size calculations – Pages 14-15
Recruitment	15	Strategies for achieving adequate participant enrolment to reach target sample size – Pages 14-15

Methods: Assignment of interventions (for controlled trials)

Allocation:

Sequence generation	16a	Method of generating the allocation sequence (eg, computer-generated random numbers), and list of any factors for stratification. To reduce predictability of a random sequence, details of any planned restriction (eg, blocking) should be provided in a separate document that is unavailable to those who enrol participants or assign interventions – N/A
Allocation concealment mechanism	16b	Mechanism of implementing the allocation sequence (eg, central telephone; sequentially numbered, opaque, sealed envelopes), describing any steps to conceal the sequence until interventions are assigned – N/A
Implementation	16c	Who will generate the allocation sequence, who will enrol participants, and who will assign participants to interventions – N/A
Blinding (masking)	17a	Who will be blinded after assignment to interventions (eg, trial participants, care providers, outcome assessors, data analysts), and how N/A
	17b	If blinded, circumstances under which unblinding is permissible, and procedure for revealing a participant's allocated intervention during the trial – N/A
**Methods: Data collection, management, and analysis**		
Data collection methods	18a	Plans for assessment and collection of outcome, baseline, and other trial data, including any related processes to promote data quality (eg, duplicate measurements, training of assessors) and a description of study instruments (eg, questionnaires, laboratory tests) along with their reliability and validity, if known. Reference to where data collection forms can be found, if not in the protocol – Pages 10-14, Supplementary file
	18b	Plans to promote participant retention and complete follow-up, including list of any outcome data to be collected for participants who discontinue or deviate from intervention protocols – Page 9 (withdrawals)
Data management	19	Plans for data entry, coding, security, and storage, including any related processes to promote data quality (eg, double data entry; range checks for data values). Reference to where details of data management procedures can be found, if not in the protocol – Page 14
Statistical methods	20a	Statistical methods for analysing primary and secondary outcomes. Reference to where other details of the statistical analysis plan can be found, if not in the protocol – Page 15-16
	20b	Methods for any additional analyses (eg, subgroup and adjusted analyses) – Page 16 (screening tool development and validation)

	20c	Definition of analysis population relating to protocol non-adherence (eg, as randomised analysis), and any statistical methods to handle missing data (eg, multiple imputation) – Page 15 (imputation)
**Methods: Monitoring**		
Data monitoring	21a	Composition of data monitoring committee (DMC); summary of its role and reporting structure; statement of whether it is independent from the sponsor and competing interests; and reference to where further details about its charter can be found, if not in the protocol. Alternatively, an explanation of why a DMC is not needed – N/A
	21b	Description of any interim analyses and stopping guidelines, including who will have access to these interim results and make the final decision to terminate the trial – N/A
Harms	22	Plans for collecting, assessing, reporting, and managing solicited and spontaneously reported adverse events and other unintended effects of trial interventions or trial conduct – N/A
Auditing	23	Frequency and procedures for auditing trial conduct, if any, and whether the process will be independent from investigators and the sponsor – N/A
**Ethics and dissemination**		
Research ethics approval	24	Plans for seeking research ethics committee/institutional review board (REC/IRB) approval – Pages 19-20
Protocol amendments	25	Plans for communicating important protocol modifications (eg, changes to eligibility criteria, outcomes, analyses) to relevant parties (eg, investigators, REC/IRBs, trial participants, trial registries, journals, regulators) – N/A
Consent or assent	26a	Who will obtain informed consent or assent from potential trial participants or authorised surrogates, and how (see Item 32) – Pages 9-10
	26b	Additional consent provisions for collection and use of participant data and biological specimens in ancillary studies, if applicable – N/A
Confidentiality	27	How personal information about potential and enrolled participants will be collected, shared, and maintained in order to protect confidentiality before, during, and after the trial – Page 14
Declaration of interests	28	Financial and other competing interests for principal investigators for the overall trial and each study site – Page 25
Access to data	29	Statement of who will have access to the final trial dataset, and disclosure of contractual agreements that limit such access for investigators – Not present

For peer review only

Ancillary and post-trial care	30	Provisions, if any, for ancillary and post-trial care, and for compensation to those who suffer harm from trial participation – N/A
Dissemination policy	31a	Plans for investigators and sponsor to communicate trial results to participants, healthcare professionals, the public, and other relevant groups (eg, via publication, reporting in results databases, or other data sharing arrangements), including any publication restrictions – N/A
	31b	Authorship eligibility guidelines and any intended use of professional writers – N/A
	31c	Plans, if any, for granting public access to the full protocol, participant-level dataset, and statistical code – N/A
**Appendices**		
Informed consent materials	32	Model consent form and other related documentation given to participants and authorised surrogates – N/A
Biological specimens	33	Plans for collection, laboratory evaluation, and storage of biological specimens for genetic or molecular analysis in the current trial and for future use in ancillary studies, if applicable – N/A

*It is strongly recommended that this checklist be read in conjunction with the SPIRIT 2013 Explanation & Elaboration for important clarification on the items. Amendments to the protocol should be tracked and dated. The SPIRIT checklist is copyrighted by the SPIRIT Group under the Creative Commons "Attribution-NonCommercial-NoDerivs 3.0 Unported" license.

For peer review only

BMJ Open

DEVELOPMENT OF A SCREENING TOOL TO PREDICT THE RISK OF CHRONIC PAIN AND DISABILITY FOLLOWING MUSCULOSKELETAL TRAUMA: PROTOCOL FOR A PROSPECTIVE OBSERVATIONAL STUDY

Journal:	*BMJ Open*
Manuscript ID	bmjopen-2017-017876.R2
Article Type:	Protocol
Date Submitted by the Author:	20-Dec-2017
Complete List of Authors:	Rushton, Alison; University of Birmingham, School of Sport, Exercise and Rehabilitation Sciences<br>Evans, David; University of Birmingham, School of Sport, Exercise and Rehabilitation Sciences<br>Middlebrook, Nicola; University of Birmingham, School of Sport, Exercise and Rehabilitation Sciences<br>Heneghan, Nicola; University of Birmingham, School of Sport, Exercise and Rehabilitation Sciences<br>Small, Charlotte; University of Birmingham, NIHR Surgical Reconstruction & Microbiology Research Centre<br>Lord, Janet; University of Birmingham, NIHR Surgical Reconstruction & Microbiology Research Centre<br>Patel, Jaimin; University of Birmingham, NIHR Surgical Reconstruction & Microbiology Research Centre<br>Falla, Deborah; University of Birmingham, School of Sport, Exercise and Rehabilitation Sciences
**Primary Subject Heading**:	Rehabilitation medicine
Secondary Subject Heading:	Medical management
Keywords:	Musculoskeletal trauma, Precision rehabilitation, Pain mechanisms

SCHOLARONE™ Manuscripts

TITLE PAGE

DEVELOPMENT OF A SCREENING TOOL TO PREDICT THE RISK OF CHRONIC PAIN AND DISABILITY FOLLOWING MUSCULOSKELETAL TRAUMA: PROTOCOL FOR A PROSPECTIVE OBSERVATIONAL STUDY

Rushton A[1,2], Evans DW[1,2], Middlebrook N[1,2], Heneghan N[1], Small C[2], Lord J[2], Patel JM[2], Falla D[1,2].

Affiliations

[1] Centre of Precision Rehabilitation for Spinal Pain
School of Sport, Exercise and Rehabilitation Sciences
College of Life and Environmental Sciences
University of Birmingham
Edgbaston
Birmingham
B15 2TT
United Kingdom

[2] NIHR Surgical Reconstruction & Microbiology Research Centre
University of Birmingham
Edgbaston
Birmingham
B15 2TT
United Kingdom

Correspondence:

Professor Deborah Falla
Centre of Precision Rehabilitation for Spinal Pain
School of Sport, Exercise and Rehabilitation Sciences,
College of Life and Environmental Sciences,
University of Birmingham,
Birmingham, B15 2TT, UK.
Tel: +44 121 415 4220
Email: d.falla@bham.ac.uk

For peer review only

For peer review only - http://bmjopen.bmj.com/site/about/guidelines.xhtml

ABSTRACT

Introduction

Pain is an expected and appropriate experience following traumatic musculoskeletal injury. By contrast, chronic pain and disability are unhelpful yet common sequelae of trauma-related injuries. Presently, the mechanisms that underlie the transition from acute to chronic disabling post-traumatic pain are not fully understood. Such knowledge would facilitate the development and implementation of precision rehabilitation approaches that match interventions to projected risk of recovery, with the aim of preventing poor long-term outcomes. The aim of this study is to identify a set of predictive factors to identify patients at risk of developing ongoing post-traumatic pain and disability following acute musculoskeletal trauma. To achieve this, we will use a unique and comprehensive combination of patient-reported outcome measures, psychophysical testing and biomarkers.

Methods/analysis

A prospective observational study will recruit two temporally staggered cohorts [n=250 each cohort; at least 10 cases per candidate predictor] of consecutive acute musculoskeletal trauma patients aged ≥16 years, who are emergency admissions into a Major Trauma Centre in the United Kingdom, with an episode inception defined as the traumatic event. The first cohort will identify candidate predictors to develop a screening tool to predict development of chronic and disabling pain, and the second will allow evaluation of the predictive performance of the tool [validation]. The outcome being predicted is an individual's absolute risk of poor outcome measured at 6 months follow-up using the Chronic Pain Grade Scale [poor outcome ≥Grade II]. Candidate predictors encompass the four primary mechanisms of pain: *nociceptive* [e.g. injury location], *neuropathic* [e.g. painDETECT], *inflammatory* [biomarkers], and *nociplastic* [e.g. quantitative sensory testing]. Concurrently, patient-

For peer review only - http://bmjopen.bmj.com/site/about/guidelines.xhtml

reported outcome measures will assess general health and psychosocial factors [e.g. pain self-efficacy]. Risk of poor outcome will be calculated using multiple variable regression analysis.

Ethics and dissemination

NHS Research Ethics Committee and institutional R&D approval are in progress.

Keywords

Keywords: Pain mechanisms, Prediction, Precision rehabilitation, Musculoskeletal trauma

Funding: NIHR SRMRC

Word count

4228 [excluding tables and references]

For peer review only

For peer review only - http://bmjopen.bmj.com/site/about/guidelines.xhtml

STRENGTHS AND LIMITATIONS OF THIS STUDY

- A comprehensive array of candidate predictive factors will allow for the prediction of chronic and disabling pain following trauma
- These predictive factors will enable the development and validation of a predictive tool to predict good and poor outcome at 6 months post-injury
- The prospective design of the study enables control of unwarranted influences, and enables a stronger case for inferring causal relationships
- Identifying predictive factors related to poor outcome of pain and disability outcome will facilitate targeting of effective interventions
- Other candidate predictors may have been useful to include [e.g. vibration thresholds], but consideration of burden to participants of testing and sample size considerations necessitated prioritisation of candidate predictive factors.

For peer review only

For peer review only - http://bmjopen.bmj.com/site/about/guidelines.xhtml

INTRODUCTION

Pain is an expected and appropriate experience that usually follows traumatic injury.[1] By contrast, chronic pain and disability are unhelpful but common sequelae of trauma-related injuries.[2] Gaining an understanding of why some people develop chronic and disabling post-traumatic pain is therefore a priority for individual patients, the military and society at large. Notwithstanding, the mechanisms that underlie the transition from acute to chronic disabling post-traumatic pain are not fully understood. Such knowledge would facilitate the development and implementation of a clinical pathway of care that matches interventions to projected risk of poor recovery, with the aim of preventing poor long-term outcomes. This project stems from advances in knowledge relating to the assessment and management of pain[3] and the quantification of potential predictive factors to inform personalised rehabilitation; identifying which patients to target with rehabilitation and when and how to target them.

Few studies have specifically explored predictive factors for recovery, whether poor or good, following physical trauma. Of those that have, psychosocial variables such as anxiety, depression and post-traumatic stress, have so far been identified as the strongest predictors of outcome.[4-7] However, only a limited number of variables have hitherto been evaluated as potential predictive factors. Indeed, current opinion regarding pain mechanisms[8] suggests that the development of chronic pain and disability cannot be entirely attributable to psychosocial factors. This is consistent with research in primary care that has identified predictive factors for poor outcome across a range of musculoskeletal pain conditions[9], which include: widespread pain, high functional disability, high pain intensity, long pain duration,

For peer review only - http://bmjopen.bmj.com/site/about/guidelines.xhtml

high depression/anxiety, presence of previous pain episodes, movement restriction, and poor coping strategies. Moreover, developments in the mechanistic understanding of pain[10-12] suggest that other measures [e.g. indicators of central sensitisation, inflammatory activity] may have potential predictive utility, especially in an acute trauma population.

Aims of study

Using a unique combination of: 1) general patient characteristics including premorbid neuropsychological status, 2) quality of life and physical functioning, 3) psychosocial features, 4) injury characteristics, 5) pain characteristics, 6) quantitative sensory testing, and 7) biomarkers; we aim to find a set of predictive factors to identify patients at risk of developing ongoing post-traumatic pain and disability following acute musculoskeletal trauma. This will subsequently inform the feasibility of developing and evaluating a new clinical care pathway of precision rehabilitation that matches interventions to the predicted risk of poor recovery.

Objectives

1) Identify predictive factors for poor outcome [chronic pain and disability at 6-months post-injury] following acute musculoskeletal trauma.
2) Develop a predictive model to inform a screening tool to identify the predicted risk of poor recovery [transition from acute post-traumatic pain to chronic pain and disability].
3) Estimate the predictive performance of the screening tool through validation of the model in a separate data set.

For peer review only - http://bmjopen.bmj.com/site/about/guidelines.xhtml

For peer review only

4) Document the clinical course of symptoms at 3 and 12 months following acute musculoskeletal trauma.

METHODS AND ANALYSIS

Source of data

The study will be a prospective, observational study of two temporally staggered cohorts of trauma patients, who are emergency department admissions into a Major Trauma Centre in the United Kingdom, with an episode inception defined as the traumatic event [Figure 1]. The first cohort will facilitate development of the prediction model to inform the screening tool, and the second will enable validation of the prediction model through evaluation of the predictive performance of the model and tool.[13 14] There will be an interval of at least 6 months between recruitment into the two respective cohorts. The prospective design enables control of unwarranted influences, and enables a stronger case for inferring causal relationships. The nature of the study necessitates predictive statistical modelling.[15] This protocol is written in line with the TRIPOD statement,[16] in which recommendations are given for the reporting of prediction model development and validation.

Self-reported and physical assessment predictive data will be collected at baseline over a period of up to 14 days [or duration of inpatient stay if shorter], which will commence immediately following recruitment. Biomarker data collection will occur throughout the same baseline period, but can commence prior to recruitment providing assent is gained from a legal consultee. The outcome data will be collected at 6-months post-injury [working definition of chronic pain];[17] ; the point of evaluation of an individual's absolute risk of poor

For peer review only - http://bmjopen.bmj.com/site/about/guidelines.xhtml

outcome [objectives 1, 2 and 3]. In addition, selected data will be measured at 3 and 12-months post-injury to explore the clinical course of recovery following injury in the shorter and longer term [objective 4].

Figure 1. Study design

[Insert Figure 1 here].

Participants

Participants will be recruited from the register of a Major Trauma Centre in the United Kingdom for a period of up to 24 months [planned start date January 2018]. All consecutive eligible patients will be approached for recruitment until the sample size is achieved.

Eligibility criteria

Inclusion criteria: Adult patients aged ≥16 years who are emergency department admissions into the Major Trauma Centre, with their main criteria for admission being acute musculoskeletal trauma within the preceding 14 days, and a capacity to use and understand written and verbal English language and a mental capacity to provide informed consent [e.g. no confusion, delirium, severe cognitive impairment, or severe mental illness, defined by a score of 6 or less on the Abbreviated Mental Test][18]. The primary reason for including patients with trauma occurring up to 14 days previously is to be inclusive of patients who are critically ill and/or with diminished mental capacity initially following their trauma, and

For peer review only - http://bmjopen.bmj.com/site/about/guidelines.xhtml

patients requiring surgery as a result of the trauma; representative of the broad trauma population.

Exclusion criteria: Exclusions will be made where the patient has an acute intra-cranial lesion [e.g. bleed] combined with a score of ≤14 on the Glasgow Coma Scale [19] [a 15-item measure of consciousness impairment with adequate reliability[20] that is routinely taken in trauma patients at hospital admission], where there is evident brain or central nervous system injury or impairment, long-term neuro-cognitive disorders [such as brain tumour, multiple sclerosis, Alzheimer's and Parkinson's diseases, etc.], comorbid cancer, the presence of an ongoing rheumatological condition, prolonged use of corticosteroids, or terminal illness with short life expectancy.

Withdrawals: Participants will be informed that they are free to withdraw from the study at any time, without needing to provide reason. In the event of death within 3 months of being recruited, participants will be automatically withdrawn from the study and the primary predictive analysis. Baseline data of all withdrawn participants will be kept and compared to those of retained participants to assess for any differences.

Recruitment

Based on feasibility data [site data from the Trauma Audit and Research Network], it is estimated that at least 1,000 eligible trauma patients will be approachable for recruitment over a 24-month period, and that 50% would be expected to consent to participation. A dedicated team of research nurses will be available to recruit patients 7 days per week [from 0700 to 1930].

For peer review only - http://bmjopen.bmj.com/site/about/guidelines.xhtml

Because of impairments resulting from their injuries, some otherwise eligible patients will lack the mental capacity to provide informed consent when first approached to enrol in the study. Recruitment into the study will therefore be undertaken under the guidance and provision of the [UK] Mental Capacity Act 2005 for research in emergency situations. If the patient lacks sufficient capacity to consent, written assent for study participation will be sought from a legal consultee to begin biomarker data collection [blood samples], with informed consent for full recruitment [and subsequent data collection] being sought from the patient only if, and when, they regain sufficient capacity to provide this. If the patient does not regain capacity to provide consent within 14 days of their trauma, they will not be recruited into the study, biomarker data collection will cease, and any blood samples already collected will be destroyed before analysis.

Once informed consent is gained and the participant recruited, following a minimum 1 hour lead time after the informed consent process [to reduce patient burden], members of the research team will visit the patient at their bedside to collect baseline self-reported data via questionnaires [Table 1]. On the next available working day following completion of the questionnaires [again, to reduce patient burden], members of the study team will return to the patient to conduct the first physical [quantitative sensory testing] assessment. At each visit, if deemed necessary the capacity of the participant will be checked using an Abbreviated Mental Test[18] [a score of 6 or lower is indicative of reduced capacity], and asked if they are happy to proceed with data collection.

Outcome

For peer review only - http://bmjopen.bmj.com/site/about/guidelines.xhtml

The outcome for the prediction model is an individual's absolute risk of poor outcome [chronic pain and disability] at 6 months post-injury. Outcome will be measured using the Chronic Pain Grade Scale [CPGS],[21] which combines pain intensity and pain-related disability over the preceding 6-months into a single measure of pain *severity*. The CPGS has previously been used to assess the severity of body-wide chronic pain in general population,[22] primary care[23] and post-trauma[24] populations. Each item of the CPGS relates to at least one of the three categories of the International Classification of Functioning, Disability and Health [ICF] [25]: impairment, activity limitations and restricted participation. Furthermore, all ICF categories are encompassed by the CPGS.[26] The CPGS is a unidimensional scale, with good internal consistency across different pain populations; Cronbach's alpha of 0.84 to 0.91 in back pain, 0.79 for headache, and 0.84 for temporomandibular pain.[21 27] With regards to construct validity, cross-sectional and longitudinal studies of general practice patients have shown high scores on the CPGS, indicating greater chronic pain, to be associated with higher rates of unemployment, greater pain impact scale scores, greater use of opioid analgesics and physician visits, depressed mood, and lower self-rated health status.[21 27 28] For convergent validity, the CPGS has been found to have good correlation with equivalent dimensions of the SF-36.[27 28] In terms of responsiveness, changes in score over time in patients with chronic musculoskeletal pain correlated significantly with changes in SF-36 scores.[29] The CPGS has also been shown to have good test-retest reliability in primary care patients with back pain [weighted kappa 0.81, 95% CI 0.65, 0.98].[27]

Although pain persistence is not used in assigning pain grade, a measure of pain days in the prior 6-months is included in the CPGS.[30] The responses on the remaining 7-items are used for computing scores for the 3 subscales of the CPGS:[21] characteristic pain intensity, disability score, and disability points. The characteristic pain intensity score [range: 0-100] is obtained by calculating the mean of 3 pain intensity measurements: 'at the present time', the

For peer review only - http://bmjopen.bmj.com/site/about/guidelines.xhtml

'worst pain' in the preceding 6 months, and the 'average' pain over the preceding 6 months. The disability score [range: 0-100] is obtained through the mean ratings of how much the pain has interfered in performing activities of daily living, recreational, social and family activities, and work [including housework] activities in the last 6-months. The disability points are scored 0-3 and are derived from a combination of ranked categories of the number of disability days [the number of days that the respondent was away from usual activities in the preceding 6 months due to pain] and disability score. Based on these scores, the participant's chronic pain and disability status can then be classified into one of the 5 ordinal categories of chronic pain severity:[21] no pain [Grade 0], low disability and low intensity pain [Grade I], low disability and high intensity pain [Grade II], high disability and moderately limiting intensity pain [Grade III], and high disability and severely limiting intensity pain [Grade IV]. As in previous studies, poor outcome will be defined as Grade ≥II.[23 31-34]

Candidate predictors

Candidate predictors have been selected that are: [1] reliable and valid measures of their domain, and [2] have a theoretical association with the development of chronic pain. Both modifiable and non-modifiable candidate predictors are included. Candidate predictors are summarised in Table 1, with further detail in Supplementary file S1. Table 1 details important data that will be measured at 3, 6 and 12-months post-injury to explore the clinical course of recovery following injury in the shorter and longer term. All data collection will be standardised through protocols and clinical report forms.

Table 1: Summary of data collection at different assessment points

**Domain / Candidate predictor**	**Measure / data item**	**Baseline** *Commencing ≤14 days*	**3 months** *Clinical course*	**6 months** *Outcome assessment*	**12 months** *Clinical course*

For peer review only - http://bmjopen.bmj.com/site/about/guidelines.xhtml

		post-trauma		point / clinical course	
**General patient characteristics including premorbid neuropsychological status**					
Age	In years	√			
Gender	Female / male / other	√			
Body Mass Index [BMI]	Calculated from height and weight measurements	√			
Education	Highest educational level attained	√			
Employment status	Full-time/ part-time / not working / retired / student Employed / self-employed	√	√	√	√
Circumstance of trauma	Military / civilian	√			
Previous history of musculoskeletal pain and injury	Patient history data from patient recollection and medical records	√			
Comorbidity of other health problems	Patient history data from patient recollection and medical records	√			
Premorbid physical health	Patient history data from patient recollection and medical records	√			
Premorbid psychological health	Patient history data from medical records and patient recollection [including non-somatic items from the Subjective Health Complaints Inventory][35]	√			
Number of days in hospital	Intensive care / ward / total	√			
**Quality of life and physical functioning**					
General health	36-item Short Form Health Survey, version 2 [SF-36v2][36]	√	√	√	√
Health-related quality of life	EuroQol EQ-5D-5L[37]	√	√	√	√
Self-care and mobility during activities of daily living	Barthel Index of Activities of Daily Living,[38] collected from hospital data	√			
Sleep quality	11-point [0-10] Numerical Rating Scales, relating to current pain, from 'best possible sleep' to 'worst possible sleep'[39]	√	√	√	√
Brain/CNS impairment	Glasgow Coma Scale [19]	√			
**Psychosocial features**					
Anxiety and depression	Hospital Anxiety and Depression Scales [HADS][40]	√	√	√	√
Coping strategies applied during a painful experience	Coping Strategies Questionnaire-24 [CSQ-24][41]	√	√	√	√
Fear of movement or fear of injury or re-injury during movement	Tampa Scale of Kinesiophobia, 11-item [TSK-11][42]	√	√	√	√
Confidence in ability to perform activities despite pain	Pain Self-Efficacy Questionnaire[43]	√	√	√	√
Subjective post-traumatic distress	Impact of Event Scale revised [IES-R][44]	√	√	√	√

For peer review only - http://bmjopen.bmj.com/site/about/guidelines.xhtml

**Injury characteristics**					
Time of injury/incident	Hospital record data	√			
Injury location	Adapted pain drawings, based on hospital record data	√			
Tissues damaged	Based on imaging data and hospital records<br>Fractures<br>Penetrating / non-penetrating injury / both	√			
Surgery required	Location and post-injury timing of surgery, based on hospital record data	√			
Assisted mechanical ventilation required	Yes / no / duration	√			
Severity of injury	Injury Severity Scale[45]	√			
**Pain characteristics**					
Chronic pain severity	Chronic Pain Grade Scale[21]			√	√
Pain intensity	11-point [0-10] Numerical Rating Scale, relating to current pain, from 'no pain' to 'pain as bad as could be' [collected as part of the Chronic Pain Grade Scale]	√[1]	√	√	√
Pain medication intake [type, dosage and timing]	Medication Quantification Scale,[46-48] based on hospital record data	√[1]			
Pain location	Pain drawing	√[1]	√	√	√
Pain extent	Electronic pain drawing[49]	√[1]			
Self-reported features of neuropathic pain	painDETECT questionnaire[50]	√[1]	√	√	√
**Quantitative sensory testing**					
Heat pain threshold	Evaluation of pain threshold using a heat stimulus	√[1]			
Cold pain threshold	Evaluation of pain threshold using a cold stimulus	√[1]			
Pressure pain threshold	Evaluation of pain threshold using a pressure stimulus	√[1]			
Temporal summation	Evaluation of pain intensity in response to repetitive pressure stimuli	√[1]			
**Biomarkers**					
C-reactive protein [CRP]	Serum levels of CRP, a broad indicator of inflammation [via blood analysis]	√[2]			
Cell-free DNA [cfDNA]	Plasma levels of cell-free [nuclear and mitochondrial] DNA, indicators of tissue damage [via blood analysis]	√[2]			

[1] Measurements to be taken repeatedly throughout the baseline period, which will commence immediately following recruitment via informed consent [up to 14 days post-trauma] for a period of up to 14 days [i.e. until a maximum of 28 days post-trauma], whilst the participant is in hospital

[2] Measurements to be taken repeatedly throughout the baseline period, but may be commenced prior to this, subject to assent from a legal consultee

Data handling

For peer review only - http://bmjopen.bmj.com/site/about/guidelines.xhtml

Blood samples will be collected through the clinical and research nurse teams, whilst the participant is in the hospital, and either analysed immediately [C-reactive protein] or securely stored for subsequent analysis [cell-free DNA]. Baseline self-reported questionnaires, pain and injury drawings, and physical assessments will be collected by one of three trained assessors from the study team. Inter-rater reliability studies [across 2 assessors] will first be conducted in both healthy and trauma populations to inform definitive testing protocols. The order of physical assessment data collection will be randomly assigned [using computerised randomisation software] according to the modality of testing and by site, to prevent order effects. Follow-up self-reported questionnaires will be posted to participants at their home addresses; with up to two postal reminders and a telephone call for non-response. All questionnaires will be formatted so that data can be scanned or entered directly into an electronic database. Following data entry, data will be checked by a second researcher for completeness and accuracy. In addition, regular audits of data collection and storage will be performed by an independent study management committee. Participant identifiable information will be securely stored within the hospital, in line with current United Kingdom data protection legislation, and only accessible by the site Principal Investigator and Research Nurse team who will not be involved in data analysis. All outcome measure data will be securely transferred within an anonymised database file to physically secure servers at the University of Birmingham, and stored for a period of 10-years in line with Research Governance procedures. Participants will receive usual care, and interventions received will be recorded for descriptive analysis. Anonymised data will be analysed using IBM SPSS Statistics.

Sample size

For peer review only - http://bmjopen.bmj.com/site/about/guidelines.xhtml

In predictive modelling, a larger sample size enables lower bias and variance, and permits the prospective prediction of new observations.[15] The number of predictors will be reduced using exploratory factor analysis. This process will ensure that the sample size provides at least 10 cases per candidate predictor, to adequately power the final regression analysis.[51 52] Data will be collected for an estimated 300 participants per cohort [n=600 total] to allow for withdrawals [primarily expected deaths within the first 3 months] and losses to follow-up, so that final data are available for 250 participants per cohort [n=500 total].

Statistical analysis methods and management of missing data

For each cohort, potentially eligible patients, numbers examined for eligibility, confirmed eligible, recruited into the study, completing follow-up, and analysed will be reported in a flow diagram. Reasons for non-participation, exclusion, drop-outs and withdrawal [e.g. death] will be reported at each stage. Participant characteristics will be descriptively presented. For each variable of interest, the number of participants with missing data will be reported.

For the first cohort to develop the predictive model, an initial exploratory data analysis stage will summarise the data.[15] Correlations between candidate predictive factors will be calculated at baseline. Outcome [CPGS] scores will be dichotomised into good and poor categories as described previously. Data reduction will use exploratory factor analysis to assess factor loading of candidate predictors [summary scores] on poor outcome at 6 months. This will enable the number of candidate predictors entered into the final model to be reduced to 25, which can be supported by the cohort sample of 250. This process reduces the risk of

For peer review only - http://bmjopen.bmj.com/site/about/guidelines.xhtml

over-fitting the model and the risk of selecting the wrong variables due to correlation between predictor variables [multicollinearity].[53]

Statistical modelling for prediction has been planned *a priori*. To explore the influence of each predictive factor on poor outcome at 6 months, a logistic multivariable regression model will be fitted to the dichotomised outcome scores to calculate low and high risk of poor outcome. Odds ratios for each candidate predictive factor will be reported, adjusted for other factors and account for clustering [e.g. level of injury severity]. If necessary, multiple imputation[54] will be used to deal with missing outcome data. The characteristics of those patients with and without 6-month data will also be compared, to inform whether patients with no 6-month data were missing at random. Reduced multivariable analyses will be considered if necessary [e.g. removing one of two candidate predictive factors that are highly correlated at baseline], to examine the robustness of the conclusions.

Risk groups and development of the predictive screening tool

The predictive model will be used to develop a risk stratification tool to inform an individual's absolute risk of poor outcome. The stratification tool will inform clinical decision-making for precision rehabilitation. Items will be selected for the model if they are statistically significantly [$p<0.05$] associated with poor outcome in the logistic regression analysis, and those deemed clinically important to retain using expert opinion [regardless of statistical significance, study steering group] to improve face validity for clinicians and avoid over-fitting of the model.[53] The regression model with included predictive factors will be fitted to the data from the first of the two cohorts to obtain a final set of parameter estimates

[i.e. alpha and beta terms], which will be used to form the model. An important requirement of the stratification tool is that it should be sufficiently brief to facilitate use in clinical practice. Thus, we will look to simplify the model where possible to facilitate its use, but without important reduction in its predictive ability in terms of calibration and discrimination. For example, if multi-item questionnaire scores are included in the model, then we will evaluate whether just one of the questionnaire items is sufficient. Ideally, this process will result in a full and simplified model.

Development versus validation

For validation of the model, data from the second of the two cohorts will be compared to that of the first to enable analysis of the distribution of important variables; inclusive of demographic, predictor and outcome variables. The predictive performance of the screening tool [discrimination, calibration, and goodness of fit] will be assessed using data from the second cohort. Data in both cohorts will be consistent in terms of setting, eligibility criteria, outcome, and predictors.

DISCUSSION

There is an urgent need for a robust predictive study to predict the transition from acute to chronic pain in a musculoskeletal trauma population. Using such a comprehensive array of outcome measures will allow the development and validation of a predictive tool to predict development of chronic and disabling pain, and begin the process of identifying appropriate and precision interventions.

For peer review only - http://bmjopen.bmj.com/site/about/guidelines.xhtml

The candidate predictors used in this study have been chosen to be as comprehensive as possible, based on current knowledge of pain science. Other candidate predictors were considered [e.g. microRNA biomarkers], but their mechanistic functions and temporal progression are not yet sufficiently clear to justify the expense of their inclusion. The combination of patient reported outcome measures, psychophysical testing and biomarkers that are included are designed to act as surrogates for the four primary mechanisms of pain: [55 56 8] *nociceptive* [injury location, severity and characteristics], *neuropathic* [painDETECT tool and pain extent, *inflammatory* [biomarkers], and *nociplastic* [quantitative sensory testing, painDETECT and pain location and extent]. In addition, other patient-reported outcome measures [e.g. pain intensity, post-traumatic stress, anxiety and depression, coping, and pain self-efficacy] are included as the domains that they measure have been shown to influence prognosis for long-term outcomes in populations with pain in a range of locations.[9 23 24]

Rehabilitation is widely regarded as an important component of post-trauma healthcare;[57] however, the current position of equipoise means that precision rehabilitation has not yet been identified. Understanding mechanisms that underlie the transition from acute to chronic pain is essential to moving beyond this position. Identifying predictive factors related to poor outcome of pain and disability outcome will facilitate targeting of effective interventions. This will inform rehabilitation decision making, and facilitate improvements in clinical and cost effectiveness of rehabilitation interventions.

Limited research has identified criteria for quality in a predictive model, but authors have identified potential quality issues to ensure methodological rigour.[58] These issues are summarised in Table 2 and incorporated into the study design to ensure low risk of bias in development and validation of the predictive model.

For peer review only - http://bmjopen.bmj.com/site/about/guidelines.xhtml

Table 2: Methodological decisions to improve study quality

Criteria [58]	Methodological decisions to improve quality
**Study design**	
Inception cohort	• Clear description of population<br>• Clear description of the participants at baseline
Source population	• Clear description of population<br>• Clear description of sampling frame and recruitment [method and timing]
Inclusion and exclusion criteria	• Clarity of eligibility criteria
Prospective design	• Clarity of study design
**Study attrition**	
Number of drop-outs	• Adequate participation rate<br>• Clear description of attempts to collect information on participants who dropped out<br>• Reporting numbers and reasons for loss to follow-up
Information provided on method of management of missing data	• Appropriate methods of imputation of missing data
**Predictive factors**	
All predictive factors described used to develop the model	• Clear definition of predictive factors<br>• An adequate proportion of participants has complete data for the predictive factor
Standardised or valid measurements	• The measurement of the predictive factor is reliable and valid<br>• The measurement of the predictive factor is the same for all participants
Linearity assumption studied	• Linearity of data will be reported
No dichotomization of predictive variables	• Continuous variables will be reported where possible
Data presentation all predictive factors	• Complete data will be presented
**Outcome measures**	
Description of outcome measures	• The outcome is clearly defined
Standardised or valid measurements	• The measurement of the outcome is reliable and valid<br>• The measurement of the outcome is the same for all participants
Data presentation of most important outcome measures	• Complete data will be presented
**Analysis**	
Presentation of univariate crude estimates	• An appropriate strategy for model building is described<br>• An adequate statistical model described
Sufficient numbers of subjects per variable	• Adequate data will be presented
Selection method of variables explained	• Sufficient data will be presented to enable assessment of the adequacy of the analytic strategy

For peer review only

	• All results will be reported
Presentation of multivariate estimates	• An appropriate strategy for model building is described<br>• An adequate statistical model described
**Clinical performance / validity**	
Clinical performance	• Clinical performance of the model will be reported
Internal validation	• Internal validation will be reported
External validation	Not a focus of this study

ETHICS AND DISSEMINATION

Ethical approval will be obtained from the NHS Research Ethics Committee, and institutional R&D approval will also be obtained.

Patient burden and potential distress

The primary ethical concern is to limit distress on participants. As such, to reduce the patient burden when collecting baseline data, the self-reported questionnaires will be administered by members of the study team shortly following obtaining fully informed consent, and physical assessment outcomes will be measured at least 24 hours later. Patients will be asked to consent to not only providing new data for the study, but also for the study team to access information that will have been routinely collected by the hospital staff since the time of admission [e.g. nature and circumstances of injury, previous medical history, medication details, blood test results]. This will be fully explained to patients and explicitly detailed in the participant information sheet.

Mental capacity

For peer review only - http://bmjopen.bmj.com/site/about/guidelines.xhtml

Because of the nature of their injuries, the patient's mental capacity will be assessed on admission into hospital and thereafter by clinical staff and/or research nurses. The mental capacity of eligible patients at the time of being approached for recruitment will therefore fall into one of two groups: either they possess or are lacking mental capacity [in accordance with the Mental Capacity Act 2005] to provide informed consent to voluntarily participate in the study.

For patients possessing mental capacity to provide consent, a research nurse or member of the research team will ask if they are interested in participating in the study. If they are interested, a copy of the participant information sheet will be provided [and if necessary read to them] to give them an outline of the study. Following an opportunity to seek additional information and ask questions, the patient will be asked if they wish to provide their written informed consent to participate in the study, at which point a consent form will need to be signed.

On admission to the hospital, an otherwise eligible patient may lack the mental capacity to decide whether to provide consent to participate in a research study [e.g. due to the severity of their injuries, because they are arriving intubated and ventilated, or as a side-effect of medication for their injuries]. They may or may not regain this capacity during their stay in the hospital. Due to our wish to begin measuring biomarkers as early as possible following the onset of trauma, for some otherwise eligible patients it would be necessary to take blood samples before the patient has regained the capacity to provide informed consent. Using the convention of previous studies in trauma populations,[59] recruitment into the study will be undertaken under the provision and guidance of the Mental Capacity Act 2005 for research in emergency situations, and in accordance with the Declaration of Helsinki. As

For peer review only - http://bmjopen.bmj.com/site/about/guidelines.xhtml

such, if a patient does not possess this capacity when first approached for recruitment, the research team will request a mandate to collect blood samples from a legal consultee. This mandate can continue until the patient gains sufficient capacity to make an informed decision as to whether they wish to provide consent or not. We will use this mandate up to 14 days from the date of the trauma. If the patient does not regain capacity within 14 days following the trauma, or if informed consent is not provided by the patient once capacity to do so is regained, any samples collected will be destroyed before any non-clinical biomarker analysis [i.e. cell-free DNA] is performed. Furthermore, only once informed consent has been gained from the patient would the research team proceed to collect any self-reported questionnaire or physical assessment data. The legal consultee can either be a ‘personal consultee’ e.g. family member, or a ‘nominated consultee’ e.g. intensive care consultant. Once a consultee [personal or nominated] has been identified, they will be provided with the participant information sheet, to inform them about the study. The consultee will be asked if they feel participating in the study would be something to which the patient would agree or object to. If, in their opinion, the patient would agree to participating in the study, the consultee will be asked to sign a declaration form, and the research team can begin the schedule of blood sample collections. If, at any time prior to the patient regaining capacity, the consultee decides to withdraw assent, then no further samples will be collected until the patient can be approached for formal recruitment [if appropriate].

<u>Other ethical issues</u>

Participants will be informed that they are free to withdraw from the study at any time, without needing to provide reason. At each data collection visit, the capacity of the participant will be checked [using an Abbreviated Mental Test] and asked if they are happy to

For peer review only - http://bmjopen.bmj.com/site/about/guidelines.xhtml

proceed with data collection. Any concerns for a participant by the study team will be fed back to clinical staff. All blood samples will be collected by hospital staff and the research nurse team and will be stored, tested and disposed of in accordance with current United Kingdom guidelines and regulations. In the event of death within 3 months of being recruited, participants will be automatically withdrawn from the study and the primary predictive analysis. Baseline characteristics of withdrawn participants will be compared to those of retained participants to assess for any differences.

For peer review only

For peer review only - http://bmjopen.bmj.com/site/about/guidelines.xhtml

REFERENCES

1. Berben SA, Schoonhoven L, Meijs TH, et al. Prevalence and relief of pain in trauma patients in emergency medical services. *Clin J Pain* 2011;27(7):587-92. doi: 10.1097/AJP.0b013e3182169036
2. Williamson OD, Epi GD, Gabbe BJ, et al. Predictors of moderate or severe pain 6 months after orthopaedic injury: a prospective cohort study. *J Orthop Trauma* 2009;23(2):139-44. doi: 10.1097/BOT.0b013e3181962e29
3. Mannion RJ, Woolf CJ. Pain mechanisms and management: a central perspective. *Clin J Pain* 2000;16(3 Suppl):S144-56.
4. Holbrook TL, Anderson JP, Sieber WJ, et al. Outcome after major trauma: discharge and 6-month follow-up results from the Trauma Recovery Project. *J Trauma* 1998;45(2):315-23; discussion 23-4.
5. Holbrook TL, Anderson JP, Sieber WJ, et al. Outcome after major trauma: 12-month and 18-month follow-up results from the Trauma Recovery Project. *J Trauma* 1999;46(5):765-71; discussion 71-3.
6. Harris IA, Young JM, Rae H, et al. Predictors of general health after major trauma. *J Trauma* 2008;64(4):969-74. doi: 10.1097/01.ta.0000245972.83948.1a
7. Ponsford J, Hill B, Karamitsios M, et al. Factors influencing outcome after orthopedic trauma. *J Trauma* 2008;64(4):1001-9. doi: 10.1097/TA.0b013e31809fec16
8. Vardeh D, Mannion RJ, Woolf CJ. Toward a Mechanism-Based Approach to Pain Diagnosis. *J Pain* 2016;17(9 Suppl):T50-69. doi: 10.1016/j.jpain.2016.03.001
9. Artus M, Campbell P, Mallen CD, et al. Generic prognostic factors for musculoskeletal pain in primary care: a systematic review. *BMJ Open* 2017;7(1):e012901. doi: 10.1136/bmjopen-2016-012901
10. Latremoliere A, Woolf CJ. Central sensitization: a generator of pain hypersensitivity by central neural plasticity. *J Pain* 2009;10(9):895-926. doi: 10.1016/j.jpain.2009.06.012
11. Linley JE, Rose K, Ooi L, et al. Understanding inflammatory pain: ion channels contributing to acute and chronic nociception. *Pflugers Arch* 2010;459(5):657-69. doi: 10.1007/s00424-010-0784-6
12. Woolf CJ. Central sensitization: implications for the diagnosis and treatment of pain. *Pain* 2011;152(3):S2-15.
13. Altman DG, Vergouwe Y, Royston P, et al. Prognosis and prognostic research: validating a prognostic model. *BMJ* 2009;338(338):b605. doi: 10.1136/bmj.b605
14. Moons KG, Kengne AP, Grobbee DE, et al. Risk prediction models: II. External validation, model updating, and impact assessment. *Heart* 2012;98(9):691-8. doi: 10.1136/heartjnl-2011-301247
15. Shmueli G. To explain or to predict? *Statistical Science* 2010;25(3):289-310.
16. Collins GS, Reitsma, J.B., Altman, D.G. and Moons, K.G., . Transparent reporting of a multivariable prediction model for individual prognosis or diagnosis (TRIPOD): the TRIPOD statement. *BMC Medicine* 2015;13(1)
17. Merskey H Bogduk N. Classification of chronic pain : descriptions of chronic pain syndromes and

definitions of pain terms. USA: IASP Press 1994.
18. Hodgkinson HM. Evaluation of a mental test score for assessment of mental impairment in the elderly. *Age and Ageing* 1972;1(4):233-8.
19. Teasdale G, Jennett B. Assessment of coma and impaired consciousness. A practical scale. *Lancet* 1974;2(7872):81-4.
20. Reith FC, Van den Brande R, Synnot A, et al. The reliability of the Glasgow Coma Scale: a systematic review. *Intensive Care Med* 2016;42(1):3-15. doi: 10.1007/s00134-015-4124-3

For peer review only - http://bmjopen.bmj.com/site/about/guidelines.xhtml

21. Von Korff M, Ormel J, Keefe FJ, et al. Grading the severity of chronic pain. *Pain* 1992;50(2):133-49.
22. Parsons S, Breen A, Foster NE, et al. Prevalence and comparative troublesomeness by age of musculoskeletal pain in different body locations. *Fam Pract* 2007;24(4):308-16. doi: 10.1093/fampra/cmm027
23. Muller S, Thomas E, Dunn KM, et al. A prognostic approach to defining chronic pain across a range of musculoskeletal pain sites. *Clin J Pain* 2013;29(5):411-6. doi: 10.1097/AJP.0b013e318257099e
24. Rivara FP, Mackenzie EJ, Jurkovich GJ, et al. Prevalence of pain in patients 1 year after major trauma. *Arch Surg* 2008;143(3):282-7; discussion 88. doi: 10.1001/archsurg.2007.61
25. Organization WH. The International Classification of Functioning, Disability and Health (ICF). Geneva: WHO 2001.
26. Dixon D PD, Johnston M. What does the chronic pain grade questionnaire measure? *Pain* 2007;130:249-53.
27. Smith BH, Penny KI, Purves AM, et al. The Chronic Pain Grade questionnaire: validation and reliability in postal research. *Pain* 1997;71(2):141-7.
28. Penny KI PA, Smith BH, Chambers WA, Smith WC. Relationship between the chronic pain grade and measures of physical, social and psychological well-being. *pain* 1999;79:275–9.
29. Elliott AM SB, Smith WC, Chambers WA. Changes in chronic pain severity over time: The Chronic Pain Grade as a valid measure. *Pain* 2000;88:303.
30. Von Korff M, Jensen MP, Karoly P. Assessing global pain severity by self-report in clinical and health services research. *Spine (Phila Pa 1976)* 2000;25(24):3140-51.
31. Von Korff M, Miglioretti DL. A prognostic approach to defining chronic pain. *Pain* 2005;117(3):304-13. doi: 10.1016/j.pain.2005.06.017
32. Von Korff M, Dunn KM. Chronic pain reconsidered. *Pain* 2008;138(2):267-76. doi: 10.1016/j.pain.2007.12.010
33. Dunn KM, Croft PR, Main CJ, et al. A prognostic approach to defining chronic pain: replication in a UK primary care low back pain population. *Pain* 2008;135(1-2):48-54. doi: 10.1016/j.pain.2007.05.001
34. Thomas E, Dunn KM, Mallen C, et al. A prognostic approach to defining chronic pain: application to knee pain in older adults. *Pain* 2008;139(2):389-97. doi: 10.1016/j.pain.2008.05.010
35. Eriksen HR, Ihlebaek C, Ursin H. A scoring system for subjective health complaints (SHC). *Scand J Public Health* 1999;27(1):63-72.
36. Ware J. SF-36 Health Survey Updated. *Spine* 2000;25(24):3130-39.
37. Brooks R. EuroQol: the current state of play. *Health Policy* 1996;37(1):53-72.
38. Mahoney FI, Barthel DW. Functional Evaluation: The Barthel Index. *Md State Med J* 1965;14:61-5.
39. Cappelleri JC BA, McDermott AM, Sadosky AB, Petrie CD, Martin S. Psychometric properties of a single-item scale to assess sleep quality among individuals with fibromyalgia. *Health Qual Life Outcomes* 2009;17(7):54.
40. Zigmond AS, Snaith RP. The hospital anxiety and depression scale. *Acta Psychiatr Scand* 1983;67(6):361-70.
41. Harland NJ Georgieff K. Development of the coping strategies questionnaire 24, a clinically utilitarian version of the coping strategies questionnaire. *Rehabilitation Psychology* 2003;48(4):296–300.
42. Woby SR, Roach NK, Urmston M, et al. Psychometric properties of the TSK-11: a shortened version of the Tampa Scale for Kinesiophobia. *Pain* 2005;117(1-2):137-44. doi: 10.1016/j.pain.2005.05.029
43. Nicholas MK. The pain self-efficacy questionnaire: Taking pain into account. *Eur J Pain* 2007;11(2):153-63.

For peer review only - http://bmjopen.bmj.com/site/about/guidelines.xhtml

44. Beck JG, Grant DM, Read JP, et al. The impact of event scale-revised: psychometric properties in a sample of motor vehicle accident survivors. *J Anxiety Disord* 2008;22(2):187-98. doi: 10.1016/j.janxdis.2007.02.007
45. Baker SP, O'Neill B, Haddon W, Jr., et al. The injury severity score: a method for describing patients with multiple injuries and evaluating emergency care. *J Trauma* 1974;14(3):187-96.
46. Masters Steedman S, Middaugh SJ, Kee WG, et al. Chronic-pain medications: equivalence levels and method of quantifying usage. *Clin J Pain* 1992;8(3):204-14.
47. Harden RN, Weinland SR, Remble TA, et al. Medication Quantification Scale Version III: update in medication classes and revised detriment weights by survey of American Pain Society Physicians. *J Pain* 2005;6(6):364-71. doi: 10.1016/j.jpain.2005.01.350
48. Gallizzi M GC, Harden RN, Stanos S, Khan A. Medication Quantification Scale Version III: internal validation of detriment weights using a chronic pain population. . *Pain Pract* 2008;8(1):1-4.
49. Barbero M, Mores, F., Leoni, D., Gatti, R., Egloff, M., Falla, D. Test-retest reliability of pain extent and pain location using a novel method for pain drawing analysis. *European Journal of Pain* 2015;19:1129-38.
50. Freynhagen R, Baron R, Gockel U, et al. painDETECT: a new screening questionnaire to identify neuropathic components in patients with back pain. *Curr Med Res Opin* 2006;22(10):1911-20. doi: 10.1185/030079906X132488
51. Peduzzi P, Concato J, Kemper E, et al. A simulation study of the number of events per variable in logistic regression analysis. *J Clin Epidemiol* 1996;49(12):1373-9.
52. Vittinghoff E, McCulloch CE. Relaxing the rule of ten events per variable in logistic and Cox regression. *Am J Epidemiol* 2007;165(6):710-8. doi: 10.1093/aje/kwk052
53. Sainani KL. Multivariate Regression: The Pitfalls of Automated Variable Selection. *Physical Medicine and Rehabilitation* 2013;5:791-94,.
54. Sterne JA, White IR, Carlin JB, et al. Multiple imputation for missing data in epidemiological and clinical research: potential and pitfalls. *BMJ* 2009;338:b2393. doi: 10.1136/bmj.b2393
55. Woolf CJ, Bennett GJ, Doherty M, et al. Towards a mechanism-based classification of pain? *Pain* 1998;77(3):227-9.
56. Kosek E CM, Baron R, Gebhart GF, Mico JA, Rice AS, Rief W, Sluka AK. Do we need a third mechanistic descriptor for chronic pain states? . *Pain* 2016;157(7):1382-6.
57. NICE. Rehabilitation after critical illness in adults Clinical guideline [CG83]. 2009
58. van Oort L, van den Berg T, Koes BW, et al. Preliminary state of development of prediction models for primary care physical therapy: a systematic review. *J Clin Epidemiol* 2012;65(12):1257-66. doi: 10.1016/j.jclinepi.2012.05.007
59. Hazeldine J, Naumann DN, Toman E, et al. Prehospital immune responses and development of multiple organ dysfunction syndrome following traumatic injury: A prospective cohort study. *PLoS Med* 2017;14(7):e1002338. doi: 10.1371/journal.pmed.1002338

For peer review only

Authors' contributions

DF is Chief Investigator and guarantor. DF and AR led protocol development are leading data analysis and dissemination. DE is the Research Fellow with responsibility for study management. NM is a Doctoral Researcher focused to this study. NH is the lead for Patient and Public Involvement. JP is the Principal Investigator at the Major Trauma Centre. JP and CS are clinical representatives at the Major Trauma Centre. JL is the lead for biomarker

For peer review only - http://bmjopen.bmj.com/site/about/guidelines.xhtml

evaluation. All authors will contribute to data interpretation, conclusions, and dissemination. AR drafted the initial manuscript with DF. Subsequent drafts were developed with DE. All reviewers have read, contributed to, and agreed upon the final manuscript.

Funding statement

This work was supported by the National Institute for Health Research Surgical Reconstruction and Microbiology Research Centre [NIHR SRMRC] to DF and AR.

Competing interests

There are no competing interests.

Data sharing statement

No additional data are available.

For peer review only

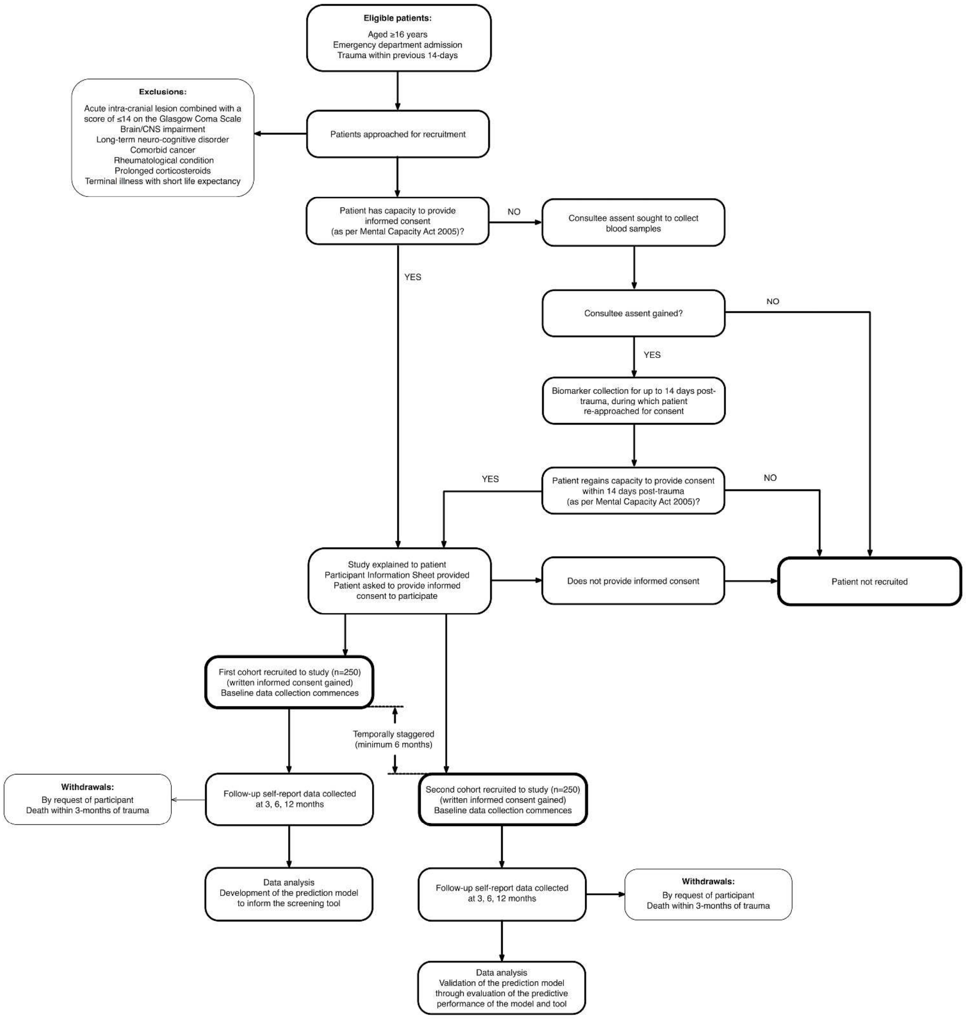

Study design

500x521mm (300 x 300 DPI)

Supplementary file 1. Candidate predictors

General participant characteristics

Several participant demographic features will be recorded at baseline, based on available hospital records and patient self-reported recollection, including smoking status, age, gender, height and weight to calculate body mass index [BMI], education [highest attained educational level], employment status [at the time of trauma], circumstance of trauma [military or civilian], previous history of musculoskeletal pain and injury, comorbidity of other current health problems.

Quality of life and physical functioning

<u>36-Item Short Form Health Survey, Version 2.0 [SF-36v2]</u>

The SF-36v2 is a self-reported measure of health-related quality of life, modified from the original SF-36, which was developed as part of the Medical Outcomes Study.[1] The 36-item questionnaire has subscales that assess physical function, social and psychological wellbeing.[2 3] The scores can be divided into physical and mental component summary scales.[4] The SF-36 has been shown to be valid and has been tested extensively in a trauma population.[5] Ware[6] reports multiple studies showing internal consistency above 0.70, with physical and mental scores exceeding 0.90. Minimal clinically important difference has been reported as 5.5 in a musculoskeletal trauma population.[7] Introduced in 1996, version 2.0 of the SF-36 is comparable to the original, retaining all subscales, with improvements to layout, presentation, response scales, wording and scoring.[6] The 'acute' [1 week recall period] version will be used, since the 4 week recall would not be appropriate for post-injury recall at baseline.

For peer review only - http://bmjopen.bmj.com/site/about/guidelines.xhtml

EuroQol Five Dimension Scale, 5-level [EQ-5D-5L]

Health-related quality of life will be quantified using the EQ-5D-5L through which 243 possible health states are converted to a single index value of range 0 to 1 where 1 is perfect health, and a visual analogue scale range 0–100, representing 'worst' to 'best' imaginable health state, respectively.[8] The EQ-5D-5L, with each item having 5 possible responses, has improved inter-observer [ICC 2,1 0.57] and test-retest [ICC 2,1 0.69] reliability compared to the previous EQ-5D-3L.[9] In addition, it has less ceiling effects [20.8% reduction] and adequate convergent validity when compared with the WHO-5 [spearman rank 0.38-0.51].[10]

Barthel Index of Activities of Daily Living

The Barthel Index of Activities of Daily Living is routinely collected by clinical staff at the hospital, and will be used to evaluate self-care and mobility during activities of daily living.[11] [12] It is a 10-item ordinal scale encompassing a range of mobility physical activity tasks. Each item is related to a specific task and rated with a given number of points. A score of '0' is given for least independence/function on that item and scores above that [1 or 2] are given for increasing independence/function [range: 0-20]. The amount of time and physical assistance required to perform each task are used in determining the assigned value of each item. A higher score is associated with a greater likelihood of being able to live at home with a degree of independence following discharge from hospital. With most measurement testing performed in the stroke population, the Barthel Index has demonstrated excellent internal consistency [0.89-0.90][13] and is highly responsive in detecting changes[14] with a minimal detectable change of 4.02 and minimally clinically important difference of 1.85.[15] High correlations have been demonstrated with the Functional Independence Measure [FIM], indicating convergent validity of the instrument.[16]

For peer review only - http://bmjopen.bmj.com/site/about/guidelines.xhtml

Subjective Health Complaints Inventory

Premorbid subjective health complaints will be assessed for the 6 months preceding the traumatic injury, using the single-item questions for non-somatic domains from the Subjective Health Complaints Inventory.[17] The Subjective Health Complaints Inventory has been shown to be a reliable measure of recording subjective health complaints for a 30 day recall period,[17] although psychometric properties have not been reported for an extended 6 month recall period.

Sleep quality

Current sleep quality [over the previous 24 hour period] will be assessed using an 11-point Numerical Rating Scale [NRS], ranging from 0 [‘best possible sleep’] to 10 [‘worst possible sleep’]. This scale has been shown to possess moderate psychometric properties in fibromyalgia patients using a symptom diary.[18] We will aim to assess current pain intensity at baseline, as frequently as every 48-hours while the patient is in hospital up to a maximum of 14 days following recruitment [depending upon patient accessibility and assessor availability], to gain accurate average and rate-of-change data. In addition, we will use the 0-10 NRS to assess average sleep quality, related to the preceding 6-months at the 6 and 12-month assessment points, although no psychometric properties have previously been reported for this recall period.

Psychosocial features

The predictive strength of psychosocial factors demonstrated in both primary care,[19 20] and post-trauma pain literature[21-24] demonstrates the importance of including these domains as candidate predictors.

For peer review only - http://bmjopen.bmj.com/site/about/guidelines.xhtml

The Hospital Anxiety and Depression Scales [HADS]

The HADS will be used to measure depression and anxiety, and their role in the manifestation of somatic symptoms.[25] There are 7 items which produce a cumulative score [range 0–21] for the anxiety [HADS-A] and depression [HADS-D] subscales, with a higher score indicative of greater anxiety and depression.[26] HADS has been tested in multiple populations demonstrating adequate to excellent internal consistency of HADS-A [0.68-0.93] and HADS-D [0.67-0.90].[26] Standard measurement of error in a coronary heart disease population was identified as 1.37 and 1.44 for anxiety and depression scales respectively, and minimal detectable change as 3.80 and 3.99 respectively.[27] The HADS has also demonstrated excellent concurrent validity when compared to various other depression/anxiety scales.[26]

Coping Strategies Questionnaire 24 [CSQ-24]

The CSQ-24 will be used to provide an indication of coping strategies used by participants when they are in pain.[28] Developed from items from the earlier, much larger Coping Strategies Questionnaire,[29] the CSQ-24 is a 23-item scale, composed of 4 subscales: *catastrophizing* [6 items], *diversion* [6 items], *reinterpreting* [6 items], and *cognitive coping* [5 items]. Participants are asked to indicate if they have particular thoughts and feelings when they are experiencing pain. A score on each item is summed to yield an aggregate score for each subscale, with a higher score reflecting greater attribution of that particular coping strategy. The CSQ-24 has demonstrated good internal consistency in populations with low back pain patients [Chronbach's alpha for the 4 factors ranged from 0.75 to 0.85] and work-related pain [0.80 to 0.86].[30] Harland & Georgieff[28] suggested that, since individuals may have a positive score on more than one subscale, the highest scoring subscale should be deemed the dominant coping strategy.[28] However, a recent study in a low back pain cohort,[31]

For peer review only - http://bmjopen.bmj.com/site/about/guidelines.xhtml

in which individual items from multiple questionnaires were factorised, suggested that *diversion*, *reinterpreting* and *cognitive coping* clustered together as a single factor, representing coping cognitions. By contrast, *catastrophizing* clustered with pain-related distress items.

Tampa Scale of Kinesiophobia [TSK-11]

The TSK-11 will be used to assess fear of movement or fear of injury or re-injury during movement.[32] It is an 11-item questionnaire, eliminating psychometrically poor items from its original 17-item version,[33] thus creating a shorter questionnaire with comparable internal consistency and a 2-factor structure [activity avoidance and harm]. Each of the 11 items is measured using a 4-point scale using the end points 1 [‘totally agree’] and 4 [‘totally disagree’] [scoring range 11–44]. Higher scores indicate more fear-avoidance behaviour. The TSK-11 has demonstrated acceptable to good internal consistency in acute and chronic musculoskeletal pain populations.[32 34] Test-retest reliability has been reported as excellent with a high standardised response mean; with good construct validity in relation to changes in disability and pain.[32]

Pain Self-Efficacy Questionnaire [PSEQ]

The patient’s confidence in their ability to perform activities despite their pain will be evaluated using the PSEQ. Developed from the Self-Efficacy Scale,[35] the PSEQ consists of 10 physical and psychosocial activity items measuring from 0 [‘not at all confident’] to 6 [‘completely confident’] thus generating a total score from 0-60.[36] The PSEQ has demonstrated excellent internal consistency [0.92], internal reliability [0.93], and test-re-test correlations [r=0.73] and has demonstrated validity when compared to other self-efficacy

For peer review only - http://bmjopen.bmj.com/site/about/guidelines.xhtml

measurements.[36] It has been used in several large population studies, for example Campbell et al.[31]

Impact of Event Scale Revised [IES-R]

The IES-R will be used to measure the subjective stress experienced by the participant following their traumatic event. The IES-R is a 22-item tool [range: 0-88] that consists of 8 intrusion and 8 avoidance items that are derived from the original IES,[37] with an additional 7-items assessing hyperarousal.[38] Accordingly, items correspond directly to symptoms of post-traumatic stress disorder.[38] Respondents are asked to identify a specific stressful life event and then indicate how much they were distressed or bothered during the past seven days by each 'difficulty' listed. Each item is rated on a 5-point scale ranging from 0 ['not at all'] to 4 ['extremely']. The IES-R yields a total score ranging 0 to 88 and subscale scores can also be calculated for the Intrusion, Avoidance, and Hyperarousal sub-scales. The IES-R has demonstrated good internal consistency for all subscales [intrusion 0.87-0.94, avoidance 0.84-0.97, hyperarousal 0.79-0.91].[39] High correlations have been found between the IES-R and the original scale, supporting the concurrent validity of both measures.[38]

Injury characteristics

Several measures relating to the characteristics of the sustained injury will be taken at baseline, since it is plausible that some of these should possess predictive value.[40] The time of the injury will be gained from hospital records. The location of the injury/injuries will be recorded using an adapted version of previously developed pain drawing software, via a tablet computer.[41] Information relating to the tissues damaged from the injury [e.g. fractures sustained, whether the injury was penetrating, non-penetrating or both, review of available imaging data] will be gathered from hospital records, where possible. Whether the participant

For peer review only - http://bmjopen.bmj.com/site/about/guidelines.xhtml

received surgery following their admission [where, for what and when], and whether the participant received assisted mechanical ventilation will also be recorded.

Injury Severity Scale [ISS]

The ISS will be retrospectively calculated for each participant, including those who withdraw. The ISS is a numerical score with a range 0-75, that is used to describe the overall severity of injury, and can be used for both multiple and single injuries. The score is calculated, based on the Abbreviated Injury Scale [AIS] scores.[42 43] Higher ISS scores have been associated with increased rates of mortality[42 44 45] and length/cost of hospital stay.[46] It is the recommended tool for summarising injury severity by the Trauma Audit and Research Network [TARN]. Both TARN and the National Institute for Clinical Excellence[47] recommend any participant with a score of >8 to be referred for rehabilitation.

Pain characteristics

Pain characteristics [e.g. pain intensity, multi-site pain] have long been reported to hold predictive value for long-term pain across a variety of conditions.[19-21 48 49] It is therefore sensible that we include these domains as candidate predictors for post-trauma pain.

Pain intensity

Pain intensity will be measured using an 11-point [0-10] Numerical Rating Scale [NRS], measuring current pain from ‘no pain’ to ‘pain as bad as could be’, from the Chronic Pain Grade Scale.[50] We will aim to assess *current* pain intensity at baseline, as frequently as every 48-hours while the participant is in hospital [depending upon participant accessibility and

For peer review only - http://bmjopen.bmj.com/site/about/guidelines.xhtml

assessor availability], to gain accurate mean and rate-of-change data. At the 6 and 12 month assessment points, c*urrent* pain intensity, as well as *average* and *worst* pain intensity related to the preceding 6 months, will be collected as part of the Chronic Pain Grade Scale. NRS scales are sensitive, reliable and valid instruments for pain intensity measurement,[51-54] and have been recommended for use in clinical populations in preference to visual analogue scales or verbal rating scales.[55] A 30% change on a pain NRS score is considered clinically meaningful.[56-60]

<u>Pain medication</u>

The patient's pain medication [type, dosage and time since trauma] intake will be noted and the Medication Quantification Score [MQS], which is a reliable and validated score for quantifying analgesics, will be calculated to obtain a comparable metric for all different analgesics.[61-63] It enables characterisation of analgesics when many different medications are involved and doses are irregular. It will be calculated for each non-opioid and opioid, based on weights assigned by medication class and dosage level [level 1 = sub-therapeutic dosage and/or on demand, level 2 = lower 50% of the therapeutic dose range, level 3 = upper 50% of the therapeutic dose range, level 4 = supra-therapeutic dose] using the 1998 detriment weights.[64] The detriment weights are summed by the dosage level to provide the final score. These scores will be summed to provide a quantitative index for analgesic usage suitable for statistical analysis.

<u>Pain drawing</u>

All participants will be requested to complete a pain drawing, indicating the spatial distribution of their pain, over two body charts; one reporting a frontal view of the body and one a dorsal view. We will also ask patients to mark their single 'most painful' site on one of

For peer review only - http://bmjopen.bmj.com/site/about/guidelines.xhtml

these body charts. Pain drawing data will be collected using a custom software application on a tablet computer, and will be analysed with Matlab software, as described previously.[41] The software automatically calculates the number of shaded pixels from the pain drawing, which is defined as *pain extent*. Summaries of, and relationships between, pre-defined painful body regions will also be evaluated. Conventional pain drawing data will also be collected on paper follow-up questionnaires to assess painful body regions.

The painDETECT questionnaire

It is assumed that all post-trauma patients will have significant nociception at baseline, but given the relatively high proportion of neuropathic pain following traumatic injury,[65] the contribution of other pain-related mechanistic pathways should also be assessed. The painDETECT questionnaire[66] will be used to facilitate the identification of neuropathic pain. It consists of 9 items [7 evaluating pain quality, 1 evaluating pain pattern, and 1 evaluating pain radiation], all of which contribute to an aggregate score [range: -1-38]. This aggregate score can be divided into three classifications that represent the likelihood of neuropathic pain: ‘unlikely’ [0-12], ‘ambiguous’ [13–18] and ‘likely’ [19–38].[66] Although developed as a screening questionnaire for neuropathic pain, painDETECT may also function as a measure of characteristics that indicate augmented central pain processing.[66] The painDETECT questionnaire has demonstrated good internal consistency [0.76][67] and excellent test-re-test reliability[68] within 1-hour of consultation [ICC{model not reported} 0.911] and 1-week post consultation [ICC{model not reported} 0.79].[69] Convergent validity has been demonstrated in comparison to pain severity,[67 70] health-related quality of life[71] and similar neuropathic pain screening tools.[66] As such, painDETECT outcomes will be measured at regular intervals while the participant is an inpatient in the hospital [subject to participant accessibility and

For peer review only - http://bmjopen.bmj.com/site/about/guidelines.xhtml

assessor availability] to assess for emerging neuropathic pain and sensitization. Measurements will also be taken at all follow-up assessment points.

Quantitative sensory testing [QST]

QST methods will be used to assess pain sensibility, throughout which measurements will be concealed from participants. Owing to the clinical heterogeneity of the post-trauma population, precise standardisation of test sites between participants will not be possible. Instead, we have developed a standardised protocol that will be used to evaluate pain thresholds for multiple stimulus modalities [mechanical pressure, heat and cold] at the same sites in each participant. Each site where multi-modality pain threshold testing is performed will be within the receptive field of the same nerve root using described regions,[72] so that segmental cross-modality excitability may be compared. All pain thresholds will be measured at the same 'local' and 'remote' sites for each participant. We define local sites as being uninjured but within [or, if not accessible, as close as possible to] the same receptive field as the most painful inured tissue [e.g. skin over gastrocnemius in a participant with an ankle fracture]. By contrast, we define remote sites as a distant, accessible, site from the receptive fields in which tissues are injured [e.g. skin over tibialis anterior in a participant without lower limb injury], and on the contralateral side of the body where injured tissue is unilateral. Where possible, remote sites will be a mirror-image of the local site [to allow for comparison of absolute values], but in a trauma population we are aware that this may not always be possible. For all threshold testing modalities, an ascending method of limits design[73] will be used, whereby stimulus intensity will begin at a low level and gradually increase until the participant first perceives pain. Participants will be instructed to push a button or tell the assessor when the sensation has changed from one of the stimulus alone [e.g. just pressure] to

For peer review only

a sensation of both the stimulus and pain [e.g. pressure and pain]. Following a brief demonstration of equipment to familiarise participants, two consecutive assessments will be performed for each modality at each site, and the means used for further analysis.[74] A minimum of 30-seconds inter-stimulus interval will be given between each threshold measurement within a single session. Measurements will be taken at baseline, while the participant is an inpatient in the hospital; we will aim to collect data as frequently as every 48 hours to gain accurate rate-of-change data, but this will depend upon participant accessibility and assessor availability. To ensure pain thresholds are consistently measured at the same sites every session, sites will be labelled using a sterile, skin marking pen [Schuco Ltd, UK]. Because sites cannot be standardised between participants, the rates-of-change of these values will be used as candidate predictive variables, to allow for comparisons between participants. The order of pain threshold testing will be randomly assigned by modality at each session to avoid order effects.

Thermal [heat and cold] pain thresholds will be measured using skin-contact stimulation, using the same thermode at the same sites, within specified local and remote dermatomes. Thermal pain threshold assessments will be performed by delivering thermal stimuli directly to the skin through a metal 30x30 mm Peltier thermode, using a TSA-II NeuroSensory Analyzer thermal stimulator and accompanying software [Medoc Ltd, Israel]. To evaluate heat pain threshold, temperature will be gradually increased, at a rate of 1°C/s from a 'neutral' baseline of 32°C, to a maximum temperature of 50.5°C to avoid thermal injury.[75] During each measurement, participants will be instructed to press a button when the stimulus becomes painful, and this will be documented as the threshold value. Once pain threshold is achieved [and recorded], the temperature will return to the baseline value at the same rate [1°C/s]. For cold pain threshold measurements, the temperature will be gradually reduced, at

For peer review only - http://bmjopen.bmj.com/site/about/guidelines.xhtml

a rate of 1°C/s from the baseline of 32°C, to a minimum temperature of 0°C,[75] before also returning to baseline at a rate of 1°C/s.

Pressure pain thresholds will be measured using a digital pressure algometer [Series 7 force gauge, Mark-10 Corporation, USA], providing real-time force measurement and an analogue output that can be linked to a computer. Skin and muscle tissue are simultaneously stimulated during pressure threshold testing; sites will therefore be chosen where a dermatome and myotome are likely to share a common nerve root innervation [e.g. skin over tibialis anterior]. The algometer has a hard rubber circular contact tip of 1.2cm^2 area, with no sharp edges so to avoid an uneven pressure stimulus.[76] In order to preserve hygiene and attend to infection control measures in trauma patients, the contact tip will be covered with a clean, thin disposable covering. The tip will be applied perpendicular to the skin at a constant rate of pressure increase of 50kPa/s [6.0N/s using the 1.2cm^2 tip], until the first onset of pain. For each measurement, pressure will be unloaded immediately once the participant indicates that their pain threshold has been reached.[74]

To measure excitability of nociceptive pathways in response to mechanical stimuli, a series of repetitive, pressure stimulus 'pulses' will be applied via the digital algometer, with the aim of provoking temporal summation responses.[77-79] A minimum of 2 minutes after all threshold tests have been completed, a series of 10 consecutive pressure pulses will be applied at the remote and local sites [the order of site being randomly assigned]. The peak pressure reached during each pulse will be the mean pressure pain threshold that was measured for that particular site, as described previously. For each pulse, pressure will be gradually increased to the peak value over a period of 5 seconds, maintained at that peak value for 1 second, and then immediately released. A 5 second inter-stimulus interval will be used between pulses,

For peer review only - http://bmjopen.bmj.com/site/about/guidelines.xhtml

during which the tip of the algometer will remain in contact with the skin.[78 79] Pain intensity from the pulses will be rated on a numerical rating scale [0 being ‘no pain’ to 10 being ‘pain as bad as could be’]. In the event that participants indicate that pain has become intolerable, the sequence will be stopped immediately, and the NRS score and number of impulses performed at that point will be noted.

Biomarkers

Serum levels of C-reactive protein [CRP] will be used as a biomarker for inflammation; one of the primary mechanistic pathways that can evoke pain.[80] CRP is an acute-phase response protein produced by hepatocytes and is usually found in concentrations of 0.3 to 1.7 mg/l[81]. Increased production is due to cytokine-dependent induction of synthesis and elevated levels may be detected within eight hours of a stimulus and can reach 500 mg/1.28. Besides trauma,[82] elevated levels of CRP may be seen in conditions such as autoimmune disease, infection and malignancy. It has also been associated with acute sciatica.[83] The level of CRP usually peaks within 48 hours of the stimulus. In contrast, when the stimulus for increased production completely ceases, the circulating CRP concentration falls rapidly, at almost the rate of plasma CRP clearance.[84] A fall in serial measurements usually indicates resolution of the underlying process, while persisting elevated levels indicates ongoing inflammation.[85] Where possible, measurements of serum CRP will be repeatedly taken on a 48 hour schedule while the participant is an inpatient; absolute and rates of change of CRP values will be used as candidate predictive factors.[86]

Plasma cell-free DNA [cfDNA] will be used as an indicator of tissue damage. This includes both nuclear DNA [nDNA] and mitochondrial DNA [mtDNA], which circulate after being

For peer review only - http://bmjopen.bmj.com/site/about/guidelines.xhtml

released from cells when they are damaged and are thought to be amongst the important initiators of systemic inflammatory responses following tissue injury known as Damage Associated Molecular Patterns [DAMPs]. [87] Clinical outcomes in trauma patients have been related to plasma mtDNA concentration.[88 89] Other work with severe trauma patients has shown that cfDNA values rise to their peak value in the second week post-trauma, and then gradually return to baseline values after approximately 2 months.[90] Where possible, measurements of circulating cfDNA will be repeatedly taken on a 48 hour schedule while the participant is an inpatient; absolute values and rates of change of cfDNA values will be used as candidate predictive factors.

For peer review only

For peer review only - http://bmjopen.bmj.com/site/about/guidelines.xhtml

References

1. Stewart AL, Greenfield S, Hays RD, et al. Functional status and well-being of patients with chronic conditions. Results from the Medical Outcomes Study. *JAMA* 1989;262(7):907-13.
2. Ware JE, Jr., Sherbourne CD. The MOS 36-item short-form health survey (SF-36). I. Conceptual framework and item selection. *Med Care* 1992;30(6):473-83.
3. Hays RD, Sherbourne CD, Mazel RM. The RAND 36-Item Health Survey 1.0. *Health Econ* 1993;2(3):217-27.
4. McHorney CA, Ware JE, Jr., Raczek AE. The MOS 36-Item Short-Form Health Survey (SF-36): II. Psychometric and clinical tests of validity in measuring physical and mental health constructs. *Med Care* 1993;31(3):247-63.
5. Revell MP, Pynsent, P.B., Abudu, A., Fairbank, J.C.T. . Trauma Scores and Trauma Outcome Measures *Trauma* 2003;5:61-70.
6. Ware J. SF-36 Health Survey Updated. *Spine* 2000;25(24):3130-39.
7. Dattani R, Slobogean, G.P., O'Brien, P.J., Broekhuyse, H.M., Blachut, P.A., Guy, P., Lefaivre, K.A. Psychometric Analysis of Measuring Functional Outcomes in Tibial Plateau Fractures using the Short Form (SF-36), Short Musculoskeletal Function Assessment (SMFA) and the Western Ontario McMaster Osteoarthritis (WOMAC) Questionnaires. *Injury Int J Care Injured* 2013;44:825-29.
8. Brooks R. EuroQol: the current state of play. *Health Policy* 1996;37(1):53-72.
9. Janssen MF, Birnie E, Haagsma JA, et al. Comparing the standard EQ-5D three-level system with a five-level version. *Value Health* 2008;11(2):275-84. doi: 10.1111/j.1524-4733.2007.00230.x
10. Janssen MF, Pickard AS, Golicki D, et al. Measurement properties of the EQ-5D-5L compared to the EQ-5D-3L across eight patient groups: a multi-country study. *Qual Life Res* 2013;22(7):1717-27. doi: 10.1007/s11136-012-0322-4
11. Mahoney FI, Barthel DW. Functional Evaluation: The Barthel Index. *Md State Med J* 1965;14:61-5.
12. Shah S, Vanclay F, Cooper B. Improving the sensitivity of the Barthel Index for stroke rehabilitation. *J Clin Epidemiol* 1989;42(8):703-9.
13. Hseuh IP, Lee, M.M., Hsieh, C.L. . Psychometric Characteristics of the Barthel Activities of Daily Living Index in Stroke Patients *J Formos Med Assoc* 2001;100(526-532)
14. Hseuh IP, Lin, J.H., Jeng, J.S., Hsieh, C.L. Comparison of the Psychometric Characteristics of the Functional Independence Measure, 5 Item Barthel Index and 10 Item Barthel Index in Patients with Stroke. *J Neurol Neurosurg Psychiatry* 2002;73:188-90.
15. Hsieh YW, Wang, C.H., Wu, S.C., Chen, P.C., Sheu, C.F., Hsieh, C.L. . Establishing the Minimally Clinically Important Difference of the Barthel Index in Stroke Patients. *Neurorehabilitation and Neural Repair* 2007;21(3):233-38.
16. Hobart JC, Thompson AJ. The five item Barthel index. *J Neurol Neurosurg Psychiatry* 2001;71(2):225-30.
17. Eriksen HR, Ihlebaek C, Ursin H. A scoring system for subjective health complaints (SHC). *Scand J Public Health* 1999;27(1):63-72.
18. Cappelleri JC BA, McDermott AM, Sadosky AB, Petrie CD, Martin S. Psychometric properties of a single-item scale to assess sleep quality among individuals with fibromyalgia. *Health Qual Life Outcomes* 2009;17(7):54.
19. Mallen CD, Peat G, Thomas E, et al. Prognostic factors for musculoskeletal pain in primary care: a systematic review. *Br J Gen Pract* 2007;57(541):655-61.
20. Artus M, Campbell P, Mallen CD, et al. Generic prognostic factors for musculoskeletal pain in primary care: a systematic review. *BMJ Open* 2017;7(1):e012901. doi: 10.1136/bmjopen-2016-012901

For peer review only - http://bmjopen.bmj.com/site/about/guidelines.xhtml

21. Clay FJ, Newstead SV, Watson WL, et al. Bio-psychosocial determinants of persistent pain 6 months after non-life-threatening acute orthopaedic trauma. *J Pain* 2010;11(5):420-30. doi: 10.1016/j.jpain.2009.12.002
22. Archer KR, Castillo RC, Wegener ST, et al. Pain and satisfaction in hospitalized trauma patients: the importance of self-efficacy and psychological distress. *J Trauma Acute Care Surg* 2012;72(4):1068-77. doi: 10.1097/TA.0b013e3182452df5
23. Rosenbloom BN, Khan S, McCartney C, et al. Systematic review of persistent pain and psychological outcomes following traumatic musculoskeletal injury. *J Pain Res* 2013;6:39-51. doi: 10.2147/JPR.S38878
24. Vranceanu AM, Bachoura A, Weening A, et al. Psychological factors predict disability and pain intensity after skeletal trauma. *J Bone Joint Surg Am* 2014;96(3):e20. doi: 10.2106/JBJS.L.00479
25. Zigmond AS, Snaith RP. The hospital anxiety and depression scale. *Acta Psychiatr Scand* 1983;67(6):361-70.
26. Bjelland I, Dahl AA, Haug TT, et al. The validity of the Hospital Anxiety and Depression Scale. An updated literature review. *J Psychosom Res* 2002;52(2):69-77.
27. Wang W, Chair, S.Y., Thompson, D.R., Twinn, S.F. A Psychometric Evaluation of the Chinese Version of the Hospital Anxiety and Depression Scale in Patients with Coronary Heart Disease *Journal of Clinical Nursing* 2009;18:1908-15.
28. Harland NJ Georgieff K. Development of the coping strategies questionnaire 24, a clinically utilitarian version of the coping strategies questionnaire. *Rehabilitation Psychology* 2003;48(4):296–300.
29. Rosenstiel AK KF. The use of coping strategies in chronic low back pain patients: Relationship to patient characteristics and current adjustment. . *Pain* 1983;17: 33–44.
30. Chiu C JJ, Fujikawa M, Strand D, Cheing G, Lee G, Chan F Measurement Structure of the Coping Strategies Questionnaire-24 in a Sample of Individuals with Musculoskeletal Pain: A Confirmatory Factor Analysis. . *Rehabilitation Research, Policy, and Education* 2014;28(2):80-90.
31. Campbell P, Foster NF, Thomas E, Dunn KM. Prognostic Indicators of Low Back Pain in Primary Care: Five-Year Prospective Study. *J Pain* 2013;14(8):873–83.
32. Woby SR, Roach NK, Urmston M, et al. Psychometric properties of the TSK-11: a shortened version of the Tampa Scale for Kinesiophobia. *Pain* 2005;117(1-2):137-44. doi: 10.1016/j.pain.2005.05.029
33. Miller RP KS, Todd D. The Tampa Scale: a measure of kinesiophobia. *Clin J Pain* 1991;7(1):51-52.
34. Swinkels-Meewisse EJCM, Swinkels RAHM, Verbeek ALM, Vlaeyen JWS. Psychometric Propertieis of the Tampa Scale for Kinesiophobia and the Fear Avoidance Beliecs Questionnaire in Acute Low Back Pain *Manual Therapy* 2003;8(1):29-36.
35. Kall LB. Psychological determinants of quality of life in patients with whiplash associated disorders-a prospective study. *Disabil Rehabil* 2009;31(3):227-36. doi: 10.1080/09638280801912030
36. Nicholas MK. The pain self-efficacy questionnaire: Taking pain into account. *Eur J Pain* 2007;11(2):153-63.
37. Horowitz M, Wilner N, Alvarez W. Impact of Event Scale: a measure of subjective stress. *Psychosom Med* 1979;41(3):209-18.
38. Beck JG, Grant DM, Read JP, et al. The impact of event scale-revised: psychometric properties in a sample of motor vehicle accident survivors. *J Anxiety Disord* 2008;22(2):187-98. doi: 10.1016/j.janxdis.2007.02.007
39. Creamer M, Bell R, Failla S. Psychometric properties of the Impact of Event Scale - Revised. *Behav Res Ther* 2003;41(12):1489-96.

For peer review only - http://bmjopen.bmj.com/site/about/guidelines.xhtml

40. Walker-Bone K HN, Ntani G, Tinati T, Jones GT, Smith BH, Macfarlane GJ, Cooper C. . Chronic widespread bodily pain is increased among individuals with history of fracture: findings from UK Biobank. . *Arch Osteoporos* 2016;11:1.
41. Barbero M, Mores, Leoni D, Gatti R, Egloff M, Falla D. Test-retest reliability of pain extent and pain location using a novel method for pain drawing analysis. *European Journal of Pain* 2015;19:1129-38.
42. Baker SP, O'Neill B. The injury severity score: an update. *J Trauma* 1976;16(11):882-5.
43. Akmal M, Trivedi R, Sutcliffe J. Functional outcome in trauma patients with spinal injury. *Spine (Phila Pa 1976)* 2003;28(2):180-5. doi: 10.1097/01.BRS.0000041577.12233.96
44. Baker SP, O'Neill B, Haddon W, Jr., et al. The injury severity score: a method for describing patients with multiple injuries and evaluating emergency care. *J Trauma* 1974;14(3):187-96.
45. Linn S. The injury severity score--importance and uses. *Ann Epidemiol* 1995;5(6):440-6.
46. Sears JM, Blanar L, Bowman SM. Predicting work-related disability and medical cost outcomes: a comparison of injury severity scoring methods. *Injury* 2014;45(1):16-22. doi: 10.1016/j.injury.2012.12.024
47. NICE. Low back pain and sciatica in over 16s: assessment and management, NICE guideline [NG59]: National Institute for Health and Care Excellence, 2016.
48. Kamaleri Y, Natvig B, Ihlebaek CM, et al. Change in the number of musculoskeletal pain sites: A 14-year prospective study. *Pain* 2009;141(1-2):25-30. doi: 10.1016/j.pain.2008.09.013
49. Clay FJ, Watson WL, Newstead SV, et al. A systematic review of early prognostic factors for persisting pain following acute orthopedic trauma. *Pain Res Manag* 2012;17(1):35-44.
50. Von Korff M, Ormel J, Keefe FJ, et al. Grading the severity of chronic pain. *Pain* 1992;50(2):133-49.
51. Jensen MP, McFarland CA. Increasing the reliability and validity of pain intensity measurement in chronic pain patients. *Pain* 1993;55(2):195-203.
52. Jensen MP, Turner, J.A. and Romano, J.M. . What is the maximum number of levels needed in pain intensity measurement? . *Pain* 1994;58:387–92.
53. Jensen MP, Turner, L.R., Turner, J.A. and Romano, J.M. The use of multiple-item scales for pain intensity measurement in chronic pain patients. *Pain* 1996;67:35–40.
54. Jensen MP, Turner JA, Romano JM, et al. Comparative reliability and validity of chronic pain intensity measures. *Pain* 1999;83(2):157-62.
55. Von Korff M. Epidemiological and survey methods – assessment of chronic pain. In Turk, D.C. and Melzack, R., editors, Handbook of Pain Assessment. . New York, USA: Guildford Press 2001:603-18.
56. Farrar JT, Young JP, Jr., LaMoreaux L, et al. Clinical importance of changes in chronic pain intensity measured on an 11-point numerical pain rating scale. *Pain* 2001;94(2):149-58.
57. Salaffi F, Stancati A, Silvestri CA, et al. Minimal clinically important changes in chronic musculoskeletal pain intensity measured on a numerical rating scale. *Eur J Pain* 2004;8(4):283-91. doi: 10.1016/j.ejpain.2003.09.004
58. Childs JD, Piva SR, Fritz JM. Responsiveness of the numeric pain rating scale in patients with low back pain. *Spine (Phila Pa 1976)* 2005;30(11):1331-4.
59. Ostelo RW, Deyo RA, Stratford P, Waddell G, Croft P, Von Korff M, Bouter LM, Henrica C. Interpreting change scores for pain and functional status in low back pain: towards international consensus regarding minimal important change. *Spine* 2008;33(1):90-94.
60. Michener LA, Snyder AR, Leggin BG. Responsiveness of the numeric pain rating scale in patients with shoulder pain and the effect of surgical status. *J Sport Rehabil* 2011;20(1):115-28.
61. Masters Steedman S, Middaugh SJ, Kee WG, et al. Chronic-pain medications: equivalence levels and method of quantifying usage. *Clin J Pain* 1992;8(3):204-14.
62. Harden RN, Weinland SR, Remble TA, et al. Medication Quantification Scale Version III: update in medication classes and revised detriment weights by survey of American Pain Society Physicians. *J Pain* 2005;6(6):364-71. doi: 10.1016/j.jpain.2005.01.350

For peer review only - http://bmjopen.bmj.com/site/about/guidelines.xhtml

63. Gallizzi M GC, Harden RN, Stanos S, Khan A. Medication Quantification Scale Version III: internal validation of detriment weights using a chronic pain population. . *Pain Pract* 2008;8(1):1-4.
64. Stormo KJ, Kee WG, Steedham S, et al. Medication quantification scores and evaluation of patient pharmacology profiles. *Current Review of Pain* 1998;2(3):171–74.
65. Rosenbloom BN, Katz J, Chin KY, et al. Predicting pain outcomes after traumatic musculoskeletal injury. *Pain* 2016;157(8):1733-43. doi: 10.1097/j.pain.0000000000000580
66. Freynhagen R, Baron R, Gockel U, et al. painDETECT: a new screening questionnaire to identify neuropathic components in patients with back pain. *Curr Med Res Opin* 2006;22(10):1911-20. doi: 10.1185/030079906X132488
67. Cappelleri JC, Koduru V, Bienen EJ, et al. A cross-sectional study examining the psychometric properties of the painDETECT measure in neuropathic pain. *J Pain Res* 2015;8(8):159-67. doi: 10.2147/JPR.S80046
68. Keller T, Freynhagen R, Tolle TR, et al. A retrospective analysis of the long-term test-retest stability of pain descriptors of the painDETECT questionnaire. *Curr Med Res Opin* 2016;32(2):343-9. doi: 10.1185/03007995.2015.1125869
69. Tampin B, Bohne T, Callan M, et al. Reliability of the English version of the painDETECT questionnaire. *Curr Med Res Opin* 2017;33(4):741-48. doi: 10.1080/03007995.2017.1278682
70. Sadosky A, Koduru V, Bienen EJ, et al. Characterizing individual painDETECT symptoms by average pain severity. *Clinicoecon Outcomes Res* 2016;8:361-6. doi: 10.2147/CEOR.S105402
71. Cappelleri JC, Koduru V, Bienen EJ, et al. Mapping painDETECT, a neuropathic pain screening tool, to the EuroQol (EQ-5D-3L). *Qual Life Res* 2017;26(2):467-77. doi: 10.1007/s11136-016-1379-2
72. O'Brien M. Aids to the Examination of the Peripheral Nervous System, 5th Edition: Saunders (Elsevier) 2010.
73. Palmer ST, Martin DJ, Steedman WM, Ravey J, S. C-and Aδ-fibre mediated thermal perception: response to rate of temperature change using method of limits. . *Somatosensory & motor research* 2000;17(4):325-33.
74. Chesterton LS SJ, Wright CC, Foster NE. Interrater reliability of algometry in measuring pressure pain thresholds in healthy humans, using multiple raters. *Clin J Pain* 2007;23(9):760-6.
75. Knutti IA SM, Opsommer E. Test-retest reliability of thermal quantitative sensory testing on two sites within the L5 dermatome of the lumbar spine and lower extremity. *Neurosci Lett* 2014;5(579):157-62.
76. Greenspan JD, McGillis SL. Stimulus features relevant to the perception of sharpness and mechanically evoked cutaneous pain. *Somatosens Mot Res* 1991;8(2):137-47.
77. Sarlani E, Greenspan JD. Gender differences in temporal summation of mechanically evoked pain. *Pain* 2002;97(1-2):163-9.
78. Nie H, Arendt-Nielsen L, Andersen H, et al. Temporal summation of pain evoked by mechanical stimulation in deep and superficial tissue. *J Pain* 2005;6(6):348-55. doi: 10.1016/j.jpain.2005.01.352
79. Nie H, Arendt-Nielsen L, Madeleine P, et al. Enhanced temporal summation of pressure pain in the trapezius muscle after delayed onset muscle soreness. *Exp Brain Res* 2006;170(2):182-90. doi: 10.1007/s00221-005-0196-6
80. Woolf CJ, Bennett GJ, Doherty M, et al. Towards a mechanism-based classification of pain? *Pain* 1998;77(3):227-9.
81. Whicher JT ES. Acute phase proteins. *Hosp Update* 1990:899-905.
82. Gosling P, Dickson GR. Serum c-reactive protein in patients with serious trauma. *Injury* 1992;23(7):483-6.
83. Sturmer T, Raum E, Buchner M, et al. Pain and high sensitivity C reactive protein in patients with chronic low back pain and acute sciatic pain. *Ann Rheum Dis* 2005;64(6):921-5. doi: 10.1136/ard.2004.027045

For peer review only - http://bmjopen.bmj.com/site/about/guidelines.xhtml

84. Pepys MB, Hirschfield GM. C-reactive protein: a critical update. *J Clin Invest* 2003;111(12):1805-12. doi: 10.1172/JCI18921
85. Giannoudis PV HF, Pape HC. . Inflammatory serum markers in patients with multiple trauma. Can they predict outcome? *J Bone Joint Surg Br* 2004;86(3):313-23.
86. Barma M, Goodbrand, J.A., Donnan, P.T., McGilchrist, M.M., Frost, H., McMurdo, M.E. and Witham, M.D. Slower Decline in C-Reactive Protein after an Inflammatory Insult Is Associated with Longer Survival in Older Hospitalised Patients. . *PloS one* 2016;11(7):e0159412.
87. Krysko DV, Agostinis P, Krysko O, et al. Emerging role of damage-associated molecular patterns derived from mitochondria in inflammation. *Trends Immunol* 2011;32(4):157-64. doi: 10.1016/j.it.2011.01.005
88. Simmons JD, Lee YL, Mulekar S, et al. Elevated levels of plasma mitochondrial DNA DAMPs are linked to clinical outcome in severely injured human subjects. *Ann Surg* 2013;258(4):591-6; discussion 96-8. doi: 10.1097/SLA.0b013e3182a4ea46
89. Yamanouchi S, Kudo D, Yamada M, et al. Plasma mitochondrial DNA levels in patients with trauma and severe sepsis: time course and the association with clinical status. *J Crit Care* 2013;28(6):1027-31. doi: 10.1016/j.jcrc.2013.05.006
90. Foster MA. Steroids and immunity from injury through to rehabilitation (SIR Study). University of Birmingham, 2013.

For peer review only

For peer review only - http://bmjopen.bmj.com/site/about/guidelines.xhtml

SPIRIT 2013 Checklist: Recommended items to address in a clinical trial protocol and related documents*

Section/item	Item No	Description
**Administrative information**		
Title	1	Descriptive title identifying the study design, population, interventions, and, if applicable, trial acronym – Page 1
Trial registration	2a	Trial identifier and registry name. If not yet registered, name of intended registry – N/A
	2b	All items from the World Health Organization Trial Registration Data Set – N/A
Protocol version	3	Date and version identifier – Page 1
Funding	4	Sources and types of financial, material, and other support – Page 25
Roles and responsibilities	5a	Names, affiliations, and roles of protocol contributors – Pages 1, 25
	5b	Name and contact information for the trial sponsor – Page 25
	5c	Role of study sponsor and funders, if any, in study design; collection, management, analysis, and interpretation of data; writing of the report; and the decision to submit the report for publication, including whether they will have ultimate authority over any of these activities – Page 25
	5d	Composition, roles, and responsibilities of the coordinating centre, steering committee, endpoint adjudication committee, data management team, and other individuals or groups overseeing the trial, if applicable (see Item 21a for data monitoring committee) – Page 25
**Introduction**		
Background and rationale	6a	Description of research question and justification for undertaking the trial, including summary of relevant studies (published and unpublished) examining benefits and harms for each intervention – Page 5 (introduction, page 6 (aims and objectives)
	6b	Explanation for choice of comparators – Supplementary file
Objectives	7	Specific objectives or hypotheses (Page 6)

For peer review only

Trial design	8	Description of trial design including type of trial (eg, parallel group, crossover, factorial, single group), allocation ratio, and framework (eg, superiority, equivalence, noninferiority, exploratory) – Page 7

Methods: Participants, interventions, and outcomes

Study setting	9	Description of study settings (eg, community clinic, academic hospital) and list of countries where data will be collected. Reference to where list of study sites can be obtained – Page 7
Eligibility criteria	10	Inclusion and exclusion criteria for participants. If applicable, eligibility criteria for study centres and individuals who will perform the interventions (eg, surgeons, psychotherapists) – Pages 8-10
Interventions	11a	Interventions for each group with sufficient detail to allow replication, including how and when they will be administered – N/A
	11b	Criteria for discontinuing or modifying allocated interventions for a given trial participant (eg, drug dose change in response to harms, participant request, or improving/worsening disease) – N/A
	11c	Strategies to improve adherence to intervention protocols, and any procedures for monitoring adherence (eg, drug tablet return, laboratory tests) – N/A
	11d	Relevant concomitant care and interventions that are permitted or prohibited during the trial – N/A
Outcomes	12	Primary, secondary, and other outcomes, including the specific measurement variable (eg, systolic blood pressure), analysis metric (eg, change from baseline, final value, time to event), method of aggregation (eg, median, proportion), and time point for each outcome. Explanation of the clinical relevance of chosen efficacy and harm outcomes is strongly recommended – Page 10 (primary outcome), Supplementary file (candidate predictors)
Participant timeline	13	Time schedule of enrolment, interventions (including any run-ins and washouts), assessments, and visits for participants. A schematic diagram is highly recommended (see Figure) – Pages 8-10
Sample size	14	Estimated number of participants needed to achieve study objectives and how it was determined, including clinical and statistical assumptions supporting any sample size calculations – Pages 14-15
Recruitment	15	Strategies for achieving adequate participant enrolment to reach target sample size – Pages 14-15

Methods: Assignment of interventions (for controlled trials)

Allocation:

Sequence generation	16a	Method of generating the allocation sequence (eg, computer-generated random numbers), and list of any factors for stratification. To reduce predictability of a random sequence, details of any planned restriction (eg, blocking) should be provided in a separate document that is unavailable to those who enrol participants or assign interventions – N/A
Allocation concealment mechanism	16b	Mechanism of implementing the allocation sequence (eg, central telephone; sequentially numbered, opaque, sealed envelopes), describing any steps to conceal the sequence until interventions are assigned – N/A
Implementation	16c	Who will generate the allocation sequence, who will enrol participants, and who will assign participants to interventions – N/A
Blinding (masking)	17a	Who will be blinded after assignment to interventions (eg, trial participants, care providers, outcome assessors, data analysts), and how N/A
	17b	If blinded, circumstances under which unblinding is permissible, and procedure for revealing a participant's allocated intervention during the trial – N/A

Methods: Data collection, management, and analysis

Data collection methods	18a	Plans for assessment and collection of outcome, baseline, and other trial data, including any related processes to promote data quality (eg, duplicate measurements, training of assessors) and a description of study instruments (eg, questionnaires, laboratory tests) along with their reliability and validity, if known. Reference to where data collection forms can be found, if not in the protocol – Pages 10-14, Supplementary file
	18b	Plans to promote participant retention and complete follow-up, including list of any outcome data to be collected for participants who discontinue or deviate from intervention protocols – Page 9 (withdrawals)
Data management	19	Plans for data entry, coding, security, and storage, including any related processes to promote data quality (eg, double data entry; range checks for data values). Reference to where details of data management procedures can be found, if not in the protocol – Page 14
Statistical methods	20a	Statistical methods for analysing primary and secondary outcomes. Reference to where other details of the statistical analysis plan can be found, if not in the protocol – Page 15-16
	20b	Methods for any additional analyses (eg, subgroup and adjusted analyses) – Page 16 (screening tool development and validation)

	20c	Definition of analysis population relating to protocol non-adherence (eg, as randomised analysis), and any statistical methods to handle missing data (eg, multiple imputation) – Page 15 (imputation)
**Methods: Monitoring**		
Data monitoring	21a	Composition of data monitoring committee (DMC); summary of its role and reporting structure; statement of whether it is independent from the sponsor and competing interests; and reference to where further details about its charter can be found, if not in the protocol. Alternatively, an explanation of why a DMC is not needed – N/A
	21b	Description of any interim analyses and stopping guidelines, including who will have access to these interim results and make the final decision to terminate the trial – N/A
Harms	22	Plans for collecting, assessing, reporting, and managing solicited and spontaneously reported adverse events and other unintended effects of trial interventions or trial conduct – N/A
Auditing	23	Frequency and procedures for auditing trial conduct, if any, and whether the process will be independent from investigators and the sponsor – N/A
**Ethics and dissemination**		
Research ethics approval	24	Plans for seeking research ethics committee/institutional review board (REC/IRB) approval – Pages 19-20
Protocol amendments	25	Plans for communicating important protocol modifications (eg, changes to eligibility criteria, outcomes, analyses) to relevant parties (eg, investigators, REC/IRBs, trial participants, trial registries, journals, regulators) – N/A
Consent or assent	26a	Who will obtain informed consent or assent from potential trial participants or authorised surrogates, and how (see Item 32) – Pages 9-10
	26b	Additional consent provisions for collection and use of participant data and biological specimens in ancillary studies, if applicable – N/A
Confidentiality	27	How personal information about potential and enrolled participants will be collected, shared, and maintained in order to protect confidentiality before, during, and after the trial – Page 14
Declaration of interests	28	Financial and other competing interests for principal investigators for the overall trial and each study site – Page 25
Access to data	29	Statement of who will have access to the final trial dataset, and disclosure of contractual agreements that limit such access for investigators – Not present

For peer review only - http://bmjopen.bmj.com/site/about/guidelines.xhtml

Ancillary and post-trial care	30	Provisions, if any, for ancillary and post-trial care, and for compensation to those who suffer harm from trial participation – N/A
Dissemination policy	31a	Plans for investigators and sponsor to communicate trial results to participants, healthcare professionals, the public, and other relevant groups (eg, via publication, reporting in results databases, or other data sharing arrangements), including any publication restrictions – N/A
	31b	Authorship eligibility guidelines and any intended use of professional writers – N/A
	31c	Plans, if any, for granting public access to the full protocol, participant-level dataset, and statistical code – N/A
**Appendices**		
Informed consent materials	32	Model consent form and other related documentation given to participants and authorised surrogates – N/A
Biological specimens	33	Plans for collection, laboratory evaluation, and storage of biological specimens for genetic or molecular analysis in the current trial and for future use in ancillary studies, if applicable – N/A

*It is strongly recommended that this checklist be read in conjunction with the SPIRIT 2013 Explanation & Elaboration for important clarification on the items. Amendments to the protocol should be tracked and dated. The SPIRIT checklist is copyrighted by the SPIRIT Group under the Creative Commons "Attribution-NonCommercial-NoDerivs 3.0 Unported" license.

For peer review only

BMJ Open

DEVELOPMENT OF A SCREENING TOOL TO PREDICT THE RISK OF CHRONIC PAIN AND DISABILITY FOLLOWING MUSCULOSKELETAL TRAUMA: PROTOCOL FOR A PROSPECTIVE OBSERVATIONAL STUDY IN THE UNITED KINGDOM

Journal:	*BMJ Open*
Manuscript ID	bmjopen-2017-017876.R3
Article Type:	Protocol
Date Submitted by the Author:	01-Feb-2018
Complete List of Authors:	Rushton, Alison; University of Birmingham, School of Sport, Exercise and Rehabilitation Sciences<br>Evans, David; University of Birmingham, School of Sport, Exercise and Rehabilitation Sciences<br>Middlebrook, Nicola; University of Birmingham, School of Sport, Exercise and Rehabilitation Sciences<br>Heneghan, Nicola; University of Birmingham, School of Sport, Exercise and Rehabilitation Sciences<br>Small, Charlotte; University of Birmingham, NIHR Surgical Reconstruction & Microbiology Research Centre<br>Lord, Janet; University of Birmingham, NIHR Surgical Reconstruction & Microbiology Research Centre<br>Patel, Jaimin; University of Birmingham, NIHR Surgical Reconstruction & Microbiology Research Centre<br>Falla, Deborah; University of Birmingham, School of Sport, Exercise and Rehabilitation Sciences
**Primary Subject Heading**:	Rehabilitation medicine
Secondary Subject Heading:	Medical management
Keywords:	Musculoskeletal trauma, Precision rehabilitation, Pain mechanisms

SCHOLARONE™
Manuscripts

TITLE PAGE

DEVELOPMENT OF A SCREENING TOOL TO PREDICT THE RISK OF CHRONIC PAIN AND DISABILITY FOLLOWING MUSCULOSKELETAL TRAUMA: PROTOCOL FOR A PROSPECTIVE OBSERVATIONAL STUDY IN THE UNITED KINGDOM

Rushton A[1,2], Evans DW[1,2], Middlebrook N[1,2], Heneghan N[1], Small C[2], Lord J[2], Patel JM[2], Falla D[1,2].

Affiliations

[1] Centre of Precision Rehabilitation for Spinal Pain
School of Sport, Exercise and Rehabilitation Sciences
College of Life and Environmental Sciences
University of Birmingham
Edgbaston
Birmingham
B15 2TT
United Kingdom

[2] NIHR Surgical Reconstruction & Microbiology Research Centre
University of Birmingham
Edgbaston
Birmingham
B15 2TT
United Kingdom

Correspondence:

Professor Deborah Falla
Centre of Precision Rehabilitation for Spinal Pain
School of Sport, Exercise and Rehabilitation Sciences,
College of Life and Environmental Sciences,
University of Birmingham,
Birmingham, B15 2TT, UK.
Tel: +44 121 415 4220
Email: d.falla@bham.ac.uk

For peer review only

For peer review only - http://bmjopen.bmj.com/site/about/guidelines.xhtml

ABSTRACT

Introduction

Pain is an expected and appropriate experience following traumatic musculoskeletal injury. By contrast, chronic pain and disability are unhelpful yet common sequelae of trauma-related injuries. Presently, the mechanisms that underlie the transition from acute to chronic disabling post-traumatic pain are not fully understood. Such knowledge would facilitate the development and implementation of precision rehabilitation approaches that match interventions to projected risk of recovery, with the aim of preventing poor long-term outcomes. The aim of this study is to identify a set of predictive factors to identify patients at risk of developing ongoing post-traumatic pain and disability following acute musculoskeletal trauma. To achieve this, we will use a unique and comprehensive combination of patient-reported outcome measures, psychophysical testing and biomarkers.

Methods/analysis

A prospective observational study will recruit two temporally staggered cohorts [n=250 each cohort; at least 10 cases per candidate predictor] of consecutive acute musculoskeletal trauma patients aged ≥16 years, who are emergency admissions into a Major Trauma Centre in the United Kingdom, with an episode inception defined as the traumatic event. The first cohort will identify candidate predictors to develop a screening tool to predict development of chronic and disabling pain, and the second will allow evaluation of the predictive performance of the tool [validation]. The outcome being predicted is an individual's absolute risk of poor outcome measured at 6 months follow-up using the Chronic Pain Grade Scale [poor outcome ≥Grade II]. Candidate predictors encompass the four primary mechanisms of pain: *nociceptive* [e.g. injury location], *neuropathic* [e.g. painDETECT], *inflammatory* [biomarkers], and *nociplastic* [e.g. quantitative sensory testing]. Concurrently, patient-

For peer review only - http://bmjopen.bmj.com/site/about/guidelines.xhtml

reported outcome measures will assess general health and psychosocial factors [e.g. pain self-efficacy]. Risk of poor outcome will be calculated using multiple variable regression analysis.

Ethics and dissemination

Approved by the NHS Research Ethics Committee (17/WA/0421).

Keywords

Keywords: Pain mechanisms, Prediction, Precision rehabilitation, Musculoskeletal trauma

Funding: NIHR SRMRC

Word count

4228 [excluding tables and references]

For peer review only

STRENGTHS AND LIMITATIONS OF THIS STUDY

- A comprehensive array of candidate predictive factors will allow for the prediction of chronic and disabling pain following trauma
- These predictive factors will enable the development and validation of a predictive tool to predict good and poor outcome at 6 months post-injury
- The prospective design of the study enables control of unwarranted influences, and enables a stronger case for inferring causal relationships
- Identifying predictive factors related to poor outcome of pain and disability outcome will facilitate targeting of effective interventions
- Other candidate predictors may have been useful to include [e.g. vibration thresholds], but consideration of burden to participants of testing and sample size considerations necessitated prioritisation of candidate predictive factors.

For peer review only

INTRODUCTION

Pain is an expected and appropriate experience that usually follows traumatic injury.[1] By contrast, chronic pain and disability are unhelpful but common sequelae of trauma-related injuries.[2] Gaining an understanding of why some people develop chronic and disabling post-traumatic pain is therefore a priority for individual patients, the military and society at large. Notwithstanding, the mechanisms that underlie the transition from acute to chronic disabling post-traumatic pain are not fully understood. Such knowledge would facilitate the development and implementation of a clinical pathway of care that matches interventions to projected risk of poor recovery, with the aim of preventing poor long-term outcomes. This project stems from advances in knowledge relating to the assessment and management of pain[3] and the quantification of potential predictive factors to inform personalised rehabilitation; identifying which patients to target with rehabilitation and when and how to target them.

Few studies have specifically explored predictive factors for recovery, whether poor or good, following physical trauma. Of those that have, psychosocial variables such as anxiety, depression and post-traumatic stress, have so far been identified as the strongest predictors of outcome.[4-7] However, only a limited number of variables have hitherto been evaluated as potential predictive factors. Indeed, current opinion regarding pain mechanisms[8] suggests that the development of chronic pain and disability cannot be entirely attributable to psychosocial factors. This is consistent with research in primary care that has identified predictive factors for poor outcome across a range of musculoskeletal pain conditions[9], which include: widespread pain, high functional disability, high pain intensity, long pain duration,

For peer review only - http://bmjopen.bmj.com/site/about/guidelines.xhtml

high depression/anxiety, presence of previous pain episodes, movement restriction, and poor coping strategies. Moreover, developments in the mechanistic understanding of pain[10-12] suggest that other measures [e.g. indicators of central sensitisation, inflammatory activity] may have potential predictive utility, especially in an acute trauma population.

Aims of study

Using a unique combination of: 1) general patient characteristics including premorbid neuropsychological status, 2) quality of life and physical functioning, 3) psychosocial features, 4) injury characteristics, 5) pain characteristics, 6) quantitative sensory testing, and 7) biomarkers; we aim to find a set of predictive factors to identify patients at risk of developing ongoing post-traumatic pain and disability following acute musculoskeletal trauma. This will subsequently inform the feasibility of developing and evaluating a new clinical care pathway of precision rehabilitation that matches interventions to the predicted risk of poor recovery.

Objectives

1) Identify predictive factors for poor outcome [chronic pain and disability at 6-months post-injury] following acute musculoskeletal trauma.

2) Develop a predictive model to inform a screening tool to identify the predicted risk of poor recovery [transition from acute post-traumatic pain to chronic pain and disability].

3) Estimate the predictive performance of the screening tool through validation of the model in a separate data set.

For peer review only - http://bmjopen.bmj.com/site/about/guidelines.xhtml

For peer review only

4) Document the clinical course of symptoms at 3 and 12 months following acute musculoskeletal trauma.

METHODS AND ANALYSIS

Source of data

The study will be a prospective, observational study of two temporally staggered cohorts of trauma patients, who are emergency department admissions into a Major Trauma Centre in the United Kingdom, with an episode inception defined as the traumatic event [Figure 1]. The first cohort will facilitate development of the prediction model to inform the screening tool, and the second will enable validation of the prediction model through evaluation of the predictive performance of the model and tool.[13 14] There will be an interval of at least 6 months between recruitment into the two respective cohorts. The prospective design enables control of unwarranted influences, and enables a stronger case for inferring causal relationships. The nature of the study necessitates predictive statistical modelling.[15] This protocol is written in line with the TRIPOD statement,[16] in which recommendations are given for the reporting of prediction model development and validation.

Self-reported and physical assessment predictive data will be collected at baseline over a period of up to 14 days [or duration of inpatient stay if shorter], which will commence immediately following recruitment. Biomarker data collection will occur throughout the same baseline period, but can commence prior to recruitment providing assent is gained from a legal consultee. The outcome data will be collected at 6-months post-injury [working definition of chronic pain];[17] ; the point of evaluation of an individual's absolute risk of poor

For peer review only - http://bmjopen.bmj.com/site/about/guidelines.xhtml

outcome [objectives 1, 2 and 3]. In addition, selected data will be measured at 3 and 12-months post-injury to explore the clinical course of recovery following injury in the shorter and longer term [objective 4].

Figure 1. Study design

[Insert Figure 1 here].

Participants

Participants will be recruited from the register of a Major Trauma Centre in the United Kingdom for a period of up to 24 months [planned start date January 2018]. All consecutive eligible patients will be approached for recruitment until the sample size is achieved.

Eligibility criteria

Inclusion criteria: Adult patients aged ≥16 years who are emergency department admissions into the Major Trauma Centre, with their main criteria for admission being acute musculoskeletal trauma within the preceding 14 days, and a capacity to use and understand written and verbal English language and a mental capacity to provide informed consent [e.g. no confusion, delirium, severe cognitive impairment, or severe mental illness, defined by a score of 6 or less on the Abbreviated Mental Test][18]. The primary reason for including patients with trauma occurring up to 14 days previously is to be inclusive of patients who are critically ill and/or with diminished mental capacity initially following their trauma, and

For peer review only - http://bmjopen.bmj.com/site/about/guidelines.xhtml

patients requiring surgery as a result of the trauma; representative of the broad trauma population.

Exclusion criteria: Exclusions will be made where the patient has an acute intra-cranial lesion [e.g. bleed] combined with a score of ≤14 on the Glasgow Coma Scale [19] [a 15-item measure of consciousness impairment with adequate reliability[20] that is routinely taken in trauma patients at hospital admission], where there is evident brain or central nervous system injury or impairment, long-term neuro-cognitive disorders [such as brain tumour, multiple sclerosis, Alzheimer's and Parkinson's diseases, etc.], comorbid cancer, the presence of an ongoing rheumatological condition, prolonged use of corticosteroids, or terminal illness with short life expectancy.

Withdrawals: Participants will be informed that they are free to withdraw from the study at any time, without needing to provide reason. In the event of death within 3 months of being recruited, participants will be automatically withdrawn from the study and the primary predictive analysis. Baseline data of all withdrawn participants will be kept and compared to those of retained participants to assess for any differences.

Recruitment

Based on feasibility data [site data from the Trauma Audit and Research Network], it is estimated that at least 1,000 eligible trauma patients will be approachable for recruitment over a 24-month period, and that 50% would be expected to consent to participation. A dedicated team of research nurses will be available to recruit patients 7 days per week [from 0700 to 1930].

For peer review only - http://bmjopen.bmj.com/site/about/guidelines.xhtml

Because of impairments resulting from their injuries, some otherwise eligible patients will lack the mental capacity to provide informed consent when first approached to enrol in the study. Recruitment into the study will therefore be undertaken under the guidance and provision of the [UK] Mental Capacity Act 2005 for research in emergency situations. If the patient lacks sufficient capacity to consent, written assent for study participation will be sought from a legal consultee to begin biomarker data collection [blood samples], with informed consent for full recruitment [and subsequent data collection] being sought from the patient only if, and when, they regain sufficient capacity to provide this. If the patient does not regain capacity to provide consent within 14 days of their trauma, they will not be recruited into the study, biomarker data collection will cease, and any blood samples already collected will be destroyed before analysis.

Once informed consent is gained and the participant recruited, following a minimum 1 hour lead time after the informed consent process [to reduce patient burden], members of the research team will visit the patient at their bedside to collect baseline self-reported data via questionnaires [Table 1]. On the next available working day following completion of the questionnaires [again, to reduce patient burden], members of the study team will return to the patient to conduct the first physical [quantitative sensory testing] assessment. At each visit, if deemed necessary the capacity of the participant will be checked using an Abbreviated Mental Test[18] [a score of 6 or lower is indicative of reduced capacity], and asked if they are happy to proceed with data collection.

Outcome

For peer review only - http://bmjopen.bmj.com/site/about/guidelines.xhtml

The outcome for the prediction model is an individual's absolute risk of poor outcome [chronic pain and disability] at 6 months post-injury. Outcome will be measured using the Chronic Pain Grade Scale [CPGS],[21] which combines pain intensity and pain-related disability over the preceding 6-months into a single measure of pain *severity*. The CPGS has previously been used to assess the severity of body-wide chronic pain in general population,[22] primary care[23] and post-trauma[24] populations. Each item of the CPGS relates to at least one of the three categories of the International Classification of Functioning, Disability and Health [ICF] [25]: impairment, activity limitations and restricted participation. Furthermore, all ICF categories are encompassed by the CPGS.[26] The CPGS is a unidimensional scale, with good internal consistency across different pain populations; Cronbach's alpha of 0.84 to 0.91 in back pain, 0.79 for headache, and 0.84 for temporomandibular pain.[21 27] With regards to construct validity, cross-sectional and longitudinal studies of general practice patients have shown high scores on the CPGS, indicating greater chronic pain, to be associated with higher rates of unemployment, greater pain impact scale scores, greater use of opioid analgesics and physician visits, depressed mood, and lower self-rated health status.[21 27 28] For convergent validity, the CPGS has been found to have good correlation with equivalent dimensions of the SF-36.[27 28] In terms of responsiveness, changes in score over time in patients with chronic musculoskeletal pain correlated significantly with changes in SF-36 scores.[29] The CPGS has also been shown to have good test-retest reliability in primary care patients with back pain [weighted kappa 0.81, 95% CI 0.65, 0.98].[27]

Although pain persistence is not used in assigning pain grade, a measure of pain days in the prior 6-months is included in the CPGS.[30] The responses on the remaining 7-items are used for computing scores for the 3 subscales of the CPGS:[21] characteristic pain intensity, disability score, and disability points. The characteristic pain intensity score [range: 0-100] is obtained by calculating the mean of 3 pain intensity measurements: 'at the present time', the

For peer review only - http://bmjopen.bmj.com/site/about/guidelines.xhtml

'worst pain' in the preceding 6 months, and the 'average' pain over the preceding 6 months. The disability score [range: 0-100] is obtained through the mean ratings of how much the pain has interfered in performing activities of daily living, recreational, social and family activities, and work [including housework] activities in the last 6-months. The disability points are scored 0-3 and are derived from a combination of ranked categories of the number of disability days [the number of days that the respondent was away from usual activities in the preceding 6 months due to pain] and disability score. Based on these scores, the participant's chronic pain and disability status can then be classified into one of the 5 ordinal categories of chronic pain severity:[21] no pain [Grade 0], low disability and low intensity pain [Grade I], low disability and high intensity pain [Grade II], high disability and moderately limiting intensity pain [Grade III], and high disability and severely limiting intensity pain [Grade IV]. As in previous studies, poor outcome will be defined as Grade ≥II.[23 31-34]

Candidate predictors

Candidate predictors have been selected that are: [1] reliable and valid measures of their domain, and [2] have a theoretical association with the development of chronic pain. Both modifiable and non-modifiable candidate predictors are included. Candidate predictors are summarised in Table 1, with further detail in Supplementary file S1. Table 1 details important data that will be measured at 3, 6 and 12-months post-injury to explore the clinical course of recovery following injury in the shorter and longer term. All data collection will be standardised through protocols and clinical report forms.

Table 1: Summary of data collection at different assessment points

**Domain / Candidate predictor**	**Measure / data item**	**Baseline** *Commencing ≤14 days*	**3 months** *Clinical course*	**6 months** *Outcome assessment*	**12 months** *Clinical course*

For peer review only - http://bmjopen.bmj.com/site/about/guidelines.xhtml

		post-trauma		*point / clinical course*	
**General patient characteristics including premorbid neuropsychological status**					
Age	In years	√			
Gender	Female / male / other	√			
Body Mass Index [BMI]	Calculated from height and weight measurements	√			
Education	Highest educational level attained	√			
Employment status	Full-time/ part-time / not working / retired / student Employed / self-employed	√	√	√	√
Circumstance of trauma	Military / civilian	√			
Previous history of musculoskeletal pain and injury	Patient history data from patient recollection and medical records	√			
Comorbidity of other health problems	Patient history data from patient recollection and medical records	√			
Premorbid physical health	Patient history data from patient recollection and medical records	√			
Premorbid psychological health	Patient history data from medical records and patient recollection [including non-somatic items from the Subjective Health Complaints Inventory][35]	√			
Number of days in hospital	Intensive care / ward / total	√			
**Quality of life and physical functioning**					
General health	36-item Short Form Health Survey, version 2 [SF-36v2][36]	√	√	√	√
Health-related quality of life	EuroQol EQ-5D-5L[37]	√	√	√	√
Self-care and mobility during activities of daily living	Barthel Index of Activities of Daily Living,[38] collected from hospital data	√			
Sleep quality	11-point [0-10] Numerical Rating Scales, relating to current pain, from 'best possible sleep' to 'worst possible sleep'[39]	√	√	√	√
Brain/CNS impairment	Glasgow Coma Scale [19]	√			
**Psychosocial features**					
Anxiety and depression	Hospital Anxiety and Depression Scales [HADS][40]	√	√	√	√
Coping strategies applied during a painful experience	Coping Strategies Questionnaire-24 [CSQ-24][41]	√	√	√	√
Fear of movement or fear of injury or re-injury during movement	Tampa Scale of Kinesiophobia, 11-item [TSK-11][42]	√	√	√	√
Confidence in ability to perform activities despite pain	Pain Self-Efficacy Questionnaire[43]	√	√	√	√
Subjective post-traumatic distress	Impact of Event Scale revised [IES-R][44]	√	√	√	√

For peer review only - http://bmjopen.bmj.com/site/about/guidelines.xhtml

**Injury characteristics**					
Time of injury/incident	Hospital record data	√			
Injury location	Adapted pain drawings, based on hospital record data	√			
Tissues damaged	Based on imaging data and hospital records<br>Fractures<br>Penetrating / non-penetrating injury / both	√			
Surgery required	Location and post-injury timing of surgery, based on hospital record data	√			
Assisted mechanical ventilation required	Yes / no / duration	√			
Severity of injury	Injury Severity Scale[45]	√			
**Pain characteristics**					
Chronic pain severity	Chronic Pain Grade Scale[21]			√	√
Pain intensity	11-point [0-10] Numerical Rating Scale, relating to current pain, from 'no pain' to 'pain as bad as could be' [collected as part of the Chronic Pain Grade Scale]	√[1]	√	√	√
Pain medication intake [type, dosage and timing]	Medication Quantification Scale,[46-48] based on hospital record data	√[1]			
Pain location	Pain drawing	√[1]	√	√	√
Pain extent	Electronic pain drawing[49]	√[1]			
Self-reported features of neuropathic pain	painDETECT questionnaire[50]	√[1]	√	√	√
**Quantitative sensory testing**					
Heat pain threshold	Evaluation of pain threshold using a heat stimulus	√[1]			
Cold pain threshold	Evaluation of pain threshold using a cold stimulus	√[1]			
Pressure pain threshold	Evaluation of pain threshold using a pressure stimulus	√[1]			
Temporal summation	Evaluation of pain intensity in response to repetitive pressure stimuli	√[1]			
**Biomarkers**					
C-reactive protein [CRP]	Serum levels of CRP, a broad indicator of inflammation [via blood analysis]	√[2]			
Cell-free DNA [cfDNA]	Plasma levels of cell-free [nuclear and mitochondrial] DNA, indicators of tissue damage [via blood analysis]	√[2]			

[1] Measurements to be taken repeatedly throughout the baseline period, which will commence immediately following recruitment via informed consent [up to 14 days post-trauma] for a period of up to 14 days [i.e. until a maximum of 28 days post-trauma], whilst the participant is in hospital

[2] Measurements to be taken repeatedly throughout the baseline period, but may be commenced prior to this, subject to assent from a legal consultee

Data handling

For peer review only - http://bmjopen.bmj.com/site/about/guidelines.xhtml

For peer review only

Blood samples will be collected through the clinical and research nurse teams, whilst the participant is in the hospital, and either analysed immediately [C-reactive protein] or securely stored for subsequent analysis [cell-free DNA]. Baseline self-reported questionnaires, pain and injury drawings, and physical assessments will be collected by one of three trained assessors from the study team. Inter-rater reliability studies [across 2 assessors] will first be conducted in both healthy and trauma populations to inform definitive testing protocols. The order of physical assessment data collection will be randomly assigned [using computerised randomisation software] according to the modality of testing and by site, to prevent order effects. Follow-up self-reported questionnaires will be posted to participants at their home addresses; with up to two postal reminders and a telephone call for non-response. All questionnaires will be formatted so that data can be scanned or entered directly into an electronic database. Following data entry, data will be checked by a second researcher for completeness and accuracy. In addition, regular audits of data collection and storage will be performed by an independent study management committee. Participant identifiable information will be securely stored within the hospital, in line with current United Kingdom data protection legislation, and only accessible by the site Principal Investigator and Research Nurse team who will not be involved in data analysis. All outcome measure data will be securely transferred within an anonymised database file to physically secure servers at the University of Birmingham, and stored for a period of 10-years in line with Research Governance procedures. Participants will receive usual care, and interventions received will be recorded for descriptive analysis. Anonymised data will be analysed using IBM SPSS Statistics.

Sample size

For peer review only - http://bmjopen.bmj.com/site/about/guidelines.xhtml

In predictive modelling, a larger sample size enables lower bias and variance, and permits the prospective prediction of new observations.[15] The number of predictors will be reduced using exploratory factor analysis. This process will ensure that the sample size provides at least 10 cases per candidate predictor, to adequately power the final regression analysis.[51 52] Data will be collected for an estimated 300 participants per cohort [n=600 total] to allow for withdrawals [primarily expected deaths within the first 3 months] and losses to follow-up, so that final data are available for 250 participants per cohort [n=500 total].

Statistical analysis methods and management of missing data

For each cohort, potentially eligible patients, numbers examined for eligibility, confirmed eligible, recruited into the study, completing follow-up, and analysed will be reported in a flow diagram. Reasons for non-participation, exclusion, drop-outs and withdrawal [e.g. death] will be reported at each stage. Participant characteristics will be descriptively presented. For each variable of interest, the number of participants with missing data will be reported.

For the first cohort to develop the predictive model, an initial exploratory data analysis stage will summarise the data.[15] Correlations between candidate predictive factors will be calculated at baseline. Outcome [CPGS] scores will be dichotomised into good and poor categories as described previously. Data reduction will use exploratory factor analysis to assess factor loading of candidate predictors [summary scores] on poor outcome at 6 months. This will enable the number of candidate predictors entered into the final model to be reduced to 25, which can be supported by the cohort sample of 250. This process reduces the risk of

For peer review only - http://bmjopen.bmj.com/site/about/guidelines.xhtml

over-fitting the model and the risk of selecting the wrong variables due to correlation between predictor variables [multicollinearity].[53]

Statistical modelling for prediction has been planned *a priori*. To explore the influence of each predictive factor on poor outcome at 6 months, a logistic multivariable regression model will be fitted to the dichotomised outcome scores to calculate low and high risk of poor outcome. Odds ratios for each candidate predictive factor will be reported, adjusted for other factors and account for clustering [e.g. level of injury severity]. If necessary, multiple imputation[54] will be used to deal with missing outcome data. The characteristics of those patients with and without 6-month data will also be compared, to inform whether patients with no 6-month data were missing at random. Reduced multivariable analyses will be considered if necessary [e.g. removing one of two candidate predictive factors that are highly correlated at baseline], to examine the robustness of the conclusions.

Risk groups and development of the predictive screening tool

The predictive model will be used to develop a risk stratification tool to inform an individual's absolute risk of poor outcome. The stratification tool will inform clinical decision-making for precision rehabilitation. Items will be selected for the model if they are statistically significantly [$p<0.05$] associated with poor outcome in the logistic regression analysis, and those deemed clinically important to retain using expert opinion [regardless of statistical significance, study steering group] to improve face validity for clinicians and avoid over-fitting of the model.[53] The regression model with included predictive factors will be fitted to the data from the first of the two cohorts to obtain a final set of parameter estimates

For peer review only - http://bmjopen.bmj.com/site/about/guidelines.xhtml

[i.e. alpha and beta terms], which will be used to form the model. An important requirement of the stratification tool is that it should be sufficiently brief to facilitate use in clinical practice. Thus, we will look to simplify the model where possible to facilitate its use, but without important reduction in its predictive ability in terms of calibration and discrimination. For example, if multi-item questionnaire scores are included in the model, then we will evaluate whether just one of the questionnaire items is sufficient. Ideally, this process will result in a full and simplified model.

Development versus validation

For validation of the model, data from the second of the two cohorts will be compared to that of the first to enable analysis of the distribution of important variables; inclusive of demographic, predictor and outcome variables. The predictive performance of the screening tool [discrimination, calibration, and goodness of fit] will be assessed using data from the second cohort. Data in both cohorts will be consistent in terms of setting, eligibility criteria, outcome, and predictors.

DISCUSSION

There is an urgent need for a robust predictive study to predict the transition from acute to chronic pain in a musculoskeletal trauma population. Using such a comprehensive array of outcome measures will allow the development and validation of a predictive tool to predict development of chronic and disabling pain, and begin the process of identifying appropriate and precision interventions.

For peer review only - http://bmjopen.bmj.com/site/about/guidelines.xhtml

The candidate predictors used in this study have been chosen to be as comprehensive as possible, based on current knowledge of pain science. Other candidate predictors were considered [e.g. microRNA biomarkers], but their mechanistic functions and temporal progression are not yet sufficiently clear to justify the expense of their inclusion. The combination of patient reported outcome measures, psychophysical testing and biomarkers that are included are designed to act as surrogates for the four primary mechanisms of pain: [55 56 8] *nociceptive* [injury location, severity and characteristics], *neuropathic* [painDETECT tool and pain extent, *inflammatory* [biomarkers], and *nociplastic* [quantitative sensory testing, painDETECT and pain location and extent]. In addition, other patient-reported outcome measures [e.g. pain intensity, post-traumatic stress, anxiety and depression, coping, and pain self-efficacy] are included as the domains that they measure have been shown to influence prognosis for long-term outcomes in populations with pain in a range of locations.[9 23 24]

Rehabilitation is widely regarded as an important component of post-trauma healthcare;[57] however, the current position of equipoise means that precision rehabilitation has not yet been identified. Understanding mechanisms that underlie the transition from acute to chronic pain is essential to moving beyond this position. Identifying predictive factors related to poor outcome of pain and disability outcome will facilitate targeting of effective interventions. This will inform rehabilitation decision making, and facilitate improvements in clinical and cost effectiveness of rehabilitation interventions.

Limited research has identified criteria for quality in a predictive model, but authors have identified potential quality issues to ensure methodological rigour.[58] These issues are summarised in Table 2 and incorporated into the study design to ensure low risk of bias in development and validation of the predictive model.

For peer review only - http://bmjopen.bmj.com/site/about/guidelines.xhtml

Table 2: Methodological decisions to improve study quality

Criteria [58]	Methodological decisions to improve quality
**Study design**	
Inception cohort	• Clear description of population<br>• Clear description of the participants at baseline
Source population	• Clear description of population<br>• Clear description of sampling frame and recruitment [method and timing]
Inclusion and exclusion criteria	• Clarity of eligibility criteria
Prospective design	• Clarity of study design
**Study attrition**	
Number of drop-outs	• Adequate participation rate<br>• Clear description of attempts to collect information on participants who dropped out<br>• Reporting numbers and reasons for loss to follow-up
Information provided on method of management of missing data	• Appropriate methods of imputation of missing data
**Predictive factors**	
All predictive factors described used to develop the model	• Clear definition of predictive factors<br>• An adequate proportion of participants has complete data for the predictive factor
Standardised or valid measurements	• The measurement of the predictive factor is reliable and valid<br>• The measurement of the predictive factor is the same for all participants
Linearity assumption studied	• Linearity of data will be reported
No dichotomization of predictive variables	• Continuous variables will be reported where possible
Data presentation all predictive factors	• Complete data will be presented
**Outcome measures**	
Description of outcome measures	• The outcome is clearly defined
Standardised or valid measurements	• The measurement of the outcome is reliable and valid<br>• The measurement of the outcome is the same for all participants
Data presentation of most important outcome measures	• Complete data will be presented
**Analysis**	
Presentation of univariate crude estimates	• An appropriate strategy for model building is described<br>• An adequate statistical model described
Sufficient numbers of subjects per variable	• Adequate data will be presented
Selection method of variables explained	• Sufficient data will be presented to enable assessment of the adequacy of the analytic strategy

For peer review only - http://bmjopen.bmj.com/site/about/guidelines.xhtml

	• All results will be reported
Presentation of multivariate estimates	• An appropriate strategy for model building is described<br>• An adequate statistical model described
**Clinical performance / validity**	
Clinical performance	• Clinical performance of the model will be reported
Internal validation	• Internal validation will be reported
External validation	Not a focus of this study

ETHICS AND DISSEMINATION

The project has been approved by a NHS Research Ethics Committee (17/WA/0421).

Patient burden and potential distress

The primary ethical concern is to limit distress on participants. As such, to reduce the patient burden when collecting baseline data, the self-reported questionnaires will be administered by members of the study team shortly following obtaining fully informed consent, and physical assessment outcomes will be measured at least 24 hours later. Patients will be asked to consent to not only providing new data for the study, but also for the study team to access information that will have been routinely collected by the hospital staff since the time of admission [e.g. nature and circumstances of injury, previous medical history, medication details, blood test results]. This will be fully explained to patients and explicitly detailed in the participant information sheet.

Mental capacity

For peer review only - http://bmjopen.bmj.com/site/about/guidelines.xhtml

Because of the nature of their injuries, the patient's mental capacity will be assessed on admission into hospital and thereafter by clinical staff and/or research nurses. The mental capacity of eligible patients at the time of being approached for recruitment will therefore fall into one of two groups: either they possess or are lacking mental capacity [in accordance with the Mental Capacity Act 2005] to provide informed consent to voluntarily participate in the study.

For patients possessing mental capacity to provide consent, a research nurse or member of the research team will ask if they are interested in participating in the study. If they are interested, a copy of the participant information sheet will be provided [and if necessary read to them] to give them an outline of the study. Following an opportunity to seek additional information and ask questions, the patient will be asked if they wish to provide their written informed consent to participate in the study, at which point a consent form will need to be signed.

On admission to the hospital, an otherwise eligible patient may lack the mental capacity to decide whether to provide consent to participate in a research study [e.g. due to the severity of their injuries, because they are arriving intubated and ventilated, or as a side-effect of medication for their injuries]. They may or may not regain this capacity during their stay in the hospital. Due to our wish to begin measuring biomarkers as early as possible following the onset of trauma, for some otherwise eligible patients it would be necessary to take blood samples before the patient has regained the capacity to provide informed consent. Using the convention of previous studies in trauma populations,[59] recruitment into the study will be undertaken under the provision and guidance of the Mental Capacity Act 2005 for research in emergency situations, and in accordance with the Declaration of Helsinki. As

For peer review only - http://bmjopen.bmj.com/site/about/guidelines.xhtml

such, if a patient does not possess this capacity when first approached for recruitment, the research team will request a mandate to collect blood samples from a legal consultee. This mandate can continue until the patient gains sufficient capacity to make an informed decision as to whether they wish to provide consent or not. We will use this mandate up to 14 days from the date of the trauma. If the patient does not regain capacity within 14 days following the trauma, or if informed consent is not provided by the patient once capacity to do so is regained, any samples collected will be destroyed before any non-clinical biomarker analysis [i.e. cell-free DNA] is performed. Furthermore, only once informed consent has been gained from the patient would the research team proceed to collect any self-reported questionnaire or physical assessment data. The legal consultee can either be a ‘personal consultee’ e.g. family member, or a ‘nominated consultee’ e.g. intensive care consultant. Once a consultee [personal or nominated] has been identified, they will be provided with the participant information sheet, to inform them about the study. The consultee will be asked if they feel participating in the study would be something to which the patient would agree or object to. If, in their opinion, the patient would agree to participating in the study, the consultee will be asked to sign a declaration form, and the research team can begin the schedule of blood sample collections. If, at any time prior to the patient regaining capacity, the consultee decides to withdraw assent, then no further samples will be collected until the patient can be approached for formal recruitment [if appropriate].

<u>Other ethical issues</u>

Participants will be informed that they are free to withdraw from the study at any time, without needing to provide reason. At each data collection visit, the capacity of the participant will be checked [using an Abbreviated Mental Test] and asked if they are happy to

For peer review only - http://bmjopen.bmj.com/site/about/guidelines.xhtml

proceed with data collection. Any concerns for a participant by the study team will be fed back to clinical staff. All blood samples will be collected by hospital staff and the research nurse team and will be stored, tested and disposed of in accordance with current United Kingdom guidelines and regulations. In the event of death within 3 months of being recruited, participants will be automatically withdrawn from the study and the primary predictive analysis. Baseline characteristics of withdrawn participants will be compared to those of retained participants to assess for any differences.

For peer review only

For peer review only - http://bmjopen.bmj.com/site/about/guidelines.xhtml

REFERENCES

1. Berben SA, Schoonhoven L, Meijs TH, et al. Prevalence and relief of pain in trauma patients in emergency medical services. *Clin J Pain* 2011;27(7):587-92. doi: 10.1097/AJP.0b013e3182169036
2. Williamson OD, Epi GD, Gabbe BJ, et al. Predictors of moderate or severe pain 6 months after orthopaedic injury: a prospective cohort study. *J Orthop Trauma* 2009;23(2):139-44. doi: 10.1097/BOT.0b013e3181962e29
3. Mannion RJ, Woolf CJ. Pain mechanisms and management: a central perspective. *Clin J Pain* 2000;16(3 Suppl):S144-56.
4. Holbrook TL, Anderson JP, Sieber WJ, et al. Outcome after major trauma: discharge and 6-month follow-up results from the Trauma Recovery Project. *J Trauma* 1998;45(2):315-23; discussion 23-4.
5. Holbrook TL, Anderson JP, Sieber WJ, et al. Outcome after major trauma: 12-month and 18-month follow-up results from the Trauma Recovery Project. *J Trauma* 1999;46(5):765-71; discussion 71-3.
6. Harris IA, Young JM, Rae H, et al. Predictors of general health after major trauma. *J Trauma* 2008;64(4):969-74. doi: 10.1097/01.ta.0000245972.83948.1a
7. Ponsford J, Hill B, Karamitsios M, et al. Factors influencing outcome after orthopedic trauma. *J Trauma* 2008;64(4):1001-9. doi: 10.1097/TA.0b013e31809fec16
8. Vardeh D, Mannion RJ, Woolf CJ. Toward a Mechanism-Based Approach to Pain Diagnosis. *J Pain* 2016;17(9 Suppl):T50-69. doi: 10.1016/j.jpain.2016.03.001
9. Artus M, Campbell P, Mallen CD, et al. Generic prognostic factors for musculoskeletal pain in primary care: a systematic review. *BMJ Open* 2017;7(1):e012901. doi: 10.1136/bmjopen-2016-012901
10. Latremoliere A, Woolf CJ. Central sensitization: a generator of pain hypersensitivity by central neural plasticity. *J Pain* 2009;10(9):895-926. doi: 10.1016/j.jpain.2009.06.012
11. Linley JE, Rose K, Ooi L, et al. Understanding inflammatory pain: ion channels contributing to acute and chronic nociception. *Pflugers Arch* 2010;459(5):657-69. doi: 10.1007/s00424-010-0784-6
12. Woolf CJ. Central sensitization: implications for the diagnosis and treatment of pain. *Pain* 2011;152(3):S2-15.
13. Altman DG, Vergouwe Y, Royston P, et al. Prognosis and prognostic research: validating a prognostic model. *BMJ* 2009;338(338):b605. doi: 10.1136/bmj.b605
14. Moons KG, Kengne AP, Grobbee DE, et al. Risk prediction models: II. External validation, model updating, and impact assessment. *Heart* 2012;98(9):691-8. doi: 10.1136/heartjnl-2011-301247
15. Shmueli G. To explain or to predict? *Statistical Science* 2010;25(3):289-310.
16. Collins GS, Reitsma, J.B., Altman, D.G. and Moons, K.G., . Transparent reporting of a multivariable prediction model for individual prognosis or diagnosis (TRIPOD): the TRIPOD statement. *BMC Medicine* 2015;13(1)
17. Merskey H Bogduk N. Classification of chronic pain : descriptions of chronic pain syndromes and

definitions of pain terms. USA: IASP Press 1994.
18. Hodgkinson HM. Evaluation of a mental test score for assessment of mental impairment in the elderly. *Age and Ageing* 1972;1(4):233-8.
19. Teasdale G, Jennett B. Assessment of coma and impaired consciousness. A practical scale. *Lancet* 1974;2(7872):81-4.
20. Reith FC, Van den Brande R, Synnot A, et al. The reliability of the Glasgow Coma Scale: a systematic review. *Intensive Care Med* 2016;42(1):3-15. doi: 10.1007/s00134-015-4124-3

For peer review only - http://bmjopen.bmj.com/site/about/guidelines.xhtml

21. Von Korff M, Ormel J, Keefe FJ, et al. Grading the severity of chronic pain. *Pain* 1992;50(2):133-49.
22. Parsons S, Breen A, Foster NE, et al. Prevalence and comparative troublesomeness by age of musculoskeletal pain in different body locations. *Fam Pract* 2007;24(4):308-16. doi: 10.1093/fampra/cmm027
23. Muller S, Thomas E, Dunn KM, et al. A prognostic approach to defining chronic pain across a range of musculoskeletal pain sites. *Clin J Pain* 2013;29(5):411-6. doi: 10.1097/AJP.0b013e318257099e
24. Rivara FP, Mackenzie EJ, Jurkovich GJ, et al. Prevalence of pain in patients 1 year after major trauma. *Arch Surg* 2008;143(3):282-7; discussion 88. doi: 10.1001/archsurg.2007.61
25. Organization WH. The International Classification of Functioning, Disability and Health (ICF). Geneva: WHO 2001.
26. Dixon D PD, Johnston M. What does the chronic pain grade questionnaire measure? *Pain* 2007;130:249-53.
27. Smith BH, Penny KI, Purves AM, et al. The Chronic Pain Grade questionnaire: validation and reliability in postal research. *Pain* 1997;71(2):141-7.
28. Penny KI PA, Smith BH, Chambers WA, Smith WC. Relationship between the chronic pain grade and measures of physical, social and psychological well-being. *pain* 1999;79:275–9.
29. Elliott AM SB, Smith WC, Chambers WA. Changes in chronic pain severity over time: The Chronic Pain Grade as a valid measure. *Pain* 2000;88:303.
30. Von Korff M, Jensen MP, Karoly P. Assessing global pain severity by self-report in clinical and health services research. *Spine (Phila Pa 1976)* 2000;25(24):3140-51.
31. Von Korff M, Miglioretti DL. A prognostic approach to defining chronic pain. *Pain* 2005;117(3):304-13. doi: 10.1016/j.pain.2005.06.017
32. Von Korff M, Dunn KM. Chronic pain reconsidered. *Pain* 2008;138(2):267-76. doi: 10.1016/j.pain.2007.12.010
33. Dunn KM, Croft PR, Main CJ, et al. A prognostic approach to defining chronic pain: replication in a UK primary care low back pain population. *Pain* 2008;135(1-2):48-54. doi: 10.1016/j.pain.2007.05.001
34. Thomas E, Dunn KM, Mallen C, et al. A prognostic approach to defining chronic pain: application to knee pain in older adults. *Pain* 2008;139(2):389-97. doi: 10.1016/j.pain.2008.05.010
35. Eriksen HR, Ihlebaek C, Ursin H. A scoring system for subjective health complaints (SHC). *Scand J Public Health* 1999;27(1):63-72.
36. Ware J. SF-36 Health Survey Updated. *Spine* 2000;25(24):3130-39.
37. Brooks R. EuroQol: the current state of play. *Health Policy* 1996;37(1):53-72.
38. Mahoney FI, Barthel DW. Functional Evaluation: The Barthel Index. *Md State Med J* 1965;14:61-5.
39. Cappelleri JC BA, McDermott AM, Sadosky AB, Petrie CD, Martin S. Psychometric properties of a single-item scale to assess sleep quality among individuals with fibromyalgia. *Health Qual Life Outcomes* 2009;17(7):54.
40. Zigmond AS, Snaith RP. The hospital anxiety and depression scale. *Acta Psychiatr Scand* 1983;67(6):361-70.
41. Harland NJ Georgieff K. Development of the coping strategies questionnaire 24, a clinically utilitarian version of the coping strategies questionnaire. *Rehabilitation Psychology* 2003;48(4):296–300.
42. Woby SR, Roach NK, Urmston M, et al. Psychometric properties of the TSK-11: a shortened version of the Tampa Scale for Kinesiophobia. *Pain* 2005;117(1-2):137-44. doi: 10.1016/j.pain.2005.05.029
43. Nicholas MK. The pain self-efficacy questionnaire: Taking pain into account. *Eur J Pain* 2007;11(2):153-63.

For peer review only

For peer review only - http://bmjopen.bmj.com/site/about/guidelines.xhtml

44. Beck JG, Grant DM, Read JP, et al. The impact of event scale-revised: psychometric properties in a sample of motor vehicle accident survivors. *J Anxiety Disord* 2008;22(2):187-98. doi: 10.1016/j.janxdis.2007.02.007
45. Baker SP, O'Neill B, Haddon W, Jr., et al. The injury severity score: a method for describing patients with multiple injuries and evaluating emergency care. *J Trauma* 1974;14(3):187-96.
46. Masters Steedman S, Middaugh SJ, Kee WG, et al. Chronic-pain medications: equivalence levels and method of quantifying usage. *Clin J Pain* 1992;8(3):204-14.
47. Harden RN, Weinland SR, Remble TA, et al. Medication Quantification Scale Version III: update in medication classes and revised detriment weights by survey of American Pain Society Physicians. *J Pain* 2005;6(6):364-71. doi: 10.1016/j.jpain.2005.01.350
48. Gallizzi M GC, Harden RN, Stanos S, Khan A. Medication Quantification Scale Version III: internal validation of detriment weights using a chronic pain population. . *Pain Pract* 2008;8(1):1-4.
49. Barbero M, Mores, F., Leoni, D., Gatti, R., Egloff, M., Falla, D. Test-retest reliability of pain extent and pain location using a novel method for pain drawing analysis. *European Journal of Pain* 2015;19:1129-38.
50. Freynhagen R, Baron R, Gockel U, et al. painDETECT: a new screening questionnaire to identify neuropathic components in patients with back pain. *Curr Med Res Opin* 2006;22(10):1911-20. doi: 10.1185/030079906X132488
51. Peduzzi P, Concato J, Kemper E, et al. A simulation study of the number of events per variable in logistic regression analysis. *J Clin Epidemiol* 1996;49(12):1373-9.
52. Vittinghoff E, McCulloch CE. Relaxing the rule of ten events per variable in logistic and Cox regression. *Am J Epidemiol* 2007;165(6):710-8. doi: 10.1093/aje/kwk052
53. Sainani KL. Multivariate Regression: The Pitfalls of Automated Variable Selection. *Physical Medicine and Rehabilitation* 2013;5:791-94,.
54. Sterne JA, White IR, Carlin JB, et al. Multiple imputation for missing data in epidemiological and clinical research: potential and pitfalls. *BMJ* 2009;338:b2393. doi: 10.1136/bmj.b2393
55. Woolf CJ, Bennett GJ, Doherty M, et al. Towards a mechanism-based classification of pain? *Pain* 1998;77(3):227-9.
56. Kosek E CM, Baron R, Gebhart GF, Mico JA, Rice AS, Rief W, Sluka AK. Do we need a third mechanistic descriptor for chronic pain states? . *Pain* 2016;157(7):1382-6.
57. NICE. Rehabilitation after critical illness in adults Clinical guideline [CG83]. 2009
58. van Oort L, van den Berg T, Koes BW, et al. Preliminary state of development of prediction models for primary care physical therapy: a systematic review. *J Clin Epidemiol* 2012;65(12):1257-66. doi: 10.1016/j.jclinepi.2012.05.007
59. Hazeldine J, Naumann DN, Toman E, et al. Prehospital immune responses and development of multiple organ dysfunction syndrome following traumatic injury: A prospective cohort study. *PLoS Med* 2017;14(7):e1002338. doi: 10.1371/journal.pmed.1002338

Authors' contributions

DF is Chief Investigator and guarantor. DF and AR led protocol development are leading data analysis and dissemination. DE is the Research Fellow with responsibility for study management. NM is a Doctoral Researcher focused to this study. NH is the lead for Patient and Public Involvement. JP is the Principal Investigator at the Major Trauma Centre. JP and CS are clinical representatives at the Major Trauma Centre. JL is the lead for biomarker

For peer review only - http://bmjopen.bmj.com/site/about/guidelines.xhtml

evaluation. All authors will contribute to data interpretation, conclusions, and dissemination. AR drafted the initial manuscript with DF. Subsequent drafts were developed with DE. All reviewers have read, contributed to, and agreed upon the final manuscript.

Funding statement

This work was supported by the National Institute for Health Research Surgical Reconstruction and Microbiology Research Centre [NIHR SRMRC] to DF and AR.

Competing interests

There are no competing interests.

Data sharing statement

No additional data are available.

For peer review only

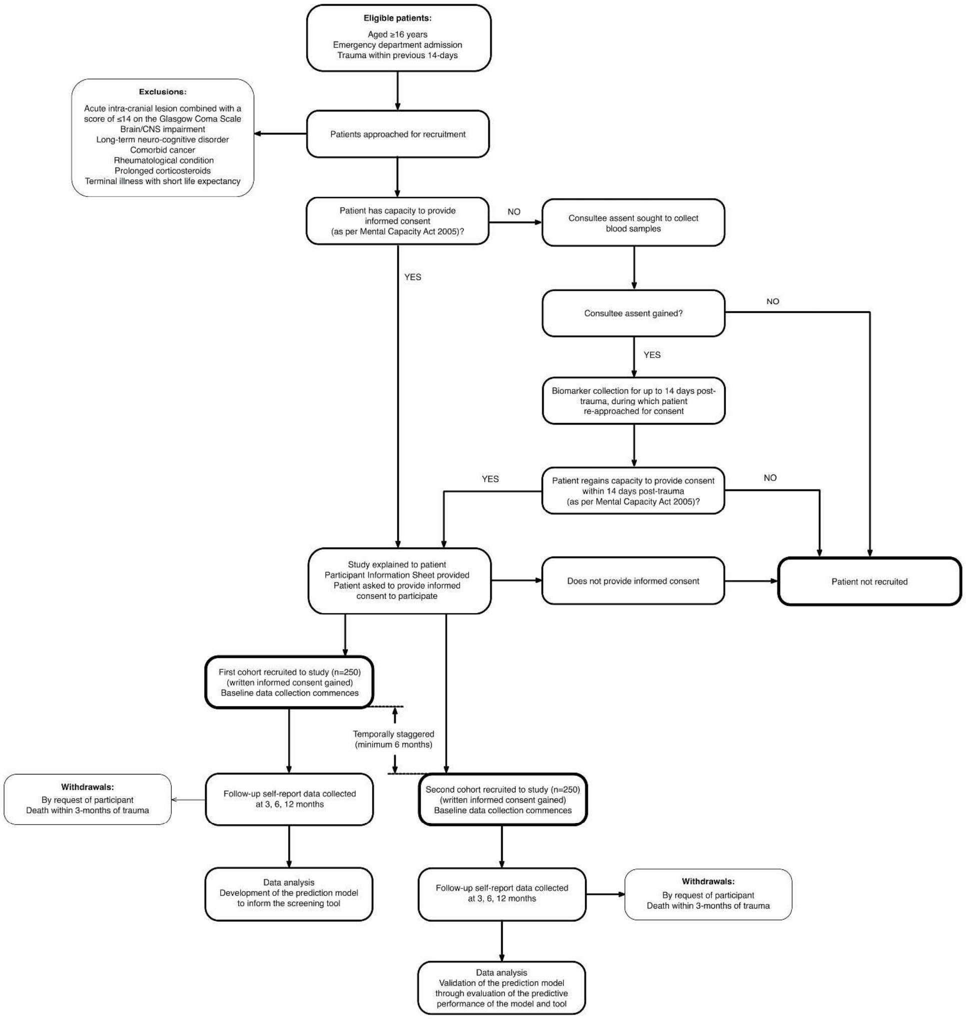

Study design

500x521mm (300 x 300 DPI)

Supplementary file 1. Candidate predictors

General participant characteristics

Several participant demographic features will be recorded at baseline, based on available hospital records and patient self-reported recollection, including smoking status, age, gender, height and weight to calculate body mass index [BMI], education [highest attained educational level], employment status [at the time of trauma], circumstance of trauma [military or civilian], previous history of musculoskeletal pain and injury, comorbidity of other current health problems.

Quality of life and physical functioning

36-Item Short Form Health Survey, Version 2.0 [SF-36v2]

The SF-36v2 is a self-reported measure of health-related quality of life, modified from the original SF-36, which was developed as part of the Medical Outcomes Study.[1] The 36-item questionnaire has subscales that assess physical function, social and psychological wellbeing.[2 3] The scores can be divided into physical and mental component summary scales.[4] The SF-36 has been shown to be valid and has been tested extensively in a trauma population.[5] Ware[6] reports multiple studies showing internal consistency above 0.70, with physical and mental scores exceeding 0.90. Minimal clinically important difference has been reported as 5.5 in a musculoskeletal trauma population.[7] Introduced in 1996, version 2.0 of the SF-36 is comparable to the original, retaining all subscales, with improvements to layout, presentation, response scales, wording and scoring.[6] The 'acute' [1 week recall period] version will be used, since the 4 week recall would not be appropriate for post-injury recall at baseline.

For peer review only - http://bmjopen.bmj.com/site/about/guidelines.xhtml

EuroQol Five Dimension Scale, 5-level [EQ-5D-5L]

Health-related quality of life will be quantified using the EQ-5D-5L through which 243 possible health states are converted to a single index value of range 0 to 1 where 1 is perfect health, and a visual analogue scale range 0–100, representing ‘worst’ to ‘best’ imaginable health state, respectively.[8] The EQ-5D-5L, with each item having 5 possible responses, has improved inter-observer [ICC 2,1 0.57] and test-retest [ICC 2,1 0.69] reliability compared to the previous EQ-5D-3L.[9] In addition, it has less ceiling effects [20.8% reduction] and adequate convergent validity when compared with the WHO-5 [spearman rank 0.38-0.51].[10]

Barthel Index of Activities of Daily Living

The Barthel Index of Activities of Daily Living is routinely collected by clinical staff at the hospital, and will be used to evaluate self-care and mobility during activities of daily living.[11 12] It is a 10-item ordinal scale encompassing a range of mobility physical activity tasks. Each item is related to a specific task and rated with a given number of points. A score of ‘0’ is given for least independence/function on that item and scores above that [1 or 2] are given for increasing independence/function [range: 0-20]. The amount of time and physical assistance required to perform each task are used in determining the assigned value of each item. A higher score is associated with a greater likelihood of being able to live at home with a degree of independence following discharge from hospital. With most measurement testing performed in the stroke population, the Barthel Index has demonstrated excellent internal consistency [0.89-0.90][13] and is highly responsive in detecting changes[14] with a minimal detectable change of 4.02 and minimally clinically important difference of 1.85.[15] High correlations have been demonstrated with the Functional Independence Measure [FIM], indicating convergent validity of the instrument.[16]

For peer review only - http://bmjopen.bmj.com/site/about/guidelines.xhtml

Subjective Health Complaints Inventory

Premorbid subjective health complaints will be assessed for the 6 months preceding the traumatic injury, using the single-item questions for non-somatic domains from the Subjective Health Complaints Inventory.[17] The Subjective Health Complaints Inventory has been shown to be a reliable measure of recording subjective health complaints for a 30 day recall period,[17] although psychometric properties have not been reported for an extended 6 month recall period.

Sleep quality

Current sleep quality [over the previous 24 hour period] will be assessed using an 11-point Numerical Rating Scale [NRS], ranging from 0 [‘best possible sleep’] to 10 [‘worst possible sleep’]. This scale has been shown to possess moderate psychometric properties in fibromyalgia patients using a symptom diary.[18] We will aim to assess current pain intensity at baseline, as frequently as every 48-hours while the patient is in hospital up to a maximum of 14 days following recruitment [depending upon patient accessibility and assessor availability], to gain accurate average and rate-of-change data. In addition, we will use the 0-10 NRS to assess average sleep quality, related to the preceding 6-months at the 6 and 12-month assessment points, although no psychometric properties have previously been reported for this recall period.

Psychosocial features

The predictive strength of psychosocial factors demonstrated in both primary care,[19,20] and post-trauma pain literature[21-24] demonstrates the importance of including these domains as candidate predictors.

For peer review only - http://bmjopen.bmj.com/site/about/guidelines.xhtml

The Hospital Anxiety and Depression Scales [HADS]

The HADS will be used to measure depression and anxiety, and their role in the manifestation of somatic symptoms.[25] There are 7 items which produce a cumulative score [range 0–21] for the anxiety [HADS-A] and depression [HADS-D] subscales, with a higher score indicative of greater anxiety and depression.[26] HADS has been tested in multiple populations demonstrating adequate to excellent internal consistency of HADS-A [0.68-0.93] and HADS-D [0.67-0.90].[26] Standard measurement of error in a coronary heart disease population was identified as 1.37 and 1.44 for anxiety and depression scales respectively, and minimal detectable change as 3.80 and 3.99 respectively.[27] The HADS has also demonstrated excellent concurrent validity when compared to various other depression/anxiety scales.[26]

Coping Strategies Questionnaire 24 [CSQ-24]

The CSQ-24 will be used to provide an indication of coping strategies used by participants when they are in pain.[28] Developed from items from the earlier, much larger Coping Strategies Questionnaire,[29] the CSQ-24 is a 23-item scale, composed of 4 subscales: *catastrophizing* [6 items], *diversion* [6 items], *reinterpreting* [6 items], and *cognitive coping* [5 items]. Participants are asked to indicate if they have particular thoughts and feelings when they are experiencing pain. A score on each item is summed to yield an aggregate score for each subscale, with a higher score reflecting greater attribution of that particular coping strategy. The CSQ-24 has demonstrated good internal consistency in populations with low back pain patients [Chronbach's alpha for the 4 factors ranged from 0.75 to 0.85] and work-related pain [0.80 to 0.86].[30] Harland & Georgieff[28] suggested that, since individuals may have a positive score on more than one subscale, the highest scoring subscale should be deemed the dominant coping strategy.[28] However, a recent study in a low back pain cohort,[31]

For peer review only - http://bmjopen.bmj.com/site/about/guidelines.xhtml

in which individual items from multiple questionnaires were factorised, suggested that *diversion*, *reinterpreting* and *cognitive coping* clustered together as a single factor, representing coping cognitions. By contrast, *catastrophizing* clustered with pain-related distress items.

Tampa Scale of Kinesiophobia [TSK-11]

The TSK-11 will be used to assess fear of movement or fear of injury or re-injury during movement.[32] It is an 11-item questionnaire, eliminating psychometrically poor items from its original 17-item version,[33] thus creating a shorter questionnaire with comparable internal consistency and a 2-factor structure [activity avoidance and harm]. Each of the 11 items is measured using a 4-point scale using the end points 1 [‘totally agree’] and 4 [‘totally disagree’] [scoring range 11–44]. Higher scores indicate more fear-avoidance behaviour. The TSK-11 has demonstrated acceptable to good internal consistency in acute and chronic musculoskeletal pain populations.[32 34] Test-retest reliability has been reported as excellent with a high standardised response mean; with good construct validity in relation to changes in disability and pain.[32]

Pain Self-Efficacy Questionnaire [PSEQ]

The patient’s confidence in their ability to perform activities despite their pain will be evaluated using the PSEQ. Developed from the Self-Efficacy Scale,[35] the PSEQ consists of 10 physical and psychosocial activity items measuring from 0 [‘not at all confident’] to 6 [‘completely confident’] thus generating a total score from 0-60.[36] The PSEQ has demonstrated excellent internal consistency [0.92], internal reliability [0.93], and test-re-test correlations [r=0.73] and has demonstrated validity when compared to other self-efficacy

For peer review only - http://bmjopen.bmj.com/site/about/guidelines.xhtml

measurements.[36] It has been used in several large population studies, for example Campbell et al.[31]

Impact of Event Scale Revised [IES-R]

The IES-R will be used to measure the subjective stress experienced by the participant following their traumatic event. The IES-R is a 22-item tool [range: 0-88] that consists of 8 intrusion and 8 avoidance items that are derived from the original IES,[37] with an additional 7-items assessing hyperarousal.[38] Accordingly, items correspond directly to symptoms of post-traumatic stress disorder.[38] Respondents are asked to identify a specific stressful life event and then indicate how much they were distressed or bothered during the past seven days by each ‘difficulty’ listed. Each item is rated on a 5-point scale ranging from 0 [‘not at all’] to 4 [‘extremely’]. The IES-R yields a total score ranging 0 to 88 and subscale scores can also be calculated for the Intrusion, Avoidance, and Hyperarousal sub-scales. The IES-R has demonstrated good internal consistency for all subscales [intrusion 0.87-0.94, avoidance 0.84-0.97, hyperarousal 0.79-0.91].[39] High correlations have been found between the IES-R and the original scale, supporting the concurrent validity of both measures.[38]

Injury characteristics

Several measures relating to the characteristics of the sustained injury will be taken at baseline, since it is plausible that some of these should possess predictive value.[40] The time of the injury will be gained from hospital records. The location of the injury/injuries will be recorded using an adapted version of previously developed pain drawing software, via a tablet computer.[41] Information relating to the tissues damaged from the injury [e.g. fractures sustained, whether the injury was penetrating, non-penetrating or both, review of available imaging data] will be gathered from hospital records, where possible. Whether the participant

For peer review only - http://bmjopen.bmj.com/site/about/guidelines.xhtml

received surgery following their admission [where, for what and when], and whether the participant received assisted mechanical ventilation will also be recorded.

Injury Severity Scale [ISS]

The ISS will be retrospectively calculated for each participant, including those who withdraw. The ISS is a numerical score with a range 0-75, that is used to describe the overall severity of injury, and can be used for both multiple and single injuries. The score is calculated, based on the Abbreviated Injury Scale [AIS] scores.[42 43] Higher ISS scores have been associated with increased rates of mortality[42 44 45] and length/cost of hospital stay.[46] It is the recommended tool for summarising injury severity by the Trauma Audit and Research Network [TARN]. Both TARN and the National Institute for Clinical Excellence[47] recommend any participant with a score of >8 to be referred for rehabilitation.

Pain characteristics

Pain characteristics [e.g. pain intensity, multi-site pain] have long been reported to hold predictive value for long-term pain across a variety of conditions.[19-21 48 49] It is therefore sensible that we include these domains as candidate predictors for post-trauma pain.

Pain intensity

Pain intensity will be measured using an 11-point [0-10] Numerical Rating Scale [NRS], measuring current pain from ‘no pain’ to ‘pain as bad as could be’, from the Chronic Pain Grade Scale.[50] We will aim to assess *current* pain intensity at baseline, as frequently as every 48-hours while the participant is in hospital [depending upon participant accessibility and

For peer review only - http://bmjopen.bmj.com/site/about/guidelines.xhtml

assessor availability], to gain accurate mean and rate-of-change data. At the 6 and 12 month assessment points, c*urrent* pain intensity, as well as *average* and *worst* pain intensity related to the preceding 6 months, will be collected as part of the Chronic Pain Grade Scale. NRS scales are sensitive, reliable and valid instruments for pain intensity measurement,[51-54] and have been recommended for use in clinical populations in preference to visual analogue scales or verbal rating scales.[55] A 30% change on a pain NRS score is considered clinically meaningful.[56-60]

<u>Pain medication</u>

The patient's pain medication [type, dosage and time since trauma] intake will be noted and the Medication Quantification Score [MQS], which is a reliable and validated score for quantifying analgesics, will be calculated to obtain a comparable metric for all different analgesics.[61-63] It enables characterisation of analgesics when many different medications are involved and doses are irregular. It will be calculated for each non-opioid and opioid, based on weights assigned by medication class and dosage level [level 1 = sub-therapeutic dosage and/or on demand, level 2 = lower 50% of the therapeutic dose range, level 3 = upper 50% of the therapeutic dose range, level 4 = supra-therapeutic dose] using the 1998 detriment weights.[64] The detriment weights are summed by the dosage level to provide the final score. These scores will be summed to provide a quantitative index for analgesic usage suitable for statistical analysis.

<u>Pain drawing</u>

All participants will be requested to complete a pain drawing, indicating the spatial distribution of their pain, over two body charts; one reporting a frontal view of the body and one a dorsal view. We will also ask patients to mark their single 'most painful' site on one of

For peer review only - http://bmjopen.bmj.com/site/about/guidelines.xhtml

For peer review only

these body charts. Pain drawing data will be collected using a custom software application on a tablet computer, and will be analysed with Matlab software, as described previously.[41] The software automatically calculates the number of shaded pixels from the pain drawing, which is defined as *pain extent*. Summaries of, and relationships between, pre-defined painful body regions will also be evaluated. Conventional pain drawing data will also be collected on paper follow-up questionnaires to assess painful body regions.

<u>The painDETECT questionnaire</u>

It is assumed that all post-trauma patients will have significant nociception at baseline, but given the relatively high proportion of neuropathic pain following traumatic injury,[65] the contribution of other pain-related mechanistic pathways should also be assessed. The painDETECT questionnaire[66] will be used to facilitate the identification of neuropathic pain. It consists of 9 items [7 evaluating pain quality, 1 evaluating pain pattern, and 1 evaluating pain radiation], all of which contribute to an aggregate score [range: -1-38]. This aggregate score can be divided into three classifications that represent the likelihood of neuropathic pain: 'unlikely' [0-12], 'ambiguous' [13–18] and 'likely' [19–38].[66] Although developed as a screening questionnaire for neuropathic pain, painDETECT may also function as a measure of characteristics that indicate augmented central pain processing.[66] The painDETECT questionnaire has demonstrated good internal consistency [0.76][67] and excellent test-re-test reliability[68] within 1-hour of consultation [ICC{model not reported} 0.911] and 1-week post consultation [ICC{model not reported} 0.79].[69] Convergent validity has been demonstrated in comparison to pain severity,[67 70] health-related quality of life[71] and similar neuropathic pain screening tools.[66] As such, painDETECT outcomes will be measured at regular intervals while the participant is an inpatient in the hospital [subject to participant accessibility and

For peer review only - http://bmjopen.bmj.com/site/about/guidelines.xhtml

assessor availability] to assess for emerging neuropathic pain and sensitization. Measurements will also be taken at all follow-up assessment points.

Quantitative sensory testing [QST]

QST methods will be used to assess pain sensibility, throughout which measurements will be concealed from participants. Owing to the clinical heterogeneity of the post-trauma population, precise standardisation of test sites between participants will not be possible. Instead, we have developed a standardised protocol that will be used to evaluate pain thresholds for multiple stimulus modalities [mechanical pressure, heat and cold] at the same sites in each participant. Each site where multi-modality pain threshold testing is performed will be within the receptive field of the same nerve root using described regions,[72] so that segmental cross-modality excitability may be compared. All pain thresholds will be measured at the same 'local' and 'remote' sites for each participant. We define local sites as being uninjured but within [or, if not accessible, as close as possible to] the same receptive field as the most painful inured tissue [e.g. skin over gastrocnemius in a participant with an ankle fracture]. By contrast, we define remote sites as a distant, accessible, site from the receptive fields in which tissues are injured [e.g. skin over tibialis anterior in a participant without lower limb injury], and on the contralateral side of the body where injured tissue is unilateral. Where possible, remote sites will be a mirror-image of the local site [to allow for comparison of absolute values], but in a trauma population we are aware that this may not always be possible. For all threshold testing modalities, an ascending method of limits design[73] will be used, whereby stimulus intensity will begin at a low level and gradually increase until the participant first perceives pain. Participants will be instructed to push a button or tell the assessor when the sensation has changed from one of the stimulus alone [e.g. just pressure] to

For peer review only - http://bmjopen.bmj.com/site/about/guidelines.xhtml

a sensation of both the stimulus and pain [e.g. pressure and pain]. Following a brief demonstration of equipment to familiarise participants, two consecutive assessments will be performed for each modality at each site, and the means used for further analysis.[74] A minimum of 30-seconds inter-stimulus interval will be given between each threshold measurement within a single session. Measurements will be taken at baseline, while the participant is an inpatient in the hospital; we will aim to collect data as frequently as every 48 hours to gain accurate rate-of-change data, but this will depend upon participant accessibility and assessor availability. To ensure pain thresholds are consistently measured at the same sites every session, sites will be labelled using a sterile, skin marking pen [Schuco Ltd, UK]. Because sites cannot be standardised between participants, the rates-of-change of these values will be used as candidate predictive variables, to allow for comparisons between participants. The order of pain threshold testing will be randomly assigned by modality at each session to avoid order effects.

Thermal [heat and cold] pain thresholds will be measured using skin-contact stimulation, using the same thermode at the same sites, within specified local and remote dermatomes. Thermal pain threshold assessments will be performed by delivering thermal stimuli directly to the skin through a metal 30x30 mm Peltier thermode, using a TSA-II NeuroSensory Analyzer thermal stimulator and accompanying software [Medoc Ltd, Israel]. To evaluate heat pain threshold, temperature will be gradually increased, at a rate of 1°C/s from a 'neutral' baseline of 32°C, to a maximum temperature of 50.5°C to avoid thermal injury.[75] During each measurement, participants will be instructed to press a button when the stimulus becomes painful, and this will be documented as the threshold value. Once pain threshold is achieved [and recorded], the temperature will return to the baseline value at the same rate [1°C/s]. For cold pain threshold measurements, the temperature will be gradually reduced, at

For peer review only - http://bmjopen.bmj.com/site/about/guidelines.xhtml

a rate of 1°C/s from the baseline of 32°C, to a minimum temperature of 0°C,[75] before also returning to baseline at a rate of 1°C/s.

Pressure pain thresholds will be measured using a digital pressure algometer [Series 7 force gauge, Mark-10 Corporation, USA], providing real-time force measurement and an analogue output that can be linked to a computer. Skin and muscle tissue are simultaneously stimulated during pressure threshold testing; sites will therefore be chosen where a dermatome and myotome are likely to share a common nerve root innervation [e.g. skin over tibialis anterior]. The algometer has a hard rubber circular contact tip of 1.2cm^2 area, with no sharp edges so to avoid an uneven pressure stimulus.[76] In order to preserve hygiene and attend to infection control measures in trauma patients, the contact tip will be covered with a clean, thin disposable covering. The tip will be applied perpendicular to the skin at a constant rate of pressure increase of 50kPa/s [6.0N/s using the 1.2cm^2 tip], until the first onset of pain. For each measurement, pressure will be unloaded immediately once the participant indicates that their pain threshold has been reached.[74]

To measure excitability of nociceptive pathways in response to mechanical stimuli, a series of repetitive, pressure stimulus 'pulses' will be applied via the digital algometer, with the aim of provoking temporal summation responses.[77-79] A minimum of 2 minutes after all threshold tests have been completed, a series of 10 consecutive pressure pulses will be applied at the remote and local sites [the order of site being randomly assigned]. The peak pressure reached during each pulse will be the mean pressure pain threshold that was measured for that particular site, as described previously. For each pulse, pressure will be gradually increased to the peak value over a period of 5 seconds, maintained at that peak value for 1 second, and then immediately released. A 5 second inter-stimulus interval will be used between pulses,

For peer review only - http://bmjopen.bmj.com/site/about/guidelines.xhtml

during which the tip of the algometer will remain in contact with the skin.[78 79] Pain intensity from the pulses will be rated on a numerical rating scale [0 being 'no pain' to 10 being 'pain as bad as could be']. In the event that participants indicate that pain has become intolerable, the sequence will be stopped immediately, and the NRS score and number of impulses performed at that point will be noted.

Biomarkers

Serum levels of C-reactive protein [CRP] will be used as a biomarker for inflammation; one of the primary mechanistic pathways that can evoke pain.[80] CRP is an acute-phase response protein produced by hepatocytes and is usually found in concentrations of 0.3 to 1.7 mg/l[81]. Increased production is due to cytokine-dependent induction of synthesis and elevated levels may be detected within eight hours of a stimulus and can reach 500 mg/1.28. Besides trauma,[82] elevated levels of CRP may be seen in conditions such as autoimmune disease, infection and malignancy. It has also been associated with acute sciatica.[83] The level of CRP usually peaks within 48 hours of the stimulus. In contrast, when the stimulus for increased production completely ceases, the circulating CRP concentration falls rapidly, at almost the rate of plasma CRP clearance.[84] A fall in serial measurements usually indicates resolution of the underlying process, while persisting elevated levels indicates ongoing inflammation.[85] Where possible, measurements of serum CRP will be repeatedly taken on a 48 hour schedule while the participant is an inpatient; absolute and rates of change of CRP values will be used as candidate predictive factors.[86]

Plasma cell-free DNA [cfDNA] will be used as an indicator of tissue damage. This includes both nuclear DNA [nDNA] and mitochondrial DNA [mtDNA], which circulate after being

For peer review only - http://bmjopen.bmj.com/site/about/guidelines.xhtml

released from cells when they are damaged and are thought to be amongst the important initiators of systemic inflammatory responses following tissue injury known as Damage Associated Molecular Patterns [DAMPs]. [87] Clinical outcomes in trauma patients have been related to plasma mtDNA concentration.[88 89] Other work with severe trauma patients has shown that cfDNA values rise to their peak value in the second week post-trauma, and then gradually return to baseline values after approximately 2 months.[90] Where possible, measurements of circulating cfDNA will be repeatedly taken on a 48 hour schedule while the participant is an inpatient; absolute values and rates of change of cfDNA values will be used as candidate predictive factors.

For peer review only - http://bmjopen.bmj.com/site/about/guidelines.xhtml

References

1. Stewart AL, Greenfield S, Hays RD, et al. Functional status and well-being of patients with chronic conditions. Results from the Medical Outcomes Study. *JAMA* 1989;262(7):907-13.
2. Ware JE, Jr., Sherbourne CD. The MOS 36-item short-form health survey (SF-36). I. Conceptual framework and item selection. *Med Care* 1992;30(6):473-83.
3. Hays RD, Sherbourne CD, Mazel RM. The RAND 36-Item Health Survey 1.0. *Health Econ* 1993;2(3):217-27.
4. McHorney CA, Ware JE, Jr., Raczek AE. The MOS 36-Item Short-Form Health Survey (SF-36): II. Psychometric and clinical tests of validity in measuring physical and mental health constructs. *Med Care* 1993;31(3):247-63.
5. Revell MP, Pynsent, P.B., Abudu, A., Fairbank, J.C.T. . Trauma Scores and Trauma Outcome Measures *Trauma* 2003;5:61-70.
6. Ware J. SF-36 Health Survey Updated. *Spine* 2000;25(24):3130-39.
7. Dattani R, Slobogean, G.P., O'Brien, P.J., Broekhuyse, H.M., Blachut, P.A., Guy, P., Lefaivre, K.A. Psychometric Analysis of Measuring Functional Outcomes in Tibial Plateau Fractures using the Short Form (SF-36), Short Musculoskeletal Function Assessment (SMFA) and the Western Ontario McMaster Osteoarthritis (WOMAC) Questionnaires. *Injury Int J Care Injured* 2013;44:825-29.
8. Brooks R. EuroQol: the current state of play. *Health Policy* 1996;37(1):53-72.
9. Janssen MF, Birnie E, Haagsma JA, et al. Comparing the standard EQ-5D three-level system with a five-level version. *Value Health* 2008;11(2):275-84. doi: 10.1111/j.1524-4733.2007.00230.x
10. Janssen MF, Pickard AS, Golicki D, et al. Measurement properties of the EQ-5D-5L compared to the EQ-5D-3L across eight patient groups: a multi-country study. *Qual Life Res* 2013;22(7):1717-27. doi: 10.1007/s11136-012-0322-4
11. Mahoney FI, Barthel DW. Functional Evaluation: The Barthel Index. *Md State Med J* 1965;14:61-5.
12. Shah S, Vanclay F, Cooper B. Improving the sensitivity of the Barthel Index for stroke rehabilitation. *J Clin Epidemiol* 1989;42(8):703-9.
13. Hseuh IP, Lee, M.M., Hsieh, C.L. . Psychometric Characteristics of the Barthel Activities of Daily Living Index in Stroke Patients *J Formos Med Assoc* 2001;100(526-532)
14. Hseuh IP, Lin, J.H., Jeng, J.S., Hsieh, C.L. Comparison of the Psychometric Characteristics of the Functional Independence Measure, 5 Item Barthel Index and 10 Item Barthel Index in Patients with Stroke. *J Neurol Neurosurg Psychiatry* 2002;73:188-90.
15. Hsieh YW, Wang, C.H., Wu, S.C., Chen, P.C., Sheu, C.F., Hsieh, C.L. . Establishing the Minimally Clinically Important Difference of the Barthel Index in Stroke Patients. *Neurorehabilitation and Neural Repair* 2007;21(3):233-38.
16. Hobart JC, Thompson AJ. The five item Barthel index. *J Neurol Neurosurg Psychiatry* 2001;71(2):225-30.
17. Eriksen HR, Ihlebaek C, Ursin H. A scoring system for subjective health complaints (SHC). *Scand J Public Health* 1999;27(1):63-72.
18. Cappelleri JC BA, McDermott AM, Sadosky AB, Petrie CD, Martin S. Psychometric properties of a single-item scale to assess sleep quality among individuals with fibromyalgia. *Health Qual Life Outcomes* 2009;17(7):54.
19. Mallen CD, Peat G, Thomas E, et al. Prognostic factors for musculoskeletal pain in primary care: a systematic review. *Br J Gen Pract* 2007;57(541):655-61.
20. Artus M, Campbell P, Mallen CD, et al. Generic prognostic factors for musculoskeletal pain in primary care: a systematic review. *BMJ Open* 2017;7(1):e012901. doi: 10.1136/bmjopen-2016-012901

For peer review only - http://bmjopen.bmj.com/site/about/guidelines.xhtml

21. Clay FJ, Newstead SV, Watson WL, et al. Bio-psychosocial determinants of persistent pain 6 months after non-life-threatening acute orthopaedic trauma. *J Pain* 2010;11(5):420-30. doi: 10.1016/j.jpain.2009.12.002
22. Archer KR, Castillo RC, Wegener ST, et al. Pain and satisfaction in hospitalized trauma patients: the importance of self-efficacy and psychological distress. *J Trauma Acute Care Surg* 2012;72(4):1068-77. doi: 10.1097/TA.0b013e3182452df5
23. Rosenbloom BN, Khan S, McCartney C, et al. Systematic review of persistent pain and psychological outcomes following traumatic musculoskeletal injury. *J Pain Res* 2013;6:39-51. doi: 10.2147/JPR.S38878
24. Vranceanu AM, Bachoura A, Weening A, et al. Psychological factors predict disability and pain intensity after skeletal trauma. *J Bone Joint Surg Am* 2014;96(3):e20. doi: 10.2106/JBJS.L.00479
25. Zigmond AS, Snaith RP. The hospital anxiety and depression scale. *Acta Psychiatr Scand* 1983;67(6):361-70.
26. Bjelland I, Dahl AA, Haug TT, et al. The validity of the Hospital Anxiety and Depression Scale. An updated literature review. *J Psychosom Res* 2002;52(2):69-77.
27. Wang W, Chair, S.Y., Thompson, D.R., Twinn, S.F. A Psychometric Evaluation of the Chinese Version of the Hospital Anxiety and Depression Scale in Patients with Coronary Heart Disease *Journal of Clinical Nursing* 2009;18:1908-15.
28. Harland NJ Georgieff K. Development of the coping strategies questionnaire 24, a clinically utilitarian version of the coping strategies questionnaire. *Rehabilitation Psychology* 2003;48(4):296–300.
29. Rosenstiel AK KF. The use of coping strategies in chronic low back pain patients: Relationship to patient characteristics and current adjustment. . *Pain* 1983;17: 33–44.
30. Chiu C JJ, Fujikawa M, Strand D, Cheing G, Lee G, Chan F Measurement Structure of the Coping Strategies Questionnaire-24 in a Sample of Individuals with Musculoskeletal Pain: A Confirmatory Factor Analysis. . *Rehabilitation Research, Policy, and Education* 2014;28(2):80-90.
31. Campbell P, Foster NF, Thomas E, Dunn KM. Prognostic Indicators of Low Back Pain in Primary Care: Five-Year Prospective Study. *J Pain* 2013;14(8):873–83.
32. Woby SR, Roach NK, Urmston M, et al. Psychometric properties of the TSK-11: a shortened version of the Tampa Scale for Kinesiophobia. *Pain* 2005;117(1-2):137-44. doi: 10.1016/j.pain.2005.05.029
33. Miller RP KS, Todd D. The Tampa Scale: a measure of kinesiophobia. *Clin J Pain* 1991;7(1):51-52.
34. Swinkels-Meewisse EJCM, Swinkels RAHM, Verbeek ALM, Vlaeyen JWS. Psychometric Propertieis of the Tampa Scale for Kinesiophobia and the Fear Avoidance Beliecs Questionnaire in Acute Low Back Pain *Manual Therapy* 2003;8(1):29-36.
35. Kall LB. Psychological determinants of quality of life in patients with whiplash associated disorders-a prospective study. *Disabil Rehabil* 2009;31(3):227-36. doi: 10.1080/09638280801912030
36. Nicholas MK. The pain self-efficacy questionnaire: Taking pain into account. *Eur J Pain* 2007;11(2):153-63.
37. Horowitz M, Wilner N, Alvarez W. Impact of Event Scale: a measure of subjective stress. *Psychosom Med* 1979;41(3):209-18.
38. Beck JG, Grant DM, Read JP, et al. The impact of event scale-revised: psychometric properties in a sample of motor vehicle accident survivors. *J Anxiety Disord* 2008;22(2):187-98. doi: 10.1016/j.janxdis.2007.02.007
39. Creamer M, Bell R, Failla S. Psychometric properties of the Impact of Event Scale - Revised. *Behav Res Ther* 2003;41(12):1489-96.

For peer review only - http://bmjopen.bmj.com/site/about/guidelines.xhtml

40. Walker-Bone K HN, Ntani G, Tinati T, Jones GT, Smith BH, Macfarlane GJ, Cooper C. . Chronic widespread bodily pain is increased among individuals with history of fracture: findings from UK Biobank. . *Arch Osteoporos* 2016;11:1.
41. Barbero M, Mores, Leoni D, Gatti R, Egloff M, Falla D. Test-retest reliability of pain extent and pain location using a novel method for pain drawing analysis. *European Journal of Pain* 2015;19:1129-38.
42. Baker SP, O'Neill B. The injury severity score: an update. *J Trauma* 1976;16(11):882-5.
43. Akmal M, Trivedi R, Sutcliffe J. Functional outcome in trauma patients with spinal injury. *Spine (Phila Pa 1976)* 2003;28(2):180-5. doi: 10.1097/01.BRS.0000041577.12233.96
44. Baker SP, O'Neill B, Haddon W, Jr., et al. The injury severity score: a method for describing patients with multiple injuries and evaluating emergency care. *J Trauma* 1974;14(3):187-96.
45. Linn S. The injury severity score--importance and uses. *Ann Epidemiol* 1995;5(6):440-6.
46. Sears JM, Blanar L, Bowman SM. Predicting work-related disability and medical cost outcomes: a comparison of injury severity scoring methods. *Injury* 2014;45(1):16-22. doi: 10.1016/j.injury.2012.12.024
47. NICE. Low back pain and sciatica in over 16s: assessment and management, NICE guideline [NG59]: National Institute for Health and Care Excellence, 2016.
48. Kamaleri Y, Natvig B, Ihlebaek CM, et al. Change in the number of musculoskeletal pain sites: A 14-year prospective study. *Pain* 2009;141(1-2):25-30. doi: 10.1016/j.pain.2008.09.013
49. Clay FJ, Watson WL, Newstead SV, et al. A systematic review of early prognostic factors for persisting pain following acute orthopedic trauma. *Pain Res Manag* 2012;17(1):35-44.
50. Von Korff M, Ormel J, Keefe FJ, et al. Grading the severity of chronic pain. *Pain* 1992;50(2):133-49.
51. Jensen MP, McFarland CA. Increasing the reliability and validity of pain intensity measurement in chronic pain patients. *Pain* 1993;55(2):195-203.
52. Jensen MP, Turner, J.A. and Romano, J.M. . What is the maximum number of levels needed in pain intensity measurement? . *Pain* 1994;58:387–92.
53. Jensen MP, Turner, L.R., Turner, J.A. and Romano, J.M. The use of multiple-item scales for pain intensity measurement in chronic pain patients. *Pain* 1996;67:35–40.
54. Jensen MP, Turner JA, Romano JM, et al. Comparative reliability and validity of chronic pain intensity measures. *Pain* 1999;83(2):157-62.
55. Von Korff M. Epidemiological and survey methods – assessment of chronic pain. In Turk, D.C. and Melzack, R., editors, Handbook of Pain Assessment. . New York, USA: Guildford Press 2001:603-18.
56. Farrar JT, Young JP, Jr., LaMoreaux L, et al. Clinical importance of changes in chronic pain intensity measured on an 11-point numerical pain rating scale. *Pain* 2001;94(2):149-58.
57. Salaffi F, Stancati A, Silvestri CA, et al. Minimal clinically important changes in chronic musculoskeletal pain intensity measured on a numerical rating scale. *Eur J Pain* 2004;8(4):283-91. doi: 10.1016/j.ejpain.2003.09.004
58. Childs JD, Piva SR, Fritz JM. Responsiveness of the numeric pain rating scale in patients with low back pain. *Spine (Phila Pa 1976)* 2005;30(11):1331-4.
59. Ostelo RW, Deyo RA, Stratford P, Waddell G, Croft P, Von Korff M, Bouter LM, Henrica C. Interpreting change scores for pain and functional status in low back pain: towards international consensus regarding minimal important change. *Spine* 2008;33(1):90-94.
60. Michener LA, Snyder AR, Leggin BG. Responsiveness of the numeric pain rating scale in patients with shoulder pain and the effect of surgical status. *J Sport Rehabil* 2011;20(1):115-28.
61. Masters Steedman S, Middaugh SJ, Kee WG, et al. Chronic-pain medications: equivalence levels and method of quantifying usage. *Clin J Pain* 1992;8(3):204-14.
62. Harden RN, Weinland SR, Remble TA, et al. Medication Quantification Scale Version III: update in medication classes and revised detriment weights by survey of American Pain Society Physicians. *J Pain* 2005;6(6):364-71. doi: 10.1016/j.jpain.2005.01.350

For peer review only - http://bmjopen.bmj.com/site/about/guidelines.xhtml

63. Gallizzi M GC, Harden RN, Stanos S, Khan A. Medication Quantification Scale Version III: internal validation of detriment weights using a chronic pain population. . *Pain Pract* 2008;8(1):1-4.
64. Stormo KJ, Kee WG, Steedham S, et al. Medication quantification scores and evaluation of patient pharmacology profiles. *Current Review of Pain* 1998;2(3):171–74.
65. Rosenbloom BN, Katz J, Chin KY, et al. Predicting pain outcomes after traumatic musculoskeletal injury. *Pain* 2016;157(8):1733-43. doi: 10.1097/j.pain.0000000000000580
66. Freynhagen R, Baron R, Gockel U, et al. painDETECT: a new screening questionnaire to identify neuropathic components in patients with back pain. *Curr Med Res Opin* 2006;22(10):1911-20. doi: 10.1185/030079906X132488
67. Cappelleri JC, Koduru V, Bienen EJ, et al. A cross-sectional study examining the psychometric properties of the painDETECT measure in neuropathic pain. *J Pain Res* 2015;8(8):159-67. doi: 10.2147/JPR.S80046
68. Keller T, Freynhagen R, Tolle TR, et al. A retrospective analysis of the long-term test-retest stability of pain descriptors of the painDETECT questionnaire. *Curr Med Res Opin* 2016;32(2):343-9. doi: 10.1185/03007995.2015.1125869
69. Tampin B, Bohne T, Callan M, et al. Reliability of the English version of the painDETECT questionnaire. *Curr Med Res Opin* 2017;33(4):741-48. doi: 10.1080/03007995.2017.1278682
70. Sadosky A, Koduru V, Bienen EJ, et al. Characterizing individual painDETECT symptoms by average pain severity. *Clinicoecon Outcomes Res* 2016;8:361-6. doi: 10.2147/CEOR.S105402
71. Cappelleri JC, Koduru V, Bienen EJ, et al. Mapping painDETECT, a neuropathic pain screening tool, to the EuroQol (EQ-5D-3L). *Qual Life Res* 2017;26(2):467-77. doi: 10.1007/s11136-016-1379-2
72. O'Brien M. Aids to the Examination of the Peripheral Nervous System, 5th Edition: Saunders (Elsevier) 2010.
73. Palmer ST, Martin DJ, Steedman WM, Ravey J, S. C-and Aδ-fibre mediated thermal perception: response to rate of temperature change using method of limits. . *Somatosensory & motor research* 2000;17(4):325-33.
74. Chesterton LS SJ, Wright CC, Foster NE. Interrater reliability of algometry in measuring pressure pain thresholds in healthy humans, using multiple raters. *Clin J Pain* 2007;23(9):760-6.
75. Knutti IA SM, Opsommer E. Test-retest reliability of thermal quantitative sensory testing on two sites within the L5 dermatome of the lumbar spine and lower extremity. *Neurosci Lett* 2014;5(579):157-62.
76. Greenspan JD, McGillis SL. Stimulus features relevant to the perception of sharpness and mechanically evoked cutaneous pain. *Somatosens Mot Res* 1991;8(2):137-47.
77. Sarlani E, Greenspan JD. Gender differences in temporal summation of mechanically evoked pain. *Pain* 2002;97(1-2):163-9.
78. Nie H, Arendt-Nielsen L, Andersen H, et al. Temporal summation of pain evoked by mechanical stimulation in deep and superficial tissue. *J Pain* 2005;6(6):348-55. doi: 10.1016/j.jpain.2005.01.352
79. Nie H, Arendt-Nielsen L, Madeleine P, et al. Enhanced temporal summation of pressure pain in the trapezius muscle after delayed onset muscle soreness. *Exp Brain Res* 2006;170(2):182-90. doi: 10.1007/s00221-005-0196-6
80. Woolf CJ, Bennett GJ, Doherty M, et al. Towards a mechanism-based classification of pain? *Pain* 1998;77(3):227-9.
81. Whicher JT ES. Acute phase proteins. *Hosp Update* 1990:899-905.
82. Gosling P, Dickson GR. Serum c-reactive protein in patients with serious trauma. *Injury* 1992;23(7):483-6.
83. Sturmer T, Raum E, Buchner M, et al. Pain and high sensitivity C reactive protein in patients with chronic low back pain and acute sciatic pain. *Ann Rheum Dis* 2005;64(6):921-5. doi: 10.1136/ard.2004.027045

For peer review only - http://bmjopen.bmj.com/site/about/guidelines.xhtml

84. Pepys MB, Hirschfield GM. C-reactive protein: a critical update. *J Clin Invest* 2003;111(12):1805-12. doi: 10.1172/JCI18921

85. Giannoudis PV HF, Pape HC. . Inflammatory serum markers in patients with multiple trauma. Can they predict outcome? *J Bone Joint Surg Br* 2004;86(3):313-23.

86. Barma M, Goodbrand, J.A., Donnan, P.T., McGilchrist, M.M., Frost, H., McMurdo, M.E. and Witham, M.D. Slower Decline in C-Reactive Protein after an Inflammatory Insult Is Associated with Longer Survival in Older Hospitalised Patients. . *PloS one* 2016;11(7):e0159412.

87. Krysko DV, Agostinis P, Krysko O, et al. Emerging role of damage-associated molecular patterns derived from mitochondria in inflammation. *Trends Immunol* 2011;32(4):157-64. doi: 10.1016/j.it.2011.01.005

88. Simmons JD, Lee YL, Mulekar S, et al. Elevated levels of plasma mitochondrial DNA DAMPs are linked to clinical outcome in severely injured human subjects. *Ann Surg* 2013;258(4):591-6; discussion 96-8. doi: 10.1097/SLA.0b013e3182a4ea46

89. Yamanouchi S, Kudo D, Yamada M, et al. Plasma mitochondrial DNA levels in patients with trauma and severe sepsis: time course and the association with clinical status. *J Crit Care* 2013;28(6):1027-31. doi: 10.1016/j.jcrc.2013.05.006

90. Foster MA. Steroids and immunity from injury through to rehabilitation (SIR Study). University of Birmingham, 2013.

For peer review only

SPIRIT

STANDARD PROTOCOL ITEMS: RECOMMENDATIONS FOR INTERVENTIONAL TRIALS

SPIRIT 2013 Checklist: Recommended items to address in a clinical trial protocol and related documents*

Section/item	Item No	Description
**Administrative information**		
Title	1	Descriptive title identifying the study design, population, interventions, and, if applicable, trial acronym – Page 1
Trial registration	2a	Trial identifier and registry name. If not yet registered, name of intended registry – N/A
	2b	All items from the World Health Organization Trial Registration Data Set – N/A
Protocol version	3	Date and version identifier – Page 1
Funding	4	Sources and types of financial, material, and other support – Page 25
Roles and responsibilities	5a	Names, affiliations, and roles of protocol contributors – Pages 1, 25
	5b	Name and contact information for the trial sponsor – Page 25
	5c	Role of study sponsor and funders, if any, in study design; collection, management, analysis, and interpretation of data; writing of the report; and the decision to submit the report for publication, including whether they will have ultimate authority over any of these activities – Page 25
	5d	Composition, roles, and responsibilities of the coordinating centre, steering committee, endpoint adjudication committee, data management team, and other individuals or groups overseeing the trial, if applicable (see Item 21a for data monitoring committee) – Page 25
**Introduction**		
Background and rationale	6a	Description of research question and justification for undertaking the trial, including summary of relevant studies (published and unpublished) examining benefits and harms for each intervention – Page 5 (introduction, page 6 (aims and objectives)
	6b	Explanation for choice of comparators – Supplementary file
Objectives	7	Specific objectives or hypotheses (Page 6)

For peer review only

Trial design	8	Description of trial design including type of trial (eg, parallel group, crossover, factorial, single group), allocation ratio, and framework (eg, superiority, equivalence, noninferiority, exploratory) – Page 7

Methods: Participants, interventions, and outcomes

Study setting	9	Description of study settings (eg, community clinic, academic hospital) and list of countries where data will be collected. Reference to where list of study sites can be obtained – Page 7
Eligibility criteria	10	Inclusion and exclusion criteria for participants. If applicable, eligibility criteria for study centres and individuals who will perform the interventions (eg, surgeons, psychotherapists) – Pages 8-10
Interventions	11a	Interventions for each group with sufficient detail to allow replication, including how and when they will be administered – N/A
	11b	Criteria for discontinuing or modifying allocated interventions for a given trial participant (eg, drug dose change in response to harms, participant request, or improving/worsening disease) – N/A
	11c	Strategies to improve adherence to intervention protocols, and any procedures for monitoring adherence (eg, drug tablet return, laboratory tests) – N/A
	11d	Relevant concomitant care and interventions that are permitted or prohibited during the trial – N/A
Outcomes	12	Primary, secondary, and other outcomes, including the specific measurement variable (eg, systolic blood pressure), analysis metric (eg, change from baseline, final value, time to event), method of aggregation (eg, median, proportion), and time point for each outcome. Explanation of the clinical relevance of chosen efficacy and harm outcomes is strongly recommended – Page 10 (primary outcome), Supplementary file (candidate predictors)
Participant timeline	13	Time schedule of enrolment, interventions (including any run-ins and washouts), assessments, and visits for participants. A schematic diagram is highly recommended (see Figure) – Pages 8-10
Sample size	14	Estimated number of participants needed to achieve study objectives and how it was determined, including clinical and statistical assumptions supporting any sample size calculations – Pages 14-15
Recruitment	15	Strategies for achieving adequate participant enrolment to reach target sample size – Pages 14-15

Methods: Assignment of interventions (for controlled trials)

Allocation:

Sequence generation	16a	Method of generating the allocation sequence (eg, computer-generated random numbers), and list of any factors for stratification. To reduce predictability of a random sequence, details of any planned restriction (eg, blocking) should be provided in a separate document that is unavailable to those who enrol participants or assign interventions – N/A
Allocation concealment mechanism	16b	Mechanism of implementing the allocation sequence (eg, central telephone; sequentially numbered, opaque, sealed envelopes), describing any steps to conceal the sequence until interventions are assigned – N/A
Implementation	16c	Who will generate the allocation sequence, who will enrol participants, and who will assign participants to interventions – N/A
Blinding (masking)	17a	Who will be blinded after assignment to interventions (eg, trial participants, care providers, outcome assessors, data analysts), and how N/A
	17b	If blinded, circumstances under which unblinding is permissible, and procedure for revealing a participant's allocated intervention during the trial – N/A

Methods: Data collection, management, and analysis

Data collection methods	18a	Plans for assessment and collection of outcome, baseline, and other trial data, including any related processes to promote data quality (eg, duplicate measurements, training of assessors) and a description of study instruments (eg, questionnaires, laboratory tests) along with their reliability and validity, if known. Reference to where data collection forms can be found, if not in the protocol – Pages 10-14, Supplementary file
	18b	Plans to promote participant retention and complete follow-up, including list of any outcome data to be collected for participants who discontinue or deviate from intervention protocols – Page 9 (withdrawals)
Data management	19	Plans for data entry, coding, security, and storage, including any related processes to promote data quality (eg, double data entry; range checks for data values). Reference to where details of data management procedures can be found, if not in the protocol – Page 14
Statistical methods	20a	Statistical methods for analysing primary and secondary outcomes. Reference to where other details of the statistical analysis plan can be found, if not in the protocol – Page 15-16
	20b	Methods for any additional analyses (eg, subgroup and adjusted analyses) – Page 16 (screening tool development and validation)

	20c	Definition of analysis population relating to protocol non-adherence (eg, as randomised analysis), and any statistical methods to handle missing data (eg, multiple imputation) – Page 15 (imputation)
**Methods: Monitoring**		
Data monitoring	21a	Composition of data monitoring committee (DMC); summary of its role and reporting structure; statement of whether it is independent from the sponsor and competing interests; and reference to where further details about its charter can be found, if not in the protocol. Alternatively, an explanation of why a DMC is not needed – N/A
	21b	Description of any interim analyses and stopping guidelines, including who will have access to these interim results and make the final decision to terminate the trial – N/A
Harms	22	Plans for collecting, assessing, reporting, and managing solicited and spontaneously reported adverse events and other unintended effects of trial interventions or trial conduct – N/A
Auditing	23	Frequency and procedures for auditing trial conduct, if any, and whether the process will be independent from investigators and the sponsor – N/A
**Ethics and dissemination**		
Research ethics approval	24	Plans for seeking research ethics committee/institutional review board (REC/IRB) approval – Pages 19-20
Protocol amendments	25	Plans for communicating important protocol modifications (eg, changes to eligibility criteria, outcomes, analyses) to relevant parties (eg, investigators, REC/IRBs, trial participants, trial registries, journals, regulators) – N/A
Consent or assent	26a	Who will obtain informed consent or assent from potential trial participants or authorised surrogates, and how (see Item 32) – Pages 9-10
	26b	Additional consent provisions for collection and use of participant data and biological specimens in ancillary studies, if applicable – N/A
Confidentiality	27	How personal information about potential and enrolled participants will be collected, shared, and maintained in order to protect confidentiality before, during, and after the trial – Page 14
Declaration of interests	28	Financial and other competing interests for principal investigators for the overall trial and each study site – Page 25
Access to data	29	Statement of who will have access to the final trial dataset, and disclosure of contractual agreements that limit such access for investigators – Not present

For peer review only - http://bmjopen.bmj.com/site/about/guidelines.xhtml

Ancillary and post-trial care	30	Provisions, if any, for ancillary and post-trial care, and for compensation to those who suffer harm from trial participation – N/A
Dissemination policy	31a	Plans for investigators and sponsor to communicate trial results to participants, healthcare professionals, the public, and other relevant groups (eg, via publication, reporting in results databases, or other data sharing arrangements), including any publication restrictions – N/A
	31b	Authorship eligibility guidelines and any intended use of professional writers – N/A
	31c	Plans, if any, for granting public access to the full protocol, participant-level dataset, and statistical code – N/A
**Appendices**		
Informed consent materials	32	Model consent form and other related documentation given to participants and authorised surrogates – N/A
Biological specimens	33	Plans for collection, laboratory evaluation, and storage of biological specimens for genetic or molecular analysis in the current trial and for future use in ancillary studies, if applicable – N/A

*It is strongly recommended that this checklist be read in conjunction with the SPIRIT 2013 Explanation & Elaboration for important clarification on the items. Amendments to the protocol should be tracked and dated. The SPIRIT checklist is copyrighted by the SPIRIT Group under the Creative Commons "Attribution-NonCommercial-NoDerivs 3.0 Unported" license.

For peer review only
